# Quantitative proteomic profiling of tumor-associated vascular endothelial cells in colorectal cancer

**DOI:** 10.1242/bio.042838

**Published:** 2019-04-29

**Authors:** Guoqiang Wang, Qiongzhi Yang, Maoyu Li, Ye Zhang, Yuxiang Cai, Xujun Liang, Ying Fu, Zhefeng Xiao, Minze Zhou, Zhongpeng Xie, Huichao Huang, Yahui Huang, Yongheng Chen, Qiongqiong He, Fang Peng, Zhuchu Chen

**Affiliations:** 1 NHC Key Laboratory of Cancer Proteomics, XiangYa Hospital, Central South University, Changsha, Hunan 410008, China; 2 Department of Pathology, XiangYa Hospital, Central South University, Changsha, Hunan 410008, China; 3 Department of Pathology, School of Basic Medical, Central South University, Changsha 410008, China

**Keywords:** Colorectal cancer, Quantitative proteomics, Angiogenesis, Tenascin-C

## Abstract

To investigate the global proteomic profiles of vascular endothelial cells (VECs) in the tumor microenvironment and antiangiogenic therapy for colorectal cancer (CRC), matched pairs of normal (NVECs) and tumor-associated VECs (TVECs) were purified from CRC tissues by laser capture microdissection and subjected to iTRAQ-based quantitative proteomics analysis. Here, 216 differentially expressed proteins (DEPs) were identified and used for bioinformatics analysis. Interestingly, these proteins were implicated in epithelial mesenchymal transition (EMT), ECM-receptor interaction, focal adhesion, PI3K-Akt signaling pathway, angiogenesis and HIF-1 signaling pathway, which may play important roles in CRC angiogenesis. Among these DEPs we found that Tenascin-C (TNC) was upregulated in TVECs of CRC and correlated with CRC multistage carcinogenesis and metastasis. Furthermore, the reduction of tumor-derived TNC could attenuate human umbilical vein endothelial cell (HUVEC) proliferation, migration and tube formation through ITGB3/FAK/Akt signaling pathway. Based on the present work, we provided a large-scale proteomic profiling of VECs in CRC with quantitative information, a certain number of potential antiangiogenic targets and a novel vision in the angiogenesis bio-mechanism of CRC.

## Abstract (precis)

**Summary:** We provided large-scale proteomic profiling of vascular endothelial cells in colorectal cancer with quantitative information, a number of potential antiangiogenic targets and a novel vision in the angiogenesis bio-mechanism of CRC.

## INTRODUCTION

Tumor angiogenesis plays a vital role in creating the tumor microenvironment and is necessary for tumor growth and metastasis. Currently, angiogenesis inhibitors have become important drugs in the treatment of solid tumors, including colorectal cancer (CRC) (Lopez et al., 2019; Riechelmann and Grothey, 2017). Vascular endothelial cells (VECs), which interact with tumor cells, extracellular matrix and immune killer cells, form the major components of tumor microenvironment. Recently antiangiogenic strategies have largely focused on targeting VECs (Liu et al., 2016, 2015). Tumor-associated VECs (TVECs) undergo phenotypic and epigenetic changes during tumor initiation and progression (Xiong et al., 2009). Increasing evidence indicates that proteins are primary targets of therapeutic drugs, and that proteins located in TVECs are potential therapeutic targets against tumor angiogenesis and widely used in screening antiangiogenic drugs (Kalén et al., 2009; Sonveaux, 2008).

Proteomics has introduced an effective cancer research method that provides new opportunities for discovering the therapeutic targets of CRC and for revealing the underlying molecular mechanism of this disease. The proteomics of CRC have been extensively studied (Álvarez-Chaver et al., 2018; Peng et al., 2016), but relatively few studies have focused on tumor microenvironment, especially the proteome of VECs in CRC. However, the VEC comprises only a relatively small percentage of the total tumor volume. Consequently, an altered VEC expression signature may be easily masked in whole-tumor studies. To overcome this limitation, laser capture microdissection (LCM) was exploited to isolate a relatively pure VECs from heterogeneous frozen tumor tissue samples in this study.

Tenascin-C (TNC), a large extracellular matrix protein, is highly expressed in pathological contexts, especially in cancer (Midwood et al., 2011). The high expression of TNC correlates with poor prognosis in several cancer types, including breast cancer, glioblastoma and colorectal cancer (Midwood et al., 2016). It has been clearly demonstrated that TNC promotes multiple events in cancer progression, such as tumor cell survival, proliferation, invasion and lung metastasis (Sun et al., 2018). Moreover, high TNC levels are also correlated with angiogenic switch, higher tumor vessel density and vessel leakiness (Midwood et al., 2016), suggesting that TNC plays multiple roles in angiogenesis.

We compared the protein expression level between CRC tumor VECs and matched adjacent nonmalignant colorectal (ANC) tissue VECs from the same patient. This approach has the advantage of eliminating some of the inherent heterogeneity between individual patients and between different cell types presented in the samples (Johann et al., 2010; Unwin et al., 2003). The resulting peptides of the enzymatically digested proteins were measured by iTRAQ-based quantitative proteomics. Compared to conventional proteomic technology, this approach possesses many advantages, such as high throughput, high accuracy, high repeatability and high sensitivity, and it can be used for various types of biological samples (Pierce et al., 2008). A total of 216 differentially expressed proteins (DEPs) were identified. TNC was further validated by immunohistochemical analysis, and the effects of TNC on human umbilical vein endothelial cells (HUVECs) proliferation, migration and tube formation were determined. Based on our data, we presented the proteome of VECs in CRC using LCM technology and quantitative proteomics analysis. These TVECs-related proteins may serve as potential therapeutic targets and increase our understanding of the of CRC angiogenesis mechanisms.

## RESULTS

### Proteomic profiling of VECs in CRC

**Fig. 1.**
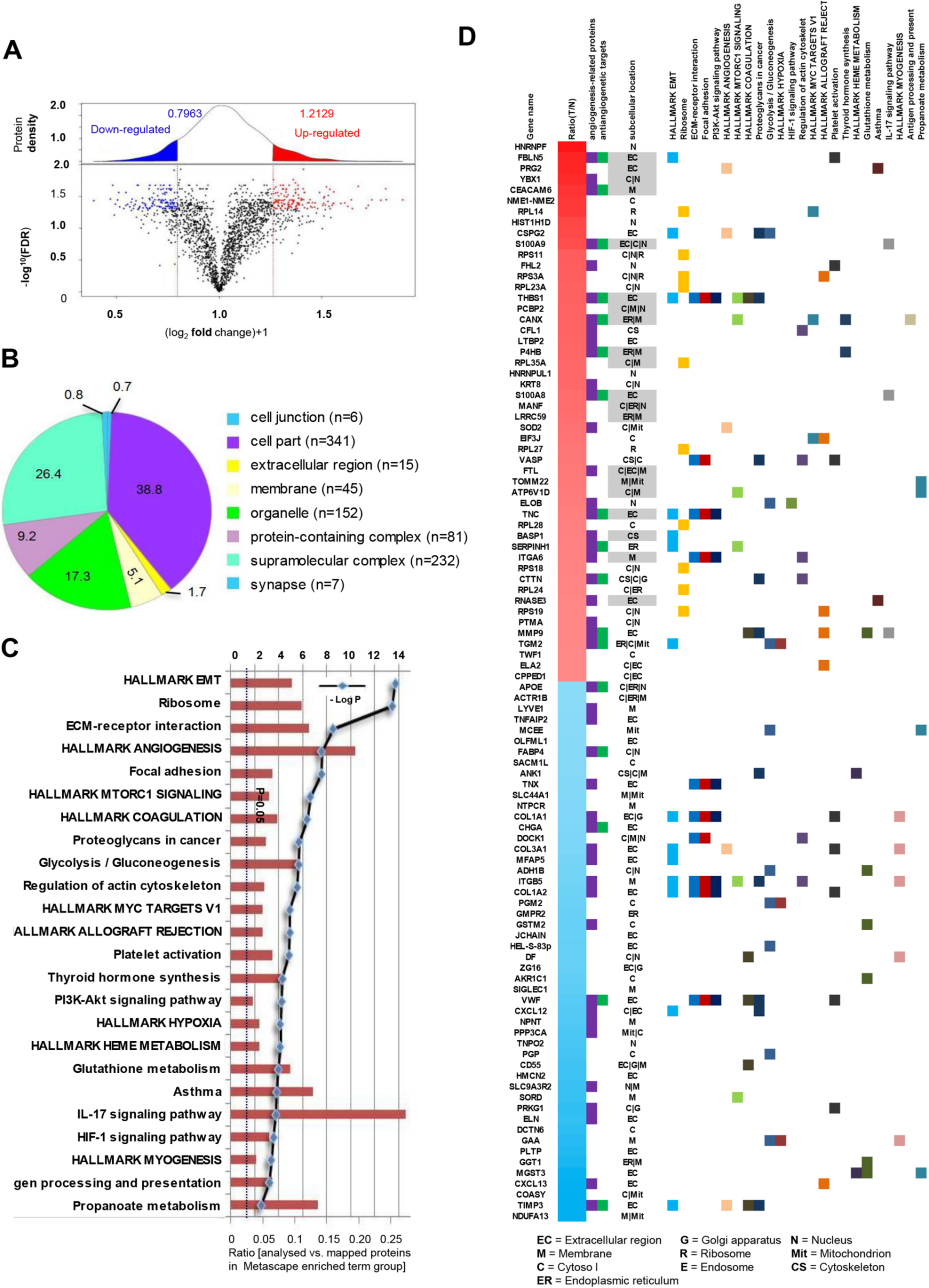
**Integrative analysis of identified proteins.** (A) The ratio intensity plot representing protein fold change (iTRAQ ratio versus corresponding summed peptide intensity distribution) and protein density plot (upper panel). Red, blue and black clusters indicate up-, down- and unregulated proteins, respectively. (B) A total of 2058 proteins was classified according to the cell components with PANTHER. (C) Metascape pathway analysis mapped the DEPs to 24 signaling pathways. (D) Heatmap of top 50 up- and top 50 downregulated proteins mapping to each Metascape pathway terms group. The subcellular location of each protein is also presented and upregulated proteins that are either secreted or membrane are highlighted in gray as potential therapeutic targets.

We compared the global proteomic profiles of paired TVECs and NVECs in ten patients with CRC to identify proteins and pathways that may be associated with the CRC angiogenesis. As a result, A total of 2058 non-redundant proteins were repeatedly identified and quantified at a minimum confidence level of 95% (unused ProtScore >1.3) by triplicate iTRAQ labeling and 2D LC-MS/MS analyses (Table S1). A protein density plot was subsequently generated to determine the thresholds for clustering DEPs using the ratios of those quantified proteins (Chakraborty et al., 2015). Using 10%, 90% and in-between quantile-based thresholds, averaged ratio-fold changes >1.2129 or <0.7963 between two cohorts of proteins were categorized as upregulated and downregulated proteins, respectively (Fig. 1A).

A total of 216 proteins were found to be differentially expressed, including 119 upregulated proteins and 97 downregulated proteins in TVECs relative to NVECs (Table S2). Compared to previous literature, 45 of the top 100 DEPs (top 50 up- and top 50 downregulated proteins) have been proved as angiogenesis-related proteins, and 17 of the 45 proteins have been reported or predicted as potential targets for cancer antiangiogenic treatment. These proteins are presented in a heatmap format in Fig. 1D. In addition, the top 50 upregulated proteins that are either secreted or localized in the membrane are highlighted in gray in the heatmap as potential therapeutic targets in TVECs (gene names of the respective proteins are: FBLN5, PRG2, YBX1, CEACAM6, S100A9, THBS1, PCBP2, CANX, P4HB, RPL35A, S100A8, MANF, LRRC59, FTL, TOMM22, ATP6V1D, TNC, BASP1 and RNASE3) (Fig. 1D). To some degree, these results indicate that our findings were consistent with previous studies and also make new discoveries.

### Bioinformatics analysis

**Fig. 2.**
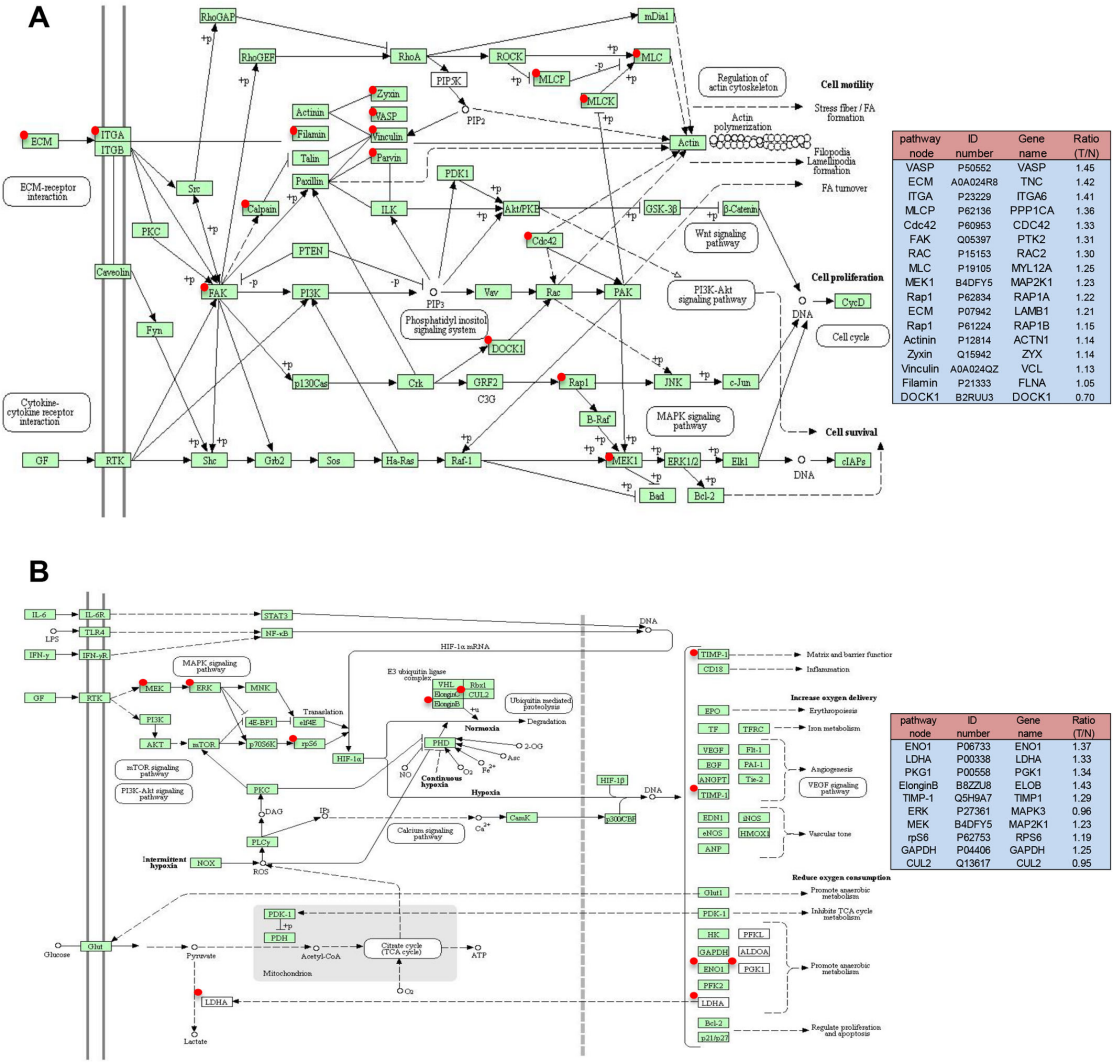
**Focal adhesion pathway (A) and HIF-1 signaling pathway (B) altered in a CRC.** Green rectangles with a red mark indicate the identified proteins. Green rectangles without a red mark indicate species-specific enzymes. White rectangles indicate reference pathway. The solid line indicates molecular interaction. The dashed line indicates indirect effect. The pathway node in the right panel corresponds to the red marked node in the left diagram. ID number is the Swiss-Prot accession number. Ratio (T/N)=Ratio of TVECs to controls.

To obtain a biological view of the identified proteins, a total of 2058 proteins were classified according to cellular compartment levels using the PANTHER GO classification system. Variable cellular compartments cell part (38.8%), organelle (26.4%) and membrane (5.1%) are shown in (Fig. 1B). Metascape enrichment analysis, accounting for all the DEPs, demonstrated that processes related EMT, ECM-receptor interaction, focal adhesion, PI3K-Akt signaling pathway, angiogenesis and HIF-1 signaling pathway were over-represented (Fig. 1C; Table S3). The top 50 upregulated and downregulated proteins mapping to each Metascape enrichment term group are presented in a heatmap format in Fig. 1D. The subcellular localization of these proteins is also presented in the heatmap. Moreover, Metascape enrichment analysis mapped all the identified proteins to 66 signaling pathways (Table S4). Interestingly, the results demonstrated that TVECs exhibited an increased dependence on processes related to focal adhesion, PI3K-Akt signaling pathway, HIF-1 signaling pathway and EMT (Fig. 2A,B; Fig. S1, Table S5), which might play an important role in CRC angiogenesis.

### Validation of the expression of TNC in VECs using immunohistochemistry

**Fig. 3.**
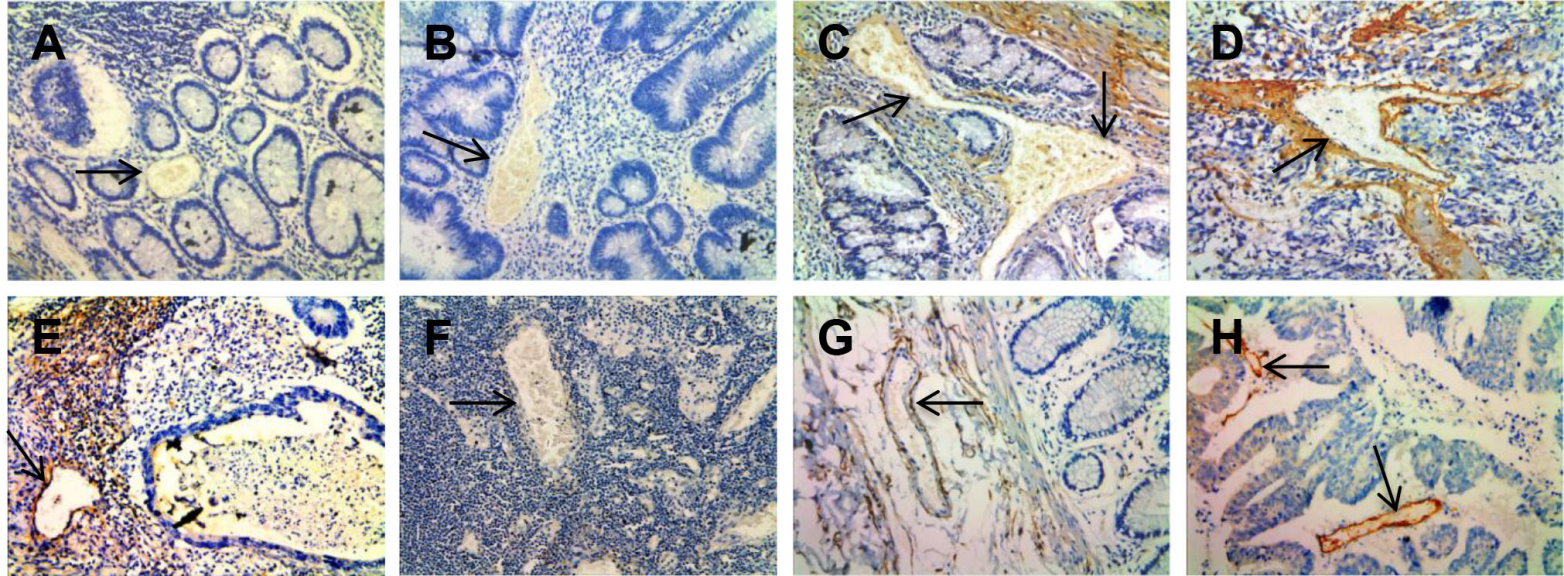
**Representative results of immunohistochemistry show the expression of TNC in vessels.** Original magnification: ×200. Top panel: TNC immunostaining of NCM (A), ACP (B), CIS (C) and ICC (D). Negative staining was observed in VECs of NCM and ACP, moderate staining in CIS, and strong staining in ICC. Bottom panel: strong staining was observed in VECs of lymph nodes with metastasis (E). Negative staining was observed in VECs of lymph nodes without metastasis (F). Endothelial cell marker (anti-CD34) immunostaining of NCM (G) and ICC (H). The arrows indicate the vessels.

In our study, we found TNC was involved in focal adhesion, PI3K/Akt signaling pathway and EMT. To confirm the expression and location of TNC in CRC tissues, we detected the expression of TNC using immunohistochemistry in 30 cases of non-neoplastic colonic mucosa (NCM), 30 cases of adenomatous colorectal polyps (AD), 30 cases of colorectal carcinoma *in situ* (CIS) and 50 cases of invasive colorectal carcinoma (ICC). As shown in Fig. 3 and Table 1, the expression levels of TNC were progressively increased during the CRC carcinogenic process from early stage, AD, to late stage, ICC. (*P*<0.05). Strong TNC immunostaining was readily detected in the stoma and VECs of the CIS and ICC (Fig. 3A,B), whereas there was weak staining in AD and negative staining in NCM (Fig. 3C,D). In addition, the expression levels of TNC in VECs of lymph nodes with metastasis were stronger compared to those in lymph nodes without metastasis (Fig. 3E,F). Immunostaining of NCM and ICC for the EC marker CD34 was used as a positive control for VECs (Fig. 3G,H).

**Table 1.**
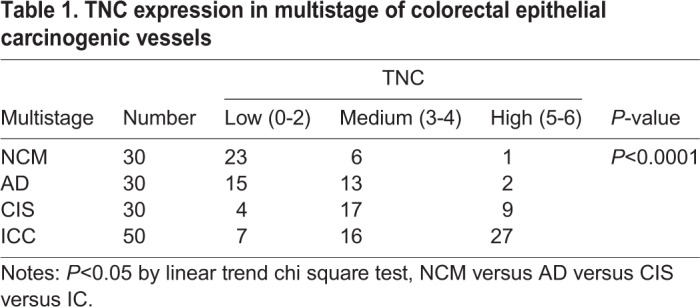
**TNC expression in multistage of colorectal epithelial carcinogenic vessels**

Furthermore, we examined the relationship between the expression levels of TNC and clinico-pathological characteristics in the 80 cases of CRC tissues above (30 cases of CIS and 50 cases of ICC). The results showed that TNC expression levels in VECs of CRC were closely correlated with lymph nodes metastasis (*P*<0.05) and distant metastasis (*P*<0.01) but did not correlate with age or gender (*P*>0.05; Table 2).

**Table 2.**
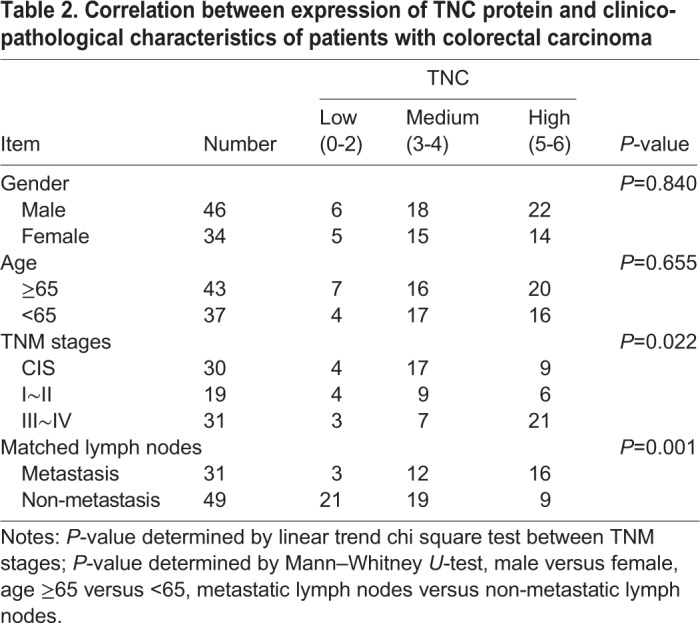
**Correlation between expression of TNC protein and clinico-pathological characteristics of patients with colorectal carcinoma**

### Reduction of tumor-derived TNC inactivates ITGB3/FAK/Akt signaling

**Fig. 4.**
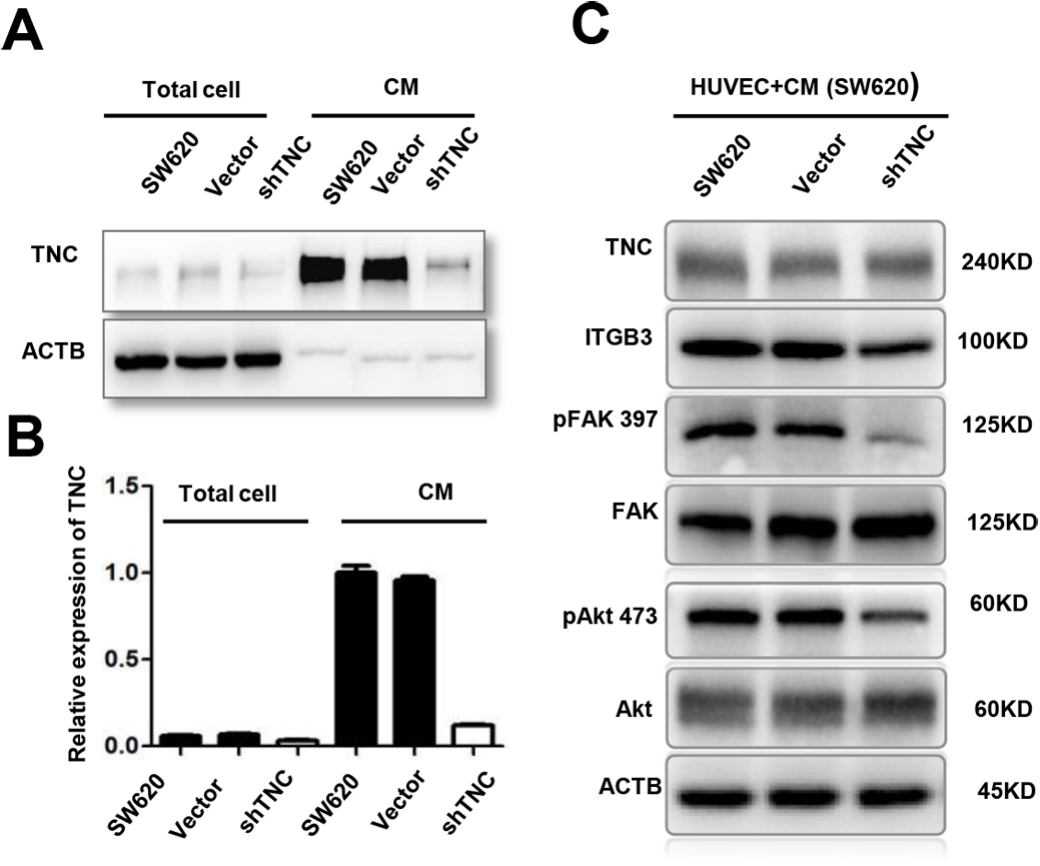
**Reduction of tumor-derived TNC inactivate ITGB3/FAK/Akt signaling in HUVECs.** (A,B) TNC protein levels in total cell and conditioned medium from untransfected (SW620), empty vector (SW620/Vector) and TNC-shRNA plasmid transfected SW620 cells (SW620/shTNC) were detected by western blot analysis. (C) Treatment with conditioned media (CM) from SW620/sh-TNC cells decreased ITGB3, phosphorylation of FAK-397 and phosphorylation of Akt-473 in HUVECs as compared with CM from untransfected SW620 cells.

Tumor-derived TNC promotes or enhances the process of angiogenesis in different tumor models (Hirata et al., 2009; Kawamura et al., 2018; Pezzolo et al., 2011; Rupp et al., 2016), but the mechanistic insight has not been fully elucidated. In our previous study, the TNC level is much higher in high metastatic potential CRC cell line SW620 compared to the other four CRC cell lines, particularly in the conditioned medium (Li et al., 2016). In the current study, we generated CRC cell line SW620 with knockdown of TNC (Fig. 4A,B), then collected conditioned media from SW620, SW620/Vector and SW620/shTNC cells and subsequently cultured HUVECs with the conditioned media for 24 h. The results indicated that conditioned media from SW620/shTNC cells reduced the expression of ITGB3, Phospho-FAK Tyr397, Phospho-Akt Ser473 (Fig. 4C); however, it did not alter the expression of TNC (Fig. 4C). These results demonstrated that tumor-derived TNC has a positive influence on ITGB3/FAK/Akt signaling pathway.

### Reduction of tumor-derived TNC impairs tubulogenesis, proliferation and migration of HUVECs *in vitro*

**Fig. 5.**
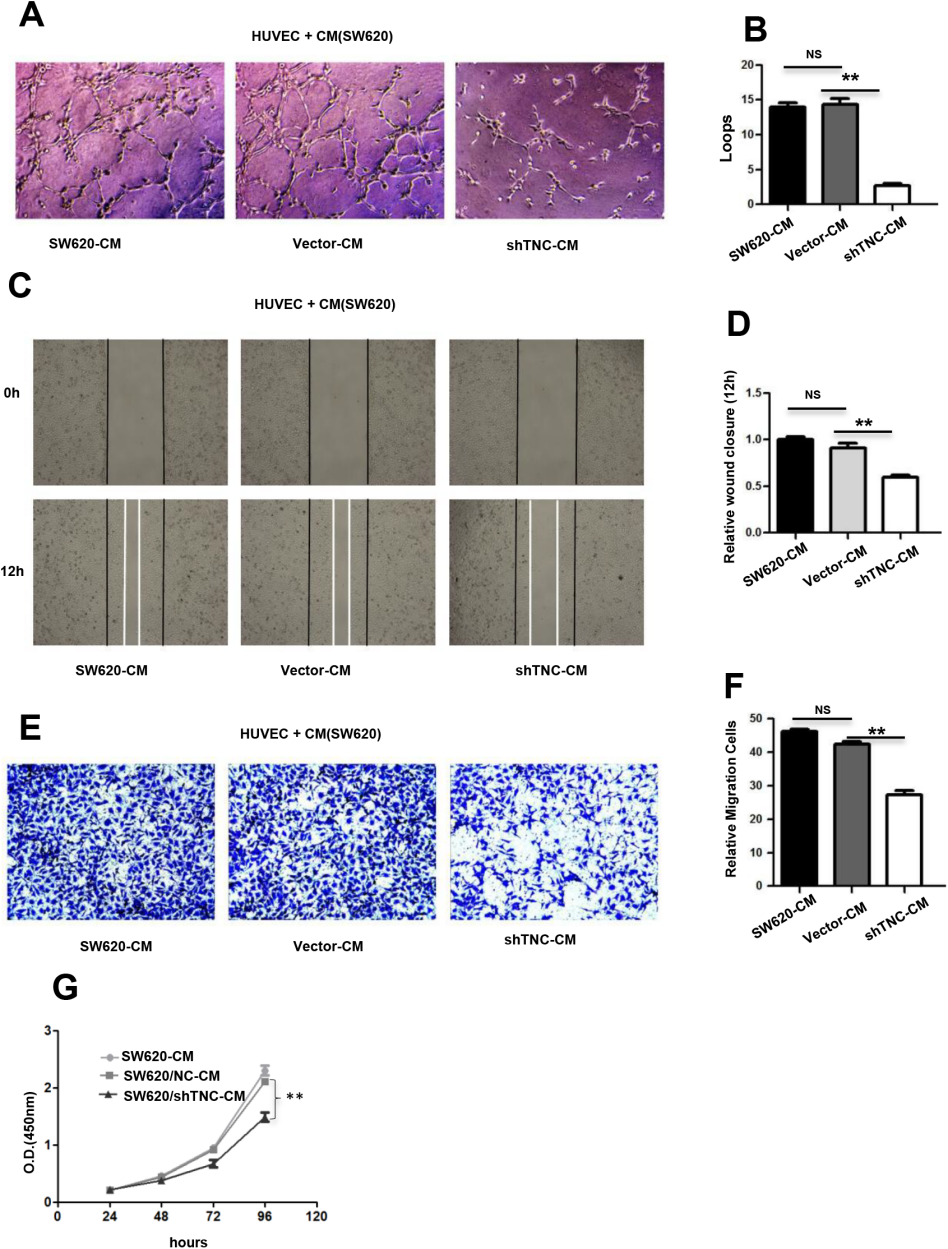
**Reduction of SW620-derived TNC** **leads to the decrease in proliferation, tubulogenesis and migration of HUVECs *in vitro*.** (A,B) Representative images (A) of HUVECs 7 h after plating on Matrigel together with conditioned medium from untransfected (SW620-CM) cells and empty vector (Vector-CM)- and TNC-shRNA plasmid-transfected SW620 cells (shTNC-CM) followed by quantification of the number of endothelial closed loops (B). Values are mean±s.e.m. from three independent experiments with three replicates. (C,D) Wound closure of HUVECs at 12 h was quantified upon addition of SW620-CM, Vector-CM and shTNC-CM. Values are the mean±s.e.m. from three independent experiments with three replicates. (E,F) Representative pictures (E) and quantification (F) of HUVEC migration through a transwell chamber containing SW620-CM, Vector-CM and shTNC-CM after 24 h. Values are the mean±s.e.m. from three independent experiments with three replicates. The images in A,C,E were presented under a microscope at 100x magnification. (G) CCK8 assay of HUVECs after treatment with SW620-CM, Vector-CM and shTNC-CM up to 96 h. Values are the mean±s.e.m. in HUVECs from three independent experiments with five replicates.

To further confirm whether TNC has an impact on tubulogenesis activity of HUVECs, we plated HUVECs on matrigel together with conditioned media from SW620, SW620/Vector and SW620/shTNC cells, respectively. As shown in Fig. 5A and B, when TNC knockdown, the incubation of HUVECs with SW620/shTNC conditioned media resulted in a 60% decrease in the formation of capillary-like structures compared with the tubules formed by HUVECs incubated with SW620 conditioned media, and similar results were obtained in cell proliferation and migration of HUVECs using CCK8, wound-healing and transwell chamber assays (Fig. 5C–G). Our results suggested that TNC has a positive influence on the proliferation, migration and tubulogenesis of HUVECs.

## DISCUSSION

Tumor angiogenesis is a complex process leading to abnormalities in vascular structure and function (Sasaki et al., 1991). Similar to vasculature in individual organs, TVECs are tissue-specific, which mostly depends on the tumor microenvironment (Jin et al., 2016). In this study, we compared global proteome profiles of VECs derived from normal and tumor tissues to gain an insight into CRC angiogenesis and discover antiangiogenic targets for tumor therapy. A total of 216 DEPs were identified, and then GO analysis and systematic pathway-based enrichment analysis were performed. Among the DEPs, many have been reported or predicted as potential targets for cancer antiangiogenic treatment (see Fig. 1D and Table S4), such as FBLN5 (Albig and Schiemann, 2004), CEACAM6 (Zang et al., 2015), S100A9 (Eisenblaetter et al., 2017; Zhang et al., 2017), THBS1 (Lawler, 2002), CANX (Demeure et al., 2016), TNC (Kawamura et al., 2018), HSP47 (Wu et al., 2016), CTTN (Ramos-Garcia and Gonzalez-Moles, 2018), MMP9 (Gupta et al., 2013), TGM2 (Lei et al., 2018), S100A7 (Padilla et al., 2017), LCN2 (Hu et al., 2018), RACK1 (Wang et al., 2011), PGK1 (Shichijo et al., 2004), EPO (Samoszuk et al., 1996), CD74 (Gai et al., 2018) and GRP78 (Kao et al., 2018). Regarding these candidate antiangiogenic targets, our results are consistent with previously published data. Furthermore, the focal adhesion, PI3K-Akt signaling pathway, HIF-1 signaling pathway and EMT were identified as significantly and consistently proangiogenic categories in CRC as these pathway-related proteins were significantly upregulated in TVECs compared to controls.

Focal adhesion is a subcellular structure which acts as a scaffold for many signaling pathways involving integrin or the mechanical force exerted on cells (Shen et al., 2018). Many molecules in the focal adhesion complex are implicated in downstream signaling pathways, such as the AKT1 (Higuchi et al., 2013), MAPK/ERK pathway (Ye et al., 2017) and Wnt signaling (Yu et al., 2012). In this way, pathways impacted by the focal adhesion complex are as varied as apoptosis (Bouchard et al., 2008), cell proliferation (Luo et al., 2018), cell migration and angiogenesis (Zhao and Guan, 2011). In our findings, the focal adhesion-related proteins were significantly increased in TVECs as compared to controls (Fig. 2A), which indicated that the focal adhesion pathway may play a crucial role in CRC angiogenesis. Therefore, uncovering the molecular processes underlying focal adhesion hub signaling will foster the development of reasonable and feasible multimodal treatment options towards CRC.

The role of PI3K/Akt signaling pathway and HIF-1 signaling pathway in angiogenesis and tumor progression were well documented (Karar and Maity, 2011). Hypoxia leads to the stabilization of HIF-1α and is a major stimulus for tumor cells to increase the expression of VEGF. However, the activation of the PI3K/AKT pathway in tumor cells can also increase the secretion of VEGF. Moreover, PTEN/PI3K/AKT regulates the proteasome-dependent stability of HIF-1α under hypoxic conditions and controls tumor-induced angiogenesis and metastasis (Joshi et al., 2014). The HIF-1signaling pathway and PI3K signaling pathway have been exploited for the development of new cancer therapies (Post et al., 2004; Tanaka et al., 2015; Thorpe et al., 2015). In the current study, HIF-1 and PI3K/AKT signaling pathway-related proteins were significantly upregulated in TVECs compared with controls (Fig. 2B; Fig. S1). Those findings indicate that PI3K and HIF-1 inhibitors are excellent candidates for the treatment of CRC.

EMT underlies the progression and metastasis of malignant tumors by enabling cancer cells to depart from their primary tumors, invade surrounding tissues and spread to distant organs. Endothelial-to-mesenchymal transition (EndMT) is often categorized as a specialized form of EMT. Recent studies suggested that EndMT may play a role in angiogenic sprouting by enabling the so-called tip cells, which lead an emerging vascular plexus, to migrate into adjacent tissue (Potenta et al., 2008; Welch-Reardon et al., 2015). In our study, EMT-related proteins were significantly increased in TVECs as compared to controls (Table S5). For example, FBLN5 (fold change=1.81) initiates EMT and enhances EMT induced by TGF-beta in mammary epithelial cells via a MMP-dependent mechanism (Lee et al., 2008). Based on the clinical application of anti-angiogenesis therapy in metastatic CRC, the EMT-angiogenesis and EMT stemness links in the CRC cells, newly synthesized drugs with antiangiogenic/anti-EMT properties could be one of the medications used in the future in the targeted therapy of patients with CRC (Gurzu et al., 2016).

In our findings, TNC was implicated in focal adhesion, PI3K/Akt signaling pathway and EMT. Several researchers have demonstrated that TNC promotes neoplastic angiogenesis in various cancers including breast cancer, glioblastoma and oral and pharyngeal squamous cell carcinoma (Atula et al., 2003; Mai et al., 2002; Rupp et al., 2016). However, few reports have investigated the expression of TNC in the VECs of CRC and the mechanism for promoting CRC angiogenesis. In our study, we found that the expression of TNC in VECs was correlated with CRC multistage carcinogenesis. This finding suggested that TNC may have a positive effect on CRC angiogenesis and progression. As a following step, we highlighted the role of TNC in triggering ITGB3/FAK/Akt-473 signaling pathway, which promoted HUVEC migration and angiogenesis. Moreover, TNC can be secreted in large amounts by SW620 cells (Li et al., 2016), indicating that TNC is more likely to end up in the blood or other body fluids in a ‘measurable’ concentration in CRC patients, which can be used for blood-based diagnostics. Therefore, we demonstrated the potential regulation mechanism of TNC and indicated its possible use as a diagnostic and therapeutic target for CRC.

In conclusion, our study built the first proteome of VECs in CRC and investigated the role of vascular proteins in the process of colorectal angiogenesis. In addition, we demonstrated the function of TNC in regulating ITGB3/FAK/Akt signaling and promoting angiogenesis in human CRC. These findings not only provide the proteomic profiling of VECs in CRC, but also highlight some angiogenesis-related proteins, which may be the potential antiangiogenic targets and suggest new insights into the mechanism of angiogenesis in CRC.

## MATERIALS AND METHODS

### Sample collection

Ten fresh-frozen samples of CRC and ten matched ANC tissues were taken from the Department of Surgery, Xiangya Hospital, Central South University, China, and used for iTRAQ labeling. The patients received neither radiotherapy nor chemotherapy before curative surgery and provided a written informed consent form for the study, which was approved by the local ethical committee. All specimens were obtained from surgical resection and stored at −80°C until further investigation.

An independent set of formalin-fixed paraffin-embedded tissue specimens, namely, 30 cases of NCM, 30 cases of AD, 30 cases of CIS and 50 cases of ICC, were obtained from the Department of Pathology of the Xiangya Hospital at Central South University and used for immunohistochemical analysis.

### Laser capture microdissection

The frozen samples were prepared, stained and diagnosed by pathological examination and a rapid immunohistochemical staining technique was performed in less than 13 min with the DAKO rapid EnvisionTM–HRP system as described (Wu et al., 2010). To identify and visualize the VECs in human colorectal tissues for sequential LCM, the specific VEC marker CD31 was used for immunostaining. After the blood vessels were apparent, the glass slides were replaced with LCM-specific glass slides. The stained areas that had the characteristic morphology of VECs were captured by LCM. LCM was performed with a Leica AS LMD system to purify the cells of interest from each type of tissue as previously described (Cheng et al., 2008; Li et al., 2012). Each captured cell population was over 95% homogeneous as determined by direct microscopic visualization.

### Protein extraction

LCM-enriched VECs were dissolved with an addition of lysis buffer (7 mol/l urea, 2 mol/l thiourea, 65 mmol/l dithiothreitol, 0.1 mmol/l phenylmethylsulfonyl fluoride), and followed by centrifugation (12, 000 rpm, 30 min, 4°C). The protein content was determined using 2D Quantification Kit (GE Healthcare). To diminish the effects of biological variation on the proteomic results, equal amounts of proteins from ten cases of microdissected samples of CRC and ANC were pooled respectively.

### Trypsin digestion and labeling with iTRAQ reagents

Trypsin digestion and iTRAQ labelling were performed according to the manufacturer's protocol (Applied Biosystems). Briefly, 100 μg protein of each pooled sample was reduced, alkylated and then digested overnight at 37°C with trypsin (mass spectrometry grade; Promega). The peptide samples were collected and then labeled with iTRAQ reagents as follows: CRC, labeled with iTRAQ 116, 117 and 118; ANC, labeled with iTRAQ 113, 114 and 115. The labeled digests were then mixed and dried.

### Off-line 2D Liquid chromatography (LC)-electrospray ionization (ESI) tandem MS (MS/MS) analysis

The mixed peptides were first separated on a strong cation exchange column into ten fractions according to the procedure described in our previous study (Liu et al., 2017). Each fraction was dried, dissolved in buffer C (0.1% formic acid, 5% acetonitrile) and analyzed on a Q-Exactive mass spectrometer (Thermo Fisher Scientific, Waltham, MA, USA) in information-dependent mode. Briefly, peptides were separated on reverse-phase columns (EASY column, 10 cm, ID 75 μm, 3 μm, C18-A2) with an EASY-nLC™ system. Peptides were separated by a linear gradient mobile phase A (5% acetonitrile, 0.1% formic acid) and mobile phase B (84% acetonitrile, 0.1% formic acid) from 5% to 40% of mobile phase B in 60 min at a flow rate of 300 nl/min. MS data were acquired from 300–1800 m/z for fragmentation by higher-energy collisional dissociation, with up to 20 precursors selected for MS/MS and dynamic exclusion for 60 s. The target value was determined based on the predictive automatic gain control technique. Survey scans were acquired at a resolution of 70, 000 at m/z 200 and resolution for higher-energy collisional dissociation spectra was set to 17, 500 at m/z 200. Full MS automatic gain control (AGC) target was 1e6, maximum IT was 50 ms and isolation window was 2 m/z. The normalized collision energy was 30 eV and the underfill ratio was defined as 0.1%. The instrument was run with peptide recognition mode enabled.

### Data analysis

MS/MS spectra were searched using the MASCOT engine (Matrix Science, London, UK; version 2.2) embedded into Proteome Discoverer 1.4 (Thermo Fisher Scientific) against a Human protein sequence database (Uniprot_human_149633_20151117.fasta, 149633 sequences; downloaded on Nov 17, 2015). The peptide mass tolerance was set at ±20 ppm and the fragment tolerance mass was set at 0.1 Da. The data analysis parameters were set as follows: Digestion, Trypsin; Instrument, Q-Exactive systems; Species, Homo sapiens; fixed modification: carbamidomethyl (C), iTRAQ8plex (K), iTRAQ8plex (N-term); variable modification: oxidation (M); database pattern: target-Decoy; Max missed cleavages, 2; False discovery rate (FDR) Analysis, Yes; User Modified Parameter Files, No; Bias Correction, Auto; Background Correction, Yes. Identified proteins were grouped by the software to minimize redundancy. All peptides used for the calculation of protein ratios were unique to the given protein or proteins within the group, and peptides that were common to other isoforms or proteins of the same family were ignored.

The average iTRAQ ratios from the triple experiments were calculated for each protein. The confidence level of the altered expression of proteins was calculated by *t*-test as a *P*-value, which allows the results to be evaluated based on the confidence level of expression change.

### Bioinformatics analysis

The DEPs were first annotated by Gene Ontology (GO) using the PANTHER database (http://www.pantherdb.org/). Briefly, GO analysis was used to elucidate the genetic regulatory networks of interest by forming hierarchical categories according to the biological process, cellular component and molecular function aspects of the DEPs. Pathway analysis was performed to examine the significant pathways of the DEPs according to Metascape (http://metascape.org/gp/index.html#/main/step1).

### Immunohistochemistry

Immunohistochemistry was performed according to the procedure described in our previous study (Zeng et al., 2012). Briefly, 4-μm-thick tissue sections were deparaffinized, rehydrated, and treated with an antigen retrieval solution (10 mmol/l sodium citrate buffer, pH 6.0). The sections were incubated with anti-TNC (1:250; Abcam) or anti-CD34 (1:100; ZSGB-BIO) overnight at 4°C and then incubated with biotinylated secondary antibody followed by addition of avidin-biotin peroxidase. Finally, tissue sections were incubated with 3′,3′-diaminobenzidine until a brown color developed, and they were counterstained with Harris' modified Hematoxylin. In negative controls primary antibodies were omitted. The evaluation of immunostaining was performed as previously described (Zeng et al., 2012). A score (ranging from 0–6) was obtained for each case. A combined staining score of≤2 was considered to be weak staining (no/low expression), a score between 3 and 4 was considered to be moderate staining (expression), and a score between 5 and 6 was considered to be strong staining (high expression).

### Western blotting

For western blot analysis, the same amount (30 µg) of whole cell lysate samples or enriched conditioned media samples were subjected to SDS-PAGE and subsequently transferred to PVDF membranes. Membranes were blocked in 5% milk for 2 h prior to incubation with primary antibodies against TNC (1:1000; Santa Cruz Biotechnology, sc20932), ITGB3 (1:1000; Proteintech, 10309-1-AP), FAK (1:1000; Proteintech, 66258-1-Ig), Phospho-FAK Tyr397 (1:1000; Cell Signaling Technology, #8556), Akt (1:1000; Cell Signaling Technology, #4691P), Phospho-Akt Ser473 (1:1000; Cell Signaling Technology, #4060P) overnight at 4°C. ACTB was used as a control for protein loading and was detected using a mouse anti-ACTB antibody (1:2000; Proteintech, 20536-1-AP). Membranes were incubated with the corresponding secondary antibodies, including anti-rabbit and anti-mouse peroxidase (HRP)-linked IgG (1:1000; KPL) for 1 h.

### Cell culture, cell transfection and conditioned media preparation

Human colon cancer cell lines SW620 and HUVECs were maintained in RPMI-1640 or DMEM medium supplemented with 10% FBS in a humidified chamber with 5% CO_2_ at 37°C. SW620 cells and HUVECs were purchased from the Cell Bank of Type Culture Collection of the Chinese Academy of Sciences (Shanghai, China).

SW620 cells were cultured in six-well plates until reaching 70% of confluency, the cells were transduced with lentiviral shRNA-TNC (GGAGTACTTTATCCGTGTATT) and lentiviral control (Cyagen Bioscience Inc., Guangzhou, China) at an MOI of 20 in the presence of 5 μg/ml polybrene for 24 h and treated with 3 μg/ml puromycin for three days. The generated cell clones were tested for shRNA-TNC stable expression.

The untransfected (SW620), empty vector (SW620/Vector) and TNC shRNA plasmid-transfected SW620 (SW620/shTNC) cells were chosen for conditioned media preparation. Conditioned media were collected as previously described (Li et al., 2016). Briefly, approximately 3×10^6^ cells were grown to 70% confluency, and the medium was exchanged with serum-free RPMI-1640 medium. Conditioned media were collected 24 h after the change of media and stored at −20°C for use. For subsequent experiments, the conditioned media were filtered using a 0.22 μm filter (Millipore) and concentrated using a Millipore centrifugal filter (3 kDa). The protein concentration was determined by a standard Bradford protein assay (Thermo Fisher Scientific). After conditioned media were removed, the cell monolayer was washed, scraped and lysed in the presence of protease inhibitors. Protein concentration was determined using a Pierce BCA Protein Assay Kit (Thermo Fisher Scientific).

### HUVEC proliferation assay

The HUVECs were plated at 2×10^3^ cells per well in 96-well tissue culture plates and cultured in a 1:1 mixture of 10% FBS complete DMEM and conditioned media (total 200 μl). The cells were cultured for 6 days. Every 24 h, 20 μl of CCK8 (5 mg/ml; Beyotime) was added to the wells, and cells were further incubated for 2 h. The absorbance of each well was read with a Bio-Tek Instruments EL310 Microplate Autoreader at 450 nm. The CCK8 assay was performed three times in triplicate.

### Wound healing assay

Cell migration was determined by scratch wound healing assay. Briefly, cells were grown in a 1:1 mixture of 10% FBS complete DMEM and conditioned media overnight to confluence in a six-well plate. Monolayers of cells were wounded by dragging a pipette tip. Cells were washed to remove cellular debris and allowed to migrate for 12–24 h. Images were taken at 0 h and 12 h after wounding under the inverted microscope.

### HUVEC migration assay

Migration activity was measured by Transwell assay (Corning, 3422). Approximately 5×10^4^ HUVECs were added to the upper chamber in 200 μl of 1% FBS DMEM medium. The lower chamber contained 250 μl of 10% FBS complete DMEM and 250 μl of conditioned media. The plates were incubated for 24 h at 37°C in 5% CO_2_. After incubation at 37°C for 48 h, cells were fixed with 4% paraformaldehyde and stained with 0.5% Crystal Violet. Each clone was plated in triplicate for each experiment, and each experiment was repeated at least three times.

### HUVEC angiogenesis assay

Matrigel (BD Biosciences, 354248) was melted at 4°C, added to 48-multiwell plates (Corning) at 100 μl/well, and then incubated at 37°C for 30 min. HUVECs (4×10^3^ cells) were resuspended in a 1:1 mixture of 250 μl of 20% FBS complete DMEM and 250 μl of conditioned media (total 500 μl with 10% FBS). The cells were added to the wells, and after 7 h incubation at 37°C, HUVEC tube formation was assessed by microscopy. Each well was imaged under a light microscope. The numbers of branches were calculated and quantified using MacBiophotonics ImageJ software (NIH).

### Statistical analysis

All statistical analyses were performed using the SPSS software package (version 13.0; SPSS, Inc.) and Prism 5.0 software (GraphPad). Data are shown as the mean±s.e.m. The difference in TNC protein expression between the two different kinds of tissue (CRC versus ANC) was analyzed using a linear trend chi-square test. The relationship between TNC expression and the clinico-pathological characteristics in patients with CRC was analyzed using the Mann–Whitney *U*-test and linear trend chi-square test. For *in vitro* assays, the data are reported as biological replicates, with technical replicates indicated in the figure legends. Student's *t*-tests (unpaired, two-tailed) were performed to determine whether a difference between two values was statistically significant, with *P*<0.05 considered significant. *In vitro* assays were performed in triplicate unless otherwise stated. *P*-values <0.05 were considered statistically significant (*P<0.05; **P<0.01; ***P<0.001).

## Supplementary Material

Supplementary information

## References

[BIO042838C1] Albig A. R. and Schiemann W. P. (2004). Fibulin-5 antagonizes vascular endothelial growth factor (VEGF) signaling and angiogenic sprouting by endothelial cells. *DNA Cell Biol.* 23, 367-379. 10.1089/104454904323145254 15231070

[BIO042838C2] Álvarez-Chaver P., De Chiara L. and Martínez-Zorzano V. S. (2018). Proteomic profiling for colorectal cancer biomarker discovery. *Methods Mol. Biol.* 1765, 241-269. 10.1007/978-1-4939-7765-9_16 29589313

[BIO042838C3] Atula T., Hedstrom J., Finne P., Leivo I., Markkanen-Leppanen M. and Haglund C. (2003). Tenascin-C expression and its prognostic significance in oral and pharyngeal squamous cell carcinoma. *Anticancer Res.* 23, 3051-3056. 10.3892/or.16.3.485 12926160

[BIO042838C4] Bouchard V., Harnois C., Demers M.-J., Thibodeau S., Laquerre V., Gauthier R., Vézina A., Noël D., Fujita N., Tsuruo T. et al. (2008). B1 integrin/Fak/Src signaling in intestinal epithelial crypt cell survival: integration of complex regulatory mechanisms. *Apoptosis* 13, 531-542. 10.1007/s10495-008-0192-y 18322799

[BIO042838C5] Chakraborty S., Lakshmanan M., Swa H. L. F., Chen J., Zhang X., Ong Y. S., Loo L. S., Akincilar S. C., Gunaratne J., Tergaonkar V. et al. (2015). An oncogenic role of Agrin in regulating focal adhesion integrity in hepatocellular carcinoma. *Nat. Commun.* 6, 6184 10.1038/ncomms7184 25630468 PMC4317502

[BIO042838C6] Cheng A.-L., Huang W.-G., Chen Z.-C., Peng F., Zhang P.-F., Li M.-Y., Li F., Li J.-L., Li C., Yi H. et al. (2008). Identification of novel nasopharyngeal carcinoma biomarkers by laser capture microdissection and proteomic analysis. *Clin. Cancer Res.* 14, 435-445. 10.1158/1078-0432.CCR-07-1215 18223218

[BIO042838C7] Demeure K., Fack F., Duriez E., Tiemann K., Bernard A., Golebiewska A., Bougnaud S., Bjerkvig R., Domon B. and Niclou S. P. (2016). Targeted proteomics to assess the response to anti-angiogenic treatment in human glioblastoma (GBM). *Mol. Cell. Proteomics* 15, 481-492. 10.1074/mcp.M115.052423 26243272 PMC4739668

[BIO042838C8] Eisenblaetter M., Flores-Borja F., Lee J. J., Wefers C., Smith H., Hueting R., Cooper M. S., Blower P. J., Patel D., Rodriguez-Justo M. et al. (2017). Visualization of tumor-immune interaction-target-specific imaging of S100A8/A9 reveals pre-metastatic niche establishment. *Theranostics* 7, 2392-2401. 10.7150/thno.17138 28744322 PMC5525744

[BIO042838C9] Gai J. W., Wahafu W., Song L., Ping H., Wang M., Yang F., Niu Y., Qing W. and Xing N. (2018). Expression of CD74 in bladder cancer and its suppression in association with cancer proliferation, invasion and angiogenesis in HT-1376 cells. *Oncol. Lett.* 15, 7631-7638. 10.3892/ol.2018.8309 29731899 PMC5920967

[BIO042838C10] Gupta A., Zhou C. Q. and Chellaiah M. A. (2013). Osteopontin and MMP9: associations with VEGF expression/secretion and angiogenesis in PC3 prostate cancer cells. *Cancers* 5, 617-638. 10.3390/cancers5020617 24216994 PMC3730333

[BIO042838C11] Gurzu S., Silveanu C., Fetyko A., Butiurca V., Kovacs Z. and Jung I. (2016). Systematic review of the old and new concepts in the epithelial-mesenchymal transition of colorectal cancer. *World J. Gastroenterol.* 22, 6764-6775. 10.3748/wjg.v22.i30.6764 27570416 PMC4974578

[BIO042838C12] Higuchi M., Kihara R., Okazaki T., Aoki I., Suetsugu S. and Gotoh Y. (2013). Akt1 promotes focal adhesion disassembly and cell motility through phosphorylation of FAK in growth factor-stimulated cells. *J. Cell Sci.* 126, 745-755. 10.1242/jcs.112722 23264741

[BIO042838C13] Hirata E., Arakawa Y., Shirahata M., Yamaguchi M., Kishi Y., Okada T., Takahashi J. A., Matsuda M. and Hashimoto N. (2009). Endogenous tenascin-C enhances glioblastoma invasion with reactive change of surrounding brain tissue. *Cancer Sci.* 100, 1451-1459. 10.1111/j.1349-7006.2009.01189.x 19459858 PMC11158953

[BIO042838C14] Hu C., Yang K., Li M., Huang W., Zhang F. and Wang H. (2018). Lipocalin 2: a potential therapeutic target for breast cancer metastasis. *Onco Targets Ther.* 11, 8099-8106. 10.2147/OTT.S181223 30519052 PMC6239117

[BIO042838C15] Jin H., Cheng X., Pei Y., Fu J., Lyu Z., Peng H., Yao Q., Jiang Y., Luo L. and Zhuo H. (2016). Identification and verification of transgelin-2 as a potential biomarker of tumor-derived lung-cancer endothelial cells by comparative proteomics. *J. Proteomics* 136, 77-88. 10.1016/j.jprot.2015.12.012 26721444

[BIO042838C16] Johann D. J. Jr., Wei B. R., Prieto D. A., Chan K. C., Ye X., Valera V. A., Simpson R. M., Rudnick P. A., Xiao Z., Issaq H. J. et al. (2010). Combined blood/tissue analysis for cancer biomarker discovery: application to renal cell carcinoma. *Anal. Chem.* 82, 1584-1588. 10.1021/ac902204k 20121140 PMC3251958

[BIO042838C17] Joshi S., Singh A. R., Zulcic M. and Durden D. L. (2014). A macrophage-dominant PI3K isoform controls hypoxia-induced HIF1alpha and HIF2alpha stability and tumor growth, angiogenesis, and metastasis. *Mol. Cancer Res.* 12, 1520-1531. 10.1158/1541-7786.MCR-13-0682 25103499

[BIO042838C18] Kalén M., Wallgard E., Asker N., Nasevicius A., Athley E., Billgren E., Larson J. D., Wadman S. A., Norseng E., Clark K. J. et al. (2009). Combination of reverse and chemical genetic screens reveals angiogenesis inhibitors and targets. *Chem. Biol.* 16, 432-441. 10.1016/j.chembiol.2009.02.010 19389629 PMC3984492

[BIO042838C19] Kao C., Chandna R., Ghode A., Dsouza C., Chen M., Larsson A., Lim S. H., Wang M., Cao Z., Zhu Y. et al. (2018). Proapoptotic cyclic peptide BC71 targets cell-surface GRP78 and functions as an anticancer therapeutic in mice. *EBioMedicine* 33, 22-32. 10.1016/j.ebiom.2018.06.004 29907328 PMC6085501

[BIO042838C20] Karar J. and Maity A. (2011). PI3K/AKT/mTOR pathway in angiogenesis. *Front. Mol. Neurosci.* 4, 51 10.3389/fnmol.2011.00051 22144946 PMC3228996

[BIO042838C21] Kawamura T., Yamamoto M., Suzuki K., Suzuki Y., Kamishima M., Sakata M., Kurachi K., Setoh M., Konno H. and Takeuchi H. (2018). Tenascin-C produced by intestinal myofibroblasts promotes colitis-associated cancer development through angiogenesis. *Inflamm. Bowel Dis*. 25, 732-741. 10.1093/ibd/izy368 30517646

[BIO042838C22] Lawler J. (2002). Thrombospondin-1 as an endogenous inhibitor of angiogenesis and tumor growth. *J. Cell. Mol. Med.* 6, 1-12. 10.1111/j.1582-4934.2002.tb00307.x 12003665 PMC6740251

[BIO042838C23] Lee Y.-H., Albig A. R., Regner M., Schiemann B. J. and Schiemann W. P. (2008). Fibulin-5 initiates epithelial-mesenchymal transition (EMT) and enhances EMT induced by TGF-beta in mammary epithelial cells via a MMP-dependent mechanism. *Carcinogenesis* 29, 2243-2251. 10.1093/carcin/bgn199 18713838 PMC2621102

[BIO042838C24] Lei Z., Chai N., Tian M., Zhang Y., Wang G., Liu J., Tian Z., Yi X., Chen D., Li X. et al. (2018). Novel peptide GX1 inhibits angiogenesis by specifically binding to transglutaminase-2 in the tumorous endothelial cells of gastric cancer. *Cell Death Dis.* 9, 579 10.1038/s41419-018-0594-x 29785022 PMC5962530

[BIO042838C25] Li M., Li C., Li D., Xie Y., Shi J., Li G., Guan Y., Li M., Zhang P., Peng F. et al. (2012). Periostin, a stroma-associated protein, correlates with tumor invasiveness and progression in nasopharyngeal carcinoma. *Clin. Exp. Metastasis* 29, 865-877. 10.1007/s10585-012-9465-5 22706927

[BIO042838C26] Li M., Peng F., Li G., Fu Y., Huang Y., Chen Z. and Chen Y. (2016). Proteomic analysis of stromal proteins in different stages of colorectal cancer establishes Tenascin-C as a stromal biomarker for colorectal cancer metastasis. *Oncotarget* 7, 37226-37237. 10.18632/oncotarget.9362 27191989 PMC5095071

[BIO042838C27] Liu Y.-R., Guan Y.-Y., Luan X., Lu Q., Wang C., Liu H.-J., Gao Y.-G., Yang S.-C., Dong X., Chen H.-Z. et al. (2015). Delta-like ligand 4-targeted nanomedicine for antiangiogenic cancer therapy. *Biomaterials* 42, 161-171. 10.1016/j.biomaterials.2014.11.039 25542804

[BIO042838C28] Liu S., Liu J., Ma Q., Cao L., Fattah R. J., Yu Z., Bugge T. H., Finkel T. and Leppla S. H. (2016). Solid tumor therapy by selectively targeting stromal endothelial cells. *Proc. Natl. Acad. Sci. USA* 113, E4079-E4087. 10.1073/pnas.1600982113 27357689 PMC4948345

[BIO042838C29] Liu X., Wang J., Gao L., Liu H. and Liu C. (2017). iTRAQ-Based proteomic analysis of neonatal kidney from offspring of protein restricted rats reveals abnormalities in intraflagellar transport proteins. *Cell. Physiol. Biochem.* 44, 185-199. 10.1159/000484626 29130966

[BIO042838C30] Lopez A., Harada K., Vasilakopoulou M., Shanbhag N. and Ajani J. A. (2019). Targeting angiogenesis in colorectal carcinoma. *Drugs* 79, 63-74. 10.1007/s40265-018-1037-9 30617958

[BIO042838C31] Luo X., Guo L., Zhang L., Hu Y., Shang D. and Ji D. (2018). Bioinformatics analysis of microarray profiling identifies the mechanism of focal adhesion kinase signalling pathway in proliferation and apoptosis of breast cancer cells modulated by green tea polyphenol epigallocatechin 3-gallate. *J. Pharm. Pharmacol.* 70, 1606-1618. 10.1111/jphp.13010 30187481

[BIO042838C32] Mai J., Sameni M., Mikkelsen T. and Sloane B. F. (2002). Degradation of extracellular matrix protein tenascin-C by cathepsin B: an interaction involved in the progression of gliomas. *Biol. Chem.* 383, 1407-1413. 10.1515/BC.2002.159 12437133

[BIO042838C33] Midwood K. S., Hussenet T., Langlois B. and Orend G. (2011). Advances in tenascin-C biology. *Cell. Mol. Life Sci.* 68, 3175-3199. 10.1007/s00018-011-0783-6 21818551 PMC3173650

[BIO042838C34] Midwood K. S., Chiquet M., Tucker R. P. and Orend G. (2016). Tenascin-C at a glance. *J. Cell Sci.* 129, 4321-4327. 10.1242/jcs.190546 27875272

[BIO042838C35] Padilla L., Dakhel S., Adan J., Masa M., Martinez J. M., Roque L., Coll T., Hervas R., Calvis C., Llinas L. et al. (2017). S100A7: from mechanism to cancer therapy. *Oncogene* 36, 6749-6761. 10.1038/onc.2017.283 28825725

[BIO042838C36] Peng F., Huang Y., Li M. Y., Li G. Q., Huang H. C., Guan R., Chen Z. C., Liang S. P. and Chen Y. H. (2016). Dissecting characteristics and dynamics of differentially expressed proteins during multistage carcinogenesis of human colorectal cancer. *World J. Gastroenterol.* 22, 4515-4528. 10.3748/wjg.v22.i18.4515 27182161 PMC4858633

[BIO042838C37] Pezzolo A., Parodi F., Marimpietri D., Raffaghello L., Cocco C., Pistorio A., Mosconi M., Gambini C., Cilli M., Deaglio S. et al. (2011). Oct-4+/Tenascin C+ neuroblastoma cells serve as progenitors of tumor-derived endothelial cells. *Cell Res.* 21, 1470-1486. 10.1038/cr.2011.38 21403679 PMC3193450

[BIO042838C38] Pierce A., Unwin R. D., Evans C. A., Griffiths S., Carney L., Zhang L., Jaworska E., Lee C.-F., Blinco D., Okoniewski M. J. et al. (2008). Eight-channel iTRAQ enables comparison of the activity of six leukemogenic tyrosine kinases. *Mol. Cell. Proteomics* 7, 853-863. 10.1074/mcp.M700251-MCP200 17951628

[BIO042838C39] Post D. E., Devi N. S., Li Z., Brat D. J., Kaur B., Nicholson A., Olson J. J., Zhang Z. and Van Meir E. G. (2004). Cancer therapy with a replicating oncolytic adenovirus targeting the hypoxic microenvironment of tumors. *Clin. Cancer Res.* 10, 8603-8612. 10.1158/1078-0432.CCR-04-1432 15623644

[BIO042838C40] Potenta S., Zeisberg E. and Kalluri R. (2008). The role of endothelial-to-mesenchymal transition in cancer progression. *Br. J. Cancer* 99, 1375-1379. 10.1038/sj.bjc.6604662 18797460 PMC2579683

[BIO042838C41] Ramos-Garcia P. and Gonzalez-Moles M. A. (2018). An update of knowledge on cortactin as a metastatic driver and potential therapeutic target in oral squamous cell carcinoma. *Oral Dis.* 25, 949-971. 10.1111/odi.12913 29878474

[BIO042838C42] Riechelmann R. and Grothey A. (2017). Antiangiogenic therapy for refractory colorectal cancer: current options and future strategies. *Ther. Adv. Med. Oncol.* 9, 106-126. 10.1177/1758834016676703 28203302 PMC5298403

[BIO042838C43] Rupp T., Langlois B., Koczorowska M. M., Radwanska A., Sun Z., Hussenet T., Lefebvre O., Murdamoothoo D., Arnold C., Klein A. et al. (2016). Tenascin-C orchestrates glioblastoma angiogenesis by modulation of pro- and anti-angiogenic signaling. *Cell Rep.* 17: 2607-2619. 10.1016/j.celrep.2016.11.012 27926865

[BIO042838C44] Samoszuk M., Lin F., Rim P. and Strathearn G. (1996). New marker for blood vessels in human ovarian and endometrial cancers. *Clin. Cancer Res.* 2, 1867-1871. 9816142

[BIO042838C45] Sasaki K., Kiuchi Y., Sato Y. and Yamamori S. (1991). Morphological analysis of neovascularization at early stages of rat splenic autografts in comparison with tumor angiogenesis. *Cell Tissue Res.* 265, 503-510. 10.1007/BF00340873 1723929

[BIO042838C46] Shen J., Cao B., Wang Y., Ma C., Zeng Z., Liu L., Li X., Tao D., Gong J. and Xie D. (2018). Hippo component YAP promotes focal adhesion and tumour aggressiveness via transcriptionally activating THBS1/FAK signalling in breast cancer. *J. Exp. Clin. Cancer Res.* 37, 175 10.1186/s13046-018-0850-z 30055645 PMC6064138

[BIO042838C47] Shichijo S., Azuma K., Komatsu N., Ito M., Maeda Y., Ishihara Y. and Itoh K. (2004). Two proliferation-related proteins, TYMS and PGK1, could be new cytotoxic T lymphocyte-directed tumor-associated antigens of HLA-A2+ colon cancer. *Clin. Cancer Res.* 10, 5828-5836. 10.1158/1078-0432.CCR-04-0350 15355913

[BIO042838C48] Sonveaux P. (2008). Provascular strategy: targeting functional adaptations of mature blood vessels in tumors to selectively influence the tumor vascular reactivity and improve cancer treatment. *Radiother. Oncol.* 86, 300-313. 10.1016/j.radonc.2008.01.024 18313779

[BIO042838C49] Sun Z., Schwenzer A., Rupp T., Murdamoothoo D., Vegliante R., Lefebvre O., Klein A., Hussenet T. and Orend G. (2018). Tenascin-C promotes tumor cell migration and metastasis through integrin alpha9beta1-mediated YAP inhibition. *Cancer Res.* 78, 950-961. 10.1158/0008-5472.CAN-17-1597 29259017 PMC5901716

[BIO042838C50] Tanaka T., Li T.-S., Urata Y., Goto S., Ono Y., Kawakatsu M., Matsushima H., Hirabaru M., Adachi T., Kitasato A. et al. (2015). Increased expression of PHD3 represses the HIF-1 signaling pathway and contributes to poor neovascularization in pancreatic ductal adenocarcinoma. *J. Gastroenterol.* 50, 975-983. 10.1007/s00535-014-1030-3 25542265 PMC4561234

[BIO042838C51] Thorpe L. M., Yuzugullu H. and Zhao J. J. (2015). PI3K in cancer: divergent roles of isoforms, modes of activation and therapeutic targeting. *Nat. Rev. Cancer* 15, 7-24. 10.1038/nrc3860 25533673 PMC4384662

[BIO042838C52] Unwin R. D., Craven R. A., Harnden P., Hanrahan S., Totty N., Knowles M., Eardley I., Selby P. J. and Banks R. E. (2003). Proteomic changes in renal cancer and co-ordinate demonstration of both the glycolytic and mitochondrial aspects of the Warburg effect. *Proteomics* 3, 1620-1632. 10.1002/pmic.200300464 12923786

[BIO042838C53] Wang F., Osawa T., Tsuchida R., Yuasa Y. and Shibuya M. (2011). Downregulation of receptor for activated C-kinase 1 (RACK1) suppresses tumor growth by inhibiting tumor cell proliferation and tumor-associated angiogenesis. *Cancer Sci.* 102, 2007-2013. 10.1111/j.1349-7006.2011.02065.x 21848913 PMC11159629

[BIO042838C54] Welch-Reardon K. M., Wu N. and Hughes C. C. W. (2015). A role for partial endothelial-mesenchymal transitions in angiogenesis? *Arterioscler. Thromb. Vasc. Biol.* 35, 303-308. 10.1161/ATVBAHA.114.303220 25425619 PMC4911209

[BIO042838C55] Wu M., Han L., Shi Y., Xu G., Wei J., You L., Chen Y., Zhu T., Li Q., Li S. et al. (2010). Development and characterization of a novel method for the analysis of gene expression patterns in lymphatic endothelial cells derived from primary breast tissues. *J. Cancer Res. Clin. Oncol.* 136, 863-872. 10.1007/s00432-009-0727-9 19936789 PMC11828302

[BIO042838C56] Wu Z. B., Cai L., Lin S. J., Leng Z. G., Guo Y. H., Yang W. L., Chu Y. W., Yang S. H. and Zhao W. G. (2016). Heat shock protein 47 promotes glioma angiogenesis. *Brain Pathol.* 26 31-42. 10.1111/bpa.12256 25758142 PMC8029092

[BIO042838C57] Xiong Y.-Q., Sun H.-C., Zhang W., Zhu X.-D., Zhuang P.-Y., Zhang J.-B., Wang L., Wu W.-Z., Qin L.-X. and Tang Z.-Y. (2009). Human hepatocellular carcinoma tumor-derived endothelial cells manifest increased angiogenesis capability and drug resistance compared with normal endothelial cells. *Clin. Cancer Res.* 15, 4838-4846. 10.1158/1078-0432.CCR-08-2780 19638466

[BIO042838C58] Ye D.-J., Kwon Y.-J., Shin S., Baek H.-S., Shin D.-W. and Chun Y.-J. (2017). Induction of integrin signaling by steroid sulfatase in human cervical cancer cells. *Biomol. Ther.* 25, 321-328. 10.4062/biomolther.2016.155 PMC5424643 27956712

[BIO042838C59] Yu Y., Wu J., Wang Y., Zhao T., Ma B., Liu Y., Fang W., Zhu W.-G. and Zhang H. (2012). Kindlin 2 forms a transcriptional complex with beta-catenin and TCF4 to enhance Wnt signalling. *EMBO Rep.* 13, 750-758. 10.1038/embor.2012.88 22699938 PMC3410388

[BIO042838C60] Zang M., Zhang Y., Zhang B., Hu L., Li J., Fan Z., Wang H., Su L., Zhu Z., Li C. et al. (2015). CEACAM6 promotes tumor angiogenesis and vasculogenic mimicry in gastric cancer via FAK signaling. *Biochim. Biophys. Acta* 1852, 1020-1028. 10.1016/j.bbadis.2015.02.005 25703140

[BIO042838C61] Zeng G. Q., Zhang P. F., Deng X., Yu F. L., Li C., Xu Y., Yi H., Li M. Y., Hu R., Zuo J. H. et al. (2012). Identification of candidate biomarkers for early detection of human lung squamous cell cancer by quantitative proteomics. *Mol. Cell. Proteomics* 11: M111.013946 10.1074/mcp.M111.013946 PMC3433890 22298307

[BIO042838C62] Zhang X., Wei L., Wang J., Qin Z., Wang J., Lu Y., Zheng X., Peng Q., Ye Q., Ai F. et al. (2017). Suppression colitis and colitis-associated colon cancer by anti-S100a9 antibody in mice. *Front. Immunol.* 8, 1774 10.3389/fimmu.2017.01774 29326691 PMC5733461

[BIO042838C63] Zhao X. and Guan J.-L. (2011). Focal adhesion kinase and its signaling pathways in cell migration and angiogenesis. *Adv. Drug Delivery. Rev.* 63, 610-615. 10.1016/j.addr.2010.11.001 PMC3132829 21118706

