# Supplementary material for: Quantitative proteomic profiling of tumor-associated vascular endothelial cells in colorectal cancer
Source: Biol Open. 2019 Apr 29;8(5):bio042838. doi: 10.1242/bio.042838 (PMC6550088; doi:10.1242/bio.042838)
Supplement: Supplementary information [file biolopen-8-042838-s1.pdf]

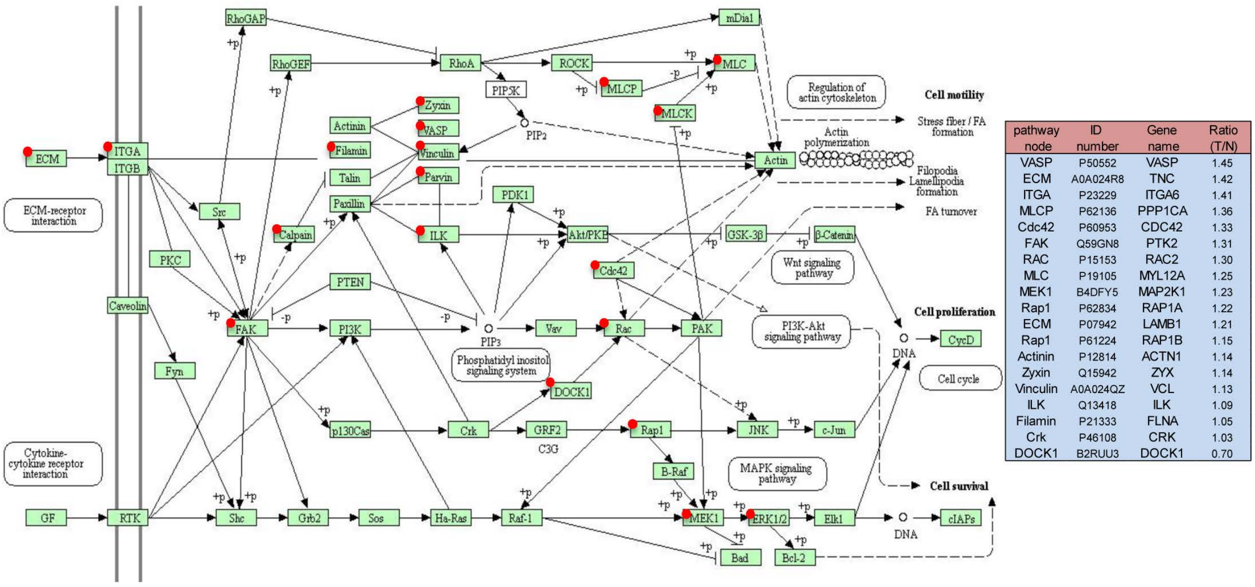

pathway node	ID number	Gene name	Ratio (T/N)
VASP	P50552	VASP	1.45
ECM	A0A024R8	TNC	1.42
ITGA	P23229	ITGA6	1.41
MLCP	P62136	PPP1CA	1.36
Cdc42	P60953	CDC42	1.33
FAK	Q59GN8	PTK2	1.31
RAC	P15153	RAC2	1.30
MLC	P19105	MYL12A	1.25
MEK1	B4DFY5	MAP2K1	1.23
Rap1	P62834	RAP1A	1.22
ECM	P07942	LAMB1	1.21
Rap1	P61224	RAP1B	1.15
Actinin	P12814	ACTN1	1.14
Zyxin	Q15942	ZYX	1.14
Vinculin	A0A024QZ	VCL	1.13
ILK	Q13418	ILK	1.09
Filamin	P21333	FLNA	1.05
Crk	P46108	CRK	1.03
DOCK1	B2RUU3	DOCK1	0.70

Figure S1: Focal adhesion pathway altered in a CRC. Green rectangle with red mark means the identified proteins. Green rectangle without red mark means species-specific enzymes. White rectangle means reference pathway. The solid line indicates molecular interaction. The dot line means indirect effect. The pathway node in the right panel corresponds to the red marked node in the left diagram. ID number is the Swiss-Prot accession number. Ratio (T/N) = Ratio of tumors to controls.

Biology Open • Supplementary information

Table S1: 2058 identified proteins between colorectal cancer and control in an 6-plex iTRAQ proteomics

Accession	Description	Coverage	Unique Peptides	Peptides	PSMs	AAs	MW [kDa]	calc. pI	CRC/ANC 1	CRC/ANC 2	CRC/ANC 3	average CRC/ANC	one sample t-test p value	FDR
P52597	Heterogeneous nuclear ribonucleoprotein F OS=Homo sapiens GN=HNRNPF PE=1 SV=3 - [HNRPF_HUMAN]	12.29	3	4	8	415	45.64286	5.57959	1.80005	1.92074	1.95987	1.8935542	0.0028848	0.0360252
Q9UBX5	Fibulin-5 OS=Homo sapiens GN=FBLN5 PE=1 SV=1 - [FBLN5_HUMAN]	23.88	10	10	16	448	50.14667	4.729004	1.84631	1.86427	1.73048	1.8136875	0.00264451	0.0357839
P13727	Bone marrow proteoglycan OS=Homo sapiens GN=PRG2 PE=1 SV=2 - [PRG2_HUMAN]	32.43	8	8	15	222	25.18926	6.756348	1.8487	1.81131	1.77466	1.8115533	0.000692846	0.0241782
Q6PKI6	YBX1 protein (Fragment) OS=Homo sapiens GN=YBX1 PE=2 SV=1 - [Q6PKI6_HUMAN]	19.17	2	3	5	266	29.35674	10.22803	1.74384	1.87525	1.74133	1.786806	0.00314474	0.0364553
P40199	Carcinoembryonic antigen-related cell adhesion molecule 6 OS=Homo sapiens GN=CEACAM6 PE=1 SV=3 - [CEAM6_HUMAN]	9.88	2	2	3	344	37.17169	5.820801	1.81045	1.67278	1.74709	1.7434418	0.0028515	0.0360252
J3KPD9	Nucleoside diphosphate kinase B OS=Homo sapiens GN=NME2 PE=1 SV=1 - [J3KPD9_HUMAN]	47.21	7	7	14	197	22.40773	9.539551	1.76728	1.60445	1.80617	1.7259647	0.00716588	0.0439255
A0PJ62	RPL14 protein (Fragment) OS=Homo sapiens GN=RPL14 PE=2 SV=1 - [A0PJ62_HUMAN]	28.23	3	3	6	124	14.60195	10.62354	1.7949	1.78122	1.57005	1.7153893	0.0101914	0.0485383
A0A024QZV0	HCG1811539, isoform CRA_b OS=Homo sapiens GN=hCG_1811539 PE=3 SV=1 - [A0A024QZV0_HUMAN]	38.27	12	12	19	324	36.15501	5.465332	1.65214	1.68004	1.69995	1.6773763	0.000418756	0.0220588
P16402	Histone H1.3 OS=Homo sapiens GN=HIST1H1D PE=1 SV=2 - [H13_HUMAN]	33.94	4	12	38	221	22.33632	11.01904	1.58874	1.78617	1.57235	1.6490858	0.0110193	0.0493307
A0A024RAL1	Chondroitin sulfate proteoglycan 2 (Versican), isoform CRA_c OS=Homo sapiens GN=CSPG2 PE=4 SV=1 - [A0A024RAL1_HUMAN]	5.98	8	14	19	2409	264.8861	4.538574	1.57643	1.66207	1.69287	1.6437899	0.00291471	0.0360252

Biology Open • Supplementary information

P06702	Protein S100-A9 OS=Homo sapiens GN=S100A9 PE=1 SV=1 - [S10A9_HUMAN]	70.18	7	7	30	114	13.23351	6.125488	1.54392	1.73564	1.60081	1.626792	0.00812617	0.0456419
A8KAJ3	cDNA FLJ77823, highly similar to Homo sapiens EGF-containing fibulin-like extracellular matrix protein 1, transcript variant 3, mRNA OS=Homo sapiens PE=2 SV=1 - [A8KAJ3_HUMAN]	25.96	11	11	21	493	54.63228	5.135254	1.53778	1.69272	1.60485	1.6117827	0.00533464	0.0401071
Q08ES8	Cell growth-inhibiting protein 34 OS=Homo sapiens PE=2 SV=1 - [Q08ES8_HUMAN]	16.95	3	3	6	177	20.08157	9.598145	1.5509	1.68204	1.54453	1.5924889	0.00567208	0.040913
B3KUH7	Coronin OS=Homo sapiens PE=2 SV=1 - [B3KUH7_HUMAN]	6.02	3	3	3	615	66.38416	6.188965	1.58557	1.5249	1.56251	1.5576592	0.00100395	0.0269712
P62280	40S ribosomal protein S11 OS=Homo sapiens GN=RPS11 PE=1 SV=3 - [RS11_HUMAN]	36.71	6	6	7	158	18.41899	10.30127	1.49299	1.63622	1.54266	1.5572911	0.00562872	0.0408901
Q53T40	Putative uncharacterized protein FHL2 (Fragment) OS=Homo sapiens GN=FHL2 PE=4 SV=1 - [Q53T40_HUMAN]	13.69	2	2	2	168	19.29395	8.147949	1.59256	1.60057	1.47049	1.5545427	0.00571149	0.0409606
D6R9B6	40S ribosomal protein S3a OS=Homo sapiens GN=RPS3A PE=1 SV=1 - [D6R9B6_HUMAN]	35.17	6	6	10	145	16.52563	9.246582	1.46716	1.62879	1.55413	1.5500277	0.00713285	0.0439255
P10412	Histone H1.4 OS=Homo sapiens GN=HIST1H1E PE=1 SV=2 - [H14_HUMAN]	45.66	5	14	43	219	21.85201	11.03369	1.50413	1.70891	1.41738	1.5434732	0.0243672	0.0682241
P62750	60S ribosomal protein L23a OS=Homo sapiens GN=RPL23A PE=1 SV=1 - [RL23A_HUMAN]	31.41	6	6	7	156	17.68414	10.44775	1.58548	1.61345	1.42275	1.5405606	0.011882	0.0495204
A0A024R9Q1	Thrombospondin 1, isoform CRA_a OS=Homo sapiens GN=THBS1 PE=4 SV=1 - [A0A024R9Q1_HUMAN]	10.43	11	11	14	1170	129.2696	4.944824	1.51822	1.56894	1.5287	1.5386207	0.000822568	0.0246406
H3BRU6	Poly(rC)-binding protein 2 (Fragment) OS=Homo sapiens GN=PCBP2 PE=1 SV=1 - [H3BRU6_HUMAN]	25.25	2	6	10	301	31.69025	8.440918	1.46312	1.62602	1.51515	1.5347614	0.00797253	0.0454327
P27824	Calnexin OS=Homo sapiens GN=CANX PE=1 SV=2 - [CALX_HUMAN]	15.37	5	7	13	592	67.52585	4.602051	1.55923	1.54775	1.48818	1.5317186	0.00171092	0.0307118

Biology Open • Supplementary information

G3V1A4	Cofilin 1 (Non-muscle), isoform CRA_a OS=Homo sapiens GN=CFL1 PE=1 SV=1 - [G3V1A4_HUMAN]	76.51	8	12	42	149	16.80082	8.353027	1.4897	1.59328	1.5069	1.5299591	0.00363716	0.0381621
G3V511	Latent-transforming growth factor beta-binding protein 2 OS=Homo sapiens GN=LTBP2 PE=1 SV=1 - [G3V511_HUMAN]	3.45	6	6	8	1769	189.1168	5.401855	1.5021	1.54041	1.52422	1.5222444	0.000451609	0.0224205
P07237	Protein disulfide-isomerase OS=Homo sapiens GN=P4HB PE=1 SV=3 - [PDIA1_HUMAN]	45.67	21	21	37	508	57.08068	4.868652	1.45226	1.5984	1.49964	1.5167658	0.0068679	0.0433728
P59665	Neutrophil defensin 1 OS=Homo sapiens GN=DEFA1 PE=1 SV=1 - [DEF1_HUMAN]	20.21	3	3	9	94	10.19418	6.990723	1.47944	1.436	1.63232	1.5159211	0.0130564	0.0513309
B2RA03	cDNA, FLJ94640, highly similar to Homo sapiens keratin 18 (KRT18), mRNA OS=Homo sapiens PE=2 SV=1 - [B2RA03_HUMAN]	24.88	10	11	13	430	48.0005	5.376465	1.50649	1.55476	1.47667	1.5126397	0.00196411	0.0321184
B3KTM6	Ribosomal protein L5, isoform CRA_b OS=Homo sapiens GN=RPL5 PE=2 SV=1 - [B3KTM6_HUMAN]	33.6	5	8	11	247	28.02619	9.129395	1.49935	1.64935	1.38276	1.5104869	0.0220916	0.0645367
P18077	60S ribosomal protein L35a OS=Homo sapiens GN=RPL35A PE=1 SV=2 - [RL35A_HUMAN]	28.18	4	4	6	110	12.52977	11.06299	1.46865	1.5505	1.5091	1.5094154	0.00214433	0.0336044
M0R3F1	Heterogeneous nuclear ribonucleoprotein U-like protein 1 (Fragment) OS=Homo sapiens GN=HNRNPUL1 PE=1 SV=1 - [M0R3F1_HUMAN]	5.62	3	3	3	641	71.67696	8.98291	1.43981	1.51395	1.57199	1.5085824	0.00560924	0.0408901
P05787	Keratin, type II cytoskeletal 8 OS=Homo sapiens GN=KRT8 PE=1 SV=7 - [K2C8_HUMAN]	42.86	19	26	53	483	53.67114	5.592285	1.54797	1.54664	1.418	1.504202	0.00722882	0.0439255
K7ENT6	Tropomyosin alpha-4 chain (Fragment) OS=Homo sapiens GN=TPM4 PE=1 SV=1 - [K7ENT6_HUMAN]	64.25	2	19	63	179	20.60838	4.614746	1.72265	1.461	1.32402	1.5025561	0.0501056	0.1023582
P05109	Protein S100-A8 OS=Homo sapiens GN=S100A8 PE=1 SV=1 - [S10A8_HUMAN]	68.82	9	9	32	93	10.82765	7.034668	1.46005	1.51546	1.49028	1.4885971	0.00107317	0.0276207

Biology Open • Supplementary information

P55145	Mesencephalic astrocyte-derived neurotrophic factor OS=Homo sapiens GN=MANF PE=1 SV=3 - [MANF_HUMAN]	21.43	3	3	4	182	20.68681	8.689941	1.46298	1.48293	1.51914	1.488351	0.00113119	0.0276207
Q96AG4	Leucine-rich repeat-containing protein 59 OS=Homo sapiens GN=LRRC59 PE=1 SV=1 - [LRC59_HUMAN]	25.08	6	6	8	307	34.90888	9.568848	1.46894	1.57368	1.39676	1.4797938	0.0112661	0.0493721
O15143	Actin-related protein 2/3 complex subunit 1B OS=Homo sapiens GN=ARPC1B PE=1 SV=3 - [ARC1B_HUMAN]	9.41	4	4	5	372	40.92342	8.353027	1.39492	1.59933	1.42675	1.4736662	0.0175015	0.0579673
Q7Z7M4	Superoxide dismutase (Fragment) OS=Homo sapiens GN=SOD2 PE=2 SV=1 - [Q7Z7M4_HUMAN]	31.92	8	8	20	213	23.65802	7.312988	1.4621	1.52989	1.41175	1.4679128	0.00530828	0.0401071
O75822	Eukaryotic translation initiation factor 3 subunit J OS=Homo sapiens GN=EIF3J PE=1 SV=2 - [EIF3J_HUMAN]	8.14	2	2	2	258	29.04454	4.830566	1.38731	1.46255	1.54311	1.4643235	0.00925534	0.0469705
P40261	Nicotinamide N-methyltransferase OS=Homo sapiens GN=NNMT PE=1 SV=1 - [NNMT_HUMAN]	17.05	4	4	5	264	29.55506	5.744629	1.36378	1.62541	1.38786	1.4590165	0.0315235	0.0784034
E4W6B6	RPL27/NME2 fusion protein (Fragment) OS=Homo sapiens GN=RPL27 PE=2 SV=1 - [E4W6B6_HUMAN]	19.84	3	3	4	126	14.24795	10.4624	1.50573	1.46088	1.39814	1.4549173	0.00467107	0.0398629
P50552	Vasodilator-stimulated phosphoprotein OS=Homo sapiens GN=VASP PE=1 SV=3 - [VASP_HUMAN]	12.11	4	4	4	380	39.80509	8.938965	1.42434	1.53852	1.39324	1.4520338	0.00941239	0.0472949
P61619	Protein transport protein Sec61 subunit alpha isoform 1 OS=Homo sapiens GN=SEC61A1 PE=1 SV=2 - [S61A1_HUMAN]	3.78	2	2	3	476	52.23052	8.060059	1.49856	1.55114	1.28854	1.4460791	0.0308519	0.0773766
P02792	Ferritin light chain OS=Homo sapiens GN=FTL PE=1 SV=2 - [FRIL_HUMAN]	32.57	5	5	7	175	20.0071	5.782715	1.52189	1.43501	1.38036	1.4457547	0.00843711	0.0456887
Q9NS69	Mitochondrial import receptor subunit TOM22 homolog OS=Homo sapiens GN=TOMM22 PE=1 SV=3 - [TOM22_HUMAN]	25.35	2	2	2	142	15.51179	4.335449	1.43332	1.48568	1.40784	1.4422785	0.00267284	0.0357839
B4DM63	Actin-related protein 2/3 complex subunit 3 OS=Homo sapiens PE=2 SV=1 - [B4DM63_HUMAN]	22.16	4	4	6	167	19.04891	8.880371	1.40081	1.31903	1.59565	1.4384968	0.0332723	0.0806036

Biology Open • Supplementary information

G3V2V6	V-type proton ATPase subunit D OS=Homo sapiens GN=ATP6V1D PE=1 SV=1 - [G3V2V6_HUMAN]	22.97	2	2	2	148	17.41163	9.524902	1.4914	1.3886	1.43397	1.437989	0.00458027	0.0398629
F5H3P3	Rho GDP-dissociation inhibitor 2 (Fragment) OS=Homo sapiens GN=ARHGDIB PE=1 SV=6 - [F5H3P3_HUMAN]	32.91	5	5	8	158	17.82615	5.19873	1.45921	1.53199	1.30886	1.4333543	0.0222184	0.0647174
B8ZZU8	Transcription elongation factor B (SIII), polypeptide 2 (18kDa, elongin B), isoform CRA_b OS=Homo sapiens GN=TCEB2 PE=1 SV=1 - [B8ZZU8_HUMAN]	30.09	2	2	4	113	12.51926	4.970215	1.37727	1.48732	1.43019	1.4315904	0.00537667	0.0401071
B4DV64	cDNA FLJ61420, highly similar to Homo sapiens olfactomedin 4 (OLFM4), mRNA OS=Homo sapiens PE=2 SV=1 - [B4DV64_HUMAN]	6.81	2	3	7	367	41.939	5.592285	1.40018	1.5212	1.36737	1.4295833	0.011649	0.0494101
P04080	Cystatin-B OS=Homo sapiens GN=CSTB PE=1 SV=2 - [CYTB_HUMAN]	18.37	2	2	2	98	11.13259	7.562012	1.40395	1.57631	1.29943	1.4265666	0.0339971	0.0813863
A0A024R884	Tenascin C (Hexabrachion), isoform CRA_a OS=Homo sapiens GN=TNC PE=4 SV=1 - [A0A024R884_HUMAN]	22.9	39	39	67	2201	240.7553	4.919434	1.4227	1.43696	1.3901	1.4165865	0.00110659	0.0276207
P46779	60S ribosomal protein L28 OS=Homo sapiens GN=RPL28 PE=1 SV=3 - [RL28_HUMAN]	32.12	5	5	9	137	15.73767	12.01514	1.33875	1.481	1.42737	1.4157054	0.00980827	0.0478721
P80723	Brain acid soluble protein 1 OS=Homo sapiens GN=BASP1 PE=1 SV=2 - [BASP1_HUMAN]	41.41	6	6	12	227	22.68001	4.627441	1.43544	1.41844	1.38709	1.4136564	0.00117024	0.0276207
P50454	Serpin H1 OS=Homo sapiens GN=SERPINH1 PE=1 SV=2 - [SERPH_HUMAN]	43.3	16	16	40	418	46.41118	8.689941	1.37136	1.44501	1.41759	1.4113187	0.0027188	0.0357839
A8K486	Peptidyl-prolyl cis-trans isomerase OS=Homo sapiens PE=2 SV=1 - [A8K486_HUMAN]	47.27	9	10	39	165	18.00183	6.902832	1.41017	1.40762	1.41295	1.4102468	1.41E-05	0.0119321
B4DNW7	Adenylyl cyclase-associated protein OS=Homo sapiens PE=2 SV=1 - [B4DNW7_HUMAN]	32.33	10	11	31	433	47.36054	8.221191	1.39302	1.40617	1.42933	1.4095095	0.000671152	0.0241782
P23229	Integrin alpha-6 OS=Homo sapiens GN=ITGA6 PE=1 SV=5 - [ITA6_HUMAN]	5.66	5	5	6	1130	126.5262	6.609863	1.35323	1.39748	1.46708	1.4059327	0.00659722	0.043197

Biology Open • Supplementary information

P16403	Histone H1.2 OS=Homo sapiens GN=HIST1H1C PE=1 SV=2 - [H12_HUMAN]	42.72	3	13	41	213	21.35175	10.93115	1.44985	1.48319	1.28292	1.4053187	0.0225745	0.0652002
P62633	Cellular nucleic acid-binding protein OS=Homo sapiens GN=CNBP PE=1 SV=1 - [CNBP_HUMAN]	16.38	2	2	2	177	19.44956	7.708496	1.31173	1.33506	1.56362	1.4034691	0.0374532	0.0866569
P62269	40S ribosomal protein S18 OS=Homo sapiens GN=RPS18 PE=1 SV=3 - [RS18_HUMAN]	34.21	8	8	12	152	17.70786	10.98975	1.4097	1.43565	1.36442	1.4032558	0.00265327	0.0357839
A0A024R5M3	Cortactin, isoform CRA_c OS=Homo sapiens GN=CTTN PE=4 SV=1 - [A0A024R5M3_HUMAN]	19.88	10	10	13	513	57.43154	5.325684	1.37033	1.43583	1.39312	1.399761	0.00229806	0.0342495
C9JXB8	60S ribosomal protein L24 OS=Homo sapiens GN=RPL24 PE=1 SV=1 - [C9JXB8_HUMAN]	38.02	5	5	7	121	14.35982	11.31201	1.38496	1.44412	1.36869	1.3992569	0.00327868	0.0366282
B2R829	cDNA, FLJ93711, highly similar to Homo sapiens myeloid cell nuclear differentiation antigen (MNDA), mRNA OS=Homo sapiens PE=2 SV=1 - [B2R829_HUMAN]	11.3	4	4	9	407	45.82124	9.759277	1.25206	1.45817	1.46803	1.3927503	0.0306633	0.0773154
P12724	Eosinophil cationic protein OS=Homo sapiens GN=RNASE3 PE=1 SV=2 - [ECP_HUMAN]	29.38	5	5	11	160	18.37335	10.02295	1.36603	1.35111	1.45505	1.3907296	0.00682653	0.0433606
C9JPM4	ADP-ribosylation factor 4 (Fragment) OS=Homo sapiens GN=ARF4 PE=1 SV=1 - [C9JPM4_HUMAN]	33.86	3	4	14	127	14.54377	8.118652	1.50239	1.43578	1.23276	1.3903097	0.0405532	0.0906037
K7EQA1	Programmed cell death protein 5 OS=Homo sapiens GN=PDCD5 PE=1 SV=1 - [K7EQA1_HUMAN]	27.59	2	2	2	87	9.998246	8.997559	1.29805	1.46112	1.40914	1.3894359	0.0149108	0.0540327
Q5T8U5	Surfeit 4 OS=Homo sapiens GN=SURF4 PE=1 SV=1 - [Q5T8U5_HUMAN]	15.05	2	2	3	186	21.1137	6.521973	1.37203	1.28889	1.50712	1.389349	0.0256537	0.0705425
P39019	40S ribosomal protein S19 OS=Homo sapiens GN=RPS19 PE=1 SV=2 - [RS19_HUMAN]	33.1	6	6	17	145	16.05054	10.31592	1.37388	1.44602	1.34815	1.3893483	0.00561338	0.0408901
P05387	60S acidic ribosomal protein P2 OS=Homo sapiens GN=RPLP2 PE=1 SV=1 - [RLA2_HUMAN]	41.74	5	5	11	115	11.65785	4.538574	1.21073	1.5908	1.36484	1.3887912	0.0719893	0.1291382
B8ZZQ6	Prothymosin alpha OS=Homo sapiens GN=PTMA PE=1 SV=1	13.08	2	2	7	107	11.75182	3.814941	1.40298	1.40866	1.35096	1.3875332	0.00223652	0.033949

Biology Open • Supplementary information

	- [B8ZZQ6_HUMAN]													
P14780	Matrix metalloproteinase-9 OS=Homo sapiens GN=MMP9 PE=1 SV=3 - [MMP9_HUMAN]	6.36	4	4	4	707	78.40836	6.062012	1.35245	1.41556	1.39154	1.3865182	0.00225613	0.0339779
P21980	Protein-glutamine gamma-glutamyltransferase 2 OS=Homo sapiens GN=TGM2 PE=1 SV=2 - [TGM2_HUMAN]	33.33	18	18	35	687	77.27967	5.224121	1.3562	1.39254	1.40908	1.3859422	0.00163351	0.0301668
K7ERG4	Small nuclear ribonucleoprotein Sm D2 OS=Homo sapiens GN=SNRPD2 PE=1 SV=1 - [K7ERG4_HUMAN]	37.18	2	2	2	78	8.771419	8.689941	1.50823	1.33792	1.30985	1.385333	0.0249087	0.0690615
F8VS81	Twinfilin-1 (Fragment) OS=Homo sapiens GN=TWF1 PE=1 SV=1 - [F8VS81_HUMAN]	9.28	2	2	4	291	33.387	6.455566	1.43406	1.32901	1.39137	1.3848117	0.00622496	0.0423147
B2MUD5	Neutrophil elastase (Fragment) OS=Homo sapiens GN=ELA2 PE=3 SV=1 - [B2MUD5_HUMAN]	9.38	2	2	3	192	20.64865	8.89502	1.39483	1.38541	1.37398	1.3847393	0.000245451	0.0212234
Q9BRF8	Serine/threonine-protein phosphatase CPPED1 OS=Homo sapiens GN=CPPED1 PE=1 SV=3 - [CPPED_HUMAN]	8.28	2	2	2	314	35.52588	6.20166	1.36686	1.34828	1.43895	1.3846959	0.00512689	0.0401071
G3V1D1	Ferritin OS=Homo sapiens GN=FTH1 PE=1 SV=1 - [G3V1D1_HUMAN]	30.09	3	3	7	113	12.86831	5.300293	1.11367	1.46567	1.57237	1.3839034	0.109346	0.1722459
Q8WU39	Marginal zone B- and B1-cell-specific protein OS=Homo sapiens GN=MZB1 PE=1 SV=1 - [MZB1_HUMAN]	38.62	5	5	8	189	20.68123	5.566895	1.38653	1.36725	1.39728	1.3836873	0.000523649	0.0241782
M0R210	40S ribosomal protein S16 OS=Homo sapiens GN=RPS16 PE=1 SV=1 - [M0R210_HUMAN]	47.29	6	6	12	129	14.40996	10.22803	1.35175	1.45952	1.33161	1.3809592	0.0106904	0.0490917
P31151	Protein S100-A7 OS=Homo sapiens GN=S100A7 PE=1 SV=4 - [S10A7_HUMAN]	22.77	2	2	2	101	11.46356	6.770996	1.33682	1.42817	1.37506	1.380015	0.00482255	0.0401071
B5MCP9	40S ribosomal protein S7 OS=Homo sapiens GN=RPS7 PE=1 SV=1 - [B5MCP9_HUMAN]	10.7	2	2	4	187	21.29877	10.27197	1.41107	1.43725	1.28957	1.3792974	0.0140842	0.0527963
P13639	Elongation factor 2 OS=Homo sapiens GN=EEF2 PE=1 SV=4 - [EF2_HUMAN]	27.16	21	22	36	858	95.27695	6.82959	1.33651	1.44066	1.35446	1.3772107	0.00718396	0.0439255

Biology Open • Supplementary information

P80188	Neutrophil gelatinase-associated lipocalin OS=Homo sapiens GN=LCN2 PE=1 SV=2 - [NGAL_HUMAN]	11.11	2	2	3	198	22.57372	8.909668	1.36694	1.43703	1.31776	1.3739109	0.0084576	0.0456887
Q15293	Reticulocalbin-1 OS=Homo sapiens GN=RCN1 PE=1 SV=1 - [RCN1_HUMAN]	11.78	3	3	6	331	38.86617	4.995605	1.33281	1.46121	1.32646	1.3734933	0.0135346	0.0520894
P52907	F-actin-capping protein subunit alpha-1 OS=Homo sapiens GN=CAPZA1 PE=1 SV=3 - [CAZA1_HUMAN]	39.51	6	7	12	286	32.90233	5.693848	1.34222	1.45028	1.32795	1.3734839	0.010524	0.0489337
B4E380	Histone H3 OS=Homo sapiens PE=2 SV=1 - [B4E380_HUMAN]	32.74	3	5	12	113	12.91035	11.92725	1.28094	1.43997	1.39612	1.3723417	0.0158367	0.0551181
P06733	Alpha-enolase OS=Homo sapiens GN=ENO1 PE=1 SV=2 - [ENOA_HUMAN]	50.92	15	19	49	434	47.13932	7.38623	1.35267	1.3686	1.39493	1.372066	0.00109488	0.0276207
Q15084	Protein disulfide-isomerase A6 OS=Homo sapiens GN=PDIA6 PE=1 SV=1 - [PDIA6_HUMAN]	27.27	10	10	23	440	48.09126	5.084473	1.35224	1.36592	1.39743	1.3718644	0.00129203	0.0281615
P52209	6-phosphogluconate dehydrogenase, decarboxylating OS=Homo sapiens GN=PGD PE=1 SV=3 - [6PGD_HUMAN]	28.99	13	13	22	483	53.10596	7.225098	1.37092	1.35341	1.39084	1.3717208	0.000845213	0.0248219
V9HWC6	Peptidyl-prolyl cis-trans isomerase OS=Homo sapiens GN=HEL-S-39 PE=2 SV=1 - [V9HWC6_HUMAN]	43.75	11	12	20	208	22.72801	9.319824	1.36992	1.37796	1.3654	1.371091	9.80E-05	0.0195135
I3L3C4	Ribosomal L1 domain-containing protein 1 (Fragment) OS=Homo sapiens GN=RSL1D1 PE=1 SV=1 - [I3L3C4_HUMAN]	10.13	2	2	2	158	18.52317	10.06689	1.32765	1.32621	1.45253	1.3687967	0.0126456	0.0508886
B7ZAL5	cDNA, FLJ79229, highly similar to Lactotransferrin (EC 3.4.21.-) OS=Homo sapiens PE=2 SV=1 - [B7ZAL5_HUMAN]	42.49	25	25	39	666	73.12321	7.781738	1.32557	1.37081	1.39601	1.3641303	0.00318755	0.0364914
A0A024R3J7	HCG2032701, isoform CRA_a OS=Homo sapiens GN=hCG_2032701 PE=4 SV=1 - [A0A024R3J7_HUMAN]	5.55	4	5	7	703	80.37573	7.811035	1.36934	1.43172	1.28518	1.3620805	0.0134725	0.0520005
Q06323	Proteasome activator complex subunit 1 OS=Homo sapiens GN=PSME1 PE=1 SV=1 - [PSME1_HUMAN]	47.79	13	13	17	249	28.70502	6.023926	1.33119	1.37237	1.3825	1.3620172	0.00187285	0.0312295

Biology Open • Supplementary information

P62136	Serine/threonine-protein phosphatase PP1-alpha catalytic subunit OS=Homo sapiens GN=PPP1CA PE=1 SV=1 - [PP1A_HUMAN]	35.45	4	9	17	330	37.48781	6.328613	1.32495	1.35944	1.4008	1.361731	0.00365355	0.0381706
P36952	Serpin B5 OS=Homo sapiens GN=SERPINB5 PE=1 SV=2 - [SPB5_HUMAN]	10.4	3	3	3	375	42.0734	6.049316	1.35072	1.40443	1.33	1.3617173	0.0037387	0.0385838
P68366	Tubulin alpha-4A chain OS=Homo sapiens GN=TUBA4A PE=1 SV=1 - [TBA4A_HUMAN]	51.79	5	19	81	448	49.89237	5.059082	1.36056	1.35626	1.36605	1.3609574	6.16E-05	0.0173763
O60613	15 kDa selenoprotein OS=Homo sapiens GN=SEP15 PE=1 SV=3 - [SEP15_HUMAN]	15.43	2	2	2	162	17.77994	5.033691	1.38335	1.30556	1.38739	1.3587696	0.00546354	0.0404685
P68104	Elongation factor 1-alpha 1 OS=Homo sapiens GN=EEF1A1 PE=1 SV=1 - [EF1A1_HUMAN]	43.29	7	15	50	462	50.10911	9.012207	1.35081	1.39631	1.32656	1.3578908	0.00324727	0.036522
P62820	Ras-related protein Rab-1A OS=Homo sapiens GN=RAB1A PE=1 SV=3 - [RAB1A_HUMAN]	54.15	4	10	14	205	22.66338	6.214355	1.2682	1.31959	1.48311	1.3569688	0.0314012	0.0782718
Q9ULC5	Long-chain-fatty-acid--CoA ligase 5 OS=Homo sapiens GN=ACSL5 PE=1 SV=1 - [ACSL5_HUMAN]	5.86	2	3	3	683	75.94217	6.91748	1.69476	1.10558	1.2656	1.355316	0.180805	0.2493346
Q53G71	Calreticulin variant (Fragment) OS=Homo sapiens PE=2 SV=1 - [Q53G71_HUMAN]	33.99	15	15	33	406	46.89009	4.449707	1.30306	1.40044	1.35905	1.3541852	0.0062864	0.0423147
P08253	72 kDa type IV collagenase OS=Homo sapiens GN=MMP2 PE=1 SV=2 - [MMP2_HUMAN]	9.24	5	5	5	660	73.83472	5.465332	1.25001	1.38271	1.42944	1.3540527	0.0222717	0.0647174
B3KQT9	Protein disulfide-isomerase OS=Homo sapiens PE=2 SV=1 - [B3KQT9_HUMAN]	45.21	15	22	52	480	54.06848	7.210449	1.33566	1.39013	1.33291	1.352897	0.00277592	0.0360019
A8K335	cDNA FLJ76254, highly similar to Homo sapiens gamma-glutamyl hydrolase (GGH), mRNA OS=Homo sapiens PE=2 SV=1 - [A8K335_HUMAN]	9.75	3	3	3	318	36.02427	7.415527	1.39712	1.37562	1.28463	1.3524549	0.00943319	0.0472949
Q9BVJ7	Dual specificity protein phosphatase 23 OS=Homo sapiens GN=DUSP23 PE=1 SV=1 - [DUS23_HUMAN]	14	2	2	2	150	16.57761	8.206543	1.26605	1.3191	1.46859	1.3512507	0.028533	0.0748135

F8VV32	Lysozyme OS=Homo sapiens GN=LYZ PE=1 SV=1 - [F8VV32_HUMAN]	45.19	5	5	6	104	11.48071	9.070801	1.33003	1.43084	1.29085	1.3505752	0.0138537	0.0525187
B4DRR0	cDNA FLJ53910, highly similar to Keratin, type II cytoskeletal 6A OS=Homo sapiens PE=2 SV=1 - [B4DRR0_HUMAN]	44.67	3	24	34	535	57.80324	8.001465	1.32433	1.40834	1.30686	1.3465101	0.00807376	0.0456255
Q9Y5P6	Mannose-1-phosphate guanyltransferase beta OS=Homo sapiens GN=GMPPB PE=1 SV=2 - [GMPPB_HUMAN]	11.67	4	4	4	360	39.80859	6.609863	1.34147	1.37741	1.3095	1.342793	0.00325839	0.036522
P52790	Hexokinase-3 OS=Homo sapiens GN=HK3 PE=1 SV=2 - [HXK3_HUMAN]	3.03	3	3	3	923	98.96213	5.401855	1.23459	1.40407	1.38776	1.3421391	0.0240004	0.0675863
A8K9J7	Histone H2B OS=Homo sapiens PE=2 SV=1 - [A8K9J7_HUMAN]	57.94	2	9	38	126	13.98761	10.31592	1.30055	1.46016	1.26358	1.3414335	0.0298188	0.0765061
Q2TNB3	Cell migration-inducing protein 22 OS=Homo sapiens PE=2 SV=1 - [Q2TNB3_HUMAN]	22.59	6	6	6	239	27.33132	5.57959	1.32618	1.39328	1.30237	1.3406073	0.00632437	0.0423147
F8VQX6	Methyltransferase-like protein 7A (Fragment) OS=Homo sapiens GN=METTL7A PE=1 SV=1 - [F8VQX6_HUMAN]	15	2	2	3	180	20.64863	8.104004	1.3498	1.33428	1.33758	1.3405546	0.000191992	0.0212234
A0A0C4DGS1	Dolichyl-diphosphooligosaccharide--protein glycosyltransferase 48 kDa subunit OS=Homo sapiens GN=DDOST PE=1 SV=1 - [A0A0C4DGS1_HUMAN]	10.48	5	5	8	439	48.76895	5.693848	1.27821	1.35631	1.38317	1.3392311	0.00850083	0.0456887
P63244	Guanine nucleotide-binding protein subunit beta-2-like 1 OS=Homo sapiens GN=GNB2L1 PE=1 SV=3 - [GBLP_HUMAN]	30.6	9	9	15	317	35.05456	7.693848	1.3375	1.2803	1.3992	1.3390001	0.0101019	0.0483662
P00558	Phosphoglycerate kinase 1 OS=Homo sapiens GN=PGK1 PE=1 SV=3 - [PGK1_HUMAN]	50.84	18	18	38	417	44.58613	8.104004	1.32882	1.37854	1.30507	1.3374791	0.00408899	0.0390309
B4DTA2	cDNA FLJ60148, highly similar to Homo sapiens heterogeneous nuclear ribonucleoprotein D-like (HNRPDL), transcript variant 2, mRNA OS=Homo sapiens PE=2 SV=1 - [B4DTA2_HUMAN]	25.09	3	5	12	271	30.19565	8.719238	1.32322	1.39323	1.29441	1.3369541	0.00749719	0.0446031

Biology Open • Supplementary information

P67936	Tropomyosin alpha-4 chain OS=Homo sapiens GN=TPM4 PE=1 SV=3 - [TPM4_HUMAN]	69.35	6	24	75	248	28.50449	4.690918	1.24378	1.36617	1.39809	1.3360156	0.0190315	0.0605312
P16401	Histone H1.5 OS=Homo sapiens GN=HIST1H1B PE=1 SV=3 - [H15_HUMAN]	28.76	4	7	17	226	22.56647	10.9165	1.3404	1.3569	1.31044	1.3359098	0.00163465	0.0301668
P62851	40S ribosomal protein S25 OS=Homo sapiens GN=RPS25 PE=1 SV=1 - [RS25_HUMAN]	24	4	4	10	125	13.7337	10.11084	1.23038	1.42365	1.35247	1.3355009	0.0271492	0.0730525
H7C2W9	60S ribosomal protein L31 (Fragment) OS=Homo sapiens GN=RPL31 PE=1 SV=1 - [H7C2W9_HUMAN]	35.19	4	4	6	108	12.80306	11.00439	1.3206	1.38505	1.29847	1.3347064	0.0059664	0.0419659
F2Z388	60S ribosomal protein L35 OS=Homo sapiens GN=RPL35 PE=1 SV=1 - [F2Z388_HUMAN]	14.58	2	2	3	96	10.63799	10.28662	1.28359	1.31754	1.39866	1.333264	0.0103299	0.0487686
Q9Y3F4	Serine-threonine kinase receptor-associated protein OS=Homo sapiens GN=STRAP PE=1 SV=1 - [STRAP_HUMAN]	10.57	3	3	3	350	38.41398	5.122559	1.34841	1.39127	1.25807	1.3325848	0.0136468	0.0523153
F8W6I7	Heterogeneous nuclear ribonucleoprotein A1 OS=Homo sapiens GN=HNRNPA1 PE=1 SV=2 - [F8W6I7_HUMAN]	39.09	8	13	28	307	33.13485	9.129395	1.37641	1.40764	1.2129	1.3323176	0.0314661	0.0783182
C9JJ34	Ran-specific GTPase-activating protein (Fragment) OS=Homo sapiens GN=RANBP1 PE=1 SV=1 - [C9JJ34_HUMAN]	13.5	2	2	2	163	18.75022	5.211426	1.3116	1.40625	1.27803	1.3319575	0.0131097	0.0513658
P00338	L-lactate dehydrogenase A chain OS=Homo sapiens GN=LDHA PE=1 SV=2 - [LDHA_HUMAN]	40.96	12	14	32	332	36.66536	8.265137	1.3277	1.30499	1.35584	1.3295094	0.00198675	0.0321884
Q5CAQ5	Tumor rejection antigen (Gp96) 1 OS=Homo sapiens GN=TRA1 PE=2 SV=1 - [Q5CAQ5_HUMAN]	35.16	26	28	56	802	92.28231	4.855957	1.34788	1.33823	1.2992	1.3284348	0.00204705	0.0323797
Q16543	Hsp90 co-chaperone Cdc37 OS=Homo sapiens GN=CDC37 PE=1 SV=1 - [CDC37_HUMAN]	10.85	3	3	5	378	44.44	5.249512	1.31206	1.34443	1.32273	1.3264061	0.000850618	0.0248219
Q5TD97	Four and a half LIM domains protein 5 OS=Homo sapiens GN=FHL5 PE=1 SV=1 - [FHL5_HUMAN]	6.69	2	2	2	284	32.69671	7.518066	1.33427	1.33539	1.3093	1.3263239	0.000680493	0.0241782

P60953	Cell division control protein 42 homolog OS=Homo sapiens GN=CDC42 PE=1 SV=2 - [CDC42_HUMAN]	35.6	2	6	18	191	21.24502	6.55127	1.34578	1.4037	1.22587	1.3251139	0.0249742	0.0691798
P61956	Small ubiquitin-related modifier 2 OS=Homo sapiens GN=SUMO2 PE=1 SV=3 - [SUMO2_HUMAN]	18.95	2	2	3	95	10.86436	5.503418	1.41238	1.37955	1.1833	1.3250759	0.0451497	0.0965456
P31146	Coronin-1A OS=Homo sapiens GN=CORO1A PE=1 SV=4 - [COR1A_HUMAN]	14.53	7	7	10	461	50.9938	6.683105	1.3042	1.33202	1.33778	1.3246668	0.00101842	0.0271445
B4DVD8	Caspase-1 OS=Homo sapiens GN=CASP1 PE=1 SV=1 - [B4DVD8_HUMAN]	16.28	4	4	4	301	32.98836	5.566895	1.25574	1.27776	1.43261	1.3220374	0.0285872	0.0748616
O15212	Prefoldin subunit 6 OS=Homo sapiens GN=PFDN6 PE=1 SV=1 - [PFD6_HUMAN]	15.5	2	2	3	129	14.5738	8.880371	1.37926	1.26429	1.31995	1.3211654	0.0105137	0.0489337
P20700	Lamin-B1 OS=Homo sapiens GN=LMNB1 PE=1 SV=2 - [LMNB1_HUMAN]	32.08	15	17	24	586	66.36763	5.160645	1.34956	1.33972	1.26972	1.3196689	0.00612577	0.0421458
J3KTJ8	60S ribosomal protein L26 (Fragment) OS=Homo sapiens GN=RPL26 PE=4 SV=6 - [J3KTJ8_HUMAN]	37.5	5	5	7	96	11.49122	10.90186	1.34901	1.29097	1.31888	1.3196233	0.00273797	0.0357839
Q2TU34	Fructose-1,6-bisphosphatase 1 OS=Homo sapiens GN=FBP1 PE=2 SV=1 - [Q2TU34_HUMAN]	17.46	5	5	8	338	36.79079	6.990723	1.32662	1.44494	1.18632	1.3192942	0.0506718	0.1028322
Q14847	LIM and SH3 domain protein 1 OS=Homo sapiens GN=LASP1 PE=1 SV=2 - [LASP1_HUMAN]	30.27	9	9	22	261	29.69822	7.049316	1.30119	1.34476	1.30573	1.3172267	0.00189481	0.0313595
H7C3T4	Peroxiredoxin-4 (Fragment) OS=Homo sapiens GN=PRDX4 PE=1 SV=1 - [H7C3T4_HUMAN]	37.27	4	6	15	161	18.31326	6.188965	1.26955	1.26619	1.40891	1.3148828	0.0215808	0.0637577
D6RDM7	Ubiquitin-conjugating enzyme E2 K (Fragment) OS=Homo sapiens GN=UBE2K PE=1 SV=1 - [D6RDM7_HUMAN]	13.82	2	2	2	123	14.02619	7.181152	1.35498	1.27115	1.31707	1.3144016	0.00589005	0.0416238
P13693	Translationally-controlled tumor protein OS=Homo sapiens GN=TPT1 PE=1 SV=1 - [TCTP_HUMAN]	28.49	5	5	12	172	19.58258	4.932129	1.30257	1.30512	1.33041	1.3126985	0.000806614	0.0243785
Q14019	Coactosin-like protein OS=Homo sapiens GN=COTL1 PE=1 SV=3 - [COTL1_HUMAN]	31.69	4	4	6	142	15.93504	5.668457	1.30111	1.38399	1.25222	1.3124421	0.0148163	0.0539862

Biology Open • Supplementary information

P13667	Protein disulfide-isomerase A4 OS=Homo sapiens GN=PDIA4 PE=1 SV=2 - [PDIA4_HUMAN]	43.88	24	25	42	645	72.88698	5.071777	1.31221	1.32039	1.30334	1.3119825	0.000249001	0.0212234
B4E3T6	cDNA FLJ56405, highly similar to Epidermal growth factor receptor kinase substrate 8 OS=Homo sapiens PE=2 SV=1 - [B4E3T6_HUMAN]	6.08	2	2	2	691	77.03985	7.85498	1.20962	1.21037	1.51379	1.3112597	0.0915437	0.1526499
B3KQF5	cDNA FLJ90381 fis, clone NT2RP2005035, highly similar to Calumenin OS=Homo sapiens PE=2 SV=1 - [B3KQF5_HUMAN]	33.02	10	10	15	315	36.99556	4.665527	1.34625	1.28616	1.3013	1.3112374	0.00334455	0.0369318
A0A0C4DFV9	Protein SET OS=Homo sapiens GN=SET PE=1 SV=1 - [A0A0C4DFV9_HUMAN]	17.67	4	4	9	266	31.10526	4.233887	1.29873	1.41609	1.21868	1.311166	0.0323051	0.0797133
P15880	40S ribosomal protein S2 OS=Homo sapiens GN=RPS2 PE=1 SV=2 - [RS2_HUMAN]	40.96	10	10	19	293	31.3046	10.24268	1.28371	1.34514	1.30436	1.3110665	0.00334941	0.0369318
Q9BSQ6	RPL13A protein (Fragment) OS=Homo sapiens GN=RPL13A PE=2 SV=2 - [Q9BSQ6_HUMAN]	25.37	6	6	10	201	23.3603	10.93115	1.30655	1.32003	1.30426	1.3102824	0.000251157	0.0212234
B7Z6B3	Receptor expression-enhancing protein OS=Homo sapiens PE=2 SV=1 - [B7Z6B3_HUMAN]	17.95	4	4	6	156	17.74626	8.645996	1.3188	1.315	1.2916	1.3084658	0.000759482	0.0242397
Q59GN8	PTK2 protein tyrosine kinase 2 isoform b variant (Fragment) OS=Homo sapiens PE=2 SV=1 - [Q59GN8_HUMAN]	8.31	6	6	6	975	110.1639	6.214355	1.38943	1.1891	1.3438	1.3074461	0.0367465	0.0856659
H0Y8G5	Heterogeneous nuclear ribonucleoprotein D0 (Fragment) OS=Homo sapiens GN=HNRNPD PE=1 SV=6 - [H0Y8G5_HUMAN]	39.23	8	10	16	260	29.64853	9.158691	1.28252	1.35877	1.27846	1.3065868	0.00718027	0.0439255
E9PPH5	Acidic leucine-rich nuclear phosphoprotein 32 family member E (Fragment) OS=Homo sapiens GN=ANP32E PE=1 SV=1 - [E9PPH5_HUMAN]	26.32	3	3	3	114	13.12044	4.221191	1.26037	1.35642	1.30084	1.3058792	0.00818338	0.0456887
P15153	Ras-related C3 botulinum toxin substrate 2 OS=Homo sapiens GN=RAC2 PE=1 SV=1 - [RAC2_HUMAN]	22.92	2	5	11	192	21.41508	7.605957	1.299	1.36896	1.24475	1.3042378	0.0136801	0.0523233

Biology Open • Supplementary information

Q15907	Ras-related protein Rab-11B OS=Homo sapiens GN=RAB11B PE=1 SV=4 - [RB11B_HUMAN]	33.94	7	7	11	218	24.47349	5.935059	1.28338	1.34835	1.27996	1.3038953	0.00531643	0.0401071
P62906	60S ribosomal protein L10a OS=Homo sapiens GN=RPL10A PE=1 SV=2 - [RL10A_HUMAN]	19.82	5	5	10	217	24.81554	9.935059	1.28566	1.33775	1.28717	1.303529	0.00316474	0.0364812
Q13404	Ubiquitin-conjugating enzyme E2 variant 1 OS=Homo sapiens GN=UBE2V1 PE=1 SV=2 - [UB2V1_HUMAN]	11.56	2	2	7	147	16.48433	7.928223	1.31381	1.40853	1.18383	1.3020554	0.0434837	0.0942707
A0A0G2JMH6	Uncharacterized protein OS=Homo sapiens GN=HLA-DRA PE=1 SV=1 - [A0A0G2JMH6_HUMAN]	28.35	6	7	17	254	28.60271	4.995605	1.29927	1.32313	1.28358	1.3019925	0.0014463	0.0287984
P21796	Voltage-dependent anion-selective channel protein 1 OS=Homo sapiens GN=VDAC1 PE=1 SV=2 - [VDAC1_HUMAN]	42.76	10	11	21	283	30.75357	8.543457	1.28483	1.37827	1.23763	1.3002442	0.0184194	0.0595261
P08779	Keratin, type I cytoskeletal 16 OS=Homo sapiens GN=KRT16 PE=1 SV=4 - [K1C16_HUMAN]	23.89	3	12	19	473	51.2362	5.046387	1.11366	1.30224	1.48421	1.3000379	0.107084	0.1700185
P11678	Eosinophil peroxidase OS=Homo sapiens GN=EPX PE=1 SV=2 - [PERE_HUMAN]	21.68	12	15	33	715	80.98914	10.28662	1.34547	1.24148	1.31122	1.2993875	0.0102827	0.0487492
E1NZA1	Peroxisome proliferator activated receptor interacting complex protein OS=Homo sapiens GN=PRIC295 PE=2 SV=1 - [E1NZA1_HUMAN]	4.98	11	11	13	2671	292.5584	7.430176	1.32938	1.1624	1.40498	1.2989198	0.0529502	0.1059317
P06748	Nucleophosmin OS=Homo sapiens GN=NPM1 PE=1 SV=2 - [NPM_HUMAN]	29.25	8	8	20	294	32.55484	4.779785	1.29714	1.29258	1.30688	1.298866	0.000199147	0.0212234
X6RA14	S-formylglutathione hydrolase OS=Homo sapiens GN=ESD PE=1 SV=1 - [X6RA14_HUMAN]	21.34	5	5	6	253	28.20786	6.785645	1.24598	1.35885	1.29101	1.2986129	0.011854	0.0495204
A6NLN1	Polypyrimidine tract binding protein 1, isoform CRA_b OS=Homo sapiens GN=PTBP1 PE=1 SV=4 - [A6NLN1_HUMAN]	11.76	5	5	10	527	56.47535	9.378418	1.17359	1.38937	1.32665	1.2965361	0.0436673	0.0944597

Biology Open • Supplementary information

E7EX53	Ribosomal protein L15 (Fragment) OS=Homo sapiens GN=RPL15 PE=1 SV=1 - [E7EX53_HUMAN]	12.03	2	2	2	133	15.71247	11.00439	1.22606	1.39223	1.27107	1.296454	0.0268904	0.0725869
Q5H9A7	Metalloproteinase inhibitor 1 OS=Homo sapiens GN=TIMP1 PE=1 SV=1 - [Q5H9A7_HUMAN]	22.38	2	2	2	143	16.04685	8.074707	1.2716	1.32599	1.28136	1.2929826	0.00324934	0.036522
P09496	Clathrin light chain A OS=Homo sapiens GN=CLTA PE=1 SV=1 - [CLCA_HUMAN]	9.27	3	3	3	248	27.06003	4.513184	1.3183	1.3153	1.23956	1.2910523	0.00774293	0.045203
B2RDE1	cDNA, FLJ96568, highly similar to Homo sapiens tropomyosin 3 (TPM3), mRNA OS=Homo sapiens PE=2 SV=1 - [B2RDE1_HUMAN]	55.65	8	21	61	248	28.99973	4.754395	1.24661	1.31372	1.30869	1.2896751	0.00550388	0.0406782
P26038	Moesin OS=Homo sapiens GN=MSN PE=1 SV=3 - [MOES_HUMAN]	45.23	22	32	52	577	67.77779	6.404785	1.29868	1.30487	1.26477	1.2894396	0.00184929	0.0312267
B3KS36	cDNA FLJ35376 fis, clone SKMUS2004044, highly similar to Homo sapiens ribosomal protein L3 (RPL3), transcript variant 2, mRNA OS=Homo sapiens PE=2 SV=1 - [B3KS36_HUMAN]	21.19	7	7	13	354	40.11965	10.30127	1.35464	1.33095	1.18273	1.2894378	0.0328477	0.0802262
A8K2Y2	cDNA FLJ78120, highly similar to Homo sapiens eukaryotic translation initiation factor 2, subunit 3 gamma, 52kDa (EIF2S3), mRNA OS=Homo sapiens PE=2 SV=1 - [A8K2Y2_HUMAN]	15.25	6	6	7	472	51.0782	8.279785	1.24196	1.25441	1.36753	1.2879679	0.0187006	0.0599446
A8K2B4	cDNA FLJ77490, highly similar to Homo sapiens enabled homolog (Drosophila) (ENAH), transcript variant 2, mRNA OS=Homo sapiens PE=2 SV=1 - [A8K2B4_HUMAN]	5.09	3	3	3	570	63.95762	6.252441	1.27976	1.26488	1.31801	1.2875499	0.00301428	0.0360868
F5H157	Ras-related protein Rab-35 (Fragment) OS=Homo sapiens GN=RAB35 PE=1 SV=1 - [F5H157_HUMAN]	24.86	2	4	4	185	21.20086	8.309082	1.35165	1.3135	1.19657	1.2872386	0.0253818	0.0700223
P55769	NHP2-like protein 1 OS=Homo sapiens GN=SNU13 PE=1 SV=3 - [NH2L1_HUMAN]	30.47	3	3	4	128	14.16456	8.455566	1.2671	1.3226	1.27129	1.2869944	0.00384281	0.0387187
P19338	Nucleolin OS=Homo sapiens GN=NCL PE=1 SV=3 -	28.17	20	20	32	710	76.56836	4.703613	1.29945	1.3192	1.23805	1.2855636	0.00724089	0.0439255

Biology Open • Supplementary information

	[NUCL_HUMAN]													
H0YHC3	Nucleosome assembly protein 1-like 1 (Fragment) OS=Homo sapiens GN=NAP1L1 PE=1 SV=1 - [H0YHC3_HUMAN]	29.29	4	5	7	198	23.40282	4.805176	1.19171	1.37364	1.28859	1.2846509	0.0324391	0.0797651
O60493	Sorting nexin-3 OS=Homo sapiens GN=SNX3 PE=1 SV=3 - [SNX3_HUMAN]	21.6	3	4	5	162	18.75076	8.660645	1.26882	1.30137	1.28226	1.2841486	0.0011031	0.0276207
B4E0J3	cDNA FLJ58965, highly similar to Nonspecific lipid-transfer protein (EC 2.3.1.176) OS=Homo sapiens PE=2 SV=1 - [B4E0J3_HUMAN]	7.44	2	2	4	121	13.03048	5.566895	1.30642	1.24935	1.28848	1.2814166	0.00356587	0.0379524
B4DQI7	cDNA FLJ58042, highly similar to Protein NipSnap1 OS=Homo sapiens PE=2 SV=1 - [B4DQI7_HUMAN]	8.33	2	2	2	264	31.38682	9.217285	1.20332	1.30577	1.33311	1.2807356	0.0192319	0.0608411
P04233	HLA class II histocompatibility antigen gamma chain OS=Homo sapiens GN=CD74 PE=1 SV=3 - [HG2A_HUMAN]	7.77	2	2	3	296	33.49368	8.440918	1.23117	1.33576	1.27292	1.2799503	0.0115854	0.0494101
P12004	Proliferating cell nuclear antigen OS=Homo sapiens GN=PCNA PE=1 SV=1 - [PCNA_HUMAN]	20.69	4	4	6	261	28.75029	4.690918	1.16783	1.4591	1.2119	1.2796109	0.0909768	0.1520224
Q05682	Caldesmon OS=Homo sapiens GN=CALD1 PE=1 SV=3 - [CALD1_HUMAN]	36.82	5	36	60	793	93.17543	5.655762	1.23476	1.36389	1.2386	1.2790854	0.0223301	0.0647707
P55010	Eukaryotic translation initiation factor 5 OS=Homo sapiens GN=EIF5 PE=1 SV=2 - [IF5_HUMAN]	11.83	3	4	4	431	49.19181	5.57959	1.26159	1.31183	1.26164	1.2783545	0.00359669	0.0379524
P48426	Phosphatidylinositol 5-phosphate 4-kinase type-2 alpha OS=Homo sapiens GN=PIP4K2A PE=1 SV=2 - [PI42A_HUMAN]	5.91	2	2	3	406	46.19549	6.990723	1.29467	1.29613	1.2438	1.2782021	0.003803	0.0387187
J3QKW1	RAC-beta serine/threonine-protein kinase (Fragment) OS=Homo sapiens GN=AKT2 PE=1 SV=1 - [J3QKW1_HUMAN]	5.48	2	2	3	292	33.44805	6.800293	1.26741	1.37478	1.18571	1.2759678	0.0371756	0.0863586
Q8TCJ2	Dolichyl-diphosphooligosaccharide--protein glycosyltransferase subunit STT3B OS=Homo sapiens	4.96	4	5	6	826	93.61368	8.909668	1.24905	1.27736	1.30148	1.2759616	0.00300117	0.0360868

Biology Open • Supplementary information

	GN=STT3B PE=1 SV=1 - [STT3B_HUMAN]														
A0A0A0MSI0	Peroxiredoxin-1 (Fragment) OS=Homo sapiens GN=PRDX1 PE=1 SV=1 - [A0A0A0MSI0_HUMAN]	44.44	6	9	23	171	18.96367	6.91748	1.29933	1.14732	1.38096	1.2758692	0.0564225	0.1107189
G3XAL9	Solute carrier family 12 (Sodium/potassium/chloride transporters), member 2, isoform CRA_a OS=Homo sapiens GN=SLC12A2 PE=1 SV=1 - [G3XAL9_HUMAN]	2.96	2	2	2	1150	124.5129	6.087402	1.26865	1.27073	1.2876	1.2756614	0.000473203	0.0225605
P11021	78 kDa glucose-regulated protein OS=Homo sapiens GN=HSPA5 PE=1 SV=2 - [GRP78_HUMAN]	49.24	31	34	86	654	72.28844	5.160645	1.28428	1.28522	1.25517	1.2748872	0.00128533	0.0281615
P31947	14-3-3 protein sigma OS=Homo sapiens GN=SFN PE=1 SV=1 - [1433S_HUMAN]	29.84	3	7	24	248	27.75669	4.741699	1.31292	1.21883	1.29266	1.2748033	0.0106517	0.0490917
P59998	Actin-related protein 2/3 complex subunit 4 OS=Homo sapiens GN=ARPC4 PE=1 SV=3 - [ARPC4_HUMAN]	32.14	6	6	10	168	19.6543	8.42627	1.27389	1.29908	1.24855	1.2738388	0.00282476	0.0360252
Q53GZ6	Heat shock 70kDa protein 8 isoform 1 variant (Fragment) OS=Homo sapiens PE=2 SV=1 - [Q53GZ6_HUMAN]	46.59	19	26	75	646	70.85517	5.414551	1.24302	1.30483	1.26874	1.2721969	0.00430822	0.0394273
Q96C98	FHL3 protein OS=Homo sapiens GN=FHL3 PE=1 SV=1 - [Q96C98_HUMAN]	21.51	3	3	4	172	18.90557	7.605957	1.3418	1.22491	1.24256	1.2697564	0.0177076	0.0583076
A0A0B4J1Z1	Serine/arginine-rich-splicing factor 7 OS=Homo sapiens GN=SRSF7 PE=1 SV=1 - [A0A0B4J1Z1_HUMAN]	15.33	2	2	3	137	15.75282	9.803223	1.27769	1.25562	1.27192	1.2684059	0.000605588	0.0241782
P07686	Beta-hexosaminidase subunit beta OS=Homo sapiens GN=HEXB PE=1 SV=3 - [HEXB_HUMAN]	12.05	5	6	8	556	63.07118	6.756348	1.23238	1.36211	1.20824	1.267576	0.030436	0.0772223
P36955	Pigment epithelium-derived factor OS=Homo sapiens GN=SERPINF1 PE=1 SV=4 - [PEDF_HUMAN]	30.62	12	12	18	418	46.28331	6.379395	1.21623	1.30262	1.28357	1.267472	0.00946101	0.0472949
B3KNL2	RuvB-like 2 (E. coli), isoform CRA_d OS=Homo sapiens GN=RUVBL2 PE=2 SV=1 - [B3KNL2_HUMAN]	13.99	6	6	7	429	47.37444	5.516113	1.28529	1.3221	1.19367	1.2670218	0.0198429	0.061532
P54709	Sodium/potassium-transporting ATPase subunit beta-3	20.43	5	5	6	279	31.4921	8.353027	1.24598	1.30285	1.2519	1.266912	0.00454318	0.0398479

Biology Open • Supplementary information

	OS=Homo sapiens GN=ATP1B3 PE=1 SV=1 - [AT1B3_HUMAN]														
B4DL49	cDNA FLJ58073, moderately similar to Cathepsin B (EC 3.4.22.1) OS=Homo sapiens PE=2 SV=1 - [B4DL49_HUMAN]	24.18	5	5	7	273	30.74773	6.624512	1.24629	1.33107	1.22206	1.2664729	0.015033	0.0541924
P29692	Elongation factor 1-delta OS=Homo sapiens GN=EEF1D PE=1 SV=5 - [EF1D_HUMAN]	23.13	5	6	7	281	31.10278	5.008301	1.32886	1.19952	1.27029	1.2662221	0.0191633	0.0606808
Q96HX3	Dolichyl-diphosphooligosaccharide--protein glycosyltransferase subunit 1 (Fragment) OS=Homo sapiens PE=2 SV=1 - [Q96HX3_HUMAN]	35.39	17	17	22	568	64.54171	6.55127	1.27161	1.29747	1.22867	1.2659186	0.00564482	0.040913
C9JFR7	Cytochrome c (Fragment) OS=Homo sapiens GN=CYCS PE=1 SV=1 - [C9JFR7_HUMAN]	25.74	3	3	5	101	11.32596	9.656738	1.24452	1.27522	1.27393	1.2645584	0.00143259	0.0287984
Q92629	Delta-sarcoglycan OS=Homo sapiens GN=SGCD PE=1 SV=2 - [SGCD_HUMAN]	10.38	2	2	3	289	32.05085	9.114746	1.24819	1.2206	1.32455	1.2644471	0.0135417	0.0520894
O15144	Actin-related protein 2/3 complex subunit 2 OS=Homo sapiens GN=ARPC2 PE=1 SV=1 - [ARPC2_HUMAN]	33	12	12	20	300	34.31149	7.356934	1.26269	1.26219	1.26821	1.2643642	5.33E-05	0.0173763
P53621	Coatomer subunit alpha OS=Homo sapiens GN=COPA PE=1 SV=2 - [COPA_HUMAN]	14.79	14	14	20	1224	138.2579	7.664551	1.19835	1.38896	1.20575	1.2643534	0.0513663	0.1038679
Q9BS40	Latexin OS=Homo sapiens GN=LXN PE=1 SV=2 - [LXN_HUMAN]	12.16	2	2	3	222	25.73389	5.782715	1.2166	1.17932	1.39598	1.2639652	0.058603	0.1132038
I2G9G1	MHC class II antigen OS=Homo sapiens GN=HLA-DPA1 PE=1 SV=1 - [I2G9G1_HUMAN]	8.85	2	2	3	260	29.37691	5.312988	1.07799	1.38111	1.32826	1.2624543	0.106916	0.1698314
Q5TEC6	Histone H3 OS=Homo sapiens GN=HIST2H3PS2 PE=1 SV=1 - [Q5TEC6_HUMAN]	27.21	2	4	8	136	15.42055	11.26807	1.36427	1.21068	1.20905	1.2613331	0.0366681	0.0855421
H0YL43	Reticulocalbin-2 (Fragment) OS=Homo sapiens GN=RCN2 PE=1 SV=1 - [H0YL43_HUMAN]	25.81	2	3	3	155	17.70841	4.589355	1.28959	1.21427	1.27378	1.2592127	0.00773557	0.045203

Biology Open • Supplementary information

E9PJD9	60S ribosomal protein L27a OS=Homo sapiens GN=RPL27A PE=1 SV=1 - [E9PJD9_HUMAN]	38.46	4	4	8	91	10.1206	10.21338	1.21818	1.29187	1.26634	1.2587975	0.00689625	0.0434708
H7BXF3	Transformer-2 protein homolog beta (Fragment) OS=Homo sapiens GN=TRA2B PE=1 SV=1 - [H7BXF3_HUMAN]	16.54	2	2	3	127	15.22042	10.19873	1.16695	1.40252	1.20301	1.257493	0.0722752	0.1295676
Q07065	Cytoskeleton-associated protein 4 OS=Homo sapiens GN=CKAP4 PE=1 SV=2 - [CKAP4_HUMAN]	36.54	17	17	25	602	65.98273	5.922363	1.27262	1.25278	1.23848	1.2546294	0.001508	0.0290552
P09211	Glutathione S-transferase P OS=Homo sapiens GN=GSTP1 PE=1 SV=2 - [GSTP1_HUMAN]	48.57	7	7	11	210	23.34102	5.643066	1.22123	1.27067	1.27139	1.2544316	0.0042315	0.0391996
Q9HCB6	Spondin-1 OS=Homo sapiens GN=SPON1 PE=1 SV=2 - [SPON1_HUMAN]	8.05	5	5	6	807	90.9136	6.112793	1.26473	1.21134	1.28494	1.2536655	0.00740742	0.0445366
Q9NR45	Sialic acid synthase OS=Homo sapiens GN=NANS PE=1 SV=2 - [SIAS_HUMAN]	7.52	3	3	4	359	40.28146	6.741699	1.29161	1.21026	1.25846	1.2534424	0.00857197	0.0458391
O94979	Protein transport protein Sec31A OS=Homo sapiens GN=SEC31A PE=1 SV=3 - [SC31A_HUMAN]	9.67	10	10	11	1220	132.931	6.888184	1.25008	1.31571	1.19346	1.2530831	0.0189284	0.0603321
P19105	Myosin regulatory light chain 12A OS=Homo sapiens GN=MYL12A PE=1 SV=2 - [ML12A_HUMAN]	56.14	3	8	31	171	19.78149	4.805176	1.24617	1.20724	1.30325	1.2522188	0.0119983	0.0495979
Q2NLC8	GSTK1 protein (Fragment) OS=Homo sapiens GN=GSTK1 PE=2 SV=1 - [Q2NLC8_HUMAN]	36.99	5	5	8	173	19.37218	8.689941	1.18792	1.29146	1.27355	1.250979	0.0158205	0.0551181
P04406	Glyceraldehyde-3-phosphate dehydrogenase OS=Homo sapiens GN=GAPDH PE=1 SV=3 - [G3P_HUMAN]	58.21	13	17	99	335	36.0304	8.455566	1.21586	1.28575	1.25062	1.2507448	0.00641245	0.0423724
Q15185	Prostaglandin E synthase 3 OS=Homo sapiens GN=PTGES3 PE=1 SV=1 - [TEBP_HUMAN]	18.75	2	2	6	160	18.68542	4.538574	1.30118	1.26241	1.18719	1.250263	0.0174121	0.05785
P49755	Transmembrane emp24 domain-containing protein 10 OS=Homo sapiens GN=TMED10 PE=1 SV=2 - [TMEDA_HUMAN]	27.4	5	5	9	219	24.96002	7.444824	1.17168	1.21148	1.36762	1.2502589	0.0526234	0.1054926
P20962	Parathymosin OS=Homo sapiens GN=PTMS PE=1 SV=2 -	22.55	3	3	4	102	11.52319	4.157715	1.27116	1.23072	1.24812	1.2499997	0.00218828	0.033949

Biology Open • Supplementary information

	[PTMS_HUMAN]													
P30837	Aldehyde dehydrogenase X, mitochondrial OS=Homo sapiens GN=ALDH1B1 PE=1 SV=3 - [AL1B1_HUMAN]	32.11	12	12	25	517	57.17023	6.800293	1.2561	1.25316	1.23422	1.2478285	0.000764796	0.0242397
Q9Y4L1	Hypoxia up-regulated protein 1 OS=Homo sapiens GN=HYOU1 PE=1 SV=1 - [HYOU1_HUMAN]	20.62	15	15	18	999	111.2662	5.224121	1.2226	1.21485	1.30572	1.2477229	0.0135053	0.0520677
B4DGN8	cDNA FLJ53377, highly similar to Procollagen-lysine, 2-oxoglutarate 5-dioxygenase 1 (EC 1.14.11.4) OS=Homo sapiens PE=2 SV=1 - [B4DGN8_HUMAN]	3.91	2	2	2	690	79.34919	7.02002	1.27768	1.23844	1.22125	1.2457898	0.00458532	0.0398629
Q09028	Histone-binding protein RBBP4 OS=Homo sapiens GN=RBBP4 PE=1 SV=3 - [RBBP4_HUMAN]	10.82	2	3	4	425	47.62607	4.894043	1.30434	1.24811	1.17872	1.2437256	0.0215043	0.0636851
P13796	Plastin-2 OS=Homo sapiens GN=LCP1 PE=1 SV=6 - [PLSL_HUMAN]	36.36	16	18	30	627	70.24389	5.427246	1.19468	1.26838	1.26643	1.2431603	0.00979864	0.0478721
Q92896	Golgi apparatus protein 1 OS=Homo sapiens GN=GLG1 PE=1 SV=2 - [GSLG1_HUMAN]	3.99	4	4	5	1179	134.4636	6.902832	1.23984	1.21011	1.27951	1.2431546	0.00676618	0.0433606
A7BI36	p180/ribosome receptor OS=Homo sapiens GN=RRBP1 PE=2 SV=2 - [A7BI36_HUMAN]	41.62	31	32	43	1540	165.6493	8.968262	1.2255	1.28467	1.21209	1.2407541	0.00846953	0.0456887
O43920	NADH dehydrogenase [ubiquinone] iron-sulfur protein 5 OS=Homo sapiens GN=NDUFS5 PE=1 SV=3 - [NDUS5_HUMAN]	22.64	2	2	2	106	12.50939	9.144043	1.178	1.2963	1.24702	1.2404374	0.0197582	0.0614155
P20674	Cytochrome c oxidase subunit 5A, mitochondrial OS=Homo sapiens GN=COX5A PE=1 SV=2 - [COX5A_HUMAN]	15.33	2	2	5	150	16.75169	6.785645	1.22516	1.22099	1.27429	1.2401473	0.00503911	0.0401071
B2RDE8	cDNA, FLJ96580, highly similar to Homo sapiens hepatoma-derived growth factor (high-mobility group protein 1-like) (HDGF), mRNA OS=Homo sapiens PE=2 SV=1 - [B2RDE8_HUMAN]	52.92	10	11	18	240	26.78885	4.665527	1.27358	1.2854	1.15953	1.2395019	0.0269495	0.0726885

Biology Open • Supplementary information

A0PJJ5	SRP72 protein (Fragment) OS=Homo sapiens GN=SRP72 PE=2 SV=1 - [A0PJJ5_HUMAN]	5.19	2	2	3	559	62.60458	8.32373	1.25217	1.22394	1.24226	1.239456	0.00118969	0.0276207
P26641	Elongation factor 1-gamma OS=Homo sapiens GN=EEF1G PE=1 SV=3 - [EF1G_HUMAN]	23.8	10	10	11	437	50.08714	6.668457	1.21374	1.27832	1.22449	1.2388484	0.00692378	0.0435632
P31949	Protein S100-A11 OS=Homo sapiens GN=S100A11 PE=1 SV=2 - [S10AB_HUMAN]	38.1	4	4	10	105	11.73283	7.122559	1.21266	1.22861	1.27519	1.2388204	0.00611288	0.0421458
P48735	Isocitrate dehydrogenase [NADP], mitochondrial OS=Homo sapiens GN=IDH2 PE=1 SV=2 - [IDHP_HUMAN]	30.97	11	11	15	452	50.87687	8.689941	1.29892	1.22998	1.18608	1.2383272	0.0184655	0.0595261
P08670	Vimentin OS=Homo sapiens GN=VIM PE=1 SV=4 - [VIME_HUMAN]	79.61	2	48	270	466	53.61908	5.122559	1.25729	1.33261	1.12368	1.2378577	0.0600817	0.1148371
P47813	Eukaryotic translation initiation factor 1A, X-chromosomal OS=Homo sapiens GN=EIF1AX PE=1 SV=2 - [IF1AX_HUMAN]	25	3	3	3	144	16.45023	5.236816	1.251	1.23588	1.22654	1.2378079	0.000896819	0.0250887
Q5T123	SH3 domain-binding glutamic acid-rich-like protein 3 OS=Homo sapiens GN=SH3BGRL3 PE=1 SV=1 - [Q5T123_HUMAN]	50	5	5	8	88	9.37476	9.36377	1.25726	1.2259	1.22971	1.2376255	0.00172385	0.0307118
P30040	Endoplasmic reticulum resident protein 29 OS=Homo sapiens GN=ERP29 PE=1 SV=4 - [ERP29_HUMAN]	26.44	5	5	10	261	28.97516	7.312988	1.18746	1.2265	1.29838	1.2374465	0.0182053	0.0591978
K7EJT5	60S ribosomal protein L22 (Fragment) OS=Homo sapiens GN=RPL22 PE=1 SV=1 - [K7EJT5_HUMAN]	51.06	2	2	6	47	5.079682	9.422363	1.26973	1.19642	1.24538	1.2371757	0.00815883	0.045649
B2R4K7	60S ribosomal protein L6 OS=Homo sapiens PE=2 SV=1 - [B2R4K7_HUMAN]	26.74	7	7	11	288	32.68163	10.62354	1.1294	1.39549	1.18395	1.2362768	0.100487	0.1627974
Q9BSV4	SFPQ protein (Fragment) OS=Homo sapiens GN=SFPQ PE=2 SV=2 - [Q9BSV4_HUMAN]	14.67	9	10	13	634	68.58792	8.821777	1.26418	1.23176	1.21229	1.2360735	0.00408474	0.0390309
Q9NQR4	Omega-amidase NIT2 OS=Homo sapiens GN=NIT2 PE=1 SV=1 - [NIT2_HUMAN]	25	5	5	7	276	30.58856	7.210449	1.17129	1.36161	1.17515	1.2360167	0.0640855	0.1192679

Biology Open • Supplementary information

P07195	L-lactate dehydrogenase B chain OS=Homo sapiens GN=LDHB PE=1 SV=2 - [LDHB_HUMAN]	42.51	12	14	33	334	36.61514	6.049316	1.23746	1.26042	1.21006	1.2359816	0.00378323	0.0387187
P49913	Cathelicidin antimicrobial peptide OS=Homo sapiens GN=CAMP PE=1 SV=1 - [CAMP_HUMAN]	11.76	2	2	2	170	19.28916	9.407715	1.31302	1.32463	1.07019	1.2359489	0.104559	0.167423
F5H0N4	Peptidyl-prolyl cis-trans isomerase (Fragment) OS=Homo sapiens GN=FKBP2 PE=1 SV=1 - [F5H0N4_HUMAN]	20.59	2	2	3	102	11.28286	9.246582	1.29399	1.31353	1.10026	1.2359263	0.074104	0.1316065
P34932	Heat shock 70 kDa protein 4 OS=Homo sapiens GN=HSPA4 PE=1 SV=4 - [HSP74_HUMAN]	20.48	12	13	19	840	94.27124	5.186035	1.21942	1.27904	1.20713	1.2351949	0.00879698	0.046376
Q9UBS4	DnaJ homolog subfamily B member 11 OS=Homo sapiens GN=DNAJB11 PE=1 SV=1 - [DJB11_HUMAN]	7.54	2	2	2	358	40.48862	6.17627	1.24649	1.21881	1.23821	1.2345041	0.00122077	0.0276207
B3KN05	cDNA FLJ13129 fis, clone NT2RP3002969, highly similar to Long-chain-fatty-acid--CoA ligase 3 (EC 6.2.1.3) OS=Homo sapiens PE=2 SV=1 - [B3KN05_HUMAN]	4.8	2	2	2	709	79.17174	8.32373	1.20519	1.28596	1.21203	1.2343934	0.0119522	0.0495204
G3V4W0	Heterogeneous nuclear ribonucleoproteins C1/C2 (Fragment) OS=Homo sapiens GN=HNRNPC PE=1 SV=1 - [G3V4W0_HUMAN]	37.02	10	10	20	262	28.89848	8.396973	1.25017	1.22769	1.22423	1.2340268	0.00120503	0.0276207
A0A087WWY0	Apolipoprotein(a) OS=Homo sapiens GN=LPA PE=1 SV=1 - [A0A087WWY0_HUMAN]	20.14	2	2	6	571	62.51328	5.731934	1.3002	1.16316	1.23633	1.2332319	0.0276261	0.0735754
B7ZLY3	LTBP1 protein OS=Homo sapiens GN=LTBP1 PE=2 SV=1 - [B7ZLY3_HUMAN]	9.61	9	9	12	1353	148.3643	5.097168	1.21346	1.27745	1.20832	1.2330772	0.00897991	0.0464786
D6R9P3	Heterogeneous nuclear ribonucleoprotein A/B OS=Homo sapiens GN=HNRNPAB PE=1 SV=1 - [D6R9P3_HUMAN]	22.86	4	5	10	280	30.28398	7.913574	1.17725	1.31297	1.20881	1.2330107	0.0295996	0.0761831
B4DSZ2	cDNA FLJ50653, highly similar to Rattus norvegicus CUG triplet repeat, RNA binding protein 2 (Cugbp2), mRNA OS=Homo sapiens PE=2 SV=1 - [B4DSZ2_HUMAN]	13.84	2	2	2	159	18.01997	7.752441	1.17665	1.24776	1.27374	1.2327179	0.015195	0.0543533

Biology Open • Supplementary information

B4DFY5	Mitogen-activated protein kinase kinase 1, isoform CRA_d OS=Homo sapiens GN=MAP2K1 PE=2 SV=1 - [B4DFY5_HUMAN]	15.9	4	5	6	371	41.35226	6.624512	1.24221	1.27042	1.18507	1.2325683	0.0114521	0.0494101
D3DTE6	SH3 domain-binding glutamic acid-rich-like protein OS=Homo sapiens GN=SH3BGRL PE=3 SV=1 - [D3DTE6_HUMAN]	45.13	6	6	8	113	12.69534	5.249512	1.17072	1.2488	1.27807	1.2325286	0.018461	0.0595261
X5D7K9	Major vault protein isoform C (Fragment) OS=Homo sapiens GN=MVP PE=2 SV=1 - [X5D7K9_HUMAN]	30.11	18	18	28	827	91.90301	5.414551	1.21437	1.28701	1.19526	1.2322131	0.0141785	0.0528571
B4DE30	cDNA FLJ51711, highly similar to T-complex protein 1 subunit epsilon OS=Homo sapiens PE=2 SV=1 - [B4DE30_HUMAN]	30.47	11	12	18	466	51.5117	5.528809	1.22645	1.24003	1.22925	1.2319076	0.00031832	0.0212234
P43490	Nicotinamide phosphoribosyltransferase OS=Homo sapiens GN=NAMPT PE=1 SV=1 - [NAMPT_HUMAN]	23.42	9	9	15	491	55.4866	7.151855	1.24763	1.22892	1.2167	1.2310832	0.0015107	0.0290552
P61081	NEDD8-conjugating enzyme Ubc12 OS=Homo sapiens GN=UBE2M PE=1 SV=1 - [UBC12_HUMAN]	18.03	3	3	5	183	20.88665	7.693848	1.2325	1.29668	1.16185	1.2303442	0.0274028	0.0733849
B1AK87	Capping protein (Actin filament) muscle Z-line, beta, isoform CRA_a OS=Homo sapiens GN=CAPZB PE=1 SV=1 - [B1AK87_HUMAN]	33.08	9	9	14	260	29.27683	6.91748	1.21001	1.25973	1.22116	1.2302982	0.00425	0.0391996
K7ESE8	Bleomycin hydrolase (Fragment) OS=Homo sapiens GN=BLMH PE=1 SV=1 - [K7ESE8_HUMAN]	10.82	2	2	2	231	26.72111	6.785645	1.30924	1.20783	1.17221	1.2297584	0.0304668	0.0772223
V9HWK1	Triosephosphate isomerase OS=Homo sapiens GN=HEL-S-49 PE=2 SV=1 - [V9HWK1_HUMAN]	63.05	15	15	31	249	26.65274	6.902832	1.22363	1.26077	1.20157	1.2286569	0.00565837	0.040913
B3KM36	cDNA FLJ10153 fis, clone HEMBA1003417, highly similar to BAG family molecular chaperone regulator 2 OS=Homo sapiens PE=2 SV=1 - [B3KM36_HUMAN]	22.75	5	5	5	211	23.69716	6.697754	1.29197	1.14643	1.24679	1.2284	0.0336776	0.0810225
J3KMX5	40S ribosomal protein S13 OS=Homo sapiens GN=RPS13 PE=1 SV=1 - [J3KMX5_HUMAN]	31.76	5	5	14	148	16.72226	10.50635	1.29479	1.16442	1.22192	1.2270433	0.026513	0.0717972
P15144	Aminopeptidase N OS=Homo sapiens GN=ANPEP PE=1	3.62	3	3	3	967	109.4709	5.478027	1.18246	1.30309	1.19456	1.2267023	0.0274478	0.0734473

	SV=4 - [AMPN_HUMAN]												
B3KUZ8	Aspartate aminotransferase OS=Homo sapiens PE=2 SV=1 - [B3KUZ8_HUMAN]	19.95	6	6	8	371	41.30008	8.836426	1.21143	1.22855	1.2395	1.2264898	0.00129784	0.0281615
Q9BTA4	Epididymis secretory protein Li 286 (Fragment) OS=Homo sapiens GN=HEL-S-286 PE=2 SV=1 - [Q9BTA4_HUMAN]	64.29	10	10	42	168	17.81053	8.645996	1.19995	1.25609	1.22318	1.2264104	0.00513447	0.0401071
Q3SYF1	Sorting nexin 12 OS=Homo sapiens GN=SNX12 PE=2 SV=1 - [Q3SYF1_HUMAN]	25.31	3	4	5	162	18.87273	8.440918	1.18771	1.22587	1.26476	1.2261145	0.00953767	0.0474082
A0A024R462	Fibronectin 1, isoform CRA_n OS=Homo sapiens GN=FN1 PE=4 SV=1 - [A0A024R462_HUMAN]	33.29	58	58	151	2355	259.0485	5.731934	1.19705	1.24095	1.23843	1.2254737	0.00396085	0.0390309
Q9BY32	Inosine triphosphate pyrophosphatase OS=Homo sapiens GN=ITPA PE=1 SV=2 - [ITPA_HUMAN]	14.95	2	2	2	194	21.4319	5.655762	1.15592	1.28462	1.23581	1.2254491	0.0265871	0.0719403
E9PBS1	Multifunctional protein ADE2 (Fragment) OS=Homo sapiens GN=PAICS PE=1 SV=1 - [E9PBS1_HUMAN]	10.17	4	4	4	413	45.62237	6.683105	1.18544	1.23868	1.25084	1.2249851	0.00787458	0.0452827
P46781	40S ribosomal protein S9 OS=Homo sapiens GN=RPS9 PE=1 SV=3 - [RS9_HUMAN]	23.2	7	7	12	194	22.57756	10.65283	1.22075	1.24906	1.20309	1.224296	0.00354446	0.0378724
B1AK13	3-hydroxymethyl-3-methylglutaryl-Coenzyme A lyase (Hydroxymethylglutaricaciduria), isoform CRA_b OS=Homo sapiens GN=HMGCL PE=2 SV=1 - [B1AK13_HUMAN]	9.33	2	2	2	300	31.71435	7.605957	1.26569	1.15671	1.24636	1.2229183	0.0219385	0.064296
C9J8E1	MAP kinase-activated protein kinase 3 (Fragment) OS=Homo sapiens GN=MAPKAPK3 PE=1 SV=1 - [C9J8E1_HUMAN]	6.02	2	2	2	332	37.36568	6.521973	1.21556	1.38152	1.07097	1.2226819	0.13114	0.1962462
J3QS48	Mannose-P-dolichol utilization defect 1 protein OS=Homo sapiens GN=MPDU1 PE=1 SV=1 - [J3QS48_HUMAN]	23.76	2	2	2	101	10.9711	8.484863	1.20965	1.25007	1.20575	1.2218229	0.00405527	0.0390309
S6C4R7	IgG L chain OS=Homo sapiens PE=2 SV=1 - [S6C4R7_HUMAN]	32.08	2	6	56	212	22.49917	8.23584	1.19289	1.23156	1.2396	1.2213524	0.00421555	0.0391996
B4DKL5	cDNA FLJ51983, highly similar to Phosphoglycerate mutase 1 (EC 5.4.2.1) OS=Homo sapiens PE=2 SV=1 -	46.86	9	9	21	239	27.0799	8.23584	1.1618	1.24737	1.25424	1.2211351	0.0176042	0.0581368

Biology Open • Supplementary information

	[B4DKL5_HUMAN]													
B2R9X3	cDNA, FLJ94599, highly similar to Homo sapiens GDP-mannose 4,6-dehydratase (GMDS), mRNA OS=Homo sapiens PE=2 SV=1 - [B2R9X3_HUMAN]	6.72	2	2	2	372	41.82417	7.122559	1.22077	1.24742	1.19448	1.2208895	0.0047529	0.0400965
B4E0R1	Integrin beta OS=Homo sapiens PE=2 SV=1 - [B4E0R1_HUMAN]	13.71	7	7	7	700	77.30442	6.888184	1.1498	1.28101	1.23149	1.2207693	0.0287368	0.0750572
Q59FQ9	DDX19-like protein variant (Fragment) OS=Homo sapiens PE=2 SV=1 - [Q59FQ9_HUMAN]	5.43	2	2	2	313	35.59341	7.942871	1.20557	1.20996	1.24649	1.2206739	0.00343795	0.0372192
P36543	V-type proton ATPase subunit E 1 OS=Homo sapiens GN=ATP6V1E1 PE=1 SV=1 - [VATE1_HUMAN]	19.03	4	4	5	226	26.12876	8.001465	1.27223	1.20985	1.17805	1.2200443	0.015435	0.0546326
Q01995	Transgelin OS=Homo sapiens GN=TAGLN PE=1 SV=4 - [TAGL_HUMAN]	80.6	18	19	149	201	22.59643	8.836426	1.18936	1.28329	1.18595	1.2195341	0.0204575	0.0624988
B4DW90	cDNA FLJ58737, highly similar to Splicing factor 3A subunit 3 OS=Homo sapiens PE=2 SV=1 - [B4DW90_HUMAN]	8.04	3	3	4	448	52.39507	5.274902	1.21127	1.18272	1.26421	1.2194	0.0116339	0.0494101
B4DJV2	Citrate synthase OS=Homo sapiens GN=CS PE=1 SV=1 - [B4DJV2_HUMAN]	18.54	7	8	13	453	50.39972	7.898926	1.22104	1.22502	1.21184	1.219298	0.000316731	0.0212234
B4DZZ1	cDNA FLJ57836, highly similar to Myb-binding protein 1A OS=Homo sapiens PE=2 SV=1 - [B4DZZ1_HUMAN]	3.81	2	2	3	603	67.51612	9.715332	1.14955	1.344	1.16379	1.2191136	0.0727789	0.129798
B4DU58	cDNA FLJ51488, highly similar to Macrophage capping protein OS=Homo sapiens PE=2 SV=1 - [B4DU58_HUMAN]	21.41	6	6	12	327	36.22537	6.252441	1.24447	1.2704	1.14153	1.2188033	0.0308592	0.0773766
P49720	Proteasome subunit beta type-3 OS=Homo sapiens GN=PSMB3 PE=1 SV=2 - [PSB3_HUMAN]	27.32	4	4	6	205	22.93345	6.55127	1.21808	1.2766	1.15597	1.2168849	0.0248295	0.0690048
P02533	Keratin, type I cytoskeletal 14 OS=Homo sapiens GN=KRT14 PE=1 SV=4 - [K1C14_HUMAN]	27.12	3	13	20	472	51.52939	5.160645	1.3097	1.08503	1.25566	1.2167963	0.085243	0.145584
P62834	Ras-related protein Rap-1A OS=Homo sapiens GN=RAP1A	43.48	2	8	13	184	20.97371	6.668457	1.26449	1.30303	1.0828	1.2167741	0.0856992	0.1459949

Biology Open • Supplementary information

	PE=1 SV=1 - [RAP1A_HUMAN]													
Q96QR8	Transcriptional activator protein Pur-beta OS=Homo sapiens GN=PURB PE=1 SV=3 - [PURB_HUMAN]	7.05	2	3	3	312	33.22048	5.427246	1.19914	1.23801	1.21253	1.2165575	0.00275948	0.0359263
P35908	Keratin, type II cytoskeletal 2 epidermal OS=Homo sapiens GN=KRT2 PE=1 SV=2 - [K22E_HUMAN]	33.33	14	20	33	639	65.39322	8.001465	1.21487	1.28647	1.14825	1.216529	0.0323331	0.0797133
P10809	60 kDa heat shock protein, mitochondrial OS=Homo sapiens GN=HSPD1 PE=1 SV=2 - [CH60_HUMAN]	49.91	2	21	35	573	61.01639	5.871582	1.29609	1.17484	1.17638	1.2157707	0.0329431	0.0802262
Q9UMS6	Synaptopodin-2 OS=Homo sapiens GN=SYNPO2 PE=1 SV=2 - [SYNP2_HUMAN]	24.25	2	20	33	1093	117.4414	8.572754	1.19342	1.3329	1.11845	1.2149249	0.0758689	0.1339678
P54577	Tyrosine--tRNA ligase, cytoplasmic OS=Homo sapiens GN=YARS PE=1 SV=4 - [SYYC_HUMAN]	10.42	6	6	8	528	59.1061	7.049316	1.2043	1.2497	1.18891	1.2143038	0.0071728	0.0439255
B4E022	cDNA FLJ56274, highly similar to Transketolase (EC 2.2.1.1) OS=Homo sapiens PE=2 SV=1 - [B4E022_HUMAN]	36.11	20	20	34	576	62.83929	7.518066	1.15837	1.28517	1.1981	1.2138822	0.0293115	0.0759139
H0Y6E7	RNA-binding motif protein, X chromosome (Fragment) OS=Homo sapiens GN=RBMX PE=1 SV=2 - [H0Y6E7_HUMAN]	19.18	5	5	11	292	31.83638	9.847168	1.22419	1.24798	1.16933	1.2138315	0.0116527	0.0494101
P08729	Keratin, type II cytoskeletal 7 OS=Homo sapiens GN=KRT7 PE=1 SV=5 - [K2C7_HUMAN]	10.02	2	7	12	469	51.35431	5.478027	1.27055	1.12764	1.24289	1.2136934	0.0394709	0.0890226
B2RAY1	cDNA, FLJ95184, highly similar to Homo sapiens signal transducing adaptor molecule (SH3 domain and ITAM motif) 1 (STAM), mRNA OS=Homo sapiens PE=2 SV=1 - [B2RAY1_HUMAN]	4.81	2	2	2	540	59.11207	4.843262	1.24067	1.20137	1.19856	1.2135348	0.00402856	0.0390309
P12111	Collagen alpha-3(VI) chain OS=Homo sapiens GN=COL6A3 PE=1 SV=5 - [CO6A3_HUMAN]	37.93	7	111	484	3177	343.4568	6.683105	1.17635	1.28306	1.18108	1.2134992	0.0255677	0.0704204
F8W7C6	60S ribosomal protein L10 OS=Homo sapiens GN=RPL10	20.25	4	4	7	163	18.55272	9.949707	1.23379	1.16677	1.23854	1.2130345	0.0116245	0.0494101

Biology Open • Supplementary information

	PE=1 SV=2 - [F8W7C6_HUMAN]														
Q68D08	Putative uncharacterized protein DKFZp686B04128 OS=Homo sapiens GN=DKFZp686B04128 PE=2 SV=1 - [Q68D08_HUMAN]	11.42	2	3	4	324	36.72663	6.062012	1.21757	1.18284	1.23807	1.2128246	0.0056875	0.040913
P49588	Alanine--tRNA ligase, cytoplasmic OS=Homo sapiens GN=AARS PE=1 SV=2 - [SYAC_HUMAN]	19.42	14	14	16	968	106.7432	5.528809	1.25896	1.19292	1.18295	1.2116107	0.0124629	0.0505179
B2R9U2	cDNA, FLJ94557, highly similar to Homo sapiens FK506 binding protein 4, 59kDa (FKBP4), mRNA OS=Homo sapiens PE=2 SV=1 - [B2R9U2_HUMAN]	19.39	7	7	9	459	51.80208	5.427246	1.29646	1.19154	1.14595	1.2113156	0.0417073	0.092274
P37802	Transgelin-2 OS=Homo sapiens GN=TAGLN2 PE=1 SV=3 - [TAGL2_HUMAN]	78.89	14	14	48	199	22.37717	8.250488	1.22826	1.20521	1.20019	1.2112216	0.00167046	0.0304006
J3QLE5	Small nuclear ribonucleoprotein-associated protein N (Fragment) OS=Homo sapiens GN=SNRPN PE=4 SV=1 - [J3QLE5_HUMAN]	21.89	4	4	6	169	17.53528	9.993652	1.09843	1.27715	1.25731	1.2109654	0.064946	0.1201788
O00151	PDZ and LIM domain protein 1 OS=Homo sapiens GN=PDLIM1 PE=1 SV=4 - [PDLI1_HUMAN]	43.77	12	12	20	329	36.04904	7.02002	1.15589	1.28692	1.18485	1.2092178	0.0342357	0.0816687
B4DPW9	cDNA FLJ51929, highly similar to Plastin-3 OS=Homo sapiens PE=2 SV=1 - [B4DPW9_HUMAN]	26.53	12	14	24	603	67.55656	5.655762	1.21485	1.19424	1.21773	1.2089418	0.00125149	0.0276882
A0A087WTL2	Armadillo repeat-containing X-linked protein 1 OS=Homo sapiens GN=ARMCX1 PE=1 SV=1 - [A0A087WTL2_HUMAN]	6.03	2	2	2	365	38.97997	9.085449	1.26109	1.17606	1.18964	1.2089295	0.0155628	0.0547825
A8KAM5	cDNA FLJ77519, highly similar to Homo sapiens secreted frizzled related protein mRNA OS=Homo sapiens PE=2 SV=1 - [A8KAM5_HUMAN]	10.22	3	3	3	313	35.29102	8.851074	1.17118	1.27726	1.17808	1.2088429	0.0258833	0.0710008
B1ALD9	Periostin OS=Homo sapiens GN=POSTN PE=1 SV=1 - [B1ALD9_HUMAN]	36.14	3	24	46	808	90.08663	7.942871	1.24459	1.18654	1.19472	1.2086156	0.00747658	0.0446031

Biology Open • Supplementary information

Q96BA4	NUCB1 protein OS=Homo sapiens PE=1 SV=1 - [Q96BA4_HUMAN]	30.68	4	4	4	176	20.45616	5.109863	1.19843	1.15206	1.27446	1.2083183	0.0281037	0.0742152
P62318	Small nuclear ribonucleoprotein Sm D3 OS=Homo sapiens GN=SNRPD3 PE=1 SV=1 - [SMD3_HUMAN]	15.08	2	2	4	126	13.9073	10.31592	1.18098	1.25823	1.18436	1.2078576	0.0143899	0.0531187
U3KPR7	Calpain small subunit 1 (Fragment) OS=Homo sapiens GN=CAPNS1 PE=1 SV=1 - [U3KPR7_HUMAN]	27	3	3	5	100	11.39149	5.414551	1.21732	1.33443	1.07055	1.2074358	0.11294	0.1763385
Q9Y371	Endophilin-B1 OS=Homo sapiens GN=SH3GLB1 PE=1 SV=1 - [SHLB1_HUMAN]	8.49	3	3	3	365	40.77078	6.036621	1.16213	1.24438	1.21578	1.2074295	0.0132398	0.051412
P61158	Actin-related protein 3 OS=Homo sapiens GN=ACTR3 PE=1 SV=3 - [ARP3_HUMAN]	48.8	17	17	33	418	47.34098	5.884277	1.24796	1.24861	1.12462	1.2070621	0.0374233	0.086647
P07942	Laminin subunit beta-1 OS=Homo sapiens GN=LAMB1 PE=1 SV=2 - [LAMB1_HUMAN]	13.1	17	17	22	1786	197.9086	4.944824	1.19097	1.24679	1.18269	1.2068183	0.00933859	0.0472949
B4DFG0	Protein DEK OS=Homo sapiens GN=DEK PE=1 SV=1 - [B4DFG0_HUMAN]	9.22	3	3	5	347	39.47627	8.51416	1.09999	1.32189	1.19708	1.206321	0.0847536	0.1450372
B4DE36	Glucose-6-phosphate isomerase OS=Homo sapiens PE=2 SV=1 - [B4DE36_HUMAN]	26.42	13	13	23	530	60.14769	8.147949	1.19585	1.23546	1.18765	1.2063209	0.00507765	0.0401071
Q0VGA5	SARS protein OS=Homo sapiens GN=SARS PE=2 SV=1 - [Q0VGA5_HUMAN]	10.76	5	5	6	511	58.36983	6.430176	1.19631	1.22852	1.19359	1.2061369	0.00294812	0.0360523
P17987	T-complex protein 1 subunit alpha OS=Homo sapiens GN=TCP1 PE=1 SV=1 - [TCPA_HUMAN]	27.88	10	10	14	556	60.30559	6.112793	1.1883	1.22534	1.20398	1.2058718	0.00270809	0.0357839
Q9BRA2	Thioredoxin domain-containing protein 17 OS=Homo sapiens GN=TXNDC17 PE=1 SV=1 - [TXD17_HUMAN]	26.83	3	3	7	123	13.9318	5.516113	1.14642	1.27651	1.19391	1.2056135	0.032508	0.0798294
B4DEA6	cDNA FLJ56566, highly similar to Small glutamine-rich tetratricopeptiderepeat-containing protein A OS=Homo sapiens PE=2 SV=1 - [B4DEA6_HUMAN]	12.71	3	3	3	291	31.40945	5.36377	1.1617	1.27037	1.18466	1.2055734	0.0249111	0.0690615

Biology Open • Supplementary information

D6RAN4	60S ribosomal protein L9 (Fragment) OS=Homo sapiens GN=RPL9 PE=1 SV=6 - [D6RAN4_HUMAN]	22.1	3	3	6	181	20.76234	10.19873	1.23008	1.26969	1.11465	1.2048078	0.0478868	0.0996284
Q9BVG4	Protein PBDC1 OS=Homo sapiens GN=PBDC1 PE=1 SV=1 - [PBDC1_HUMAN]	13.3	3	3	3	233	26.04069	4.79248	1.19682	1.21878	1.19837	1.2046593	0.00119346	0.0276207
G3V3M6	DNA-(apurinic or apyrimidinic site) lyase (Fragment) OS=Homo sapiens GN=APEX1 PE=1 SV=1 - [G3V3M6_HUMAN]	27.76	5	5	5	263	29.17194	7.327637	1.20634	1.2351	1.17209	1.2045077	0.00783794	0.045203
Q15555	Microtubule-associated protein RP/EB family member 2 OS=Homo sapiens GN=MAPRE2 PE=1 SV=1 - [MARE2_HUMAN]	16.51	3	4	5	327	37.0082	5.566895	1.14256	1.21461	1.25383	1.2036673	0.024655	0.0688023
P13645	Keratin, type I cytoskeletal 10 OS=Homo sapiens GN=KRT10 PE=1 SV=6 - [K1C10_HUMAN]	42.64	20	22	51	584	58.7917	5.211426	1.22997	1.20165	1.17861	1.2034082	0.0052886	0.0401071
H0YN67	Immunoglobulin superfamily-containing leucine-rich repeat protein (Fragment) OS=Homo sapiens GN=ISLR PE=1 SV=1 - [H0YN67_HUMAN]	24.49	3	3	3	147	16.22829	5.427246	1.22148	1.26112	1.12506	1.2025529	0.0375582	0.0868405
B4DRU0	Eukaryotic translation initiation factor 3 subunit C OS=Homo sapiens GN=EIF3C PE=2 SV=1 - [B4DRU0_HUMAN]	7.07	4	4	4	735	84.91315	6.814941	1.25204	1.25701	1.09759	1.2022135	0.0609211	0.1155283
Q15365	Poly(rC)-binding protein 1 OS=Homo sapiens GN=PCBP1 PE=1 SV=2 - [PCBP1_HUMAN]	34.27	5	9	20	356	37.47395	7.093262	1.17995	1.26805	1.15824	1.2020768	0.0265122	0.0717972
P14174	Macrophage migration inhibitory factor OS=Homo sapiens GN=MIF PE=1 SV=4 - [MIF_HUMAN]	17.39	2	2	5	115	12.46822	7.884277	1.15536	1.33106	1.11927	1.2018945	0.090897	0.152019
O15511	Actin-related protein 2/3 complex subunit 5 OS=Homo sapiens GN=ARPC5 PE=1 SV=3 - [ARPC5_HUMAN]	62.25	6	6	10	151	16.3103	5.668457	1.21886	1.20791	1.17827	1.2016832	0.0035944	0.0379524
A0A0J9YYL3	Poly(U)-binding-splicing factor PUF60 (Fragment) OS=Homo sapiens GN=PUF60 PE=1 SV=1 - [A0A0J9YYL3_HUMAN]	8.12	3	3	5	505	54.59203	5.338379	1.24015	1.25479	1.10918	1.201375	0.0489879	0.1012357

Biology Open • Supplementary information

B4E043	KH domain containing, RNA binding, signal transduction associated 1, isoform CRA_b OS=Homo sapiens GN=KHDRBS1 PE=2 SV=1 - [B4E043_HUMAN]	8.65	3	3	4	347	38.65835	6.468262	1.23186	1.20247	1.16783	1.2007227	0.00839188	0.0456887
B4DUQ1	cDNA FLJ54552, highly similar to Heterogeneous nuclear ribonucleoprotein K OS=Homo sapiens PE=2 SV=1 - [B4DUQ1_HUMAN]	43.05	17	17	37	439	48.48019	5.922363	1.21102	1.17835	1.21272	1.2006956	0.00308953	0.0360868
G3V203	60S ribosomal protein L18 OS=Homo sapiens GN=RPL18 PE=1 SV=1 - [G3V203_HUMAN]	35.37	5	5	10	164	18.74447	11.59033	1.17553	1.20376	1.22257	1.2006204	0.00461156	0.0398629
A0A0A7E7X3	Endoplasmic reticulum aminopeptidase 1 (Fragment) OS=Homo sapiens GN=ERAP1 PE=2 SV=1 - [A0A0A7E7X3_HUMAN]	7.55	6	6	7	941	107.1633	6.404785	1.22061	1.26017	1.11593	1.1989024	0.0437535	0.0945757
H0Y2W2	ATPase family AAA domain-containing protein 3A (Fragment) OS=Homo sapiens GN=ATAD3A PE=1 SV=1 - [H0Y2W2_HUMAN]	4.02	2	2	2	572	64.31427	9.437012	1.23049	1.21528	1.15091	1.1988906	0.0147086	0.0537094
B7Z593	cDNA FLJ52191, highly similar to Vacuolar ATP synthase subunit C (EC 3.6.3.14) OS=Homo sapiens PE=2 SV=1 - [B7Z593_HUMAN]	8.79	3	3	4	307	35.65568	8.23584	1.20439	1.21946	1.17269	1.1988475	0.00476915	0.0400965
P46063	ATP-dependent DNA helicase Q1 OS=Homo sapiens GN=RECQL PE=1 SV=3 - [RECQ1_HUMAN]	11.56	8	8	11	649	73.41	7.884277	1.1346	1.20994	1.25114	1.1985606	0.0282808	0.0744405
Q9NUP3	cDNA FLJ11224 fis, clone PLACE1008273, moderately similar to COATOMER GAMMA SUBUNIT OS=Homo sapiens PE=2 SV=1 - [Q9NUP3_HUMAN]	11.39	2	4	5	483	53.98971	6.214355	1.27548	1.17119	1.14884	1.1985027	0.0365417	0.0854261
Q96I99	Succinyl-CoA ligase [GDP-forming] subunit beta, mitochondrial OS=Homo sapiens GN=SUCLG2 PE=1 SV=2 - [SUCB2_HUMAN]	21.06	9	9	11	432	46.48144	6.39209	1.21564	1.20844	1.16867	1.1975836	0.00542051	0.0402547

Biology Open • Supplementary information

M0QZS6	SUMO-activating enzyme subunit 1 OS=Homo sapiens GN=SAE1 PE=1 SV=1 - [M0QZS6_HUMAN]	21.51	4	4	4	265	29.4041	4.98291	1.15248	1.22293	1.21621	1.1972047	0.0127094	0.0509731
O60701	UDP-glucose 6-dehydrogenase OS=Homo sapiens GN=UGDH PE=1 SV=1 - [UGDH_HUMAN]	9.31	4	4	5	494	54.98925	7.122559	1.32431	1.15191	1.11184	1.1960184	0.0950679	0.1563678
J3KTF8	Rho GDP-dissociation inhibitor 1 (Fragment) OS=Homo sapiens GN=ARHGDIA PE=1 SV=6 - [J3KTF8_HUMAN]	23.83	4	4	9	193	21.50395	5.490723	1.20496	1.1785	1.20173	1.1950598	0.00182066	0.0311259
P24534	Elongation factor 1-beta OS=Homo sapiens GN=EEF1B2 PE=1 SV=3 - [EF1B_HUMAN]	27.56	4	5	10	225	24.74826	4.665527	1.23152	1.25304	1.10019	1.1949201	0.0551396	0.1086526
A8K7F6	cDNA FLJ78244, highly similar to Homo sapiens eukaryotic translation initiation factor 4A, isoform 1 (EIF4A1), mRNA OS=Homo sapiens PE=2 SV=1 - [A8K7F6_HUMAN]	32.02	5	11	27	406	46.09259	5.478027	1.20861	1.08989	1.28613	1.1948748	0.0760877	0.1341442
O60739	Eukaryotic translation initiation factor 1b OS=Homo sapiens GN=EIF1B PE=1 SV=2 - [EIF1B_HUMAN]	13.27	2	2	2	113	12.81565	7.371582	1.21118	1.19662	1.17669	1.1948333	0.00262287	0.0357839
P22314	Ubiquitin-like modifier-activating enzyme 1 OS=Homo sapiens GN=UBA1 PE=1 SV=3 - [UBA1_HUMAN]	26.09	20	20	35	1058	117.7743	5.757324	1.18553	1.28259	1.11585	1.1946572	0.0565136	0.1108108
D6RAA6	Transmembrane protein 33 (Fragment) OS=Homo sapiens GN=TMEM33 PE=1 SV=1 - [D6RAA6_HUMAN]	9.01	2	2	3	222	25.20727	9.568848	1.09018	1.3554	1.13734	1.1943084	0.140444	0.2058021
M0QXS5	Heterogeneous nuclear ribonucleoprotein L (Fragment) OS=Homo sapiens GN=HNRNPL PE=1 SV=1 - [M0QXS5_HUMAN]	16.04	7	7	7	530	58.43531	6.785645	1.23253	1.21635	1.13358	1.1941548	0.0240196	0.0675863
Q00688	Peptidyl-prolyl cis-trans isomerase FKBP3 OS=Homo sapiens GN=FKBP3 PE=1 SV=1 - [FKBP3_HUMAN]	14.73	3	3	4	224	25.16132	9.275879	1.19344	1.22936	1.1571	1.1932975	0.0114454	0.0494101
F5GXX5	Dolichyl-diphosphooligosaccharide--protein glycosyltransferase subunit DAD1 OS=Homo sapiens GN=DAD1 PE=1 SV=1 - [F5GXX5_HUMAN]	25.88	2	2	3	85	9.548096	7.181152	1.13746	1.37202	1.07028	1.1932542	0.168969	0.236873

P62753	40S ribosomal protein S6 OS=Homo sapiens GN=RPS6 PE=1 SV=1 - [RS6_HUMAN]	22.49	5	5	6	249	28.66299	10.84326	1.18985	1.24812	1.14161	1.1931944	0.0244741	0.0684103
Q15435	Protein phosphatase 1 regulatory subunit 7 OS=Homo sapiens GN=PPP1R7 PE=1 SV=1 - [PP1R7_HUMAN]	14.17	6	6	7	360	41.53853	4.906738	1.19686	1.22614	1.15414	1.1923816	0.0116049	0.0494101
P08133	Annexin A6 OS=Homo sapiens GN=ANXA6 PE=1 SV=3 - [ANXA6_HUMAN]	66.12	17	41	101	673	75.82558	5.60498	1.11047	1.28054	1.18363	1.1915475	0.0602082	0.1148843
B4DQF6	Eukaryotic translation initiation factor 3 subunit L OS=Homo sapiens GN=EIF3L PE=2 SV=1 - [B4DQF6_HUMAN]	4.71	2	2	2	531	62.65148	6.163574	1.16236	1.2178	1.19186	1.1906748	0.00698021	0.0437556
P35579	Myosin-9 OS=Homo sapiens GN=MYH9 PE=1 SV=4 - [MYH9_HUMAN]	58.88	104	130	355	1960	226.3916	5.60498	1.18002	1.22977	1.16194	1.1905793	0.0111353	0.0493721
Q15075	Early endosome antigen 1 OS=Homo sapiens GN=EEA1 PE=1 SV=2 - [EEA1_HUMAN]	13.11	14	14	18	1411	162.3672	5.681152	1.1093	1.27082	1.18959	1.1899052	0.0553281	0.108887
H0Y3P2	Eukaryotic translation initiation factor 4 gamma 2 OS=Homo sapiens GN=EIF4G2 PE=1 SV=1 - [H0Y3P2_HUMAN]	10.01	8	8	10	869	98.05574	6.990723	1.17535	1.28683	1.10533	1.1891681	0.0699578	0.126432
P26373	60S ribosomal protein L13 OS=Homo sapiens GN=RPL13 PE=1 SV=4 - [RL13_HUMAN]	24.64	6	6	10	211	24.24653	11.64893	1.2078	1.18029	1.17899	1.1890259	0.00246205	0.0352411
A0A087WW59	Uncharacterized protein C11orf96 OS=Homo sapiens GN=C11orf96 PE=1 SV=1 - [A0A087WW59_HUMAN]	27.87	2	2	2	122	13.76393	8.177246	1.20191	1.24644	1.11836	1.1889029	0.0373029	0.0864865
B5BUI8	Dual specificity phosphatase 3 (Fragment) OS=Homo sapiens GN=DUSP3 PE=2 SV=1 - [B5BUI8_HUMAN]	30.27	5	5	7	185	20.56439	8.147949	1.17162	1.22343	1.17072	1.1885884	0.00842507	0.0456887
E9PN66	Tumor protein p53-inducible protein 11 (Fragment) OS=Homo sapiens GN=TP53I11 PE=1 SV=1 - [E9PN66_HUMAN]	41.94	3	3	4	62	6.860728	9.598145	1.18777	1.17155	1.20528	1.1882001	0.00266653	0.0357839
A0A087X271	Calponin (Fragment) OS=Homo sapiens GN=CNN2 PE=1 SV=1 - [A0A087X271_HUMAN]	30.73	4	5	7	179	19.74478	8.79248	1.15527	1.17796	1.22718	1.1868036	0.0126659	0.0508886
P27348	14-3-3 protein theta OS=Homo sapiens GN=YWHAQ PE=1 SV=1 - [1433T_HUMAN]	56.33	9	14	35	245	27.74677	4.779785	1.27128	1.21531	1.07314	1.1865751	0.0870569	0.1475652

Biology Open • Supplementary information

Q13409	Cytoplasmic dynein 1 intermediate chain 2 OS=Homo sapiens GN=DYNC1I2 PE=1 SV=3 - [DC1I2_HUMAN]	17.24	8	8	9	638	71.41211	5.19873	1.17423	1.11689	1.2685	1.1865406	0.0518122	0.1043663
P54886	Delta-1-pyrroline-5-carboxylate synthase OS=Homo sapiens GN=ALDH18A1 PE=1 SV=2 - [P5CS_HUMAN]	4.28	2	2	3	795	87.24755	7.122559	1.25617	1.05505	1.24789	1.1863665	0.105063	0.1679123
B2R5U1	cDNA, FLJ92620, highly similar to Homo sapiens staphylococcal nuclease domain containing 1 (SND1),mRNA OS=Homo sapiens PE=2 SV=1 - [B2R5U1_HUMAN]	20.68	2	16	24	885	99.60932	6.961426	1.19073	1.22673	1.1415	1.1863201	0.0171296	0.0575235
G3V1C3	Apoptosis inhibitor 5 OS=Homo sapiens GN=API5 PE=1 SV=1 - [G3V1C3_HUMAN]	7.25	3	3	4	510	57.52515	6.087402	1.2359	1.14065	1.18234	1.1862964	0.0212058	0.0632079
O00299	Chloride intracellular channel protein 1 OS=Homo sapiens GN=CLIC1 PE=1 SV=4 - [CLIC1_HUMAN]	56.02	8	8	17	241	26.90575	5.17334	1.17009	1.153	1.23571	1.1862681	0.0178262	0.058414
C9J3L8	Translocon-associated protein subunit alpha OS=Homo sapiens GN=SSR1 PE=1 SV=1 - [C9J3L8_HUMAN]	8.68	2	2	5	265	29.55479	4.297363	1.16352	1.23125	1.16185	1.185538	0.0148426	0.0540239
B4DJ66	Proteasome (Prosome, macropain) 26S subunit, non-ATPase, 13, isoform CRA_d OS=Homo sapiens GN=PSMD13 PE=2 SV=1 - [B4DJ66_HUMAN]	23.15	7	7	8	311	35.32438	6.01123	1.18955	1.20844	1.15847	1.1854866	0.00611207	0.0421458
Q8WUP2	Filamin-binding LIM protein 1 OS=Homo sapiens GN=FBLIM1 PE=1 SV=2 - [FBLI1_HUMAN]	17.43	6	6	7	373	40.64273	6.023926	1.15828	1.20156	1.19617	1.1853371	0.00535519	0.0401071
D6R9I9	ATP-binding cassette sub-family E member 1 OS=Homo sapiens GN=ABCE1 PE=1 SV=1 - [D6R9I9_HUMAN]	8.79	3	3	3	421	47.00639	8.309082	1.13612	1.20264	1.21671	1.185157	0.0175451	0.0580549
P61160	Actin-related protein 2 OS=Homo sapiens GN=ACTR2 PE=1 SV=1 - [ARP2_HUMAN]	25.13	9	9	15	394	44.73224	6.741699	1.13095	1.21489	1.20861	1.1848187	0.0206742	0.0626973
A6NLM8	Translocon-associated protein subunit delta OS=Homo sapiens GN=SSR4 PE=1 SV=1 - [A6NLM8_HUMAN]	20.27	2	2	3	148	16.23525	5.744629	1.23298	1.16245	1.15874	1.184725	0.0166682	0.0567917
Q5T7C4	High mobility group protein B1 OS=Homo sapiens GN=HMGB1 PE=1 SV=1 - [Q5T7C4_HUMAN]	52.53	7	8	16	158	18.29933	9.700684	1.15283	1.24405	1.15641	1.1844286	0.0251729	0.0696162

Biology Open • Supplementary information

O15247	Chloride intracellular channel protein 2 OS=Homo sapiens GN=CLIC2 PE=1 SV=3 - [CLIC2_HUMAN]	21.46	3	3	3	247	28.33845	5.592285	1.249	1.25345	1.05048	1.1843087	0.110443	0.1733192
B3KNN7	cDNA FLJ30049 fis, clone ADRGL1000033, highly similar to 26S proteasome non-ATPase regulatory subunit 3 OS=Homo sapiens PE=2 SV=1 - [B3KNN7_HUMAN]	11.24	5	5	7	498	57.19663	8.455566	1.25604	1.20853	1.08743	1.1839996	0.0670269	0.1226411
B3KW21	cDNA FLJ41945 fis, clone PLACE6019676, highly similar to Coatomer subunit gamma OS=Homo sapiens PE=2 SV=1 - [B3KW21_HUMAN]	19.67	8	10	14	610	67.7614	5.008301	1.10703	1.24696	1.19718	1.1837227	0.0462623	0.0979349
C9J931	GTP-binding protein Rheb OS=Homo sapiens GN=RHEB PE=1 SV=1 - [C9J931_HUMAN]	26.58	2	2	2	79	8.668373	6.023926	1.12064	1.27013	1.1573	1.182688	0.0556177	0.1093298
B4E324	cDNA FLJ60397, highly similar to Lysosomal protective protein (EC 3.4.16.5) OS=Homo sapiens PE=2 SV=1 - [B4E324_HUMAN]	6.25	2	3	3	480	54.23497	6.976074	1.11812	1.24446	1.1831	1.1818936	0.0379415	0.0871159
P61981	14-3-3 protein gamma OS=Homo sapiens GN=YWHAG PE=1 SV=2 - [1433G_HUMAN]	41.7	5	10	34	247	28.28491	4.894043	1.14195	1.2409	1.16233	1.1817242	0.026465	0.0717972
P22626	Heterogeneous nuclear ribonucleoproteins A2/B1 OS=Homo sapiens GN=HNRNPA2B1 PE=1 SV=2 - [ROA2_HUMAN]	40.79	12	17	39	353	37.40673	8.953613	1.19521	1.2252	1.12247	1.1809611	0.0272481	0.0732111
P09661	U2 small nuclear ribonucleoprotein A' OS=Homo sapiens GN=SNRPA1 PE=1 SV=2 - [RU2A_HUMAN]	7.84	2	2	2	255	28.39813	8.616699	1.21918	1.01139	1.31158	1.1807165	0.178704	0.2470041
P51991	Heterogeneous nuclear ribonucleoprotein A3 OS=Homo sapiens GN=HNRNPA3 PE=1 SV=2 - [ROA3_HUMAN]	23.81	6	8	14	378	39.57059	9.012207	1.10623	1.32278	1.10932	1.1794438	0.129313	0.1944436
P35241	Radixin OS=Homo sapiens GN=RDX PE=1 SV=1 - [RADI_HUMAN]	21.27	3	13	19	583	68.52139	6.366699	1.18592	1.23177	1.11798	1.1785586	0.0325983	0.0798446
B2RBI2	cDNA, FLJ95525, highly similar to Homo sapiens synapse associated protein 1, SAP47 homolog (Drosophila) (SYAP1), mRNA OS=Homo sapiens PE=2 SV=1 - [B2RBI2_HUMAN]	9.09	2	2	2	352	39.88083	4.525879	1.09617	1.18588	1.25349	1.1785133	0.0593991	0.1141772

Biology Open • Supplementary information

H3BPZ1	Very-long-chain (3R)-3-hydroxyacyl-CoA dehydratase 3 OS=Homo sapiens GN=HACD3 PE=1 SV=1 - [H3BPZ1_HUMAN]	7.42	2	2	2	337	40.04292	8.968262	1.20491	1.10591	1.22274	1.1778524	0.0392974	0.0887995
E9PKG1	Protein arginine N-methyltransferase 1 OS=Homo sapiens GN=PRMT1 PE=1 SV=1 - [E9PKG1_HUMAN]	27.69	7	7	7	325	37.68484	6.150879	1.13496	1.1724	1.22358	1.176979	0.0204217	0.0624988
P60981	Destrin OS=Homo sapiens GN=DSTN PE=1 SV=3 - [DEST_HUMAN]	32.12	6	7	17	165	18.49348	7.85498	1.14355	1.22211	1.1639	1.1765189	0.0173241	0.05785
B4DEI4	cDNA FLJ54595, highly similar to Golgi reassembly-stacking protein 2 OS=Homo sapiens PE=2 SV=1 - [B4DEI4_HUMAN]	8.64	3	3	3	405	42.01864	5.071777	1.18259	1.15546	1.19116	1.1764031	0.00370069	0.0383084
Q9NRZ7	1-acyl-sn-glycerol-3-phosphate acyltransferase gamma OS=Homo sapiens GN=AGPAT3 PE=1 SV=1 - [PLCC_HUMAN]	5.05	2	2	2	376	43.35273	8.719238	1.18906	1.17546	1.16283	1.1757859	0.00185081	0.0312267
P07602	Prosaposin OS=Homo sapiens GN=PSAP PE=1 SV=2 - [SAP_HUMAN]	17.94	8	8	26	524	58.07385	5.17334	1.20583	1.22346	1.09705	1.1754477	0.0471943	0.0991637
P28070	Proteasome subunit beta type-4 OS=Homo sapiens GN=PSMB4 PE=1 SV=4 - [PSB4_HUMAN]	20.45	4	4	6	264	29.18548	5.973145	1.20528	1.1717	1.14911	1.1753656	0.00854811	0.0457838
F5H7F6	Microsomal glutathione S-transferase 1 (Fragment) OS=Homo sapiens GN=MGST1 PE=1 SV=1 - [F5H7F6_HUMAN]	18.18	2	2	3	77	8.880476	8.79248	1.12748	1.23783	1.1607	1.1753374	0.0330382	0.0802733
H3BR35	Eukaryotic peptide chain release factor GTP-binding subunit ERF3A (Fragment) OS=Homo sapiens GN=GSPT1 PE=1 SV=1 - [H3BR35_HUMAN]	14.74	5	5	5	475	52.90871	5.630371	1.14215	1.23569	1.14689	1.1749111	0.0289438	0.0753733
P62826	GTP-binding nuclear protein Ran OS=Homo sapiens GN=RAN PE=1 SV=3 - [RAN_HUMAN]	35.65	8	8	20	216	24.40762	7.48877	1.15005	1.21668	1.15778	1.1748375	0.0141774	0.0528571
P00492	Hypoxanthine-guanine phosphoribosyltransferase OS=Homo sapiens GN=HPRT1 PE=1 SV=2 - [HPRT_HUMAN]	30.73	6	6	10	218	24.5636	6.683105	1.17558	1.21812	1.1281	1.1739335	0.021618	0.0637577

Biology Open • Supplementary information

Q9NRV9	Heme-binding protein 1 OS=Homo sapiens GN=HEBP1 PE=1 SV=1 - [HEBP1_HUMAN]	12.7	2	2	2	189	21.08352	5.79541	1.22317	1.13544	1.16227	1.1736275	0.021623	0.0637577
B2R774	cDNA, FLJ93313, highly similar to Homo sapiens lectin, mannose-binding, 1 (LMAN1), mRNA OS=Homo sapiens PE=2 SV=1 - [B2R774_HUMAN]	14.31	5	5	7	510	57.48297	6.770996	1.11868	1.14996	1.25202	1.1735511	0.0498226	0.101903
A0A087X2G1	ATP-dependent RNA helicase DDX1 OS=Homo sapiens GN=DDX1 PE=1 SV=1 - [A0A087X2G1_HUMAN]	17.3	8	8	12	659	73.92839	7.752441	1.11597	1.20095	1.19925	1.1720564	0.025558	0.0704204
B4DZZ0	cDNA FLJ52128, highly similar to PRA1 family protein 3 OS=Homo sapiens PE=2 SV=1 - [B4DZZ0_HUMAN]	10.91	2	2	3	165	19.19308	9.773926	1.07644	1.23938	1.19964	1.1718195	0.0727146	0.1297947
D3DV75	Adenosine deaminase, RNA-specific, isoform CRA_b OS=Homo sapiens GN=ADAR PE=4 SV=1 - [D3DV75_HUMAN]	3.95	4	4	4	886	98.68289	8.660645	1.15918	1.13464	1.22105	1.1716222	0.0217112	0.0639065
Q59FI5	Protein kinase C, alpha variant (Fragment) OS=Homo sapiens PE=2 SV=1 - [Q59FI5_HUMAN]	4.56	2	2	2	461	52.94142	7.10791	1.18848	1.18003	1.14618	1.1715641	0.00562918	0.0408901
A0A0D9SEN1	Prolyl endopeptidase FAP OS=Homo sapiens GN=FAP PE=1 SV=1 - [A0A0D9SEN1_HUMAN]	2.5	2	2	2	759	87.52858	6.55127	1.16416	1.19485	1.15459	1.171197	0.00499472	0.0401071
B1AHC9	X-ray repair cross-complementing protein 6 OS=Homo sapiens GN=XRCC6 PE=1 SV=1 - [B1AHC9_HUMAN]	34.88	19	19	29	559	64.24301	9.275879	1.14661	1.21712	1.14948	1.1710679	0.0176602	0.058223
B4DFP1	cDNA FLJ51818, highly similar to Phosphoglucomutase-1 (EC 5.4.2.2) OS=Homo sapiens PE=2 SV=1 - [B4DFP1_HUMAN]	20.45	9	9	13	538	58.702	6.049316	1.17602	1.19076	1.14549	1.1707556	0.00603891	0.0421458
Q5M7Z9	TARS protein (Fragment) OS=Homo sapiens GN=TARS PE=2 SV=1 - [Q5M7Z9_HUMAN]	9.82	5	5	6	682	78.55544	6.888184	1.19315	1.23121	1.0879	1.1707523	0.0576118	0.1121076
H3BMQ1	Prefoldin subunit 5 OS=Homo sapiens GN=PFDN5 PE=1 SV=1 - [H3BMQ1_HUMAN]	27.38	2	2	2	84	9.528925	8.455566	1.24309	1.05741	1.21036	1.1702863	0.0968107	0.1586945
P08311	Cathepsin G OS=Homo sapiens GN=CTSG PE=1 SV=2 - [CATG_HUMAN]	38.82	8	8	20	255	28.81907	11.19482	1.13159	1.18535	1.19379	1.1702476	0.01284	0.0510733

Biology Open • Supplementary information

Q6QN92	Mitochondrial glycine cleavage system H-protein (Fragment) OS=Homo sapiens PE=2 SV=1 - [Q6QN92_HUMAN]	24	2	2	2	125	13.80473	4.335449	1.15735	1.22873	1.12404	1.17004	0.0314321	0.0782911
Q08945	FACT complex subunit SSRP1 OS=Homo sapiens GN=SSRP1 PE=1 SV=1 - [SSRP1_HUMAN]	5.78	4	4	5	709	81.02442	6.873535	1.17536	1.1436	1.18928	1.1694123	0.00630699	0.0423147
J3KPM9	Signal transducer and activator of transcription OS=Homo sapiens GN=STAT1 PE=1 SV=1 - [J3KPM9_HUMAN]	14.43	9	9	11	714	83.30763	6.41748	1.22738	1.14653	1.13277	1.1688944	0.0291983	0.0756786
H0YGG4	Translation initiation factor eIF-2B subunit alpha (Fragment) OS=Homo sapiens GN=EIF2B1 PE=1 SV=1 - [H0YGG4_HUMAN]	11.18	2	2	2	152	16.91546	6.902832	1.13403	1.22961	1.14255	1.1687296	0.0312317	0.0779641
H7BZH6	Quinone oxidoreductase PIG3 (Fragment) OS=Homo sapiens GN=TP53I3 PE=1 SV=1 - [H7BZH6_HUMAN]	11.57	2	2	3	216	22.63983	6.536621	1.19828	1.22289	1.08412	1.1684281	0.0587973	0.1134068
B4DV28	cDNA FLJ54170, highly similar to Cytosolic nonspecific dipeptidase OS=Homo sapiens PE=2 SV=1 - [B4DV28_HUMAN]	27.65	10	10	14	463	51.46932	6.430176	1.16715	1.21356	1.12401	1.1682399	0.0228123	0.0654404
P25787	Proteasome subunit alpha type-2 OS=Homo sapiens GN=PSMA2 PE=1 SV=2 - [PSA2_HUMAN]	21.37	4	4	7	234	25.88227	7.430176	1.13137	1.14646	1.22572	1.1678512	0.0290727	0.0755846
B4E266	cDNA FLJ58466, highly similar to Leucyl-tRNA synthetase, cytoplasmic (EC 6.1.1.4) OS=Homo sapiens PE=2 SV=1 - [B4E266_HUMAN]	4.25	4	4	6	1130	129.1049	7.781738	1.07762	1.1897	1.2355	1.1676031	0.0701495	0.1267108
G3V2V8	Epididymal secretory protein E1 (Fragment) OS=Homo sapiens GN=NPC2 PE=1 SV=1 - [G3V2V8_HUMAN]	20.49	2	2	2	122	13.06967	8.338379	1.15657	1.19631	1.14986	1.1675784	0.00739937	0.0445366
Q2Q9H2	Glucose-6-phosphate 1-dehydrogenase (Fragment) OS=Homo sapiens GN=G6PD PE=3 SV=1 - [Q2Q9H2_HUMAN]	26.11	10	10	12	475	54.78982	7.078613	1.17485	1.209	1.11776	1.1672048	0.0244138	0.0682981
P57105	Synaptojanin-2-binding protein OS=Homo sapiens GN=SYNJ2BP PE=1 SV=2 - [SYJ2B_HUMAN]	17.93	2	2	3	145	15.91816	6.303223	1.04113	1.23363	1.22551	1.1667568	0.117558	0.181871

Biology Open • Supplementary information

Q14166	Tubulin--tyrosine ligase-like protein 12 OS=Homo sapiens GN=TTLL12 PE=1 SV=2 - [TTL12_HUMAN]	5.75	3	3	3	644	74.35604	5.528809	1.26528	1.06185	1.17297	1.166701	0.105181	0.1679998
P50570	Dynamin-2 OS=Homo sapiens GN=DNM2 PE=1 SV=2 - [DYN2_HUMAN]	6.78	3	5	6	870	98.00317	7.444824	1.12163	1.19823	1.17954	1.166469	0.0186501	0.0598394
P61457	Pterin-4-alpha-carbinolamine dehydratase OS=Homo sapiens GN=PCBD1 PE=1 SV=2 - [PHS_HUMAN]	20.19	2	2	2	104	11.99202	6.800293	1.18339	1.16072	1.15512	1.1664104	0.00268764	0.0357839
E7EX73	Eukaryotic translation initiation factor 4 gamma 1 OS=Homo sapiens GN=EIF4G1 PE=1 SV=1 - [E7EX73_HUMAN]	3.97	5	5	7	1436	158.547	5.211426	1.25532	1.20933	1.03446	1.1663692	0.131933	0.1970844
Q06210	Glutamine--fructose-6-phosphate aminotransferase [isomerizing] 1 OS=Homo sapiens GN=GFPT1 PE=1 SV=3 - [GFPT1_HUMAN]	11.87	7	7	7	699	78.75633	7.10791	1.15094	1.10147	1.24653	1.1663135	0.0597163	0.1143971
H6VRF8	Keratin 1 OS=Homo sapiens GN=KRT1 PE=3 SV=1 - [H6VRF8_HUMAN]	46.74	26	28	79	644	66.01302	8.118652	1.17358	1.19147	1.13383	1.1662939	0.01033	0.0487686
Q9BPW0	Serine/threonine-protein phosphatase (Fragment) OS=Homo sapiens GN=PPP5C PE=2 SV=2 - [Q9BPW0_HUMAN]	14.26	5	5	5	484	55.06136	6.20166	1.12365	1.1781	1.19621	1.1659867	0.0168258	0.0569966
P08727	Keratin, type I cytoskeletal 19 OS=Homo sapiens GN=KRT19 PE=1 SV=4 - [K1C19_HUMAN]	35.25	8	15	20	400	44.07912	5.135254	1.27877	1.13528	1.08359	1.1658819	0.104766	0.1675167
Q05CP8	CCDC6 protein (Fragment) OS=Homo sapiens GN=CCDC6 PE=2 SV=1 - [Q05CP8_HUMAN]	9.88	2	2	2	334	38.13992	9.393066	1.16505	1.16717	1.16371	1.1653105	3.71E-05	0.0173763
A0A024R0K5	Carcinoembryonic antigen-related cell adhesion molecule 5 OS=Homo sapiens GN=CEACAM5 PE=1 SV=1 - [A0A024R0K5_HUMAN]	9.83	3	4	5	702	76.74818	5.693848	1.21305	1.09317	1.18742	1.1645468	0.0457228	0.0971132
B4DUC5	cDNA FLJ53202, highly similar to Exportin-2 OS=Homo sapiens PE=2 SV=1 - [B4DUC5_HUMAN]	10.88	6	6	7	754	85.4181	5.478027	1.13435	1.13562	1.22122	1.1637266	0.0294719	0.0760964
C9JS50	Metalloreductase STEAP4 OS=Homo sapiens GN=STEAP4 PE=1 SV=1 - [C9JS50_HUMAN]	11.5	2	2	4	339	38.55423	8.880371	1.06157	1.19643	1.23266	1.1635545	0.088112	0.1489806

B3KX19	cDNA FLJ44500 fis, clone UTERU3000828, highly similar to 116 kDa U5 small nuclear ribonucleoprotein component OS=Homo sapiens PE=2 SV=1 - [B3KX19_HUMAN]	10.81	7	8	8	962	108.1417	4.995605	1.1125	1.16392	1.21396	1.1634592	0.0306388	0.0773154
P62266	40S ribosomal protein S23 OS=Homo sapiens GN=RPS23 PE=1 SV=3 - [RS23_HUMAN]	23.08	3	3	6	143	15.79772	10.4917	1.14588	1.2233	1.12118	1.1634542	0.0336448	0.0810225
Q53GR7	Solute carrier family 25, member 13 (Citrin) variant (Fragment) OS=Homo sapiens PE=2 SV=1 - [Q53GR7_HUMAN]	13.19	5	7	10	675	74.06956	8.382324	1.10323	1.27492	1.10997	1.162711	0.101281	0.1635669
D6RGG3	Collagen alpha-1(XII) chain OS=Homo sapiens GN=COL12A1 PE=1 SV=1 - [D6RGG3_HUMAN]	18.29	48	48	74	3062	332.9957	5.528809	1.14997	1.1799	1.15705	1.1623069	0.00308051	0.0360868
H0YEY4	ADP-sugar pyrophosphatase (Fragment) OS=Homo sapiens GN=NUDT5 PE=1 SV=1 - [H0YEY4_HUMAN]	18.25	2	2	2	137	15.00122	4.703613	1.18165	1.20333	1.1016	1.1621946	0.0345111	0.081979
A0A0G2JR65	Mucin-2 OS=Homo sapiens GN=MUC2 PE=1 SV=1 - [A0A0G2JR65_HUMAN]	3.87	7	7	7	2817	303.1841	5.884277	1.22086	1.10091	1.16473	1.1621655	0.0427536	0.0935455
Q04837	Single-stranded DNA-binding protein, mitochondrial OS=Homo sapiens GN=SSBP1 PE=1 SV=1 - [SSBP_HUMAN]	38.51	5	5	6	148	17.24903	9.598145	1.13979	1.20572	1.13998	1.1618309	0.017895	0.0585828
B1ANR0	Polyadenylate-binding protein OS=Homo sapiens GN=PABPC4 PE=1 SV=1 - [B1ANR0_HUMAN]	9.76	3	5	5	615	67.92792	9.45166	1.24418	1.19059	1.04916	1.1613095	0.109159	0.1722459
Q9BR76	Coronin-1B OS=Homo sapiens GN=CORO1B PE=1 SV=1 - [COR1B_HUMAN]	7.16	4	4	5	489	54.20025	5.884277	1.12042	1.15185	1.21137	1.1612146	0.026287	0.0715861
Q15717	ELAV-like protein 1 OS=Homo sapiens GN=ELAVL1 PE=1 SV=2 - [ELAV1_HUMAN]	12.88	4	4	4	326	36.06915	9.17334	1.15227	1.1997	1.13122	1.1610627	0.0154456	0.0546326
Q9Y3Z3	Deoxynucleoside triphosphate triphosphohydrolase SAMHD1 OS=Homo sapiens GN=SAMHD1 PE=1 SV=2 - [SAMH1_HUMAN]	38.02	21	21	31	626	72.15478	7.137207	1.14974	1.15418	1.17913	1.1610188	0.00321254	0.0364914

Biology Open • Supplementary information

A0A024R936	Neutrophil cytosolic factor 2 (65kDa, chronic granulomatous disease, autosomal 2), isoform CRA_a OS=Homo sapiens GN=NCF2 PE=4 SV=1 - [A0A024R936_HUMAN]	4.75	2	2	3	526	59.7515	6.163574	1.19048	1.09212	1.20019	1.1609279	0.0430562	0.0938359
P50395	Rab GDP dissociation inhibitor beta OS=Homo sapiens GN=GDI2 PE=1 SV=2 - [GDIB_HUMAN]	43.15	12	16	31	445	50.63088	6.468262	1.13833	1.19199	1.15243	1.1609178	0.00981416	0.0478721
A0A024RBE8	Solute carrier family 25 (Mitochondrial carrier phosphate carrier), member 3, isoform CRA_a OS=Homo sapiens GN=SLC25A3 PE=3 SV=1 - [A0A024RBE8_HUMAN]	13.85	5	5	9	361	39.93264	9.36377	1.14673	1.29429	1.04003	1.1603536	0.161598	0.2288742
P16615	Sarcoplasmic/endoplasmic reticulum calcium ATPase 2 OS=Homo sapiens GN=ATP2A2 PE=1 SV=1 - [AT2A2_HUMAN]	18.14	13	16	23	1042	114.6826	5.338379	1.18562	1.21554	1.07833	1.1598291	0.0616969	0.1162829
P50402	Emerin OS=Homo sapiens GN=EMD PE=1 SV=1 - [EMD_HUMAN]	17.72	4	4	4	254	28.97593	5.503418	1.12197	1.19132	1.16326	1.158851	0.015702	0.055022
P62805	Histone H4 OS=Homo sapiens GN=HIST1H4A PE=1 SV=2 - [H4_HUMAN]	60.19	10	10	96	103	11.36038	11.35596	1.14747	1.18478	1.14142	1.1578888	0.00729268	0.0440167
O75348	V-type proton ATPase subunit G 1 OS=Homo sapiens GN=ATP6V1G1 PE=1 SV=3 - [VATG1_HUMAN]	16.95	2	2	2	118	13.74908	8.79248	1.15033	1.21678	1.1032	1.15677	0.0414383	0.0920252
B4DJE7	cDNA FLJ52595, highly similar to Medium-chain specific acyl-CoA dehydrogenase, mitochondrial (EC 1.3.99.3) OS=Homo sapiens PE=2 SV=1 - [B4DJE7_HUMAN]	17.24	3	3	3	232	25.64719	8.997559	1.13096	1.18666	1.15191	1.1565104	0.0106016	0.0490917
K7ENX8	Cold-inducible RNA-binding protein (Fragment) OS=Homo sapiens GN=CIRBP PE=1 SV=2 - [K7ENX8_HUMAN]	31.25	2	2	2	80	8.908327	4.805176	1.14705	1.18283	1.13925	1.1563748	0.00728103	0.0440167
P29144	Tripeptidyl-peptidase 2 OS=Homo sapiens GN=TPP2 PE=1 SV=4 - [TPP2_HUMAN]	2.72	2	2	2	1249	138.2626	6.315918	1.15213	1.13133	1.18546	1.1563063	0.0100172	0.0483023
Q9BTQ7	Similar to ribosomal protein L23 (Fragment) OS=Homo sapiens PE=2 SV=1 - [Q9BTQ7_HUMAN]	45.52	5	5	9	134	14.14067	10.25732	1.16647	1.15522	1.14675	1.1561495	0.00133505	0.0283421

Biology Open • Supplementary information

P37837	Transaldolase OS=Homo sapiens GN=TALDO1 PE=1 SV=2 - [TALDO_HUMAN]	25.22	9	9	13	337	37.51646	6.814941	1.16708	1.21304	1.088	1.1560372	0.0506368	0.1028322
Q53HV2	Chaperonin containing TCP1, subunit 7 (Eta) variant (Fragment) OS=Homo sapiens PE=2 SV=1 - [Q53HV2_HUMAN]	31.49	14	14	20	543	59.30288	7.649902	1.11116	1.21182	1.14412	1.1556987	0.0343562	0.0817258
B2R6J2	cDNA, FLJ92973, highly similar to Homo sapiens villin 2 (ezrin) (VIL2), mRNA OS=Homo sapiens PE=2 SV=1 - [B2R6J2_HUMAN]	21.33	5	13	20	586	69.3577	6.265137	1.14144	1.19363	1.13128	1.1554485	0.0150917	0.0542884
Q6PID9	Aggrecan OS=Homo sapiens GN=ACAN PE=1 SV=1 - [Q6PID9_HUMAN]	14.98	9	9	11	721	79.56505	5.059082	1.1396	1.14772	1.17793	1.1550845	0.00560786	0.0408901
A0A087X0K0	Collagen alpha-1(XV) chain OS=Homo sapiens GN=COL15A1 PE=1 SV=1 - [A0A087X0K0_HUMAN]	8.59	9	9	13	1374	139.972	4.98291	1.07562	1.23874	1.1497	1.1546859	0.0816962	0.1412301
B4DSD7	cDNA FLJ55863, highly similar to Serine/threonine-protein phosphatase 2A 56 kDa regulatory subunit delta isoform OS=Homo sapiens PE=2 SV=1 - [B4DSD7_HUMAN]	3.76	2	2	2	559	65.36202	7.825684	1.05726	1.14564	1.26018	1.1543601	0.119418	0.1839158
A0A087WTT1	Polyadenylate-binding protein OS=Homo sapiens GN=PABPC1 PE=1 SV=1 - [A0A087WTT1_HUMAN]	31.03	13	15	19	522	58.49884	9.26123	1.12174	1.22696	1.11359	1.1540984	0.0518072	0.1043663
Q9Y2H1	Serine/threonine-protein kinase 38-like OS=Homo sapiens GN=STK38L PE=1 SV=3 - [ST38L_HUMAN]	4.96	2	2	2	464	53.96841	6.814941	1.13706	1.22928	1.09595	1.1540981	0.0596511	0.1143971
Q9BW30	Tubulin polymerization-promoting protein family member 3 OS=Homo sapiens GN=TPPP3 PE=1 SV=1 - [TPPP3_HUMAN]	17.05	3	3	6	176	18.97364	9.129395	1.15423	1.19792	1.10895	1.1537012	0.0268091	0.0724252
B1AJQ6	Syntaxin-12 (Fragment) OS=Homo sapiens GN=STX12 PE=1 SV=2 - [B1AJQ6_HUMAN]	13.95	2	2	3	215	24.56134	5.147949	1.22783	0.998019	1.23393	1.1532599	0.187092	0.2560883
A0A0D9SFK2	Unconventional myosin-XVIIIa OS=Homo sapiens GN=MYO18A PE=1 SV=1 - [A0A0D9SFK2_HUMAN]	2.16	3	3	4	2038	230.964	6.252441	1.18436	1.14847	1.12621	1.1530125	0.0120359	0.0496848

Biology Open • Supplementary information

C9JGI3	Thymidine phosphorylase (Fragment) OS=Homo sapiens GN=TYMP PE=1 SV=1 - [C9JGI3_HUMAN]	28.31	10	10	17	445	46.0582	5.516113	1.24645	1.12221	1.0891	1.1525881	0.086009	0.1464109
B4DWN1	cDNA FLJ52285, highly similar to Vesicular integral-membrane protein VIP36 OS=Homo sapiens PE=2 SV=1 - [B4DWN1_HUMAN]	24.56	6	6	7	285	32.55712	6.536621	1.13056	1.16618	1.15975	1.1521622	0.00514945	0.0401071
Q7L576	Cytoplasmic FMR1-interacting protein 1 OS=Homo sapiens GN=CYFIP1 PE=1 SV=1 - [CYFP1_HUMAN]	9.42	4	10	13	1253	145.0887	6.902832	1.163	1.10937	1.18406	1.1521447	0.0206961	0.0626973
Q13630	GDP-L-fucose synthase OS=Homo sapiens GN=TSTA3 PE=1 SV=1 - [FCL_HUMAN]	10.28	3	3	4	321	35.86996	6.595215	1.1753	1.17173	1.1093	1.1521079	0.0192767	0.0608852
P61224	Ras-related protein Rap-1b OS=Homo sapiens GN=RAP1B PE=1 SV=1 - [RAP1B_HUMAN]	47.28	3	9	14	184	20.81159	5.782715	1.19841	1.17386	1.0807	1.1509913	0.0520161	0.1046816
A8K0P2	cDNA FLJ76209, highly similar to Homo sapiens inorganic pyrophosphatase 2 (PPA2), transcript variant 2, mRNA OS=Homo sapiens PE=2 SV=1 - [A8K0P2_HUMAN]	15.33	3	4	9	274	31.55588	7.430176	1.1211	1.16427	1.16654	1.1506373	0.00949537	0.0472949
B2R8R5	cDNA, FLJ94025, highly similar to Homo sapiens tripartite motif-containing 28 (TRIM28), mRNA OS=Homo sapiens PE=2 SV=1 - [B2R8R5_HUMAN]	15.81	10	10	13	835	88.50344	5.77002	1.16925	1.18498	1.09765	1.1506278	0.0303862	0.0772202
B4DFL1	Dihydrolipoyl dehydrogenase OS=Homo sapiens PE=2 SV=1 - [B4DFL1_HUMAN]	19.17	7	7	10	459	48.86829	7.48877	1.15215	1.13852	1.16113	1.1505994	0.00189917	0.0313595
Q13347	Eukaryotic translation initiation factor 3 subunit I OS=Homo sapiens GN=EIF3I PE=1 SV=1 - [EIF3I_HUMAN]	11.69	4	4	4	325	36.47862	5.643066	1.17862	1.13076	1.14199	1.1504574	0.00909988	0.0466008
Q9UHS8	PRO1975 OS=Homo sapiens PE=2 SV=1 - [Q9UHS8_HUMAN]	40.2	14	14	20	393	44.1187	9.026855	1.12806	1.15473	1.16853	1.1504376	0.00617643	0.0423147
Q59GU6	Sorting nexin 1 isoform a variant (Fragment) OS=Homo sapiens PE=2 SV=1 - [Q59GU6_HUMAN]	17.82	5	7	11	432	50.0679	6.976074	1.10194	1.13763	1.21071	1.1500938	0.0426032	0.093462
O60664	Perilipin-3 OS=Homo sapiens GN=PLIN3 PE=1 SV=3 -	29.72	8	8	11	434	47.04595	5.439941	1.15209	1.21269	1.08462	1.1498006	0.0559061	0.1098329

Biology Open • Supplementary information

	[PLIN3_HUMAN]													
P15121	Aldose reductase OS=Homo sapiens GN=AKR1B1 PE=1 SV=3 - [ALDR_HUMAN]	8.86	2	3	4	316	35.8305	6.976074	1.14112	1.19732	1.11046	1.1496346	0.0276924	0.0736361
E9PCY7	Heterogeneous nuclear ribonucleoprotein H OS=Homo sapiens GN=HNRNPH1 PE=1 SV=1 - [E9PCY7_HUMAN]	21.68	6	8	17	429	47.05745	6.341309	1.12527	1.16626	1.1571	1.1495433	0.00682753	0.0433606
Q68E00	Putative uncharacterized protein DKFZp686G2045 (Fragment) OS=Homo sapiens GN=DKFZp686G2045 PE=4 SV=2 - [Q68E00_HUMAN]	11.43	2	2	2	315	32.55928	6.770996	1.12071	1.1606	1.1669	1.1494025	0.00923903	0.0469581
B4DNP0	Signal transducer and activator of transcription OS=Homo sapiens PE=2 SV=1 - [B4DNP0_HUMAN]	8.93	5	5	5	672	76.11339	6.303223	1.1396	1.18134	1.12714	1.1493591	0.01183	0.0495204
Q92626	Peroxidasin homolog OS=Homo sapiens GN=PXDN PE=1 SV=2 - [PXDN_HUMAN]	5.61	6	6	6	1479	165.1699	7.166504	1.14783	1.08905	1.21064	1.1491735	0.0511702	0.1035952
P49006	MARCKS-related protein OS=Homo sapiens GN=MARCKSL1 PE=1 SV=2 - [MRP_HUMAN]	14.36	2	2	2	195	19.51723	4.665527	1.18218	1.14144	1.12213	1.1485832	0.0138958	0.0525556
H0YMZ1	Proteasome subunit alpha type (Fragment) OS=Homo sapiens GN=PSMA4 PE=1 SV=6 - [H0YMZ1_HUMAN]	25.45	5	5	7	220	24.51042	6.770996	1.19876	1.16933	1.07556	1.1478819	0.0576932	0.1121076
A8K2Q6	Peptidyl-prolyl cis-trans isomerase OS=Homo sapiens PE=2 SV=1 - [A8K2Q6_HUMAN]	12.74	2	3	3	212	22.70286	8.396973	1.09922	1.20848	1.13553	1.1477432	0.0441797	0.0952537
K7ES95	Protein LZIC (Fragment) OS=Homo sapiens GN=LZIC PE=1 SV=1 - [K7ES95_HUMAN]	17.75	2	2	2	169	19.05793	5.160645	1.09729	1.22325	1.12161	1.1473847	0.0621875	0.1168729
Q8IVN8	Somatomedin-B and thrombospondin type-1 domain-containing protein OS=Homo sapiens GN=SBSPON PE=1 SV=2 - [SBSPO_HUMAN]	14.77	3	3	4	264	29.59052	7.503418	1.16467	1.15256	1.12414	1.1471224	0.00659759	0.043197
A4FU77	SNRNP200 protein (Fragment) OS=Homo sapiens GN=SNRNP200 PE=2 SV=1 - [A4FU77_HUMAN]	4.08	6	6	7	1887	216.0455	6.480957	1.10575	1.15309	1.18116	1.1466661	0.0217793	0.0640512
Q7Z759	CCT8 protein OS=Homo sapiens GN=CCT8 PE=2 SV=1 -	44.27	18	18	23	497	54.07173	5.312988	1.14884	1.17923	1.11185	1.1466407	0.017196	0.0576824

Biology Open • Supplementary information

	[Q7Z759_HUMAN]														
B2RBH2	cDNA, FLJ95508, highly similar to Homo sapiens 5'-nucleotidase, ecto (CD73) (NT5E), mRNA OS=Homo sapiens PE=2 SV=1 - [B2RBH2_HUMAN]	11.15	5	5	7	574	63.35549	7.034668	1.09437	1.17956	1.16546	1.1464629	0.0309003	0.0773918
P21399	Cytoplasmic aconitate hydratase OS=Homo sapiens GN=ACO1 PE=1 SV=3 - [ACOC_HUMAN]	6.86	6	6	8	889	98.33659	6.683105	1.13428	1.20052	1.10387	1.1462242	0.0360311	0.0844635
H0YAL7	Eukaryotic translation elongation factor 1 epsilon-1 (Fragment) OS=Homo sapiens GN=EEF1E1 PE=4 SV=1 - [H0YAL7_HUMAN]	13.97	2	2	2	136	15.32889	8.396973	1.12468	1.14691	1.16406	1.1452186	0.00610554	0.0421458
A8K690	cDNA FLJ76863, highly similar to Homo sapiens stress-induced-phosphoprotein 1 (Hsp70/Hsp90-organizing protein) (STIP1), mRNA OS=Homo sapiens PE=2 SV=1 - [A8K690_HUMAN]	27.99	13	13	19	543	62.61544	6.800293	1.13546	1.17094	1.1284	1.1449349	0.00814407	0.0456419
P60903	Protein S100-A10 OS=Homo sapiens GN=S100A10 PE=1 SV=2 - [S10AA_HUMAN]	45.36	5	5	11	97	11.19551	7.371582	1.15453	1.17238	1.10751	1.1448093	0.0173868	0.05785
P12814	Alpha-actinin-1 OS=Homo sapiens GN=ACTN1 PE=1 SV=2 - [ACTN1_HUMAN]	55.38	6	47	148	892	102.9926	5.414551	1.11491	1.13893	1.17965	1.1444958	0.0166768	0.0567917
Q14108	Lysosome membrane protein 2 OS=Homo sapiens GN=SCARB2 PE=1 SV=2 - [SCRB2_HUMAN]	8.58	4	4	7	478	54.25546	5.135254	1.22274	1.10352	1.10712	1.1444602	0.0662465	0.1217336
Q15181	Inorganic pyrophosphatase OS=Homo sapiens GN=PPA1 PE=1 SV=2 - [IPYR_HUMAN]	33.22	5	6	11	289	32.63916	5.858887	1.22455	1.09903	1.10941	1.1443319	0.0696466	0.1260715
A5Z217	Mutant desmin OS=Homo sapiens PE=2 SV=1 - [A5Z217_HUMAN]	68.09	25	30	97	470	53.56319	5.274902	1.10856	1.20547	1.11842	1.1441521	0.042726	0.0935455
Q15942	Zyxin OS=Homo sapiens GN=ZYX PE=1 SV=1 - [ZYX_HUMAN]	25.52	11	11	21	572	61.23817	6.668457	1.16896	1.17497	1.08555	1.1431584	0.0383056	0.0876111

Biology Open • Supplementary information

B4DQK1	cDNA FLJ53475, highly similar to Autophagy-related protein 7 OS=Homo sapiens PE=2 SV=1 - [B4DQK1_HUMAN]	4.85	3	3	3	681	75.45795	6.239746	1.17593	1.21616	1.03559	1.1425608	0.121174	0.1857672
Q05CK9	SYNCRIP protein (Fragment) OS=Homo sapiens GN=SYNCRIP PE=2 SV=1 - [Q05CK9_HUMAN]	37.09	11	13	19	453	50.61894	6.712402	1.18189	1.17122	1.0739	1.1423368	0.0536191	0.1068908
E1CKY7	Protein phosphatase 1 regulatory subunit 12B OS=Homo sapiens GN=sm-M20 PE=1 SV=1 - [E1CKY7_HUMAN]	23.12	4	4	4	186	21.35408	9.686035	1.07738	1.09327	1.25451	1.1417199	0.129217	0.1944436
H0Y4R1	Inosine-5'-monophosphate dehydrogenase 2 (Fragment) OS=Homo sapiens GN=IMPDH2 PE=1 SV=1 - [H0Y4R1_HUMAN]	17.23	6	6	7	470	51.03521	8.177246	1.04982	1.19038	1.18484	1.1416838	0.0910699	0.1520827
P80511	Protein S100-A12 OS=Homo sapiens GN=S100A12 PE=1 SV=2 - [S10AC_HUMAN]	28.26	2	2	2	92	10.56853	6.252441	1.12262	1.15837	1.14263	1.1412093	0.00532348	0.0401071
B4DE96	cDNA FLJ53762, highly similar to Synaptobrevin-like protein 1 OS=Homo sapiens PE=2 SV=1 - [B4DE96_HUMAN]	11.11	2	2	2	153	17.36813	9.290527	1.11068	1.17225	1.13993	1.1409544	0.0155434	0.0547825
Q68E05	Putative uncharacterized protein DKFZp686A1765 OS=Homo sapiens GN=DKFZp686A1765 PE=2 SV=1 - [Q68E05_HUMAN]	37.63	5	5	8	186	19.8891	5.478027	1.22527	1.22229	0.973827	1.1404631	0.233883	0.3030988
P07437	Tubulin beta chain OS=Homo sapiens GN=TUBB PE=1 SV=2 - [TBB5_HUMAN]	56.08	5	18	95	444	49.63897	4.894043	1.12143	1.21503	1.08308	1.1398445	0.0703363	0.1269805
Q9NTZ6	RNA-binding protein 12 OS=Homo sapiens GN=RBM12 PE=1 SV=1 - [RBM12_HUMAN]	3.76	4	4	4	932	97.33341	8.631348	1.04388	1.25013	1.12292	1.1389785	0.146806	0.2128216
D3DSM4	Collagen, type XVIII, alpha 1, isoform CRA_d OS=Homo sapiens GN=COL18A1 PE=4 SV=1 - [D3DSM4_HUMAN]	11.23	14	14	20	1336	135.426	6.468262	1.12199	1.17346	1.12088	1.1387739	0.0152636	0.0543533
P05198	Eukaryotic translation initiation factor 2 subunit 1 OS=Homo sapiens GN=EIF2S1 PE=1 SV=3 - [IF2A_HUMAN]	15.24	4	4	5	315	36.08938	5.084473	1.1745	1.09633	1.14531	1.138713	0.0259819	0.0711559
J3KR24	Isoleucine--tRNA ligase, cytoplasmic OS=Homo sapiens GN=IARS PE=1 SV=1 - [J3KR24_HUMAN]	9.2	8	8	8	1152	131.6797	6.112793	1.10048	1.14491	1.16996	1.1384484	0.0208597	0.0628695

Biology Open • Supplementary information

Q53SY7	Putative uncharacterized protein CAD (Fragment) OS=Homo sapiens GN=CAD PE=3 SV=1 - [Q53SY7_HUMAN]	3.3	6	6	6	2151	235.0286	6.595215	1.07376	1.17744	1.16286	1.1380196	0.0509534	0.1032798
A0A087WXM6	60S ribosomal protein L17 (Fragment) OS=Homo sapiens GN=RPL17 PE=3 SV=1 - [A0A087WXM6_HUMAN]	33.14	4	5	10	169	19.57324	10.0376	1.11251	1.17381	1.12656	1.1376271	0.0176647	0.058223
P07737	Profilin-1 OS=Homo sapiens GN=PFN1 PE=1 SV=2 - [PROF1_HUMAN]	63.57	10	10	43	140	15.04456	8.265137	1.1382	1.14275	1.13171	1.1375506	0.000542075	0.0241782
A0A0A0MTR7	E3 ubiquitin-protein ligase RNF213 OS=Homo sapiens GN=RNF213 PE=1 SV=1 - [A0A0A0MTR7_HUMAN]	0.6	3	3	3	5207	591.0313	6.480957	1.18145	1.08969	1.14094	1.1373595	0.0353861	0.0833458
Q05D78	MRE11A protein (Fragment) OS=Homo sapiens GN=MRE11A PE=2 SV=1 - [Q05D78_HUMAN]	6	3	3	3	517	59.68676	6.91748	1.01881	1.23171	1.16111	1.1372093	0.159756	0.2271206
H0Y614	Ubiquitin-fold modifier 1 (Fragment) OS=Homo sapiens GN=UFM1 PE=1 SV=1 - [H0Y614_HUMAN]	27.16	2	2	3	81	8.655606	9.466309	1.18198	1.16384	1.06509	1.1369714	0.0636659	0.1187378
Q15847	Adipogenesis regulatory factor OS=Homo sapiens GN=ADIRF PE=1 SV=1 - [ADIRF_HUMAN]	28.95	2	2	3	76	7.849975	5.312988	1.10956	1.24114	1.05945	1.1367147	0.127662	0.1929178
A8K646	cDNA FLJ75699, highly similar to Homo sapiens osteoclast stimulating factor 1 (OSTF1), mRNA OS=Homo sapiens PE=2 SV=1 - [A8K646_HUMAN]	14.49	2	2	3	214	23.69986	5.681152	1.12368	1.25881	1.02745	1.1366434	0.178644	0.2470041
M1VK07	HLA-DRB1 protein (Fragment) OS=Homo sapiens GN=HLA-DRB1 PE=4 SV=1 - [M1VK07_HUMAN]	23.45	2	4	4	226	25.64981	7.532715	1.1665	1.1002	1.14199	1.1362294	0.01959	0.0612255
B4DT77	Annexin OS=Homo sapiens PE=2 SV=1 - [B4DT77_HUMAN]	25.6	8	8	12	336	37.78092	7.181152	1.12079	1.14257	1.14389	1.1357507	0.00302961	0.0360868
A0A087X2D0	Serine/arginine-rich-splicing factor 3 OS=Homo sapiens GN=SRSF3 PE=1 SV=1 - [A0A087X2D0_HUMAN]	30.53	2	2	2	95	10.31397	5.135254	1.1717	1.06487	1.16938	1.1353177	0.0615936	0.1162824
P51572	B-cell receptor-associated protein 31 OS=Homo sapiens GN=BCAP31 PE=1 SV=3 - [BAP31_HUMAN]	30.49	8	8	11	246	27.97401	8.440918	1.10756	1.25416	1.0436	1.135105	0.162467	0.2296246
Q16270	Insulin-like growth factor-binding protein 7 OS=Homo sapiens GN=IGFBP7 PE=1 SV=1 - [IBP7_HUMAN]	31.91	8	8	9	282	29.11144	7.898926	1.14732	1.11704	1.13928	1.1345451	0.00450001	0.0398479

Biology Open • Supplementary information

B4DZC0	cDNA FLJ51771, highly similar to SWI/SNF-related matrix-associatedactin-dependent regulator of chromatin subfamily A member5 (EC 3.6.1.-) OS=Homo sapiens PE=2 SV=1 - [B4DZC0_HUMAN]	4.32	4	4	5	995	116.6829	8.338379	1.13351	1.10249	1.16724	1.1344155	0.0188011	0.0601507
A0A024R9R8	Synaptosomal-associated protein, 23kDa, isoform CRA_b OS=Homo sapiens GN=SNAP23 PE=4 SV=1 - [A0A024R9R8_HUMAN]	28.48	3	3	4	158	17.77779	4.690918	1.11098	1.15756	1.13465	1.1343948	0.0098655	0.0479809
B7Z565	cDNA FLJ54739, highly similar to Alpha-actinin-1 OS=Homo sapiens PE=2 SV=1 - [B7Z565_HUMAN]	54.38	2	41	139	822	94.7237	5.693848	1.2311	1.08624	1.08577	1.1343733	0.108809	0.1719131
K7ENG2	Splicing factor U2AF 65 kDa subunit OS=Homo sapiens GN=U2AF2 PE=1 SV=1 - [K7ENG2_HUMAN]	19.22	3	3	4	307	33.87993	5.033691	1.06033	1.1465	1.19467	1.1338311	0.0764538	0.1345794
P28838	Cytosol aminopeptidase OS=Homo sapiens GN=LAP3 PE=1 SV=3 - [AMPL_HUMAN]	50.87	20	20	35	519	56.13081	7.928223	1.1146	1.19131	1.09437	1.133423	0.0456414	0.0970453
A0A024R7F1	Protein kinase C substrate 80K-H, isoform CRA_a OS=Homo sapiens GN=PRKCSH PE=4 SV=1 - [A0A024R7F1_HUMAN]	21.44	9	9	17	527	59.25878	4.411621	1.15685	1.13671	1.10653	1.1333616	0.0118115	0.0495204
M0R3D6	60S ribosomal protein L18a (Fragment) OS=Homo sapiens GN=RPL18A PE=1 SV=1 - [M0R3D6_HUMAN]	37.59	5	5	11	141	16.70379	10.77002	1.09812	1.18505	1.11622	1.1331328	0.0373617	0.0865636
Q8NC73	Cation-transporting ATPase OS=Homo sapiens PE=2 SV=1 - [Q8NC73_HUMAN]	5.94	3	3	3	572	63.23739	8.367676	1.14813	1.04471	1.20562	1.1328229	0.10602	0.1689631
P53999	Activated RNA polymerase II transcriptional coactivator p15 OS=Homo sapiens GN=SUB1 PE=1 SV=3 - [TCP4_HUMAN]	26.77	4	4	6	127	14.38641	9.598145	1.1563	1.0441	1.19518	1.1318616	0.100516	0.1627974
P07954	Fumarate hydratase, mitochondrial OS=Homo sapiens GN=FH PE=1 SV=3 - [FUMH_HUMAN]	22.75	9	9	12	510	54.60217	8.763184	1.13906	1.13235	1.12343	1.1316143	0.00118003	0.0276207
Q86UX7	Fermitin family homolog 3 OS=Homo sapiens GN=FERMT3 PE=1 SV=1 - [URP2_HUMAN]	22.94	11	11	14	667	75.90501	6.976074	1.1137	1.18167	1.09913	1.1314993	0.0354312	0.0833458

Biology Open • Supplementary information

Q9Y6G9	Cytoplasmic dynein 1 light intermediate chain 1 OS=Homo sapiens GN=DYNC1LI1 PE=1 SV=3 - [DC1L1_HUMAN]	8.8	4	4	5	523	56.544	6.41748	1.0935	1.19866	1.10146	1.1312072	0.0604254	0.1150645
Q0QF37	Malate dehydrogenase (Fragment) OS=Homo sapiens GN=MDH2 PE=2 SV=1 - [Q0QF37_HUMAN]	60	14	14	34	305	31.94876	7.884277	1.12661	1.09604	1.16889	1.1305163	0.0252	0.0696343
Q9Y266	Nuclear migration protein nudC OS=Homo sapiens GN=NUDC PE=1 SV=1 - [NUDC_HUMAN]	11.78	4	4	5	331	38.21913	5.376465	1.09484	1.17701	1.11964	1.1304943	0.0330579	0.0802733
X6R433	Protein-tyrosine-phosphatase OS=Homo sapiens GN=PTPRC PE=1 SV=1 - [X6R433_HUMAN]	7.86	8	8	11	1145	131.0471	6.328613	1.17579	1.10011	1.11469	1.1301982	0.0302735	0.0771075
P54136	Arginine--tRNA ligase, cytoplasmic OS=Homo sapiens GN=RARS PE=1 SV=2 - [SYRC_HUMAN]	23.03	11	11	14	660	75.33087	6.683105	1.11411	1.13582	1.14043	1.1301192	0.00386551	0.0387187
K7ELW0	Protein deglycase DJ-1 OS=Homo sapiens GN=PARK7 PE=1 SV=1 - [K7ELW0_HUMAN]	41.42	8	8	20	169	17.89849	7.869629	1.11594	1.17729	1.09655	1.1299261	0.0333354	0.0806036
P09669	Cytochrome c oxidase subunit 6C OS=Homo sapiens GN=COX6C PE=1 SV=2 - [COX6C_HUMAN]	49.33	5	5	6	75	8.77569	10.38916	1.13298	1.11332	1.14258	1.1296266	0.00438284	0.0395189
P13647	Keratin, type II cytoskeletal 5 OS=Homo sapiens GN=KRT5 PE=1 SV=3 - [K2C5_HUMAN]	27.97	7	18	27	590	62.33998	7.737793	1.11179	1.11493	1.16062	1.129113	0.0146086	0.0534596
P36578	60S ribosomal protein L4 OS=Homo sapiens GN=RPL4 PE=1 SV=5 - [RL4_HUMAN]	26.46	9	9	18	427	47.66743	11.06299	1.07214	1.14908	1.16558	1.1289353	0.0464267	0.0980592
B3KX72	cDNA FLJ44920 fis, clone BRAMY3011501, highly similar to Heterogeneous nuclear ribonucleoprotein U OS=Homo sapiens PE=2 SV=1 - [B3KX72_HUMAN]	20.8	14	14	27	750	83.033	8.79248	1.15729	1.13618	1.0927	1.1287239	0.0211308	0.063152
B7Z832	cDNA FLJ51409, highly similar to Thrombospondin-4 OS=Homo sapiens PE=2 SV=1 - [B7Z832_HUMAN]	3.1	2	2	2	870	95.88672	4.640137	1.18877	1.01831	1.1774	1.1281601	0.145226	0.2110569
Q04917	14-3-3 protein eta OS=Homo sapiens GN=YWHAH PE=1 SV=4 - [1433F_HUMAN]	46.75	8	12	30	246	28.20102	4.843262	1.10815	1.20075	1.07508	1.1279944	0.0765495	0.1346778
Q15417	Calponin-3 OS=Homo sapiens GN=CNN3 PE=1 SV=1 -	28.57	5	7	8	329	36.39079	6.049316	1.1687	1.01204	1.20247	1.1277353	0.16136	0.2287285

Biology Open • Supplementary information

	[CNN3_HUMAN]													
P37108	Signal recognition particle 14 kDa protein OS=Homo sapiens GN=SRP14 PE=1 SV=2 - [SRP14_HUMAN]	23.53	3	3	3	136	14.56082	10.0376	1.12461	1.13258	1.12581	1.1276656	0.000378317	0.0212234
A0A087WY71	AP-2 complex subunit mu OS=Homo sapiens GN=AP2M1 PE=1 SV=1 - [A0A087WY71_HUMAN]	22.35	8	8	11	434	49.49497	9.539551	1.14398	1.12217	1.11618	1.127444	0.0043647	0.039504
P63167	Dynein light chain 1, cytoplasmic OS=Homo sapiens GN=DYNLL1 PE=1 SV=1 - [DYL1_HUMAN]	44.94	2	3	7	89	10.35911	7.400879	1.16621	1.12545	1.09047	1.1273776	0.0282698	0.0744405
O95340	Bifunctional 3'-phosphoadenosine 5'-phosphosulfate synthase 2 OS=Homo sapiens GN=PAPSS2 PE=1 SV=2 - [PAPS2_HUMAN]	9.77	5	5	5	614	69.4566	8.030762	1.11704	1.12551	1.1394	1.127318	0.00260978	0.0357839
F5GY37	Prohibitin-2 OS=Homo sapiens GN=PHB2 PE=1 SV=1 - [F5GY37_HUMAN]	28.84	6	6	8	267	29.70499	9.876465	1.18742	1.01323	1.18078	1.1271464	0.155383	0.2228693
P31153	S-adenosylmethionine synthase isoform type-2 OS=Homo sapiens GN=MAT2A PE=1 SV=1 - [METK2_HUMAN]	17.47	5	5	5	395	43.63334	6.480957	1.12024	1.08029	1.18007	1.1268681	0.0484676	0.1005287
B2RDW0	cDNA, FLJ96792, highly similar to Homo sapiens calmodulin 2 (phosphorylase kinase, delta) (CALM2), mRNA OS=Homo sapiens PE=2 SV=1 - [B2RDW0_HUMAN]	55.03	10	10	34	149	16.7668	4.221191	1.13444	1.18239	1.06268	1.1265044	0.0679836	0.1237894
P62424	60S ribosomal protein L7a OS=Homo sapiens GN=RPL7A PE=1 SV=2 - [RL7A_HUMAN]	29.7	7	7	12	266	29.97703	10.60889	1.15888	1.17036	1.05011	1.1264477	0.0808328	0.1403894
B4E3A8	cDNA FLJ53963, highly similar to Leukocyte elastase inhibitor OS=Homo sapiens PE=2 SV=1 - [B4E3A8_HUMAN]	24.63	7	8	10	341	38.66069	6.668457	1.16766	1.07321	1.13837	1.1264149	0.0454503	0.0967605
K7EJR3	26S proteasome non-ATPase regulatory subunit 8 (Fragment) OS=Homo sapiens GN=PSMD8 PE=1 SV=6 - [K7EJR3_HUMAN]	16.4	4	4	4	250	28.04763	8.177246	1.201	1.03242	1.14559	1.1263356	0.12576	0.1908958
A0A0A0MRB1	Gamma-interferon-inducible protein 16 OS=Homo sapiens GN=IFI16 PE=1 SV=1 - [A0A0A0MRB1_HUMAN]	17.28	4	4	5	243	27.30824	8.382324	1.21762	1.0442	1.11566	1.1258278	0.129556	0.1945639

Biology Open • Supplementary information

P48444	Coatomer subunit delta OS=Homo sapiens GN=ARCN1 PE=1 SV=1 - [COPD_HUMAN]	14.29	7	7	14	511	57.17413	6.214355	1.07862	1.19857	1.09984	1.1256804	0.0766599	0.134802
B2RAH7	cDNA, FLJ94921, highly similar to Homo sapiens prolyl endopeptidase (PREP), mRNA OS=Homo sapiens PE=2 SV=1 - [B2RAH7_HUMAN]	11.83	7	7	7	710	80.68202	5.858887	1.11718	1.1503	1.10951	1.1256636	0.00977536	0.0478721
A0A024QZN4	Vinculin, isoform CRA_c OS=Homo sapiens GN=VCL PE=4 SV=1 - [A0A024QZN4_HUMAN]	59.01	64	64	181	1066	116.6493	6.087402	1.12442	1.14863	1.10297	1.1253395	0.01089	0.0493157
A0A024RD07	Trinucleotide repeat containing 5, isoform CRA_c OS=Homo sapiens GN=TNRC5 PE=4 SV=1 - [A0A024RD07_HUMAN]	9.35	2	2	2	278	30.75471	5.490723	1.11942	1.15482	1.10156	1.1252686	0.015253	0.0543533
E9PQR7	Vacuolar protein sorting-associated protein 28 homolog (Fragment) OS=Homo sapiens GN=VPS28 PE=1 SV=1 - [E9PQR7_HUMAN]	15.53	2	2	2	161	18.45742	6.214355	1.10293	1.11678	1.15558	1.1250969	0.0154978	0.0547029
P12429	Annexin A3 OS=Homo sapiens GN=ANXA3 PE=1 SV=3 - [ANXA3_HUMAN]	34.98	10	10	13	323	36.35266	5.922363	1.15016	1.12844	1.09637	1.1249872	0.0152703	0.0543533
Q9BZF1	Oxysterol-binding protein-related protein 8 OS=Homo sapiens GN=OSBPL8 PE=1 SV=3 - [OSBL8_HUMAN]	4.27	3	3	3	889	101.1322	6.961426	1.01855	1.21616	1.13952	1.1247426	0.162368	0.2295805
Q96QK1	Vacuolar protein sorting-associated protein 35 OS=Homo sapiens GN=VPS35 PE=1 SV=2 - [VPS35_HUMAN]	20.48	16	16	19	796	91.64894	5.490723	1.10559	1.07682	1.18676	1.1230572	0.0646877	0.1201248
Q9BUH6	Protein PAXX OS=Homo sapiens GN=C9orf142 PE=1 SV=2 - [PAXX_HUMAN]	21.08	3	3	3	204	21.62597	5.478027	1.2638	1.04013	1.06467	1.1228673	0.224888	0.2949685
F8W651	Coatomer subunit zeta-1 OS=Homo sapiens GN=COPZ1 PE=1 SV=1 - [F8W651_HUMAN]	28.57	2	2	2	119	13.38413	4.817871	1.15353	1.07972	1.13474	1.122664	0.0310875	0.0777188
P13010	X-ray repair cross-complementing protein 5 OS=Homo sapiens GN=XRCC5 PE=1 SV=3 - [XRCC5_HUMAN]	25.82	16	17	25	732	82.65228	5.808105	1.09164	1.15558	1.11911	1.1221102	0.0222397	0.0647174
F5GYQ2	Multifunctional methyltransferase subunit TRM112-like protein OS=Homo sapiens GN=TRMT112 PE=1 SV=1 -	34.57	2	2	2	81	9.249595	4.411621	1.27765	0.784138	1.30329	1.1216942	0.54613	0.6124888

Biology Open • Supplementary information

	[F5GYQ2_HUMAN]													
E9PLT0	Cold shock domain-containing protein E1 OS=Homo sapiens GN=CSDE1 PE=1 SV=1 - [E9PLT0_HUMAN]	5.24	4	4	4	668	74.53676	6.087402	1.10674	1.11456	1.14345	1.1215851	0.00832743	0.0456887
B7Z5C0	cDNA FLJ52352, highly similar to DnaJ homolog subfamily A member 1 OS=Homo sapiens PE=2 SV=1 - [B7Z5C0_HUMAN]	11.25	3	3	4	240	27.52575	5.935059	1.08622	1.11734	1.16041	1.1213234	0.0300192	0.0767182
B5BUB5	Autoantigen La (Fragment) OS=Homo sapiens GN=SSB PE=2 SV=1 - [B5BUB5_HUMAN]	14.22	5	5	6	408	46.83817	7.122559	1.12802	1.15828	1.07729	1.1211973	0.035965	0.084367
P43034	Platelet-activating factor acetylhydrolase IB subunit alpha OS=Homo sapiens GN=PAFAH1B1 PE=1 SV=2 - [LIS1_HUMAN]	18.54	6	6	6	410	46.60816	7.371582	1.12371	1.11686	1.12215	1.1209031	0.000293956	0.0212234
B2R8A2	cDNA, FLJ93804, highly similar to Homo sapiens gp25L2 protein (HSGP25L2G), mRNA OS=Homo sapiens PE=2 SV=1 - [B2R8A2_HUMAN]	9.35	2	2	3	214	25.06191	7.181152	1.11875	1.13403	1.10954	1.1207749	0.00347634	0.0373569
A0A087WUY5	Sorting nexin-5 (Fragment) OS=Homo sapiens GN=SNX5 PE=1 SV=1 - [A0A087WUY5_HUMAN]	11.76	2	2	5	170	19.71512	6.91748	1.09131	1.21342	1.05595	1.1202256	0.127901	0.1931066
O43776	Asparagine--tRNA ligase, cytoplasmic OS=Homo sapiens GN=NARS PE=1 SV=1 - [SYNC_HUMAN]	14.42	7	7	8	548	62.90252	6.252441	1.12338	1.19915	1.03797	1.1201652	0.123022	0.1881742
A8K5M4	Non-specific serine/threonine protein kinase OS=Homo sapiens PE=2 SV=1 - [A8K5M4_HUMAN]	20.04	7	7	11	524	58.00687	5.846191	1.12567	1.14305	1.09165	1.1201255	0.0154228	0.0546326
Q4W4Y1	Dopamine receptor interacting protein 4 OS=Homo sapiens GN=DRIP4 PE=2 SV=1 - [Q4W4Y1_HUMAN]	23.39	18	18	23	868	96.01919	6.521973	1.1335	1.13041	1.09344	1.1191169	0.0114734	0.0494101
P20618	Proteasome subunit beta type-1 OS=Homo sapiens GN=PSMB1 PE=1 SV=2 - [PSB1_HUMAN]	28.22	5	5	7	241	26.47237	8.133301	1.10649	1.11659	1.13248	1.1185197	0.00404937	0.0390309
P55072	Transitional endoplasmic reticulum ATPase OS=Homo	49.01	28	28	46	806	89.26572	5.262207	1.12741	1.12517	1.10149	1.1180237	0.0049007	0.0401071

Biology Open • Supplementary information

	sapiens GN=VCP PE=1 SV=4 - [TERA_HUMAN]														
P45973	Chromobox protein homolog 5 OS=Homo sapiens GN=CBX5 PE=1 SV=1 - [CBX5_HUMAN]	13.61	2	2	2	191	22.21106	5.858887	1.07503	1.14902	1.12963	1.1178931	0.0335368	0.0809992
F5H1S8	Malectin (Fragment) OS=Homo sapiens GN=MLEC PE=1 SV=1 - [F5H1S8_HUMAN]	39.04	5	5	5	146	16.71859	5.566895	1.23029	1.01622	1.1039	1.116801	0.200883	0.2710199
Q7Z3N6	Putative uncharacterized protein DKFZp686A01173 (Fragment) OS=Homo sapiens GN=DKFZp686A01173 PE=2 SV=1 - [Q7Z3N6_HUMAN]	6.76	6	6	6	917	105.7363	9.495605	1.10769	1.12041	1.11921	1.1157697	0.00122396	0.0276207
C9J6X0	NADH dehydrogenase [ubiquinone] 1 alpha subcomplex subunit 10, mitochondrial (Fragment) OS=Homo sapiens GN=NDUFA10 PE=1 SV=6 - [C9J6X0_HUMAN]	7.38	2	2	2	244	27.6775	9.656738	1.05936	1.17494	1.11154	1.1152792	0.0747385	0.1324554
O14980	Exportin-1 OS=Homo sapiens GN=XPO1 PE=1 SV=1 - [XPO1_HUMAN]	14.75	11	11	12	1071	123.3061	6.062012	1.09635	1.13224	1.11105	1.1132162	0.00835846	0.0456887
P35580	Myosin-10 OS=Homo sapiens GN=MYH10 PE=1 SV=3 - [MYH10_HUMAN]	30.57	32	58	116	1976	228.858	5.541504	1.11192	1.14172	1.08596	1.1131978	0.0196609	0.0612255
B4DTX5	cDNA FLJ60072, highly similar to Homo sapiens sorbin and SH3 domain containing 1 (SORBS1), transcript variant 6, mRNA OS=Homo sapiens PE=2 SV=1 - [B4DTX5_HUMAN]	25.1	7	15	25	749	83.86202	7.151855	1.1157	1.12594	1.09748	1.1130393	0.00537921	0.0401071
Q07960	Rho GTPase-activating protein 1 OS=Homo sapiens GN=ARHGAP1 PE=1 SV=1 - [RHG01_HUMAN]	25.74	11	11	13	439	50.40419	6.290527	1.10643	1.15525	1.07728	1.1129862	0.0382125	0.0874573
P51149	Ras-related protein Rab-7a OS=Homo sapiens GN=RAB7A PE=1 SV=1 - [RAB7A_HUMAN]	64.25	11	11	17	207	23.47484	6.697754	1.05871	1.14638	1.13365	1.1129155	0.0539589	0.1071895
C9JRH2	Regulator of chromosome condensation (Fragment) OS=Homo sapiens GN=RCC1 PE=1 SV=1 - [C9JRH2_HUMAN]	15.65	2	2	2	230	24.44958	7.415527	1.03989	1.15134	1.14576	1.1123312	0.0902866	0.1512099

Biology Open • Supplementary information

I3L397	Eukaryotic translation initiation factor 5A (Fragment) OS=Homo sapiens GN=EIF5A PE=1 SV=6 - [I3L397_HUMAN]	22.6	4	4	10	146	16.00891	4.995605	1.11784	1.15539	1.06268	1.1119698	0.0532426	0.1064538
P00488	Coagulation factor XIII A chain OS=Homo sapiens GN=F13A1 PE=1 SV=4 - [F13A_HUMAN]	31.42	6	20	50	732	83.21461	6.087402	1.10004	1.11455	1.12086	1.1118134	0.00302528	0.0360868
Q13185	Chromobox protein homolog 3 OS=Homo sapiens GN=CBX3 PE=1 SV=4 - [CBX3_HUMAN]	22.95	3	3	3	183	20.79835	5.325684	1.09456	1.16143	1.07923	1.1117396	0.0474113	0.0993395
P04083	Annexin A1 OS=Homo sapiens GN=ANXA1 PE=1 SV=2 - [ANXA1_HUMAN]	56.65	20	20	55	346	38.68998	7.02002	1.11728	1.07606	1.14116	1.1115005	0.0278663	0.0739823
P13804	Electron transfer flavoprotein subunit alpha, mitochondrial OS=Homo sapiens GN=ETFA PE=1 SV=1 - [ETFA_HUMAN]	23.42	6	6	13	333	35.05758	8.382324	1.09574	1.15394	1.08452	1.1113988	0.0353317	0.0832854
O43390	Heterogeneous nuclear ribonucleoprotein R OS=Homo sapiens GN=HNRNPR PE=1 SV=1 - [HNRPR_HUMAN]	21.64	9	11	14	633	70.89925	8.133301	1.09025	1.08533	1.15811	1.1112317	0.0417918	0.0923402
Q05DH1	Proteasome subunit alpha type (Fragment) OS=Homo sapiens GN=PSMA7 PE=2 SV=1 - [Q05DH1_HUMAN]	34.03	6	6	10	238	26.69708	8.865723	1.10179	1.11338	1.11847	1.1112144	0.00196268	0.0321184
P30048	Thioredoxin-dependent peroxide reductase, mitochondrial OS=Homo sapiens GN=PRDX3 PE=1 SV=3 - [PRDX3_HUMAN]	11.33	3	3	8	256	27.67518	7.781738	1.07445	1.06086	1.19613	1.1104797	0.123971	0.1892836
P08238	Heat shock protein HSP 90-beta OS=Homo sapiens GN=HSP90AB1 PE=1 SV=4 - [HS90B_HUMAN]	34.53	13	26	61	724	83.21211	5.033691	1.11483	1.08376	1.1328	1.1104627	0.0164024	0.0563479
P08708	40S ribosomal protein S17 OS=Homo sapiens GN=RPS17 PE=1 SV=2 - [RS17_HUMAN]	54.81	6	6	14	135	15.54039	9.847168	1.17347	1.16533	0.992268	1.1103572	0.202783	0.2732566
Q15149	Plectin OS=Homo sapiens GN=PLEC PE=1 SV=3 - [PLEC_HUMAN]	37.51	161	166	262	4684	531.466	5.960449	1.11978	1.08969	1.12093	1.1101307	0.00851497	0.0456887
F2Z2V0	Copine-1 (Fragment) OS=Homo sapiens GN=CPNE1 PE=1 SV=1 - [F2Z2V0_HUMAN]	11.82	6	6	11	533	58.59734	5.833496	1.11084	1.1668	1.05106	1.1095676	0.0817735	0.1412301

P50479	PDZ and LIM domain protein 4 OS=Homo sapiens GN=PDLIM4 PE=1 SV=2 - [PDLI4_HUMAN]	20.61	6	6	7	330	35.37566	7.913574	1.18052	1.04727	1.09988	1.1092252	0.106185	0.1690304
P48047	ATP synthase subunit O, mitochondrial OS=Homo sapiens GN=ATP5O PE=1 SV=1 - [ATPO_HUMAN]	39.44	7	7	10	213	23.26266	9.964355	1.09377	1.13488	1.09828	1.1089735	0.0139706	0.0526821
P07339	Cathepsin D OS=Homo sapiens GN=CTSD PE=1 SV=1 - [CATD_HUMAN]	32.04	11	11	32	412	44.52363	6.536621	1.11327	1.1059	1.10759	1.1089182	0.000418661	0.0220588
A0A087X020	Ribosome maturation protein SBDS OS=Homo sapiens GN=SBDS PE=1 SV=1 - [A0A087X020_HUMAN]	36	8	8	9	250	28.84635	8.748535	1.10031	1.12934	1.09607	1.1085734	0.00914358	0.0467538
Q2TAY7	WD40 repeat-containing protein SMU1 OS=Homo sapiens GN=SMU1 PE=1 SV=2 - [SMU1_HUMAN]	3.31	2	2	2	513	57.50723	7.181152	1.07001	1.2168	1.03869	1.1085001	0.186761	0.2557387
O60763	General vesicular transport factor p115 OS=Homo sapiens GN=USO1 PE=1 SV=2 - [USO1_HUMAN]	11.33	9	9	11	962	107.8281	4.906738	1.07953	1.12526	1.1201	1.1082979	0.0173643	0.05785
H3BT29	Protein PML (Fragment) OS=Homo sapiens GN=PML PE=1 SV=1 - [H3BT29_HUMAN]	26.67	6	6	7	240	26.5324	5.681152	1.14206	1.07559	1.10723	1.1082913	0.0300072	0.0767182
A0A024R1Y2	ATP-citrate synthase OS=Homo sapiens GN=ACLY PE=3 SV=1 - [A0A024R1Y2_HUMAN]	16.59	13	13	20	1091	119.6953	7.327637	1.09634	1.13251	1.09546	1.1081045	0.0125101	0.050533
A0A0A0MR02	Voltage-dependent anion-selective channel protein 2 (Fragment) OS=Homo sapiens GN=VDAC2 PE=1 SV=1 - [A0A0A0MR02_HUMAN]	36.52	9	9	13	282	30.32887	7.811035	1.0914	1.16675	1.06514	1.1077646	0.071408	0.1283675
A0A087WYS1	UTP--glucose-1-phosphate uridylyltransferase OS=Homo sapiens GN=UGP2 PE=1 SV=1 - [A0A087WYS1_HUMAN]	24.8	11	11	17	508	56.93176	7.884277	1.11517	1.15815	1.04967	1.1076665	0.0761468	0.1341785
O95834	Echinoderm microtubule-associated protein-like 2 OS=Homo sapiens GN=EML2 PE=1 SV=1 - [EMAL2_HUMAN]	12.17	6	6	6	649	70.63408	6.315918	1.07181	1.05764	1.19248	1.1073083	0.128914	0.1942894
B4DRN6	cDNA FLJ51284, highly similar to Adapter-relatedprotein complex 3 mu-1 subunit OS=Homo sapiens PE=2 SV=1 - [B4DRN6_HUMAN]	9.62	3	3	3	364	41.00024	8.250488	1.00066	1.20652	1.11382	1.1069992	0.214073	0.2833935

Biology Open • Supplementary information

O95831	Apoptosis-inducing factor 1, mitochondrial OS=Homo sapiens GN=AIFM1 PE=1 SV=1 - [AIFM1_HUMAN]	21.37	9	9	10	613	66.85891	8.953613	1.10164	1.13691	1.08105	1.1065344	0.0226455	0.0652383
A0A024RDE8	PDZ and LIM domain 5, isoform CRA_c OS=Homo sapiens GN=PDLIM5 PE=4 SV=1 - [A0A024RDE8_HUMAN]	25.67	4	12	20	596	63.9345	8.206543	1.10846	1.15422	1.05677	1.1064837	0.0633179	0.1181539
P63104	14-3-3 protein zeta/delta OS=Homo sapiens GN=YWHAZ PE=1 SV=1 - [1433Z_HUMAN]	56.73	11	15	54	245	27.72773	4.79248	1.08372	1.1137	1.1206	1.106005	0.0112124	0.0493721
D6R938	Calcium/calmodulin-dependent protein kinase (CaM kinase) II delta, isoform CRA_e OS=Homo sapiens GN=CAMK2D PE=1 SV=1 - [D6R938_HUMAN]	12.05	4	6	9	498	56.26252	7.254395	1.04893	1.16591	1.10307	1.1059706	0.0884524	0.1494069
B3KQ51	Serine/threonine-protein phosphatase OS=Homo sapiens PE=2 SV=1 - [B3KQ51_HUMAN]	18.85	3	3	5	244	28.07174	5.960449	1.0589	1.09969	1.15916	1.1059165	0.0679188	0.123738
O60884	DnaJ homolog subfamily A member 2 OS=Homo sapiens GN=DNAJA2 PE=1 SV=1 - [DNJA2_HUMAN]	9.47	4	4	4	412	45.71659	6.480957	1.09805	1.14105	1.07852	1.1058735	0.0291099	0.0756232
O94804	Serine/threonine-protein kinase 10 OS=Homo sapiens GN=STK10 PE=1 SV=1 - [STK10_HUMAN]	3.41	2	3	3	968	112.0651	6.946777	1.14206	1.03689	1.13769	1.1055459	0.0916082	0.1526499
P10599	Thioredoxin OS=Homo sapiens GN=TXN PE=1 SV=3 - [THIO_HUMAN]	42.86	4	4	6	105	11.72974	4.919434	1.04798	1.1835	1.08408	1.1051881	0.121857	0.1867297
A8K5W7	cDNA FLJ75180, highly similar to Homo sapiens mitochondrial isoleucine tRNA synthetase, mRNA OS=Homo sapiens PE=2 SV=1 - [A8K5W7_HUMAN]	3.19	3	3	3	940	105.873	6.41748	1.1202	1.20083	0.993806	1.1049461	0.22365	0.2936599
B7Z4V2	cDNA FLJ51907, highly similar to Stress-70 protein, mitochondrial OS=Homo sapiens PE=2 SV=1 - [B7Z4V2_HUMAN]	31.73	18	18	32	665	72.35606	5.935059	1.0936	1.11654	1.1047	1.1049457	0.00395884	0.0390309
P30086	Phosphatidylethanolamine-binding protein 1 OS=Homo sapiens GN=PEBP1 PE=1 SV=3 - [PEBP1_HUMAN]	39.04	7	7	12	187	21.04367	7.532715	1.12081	1.12109	1.07271	1.1048694	0.0227144	0.0652702

Biology Open • Supplementary information

B3KML9	cDNA FLJ11352 fis, clone HEMBA1000020, highly similar to Tubulin beta-2C chain OS=Homo sapiens PE=2 SV=1 - [B3KML9_HUMAN]	50.13	4	16	80	397	44.57354	4.932129	1.09254	1.10171	1.12017	1.1048083	0.00595656	0.0419659
B2R4A2	Cytochrome b-c1 complex subunit 7 OS=Homo sapiens PE=2 SV=1 - [B2R4A2_HUMAN]	45.95	6	6	8	111	13.52296	8.265137	1.13087	1.16324	1.02015	1.1047517	0.136788	0.2018428
A0A024R152	HCG28765, isoform CRA_b OS=Homo sapiens GN=hCG_28765 PE=4 SV=1 - [A0A024R152_HUMAN]	1.8	2	2	2	1337	147.1371	5.008301	1.13375	1.09574	1.08398	1.1044909	0.0200369	0.0618276
A8K6Y1	cDNA FLJ75526, highly similar to Homo sapiens proliferation-associated 2G4, 38kDa (PA2G4), mRNA (Fragment) OS=Homo sapiens PE=2 SV=1 - [A8K6Y1_HUMAN]	26.43	8	8	11	367	40.88572	6.404785	1.09261	1.13561	1.08464	1.1042883	0.0222735	0.0647174
F8VZJ2	Nascent polypeptide-associated complex subunit alpha OS=Homo sapiens GN=NACA PE=1 SV=1 - [F8VZJ2_HUMAN]	40.44	4	4	12	136	15.00676	4.906738	1.14112	1.12532	1.04545	1.1039613	0.0724239	0.129643
X5DP48	Adenylosuccinate lyase isoform D OS=Homo sapiens GN=ADSL PE=2 SV=1 - [X5DP48_HUMAN]	15.67	4	4	4	351	39.99936	8.133301	1.03978	1.12343	1.1485	1.1039022	0.0871794	0.147625
P22695	Cytochrome b-c1 complex subunit 2, mitochondrial OS=Homo sapiens GN=UQCRC2 PE=1 SV=3 - [QCR2_HUMAN]	26.71	9	9	13	453	48.41289	8.631348	1.09888	1.10654	1.10567	1.1036988	0.000544817	0.0241782
Q5QPP3	UDP-glucose 4-epimerase (Fragment) OS=Homo sapiens GN=GALE PE=1 SV=1 - [Q5QPP3_HUMAN]	11.89	2	2	3	227	25.01463	8.060059	1.13355	1.21228	0.965075	1.1036347	0.291118	0.3635061
B4DWV5	GrpE protein homolog OS=Homo sapiens PE=2 SV=1 - [B4DWV5_HUMAN]	10.2	2	2	3	196	21.88665	8.616699	0.993088	1.22917	1.08827	1.1035109	0.270254	0.3421129
O75663	TIP41-like protein OS=Homo sapiens GN=TIPRL PE=1 SV=2 - [TIPRL_HUMAN]	7.35	2	2	2	272	31.42372	5.909668	1.08965	1.11116	1.10924	1.103349	0.00438969	0.0395189
O75947	ATP synthase subunit d, mitochondrial OS=Homo sapiens GN=ATP5H PE=1 SV=3 - [ATP5H_HUMAN]	49.69	6	6	13	161	18.4795	5.300293	1.10554	1.16154	1.04235	1.1031414	0.0956916	0.1573082

F8VYY9	5'-AMP-activated protein kinase subunit gamma-1 OS=Homo sapiens GN=PRKAG1 PE=1 SV=1 - [F8VYY9_HUMAN]	17.86	4	4	4	280	31.8852	8.572754	1.19724	0.982374	1.12838	1.1026662	0.246522	0.3162095
B4DF22	cDNA FLJ55615, highly similar to SWI/SNF-related matrix-associatedactin-dependent regulator of chromatin subfamily C member 2 OS=Homo sapiens PE=2 SV=1 - [B4DF22_HUMAN]	3.73	3	3	3	1019	112.143	5.287598	1.12792	1.12621	1.05242	1.1021795	0.0545106	0.1079486
B4DSQ5	cDNA FLJ53608, highly similar to Protein transport protein Sec23A OS=Homo sapiens PE=2 SV=1 - [B4DSQ5_HUMAN]	13.32	8	8	9	736	82.87453	7.195801	1.08947	1.05716	1.15759	1.1014052	0.0756606	0.1337395
Q53FE8	cDNA FLJ36526 fis, clone TRACH2003347, highly similar to NSFL1 cofactor p47 (Fragment) OS=Homo sapiens PE=2 SV=1 - [Q53FE8_HUMAN]	14.86	3	3	3	370	40.54733	5.135254	1.19321	1.11929	0.991596	1.101368	0.227327	0.2973346
B4DZC9	cDNA FLJ61681, highly similar to STE20-like serine/threonine-protein kinase (EC2.7.11.1) (Fragment) OS=Homo sapiens PE=2 SV=1 - [B4DZC9_HUMAN]	4.96	2	3	3	1048	119.7884	4.906738	1.18757	1.06332	1.05128	1.1007223	0.146912	0.212884
Q59GB4	Dihydropyrimidinase-like 2 variant (Fragment) OS=Homo sapiens PE=2 SV=1 - [Q59GB4_HUMAN]	41.4	16	18	40	628	68.14153	6.239746	1.08132	1.11526	1.10289	1.0998214	0.00972798	0.0478622
E5KSX8	Mitochondrial transcription factor A OS=Homo sapiens PE=4 SV=1 - [E5KSX8_HUMAN]	12.2	3	3	3	246	29.09218	9.715332	1.08957	1.14756	1.06159	1.099573	0.0589742	0.1136185
G1UI32	Pyridoxal kinase (Fragment) OS=Homo sapiens GN=PDXK PE=2 SV=1 - [G1UI32_HUMAN]	16.27	2	2	4	166	18.95553	4.970215	0.980994	1.15555	1.16202	1.099524	0.235261	0.3043021
Q13151	Heterogeneous nuclear ribonucleoprotein A0 OS=Homo sapiens GN=HNRNPA0 PE=1 SV=1 - [ROA0_HUMAN]	24.92	4	6	10	305	30.82179	9.290527	1.06563	1.05095	1.17967	1.0987505	0.135937	0.2010652
P49419	Alpha-aminoadipic semialdehyde dehydrogenase OS=Homo sapiens GN=ALDH7A1 PE=1 SV=5 - [AL7A1_HUMAN]	15.58	7	7	10	539	58.45012	7.986816	1.1	1.09501	1.1006	1.0985346	0.000323263	0.0212234
Q9Y5S9	RNA-binding protein 8A OS=Homo sapiens GN=RBM8A PE=1 SV=1 - [RBM8A_HUMAN]	31.61	5	5	8	174	19.87673	5.719238	1.05932	1.16694	1.06917	1.0984768	0.103171	0.165828

A0A024RCB5	Chitinase domain containing 1, isoform CRA_a OS=Homo sapiens GN=CHID1 PE=3 SV=1 - [A0A024RCB5_HUMAN]	14.77	6	6	7	386	44.08981	7.693848	1.07554	1.11518	1.10427	1.0983319	0.0141487	0.0528571
Q05BW9	PAPSS1 protein (Fragment) OS=Homo sapiens GN=PAPSS1 PE=2 SV=1 - [Q05BW9_HUMAN]	5.95	2	2	2	571	64.46562	6.990723	1.16612	1.03264	1.09485	1.0978721	0.126443	0.1915889
A0A024R5Z9	Pyruvate kinase OS=Homo sapiens GN=PKM2 PE=3 SV=1 - [A0A024R5Z9_HUMAN]	61.77	2	33	75	531	58.02493	7.708496	1.067	1.09766	1.12893	1.0978661	0.0317883	0.079004
P04075	Fructose-bisphosphate aldolase A OS=Homo sapiens GN=ALDOA PE=1 SV=2 - [ALDOA_HUMAN]	45.88	16	18	44	364	39.39531	8.089355	1.12841	1.06038	1.10449	1.0977609	0.0391116	0.0886206
Q9UNM1	Chaperonin 10-related protein (Fragment) OS=Homo sapiens GN=EPFP1 PE=3 SV=1 - [Q9UNM1_HUMAN]	41.24	4	4	9	97	10.28858	8.997559	1.13254	1.12887	1.03147	1.0976237	0.0982776	0.1605179
P62330	ADP-ribosylation factor 6 OS=Homo sapiens GN=ARF6 PE=1 SV=2 - [ARF6_HUMAN]	30.29	4	4	4	175	20.06944	8.953613	1.13439	0.985615	1.17279	1.0975983	0.229411	0.2989862
Q6FH24	VBP1 protein OS=Homo sapiens GN=VBP1 PE=2 SV=1 - [Q6FH24_HUMAN]	14.37	2	2	3	160	18.7236	8.309082	1.05219	1.18648	1.05402	1.0975625	0.15944	0.2268619
O00231	26S proteasome non-ATPase regulatory subunit 11 OS=Homo sapiens GN=PSMD11 PE=1 SV=3 - [PSD11_HUMAN]	21.56	7	7	8	422	47.43404	6.480957	1.11467	1.11401	1.06372	1.0974656	0.0286963	0.0750093
Q9Y3I0	tRNA-splicing ligase RtcB homolog OS=Homo sapiens GN=RTCB PE=1 SV=1 - [RTCB_HUMAN]	18.61	9	9	9	505	55.17486	7.225098	1.09078	1.1655	1.03593	1.0974027	0.121999	0.1868627
O95747	Serine/threonine-protein kinase OSR1 OS=Homo sapiens GN=OXSR1 PE=1 SV=1 - [OXSR1_HUMAN]	13.85	6	6	7	527	57.98605	6.430176	1.10553	1.02557	1.1606	1.0972359	0.131271	0.1962687
A8KAK1	cDNA FLJ77398, highly similar to Homo sapiens UDP-glucose ceramide glucosyltransferase-like 1, transcript variant 2, mRNA OS=Homo sapiens PE=2 SV=1 - [A8KAK1_HUMAN]	5.55	8	8	8	1531	174.897	5.516113	1.07704	1.11798	1.09668	1.0972323	0.0144594	0.0532358
B4E0U6	cDNA FLJ53687, highly similar to Hsc70-interacting protein OS=Homo sapiens PE=2 SV=1 - [B4E0U6_HUMAN]	18.38	6	6	11	359	40.13589	5.300293	1.07292	1.11078	1.10735	1.0970135	0.0151765	0.0543533

Biology Open • Supplementary information

A8KAP9	cDNA FLJ78448, highly similar to Homo sapiens argininosuccinate synthetase (ASS), transcript variant 1, mRNA OS=Homo sapiens PE=2 SV=1 - [A8KAP9_HUMAN]	19.9	8	8	12	412	46.52494	8.030762	1.06376	1.07587	1.15112	1.0969171	0.0711252	0.1280953
P02675	Fibrinogen beta chain OS=Homo sapiens GN=FGB PE=1 SV=2 - [FIBB_HUMAN]	64.15	29	29	105	491	55.89226	8.265137	1.11187	1.11253	1.0659	1.0967676	0.0245088	0.0684507
A0A087WYU1	Sorting nexin OS=Homo sapiens GN=SNX9 PE=1 SV=1 - [A0A087WYU1_HUMAN]	14.65	6	6	8	594	66.43457	5.57959	1.06379	1.15853	1.06793	1.0967512	0.0887022	0.1494818
Q5QPL9	RNA-binding protein Raly (Fragment) OS=Homo sapiens GN=RALY PE=1 SV=1 - [Q5QPL9_HUMAN]	16.88	4	4	6	237	24.65027	10.4917	1.08472	1.14803	1.05719	1.096649	0.0694546	0.125926
Q9Y5L4	Mitochondrial import inner membrane translocase subunit Tim13 OS=Homo sapiens GN=TIMM13 PE=1 SV=1 - [TIM13_HUMAN]	26.32	2	2	4	95	10.493	8.177246	1.08714	1.15048	1.05233	1.0966484	0.0781429	0.136629
A0A024R592	Glucosidase, alpha neutral AB, isoform CRA_b OS=Homo sapiens GN=GANAB PE=3 SV=1 - [A0A024R592_HUMAN]	24.44	20	20	30	847	96.12888	5.833496	1.09231	1.13643	1.06092	1.096553	0.0477901	0.0996118
J3QL05	Serine/arginine-rich-splicing factor 2 (Fragment) OS=Homo sapiens GN=SRSF2 PE=1 SV=1 - [J3QL05_HUMAN]	18.46	2	2	3	130	15.14663	10.96045	1.20779	1.03277	1.04879	1.0964513	0.22639	0.2965674
P23396	40S ribosomal protein S3 OS=Homo sapiens GN=RPS3 PE=1 SV=2 - [RS3_HUMAN]	53.09	13	13	29	243	26.67143	9.656738	1.06385	1.09307	1.1321	1.0963394	0.0396233	0.08917
H3BPK3	Hydroxyacylglutathione hydrolase, mitochondrial (Fragment) OS=Homo sapiens GN=HAGH PE=1 SV=1 - [H3BPK3_HUMAN]	12.97	3	3	4	239	26.3984	8.23584	1.12992	1.09954	1.05951	1.096321	0.0420054	0.0926217
B2R8K8	cDNA, FLJ93949, highly similar to Homo sapiens NIMA (never in mitosis gene a)-related kinase 7 (NEK7), mRNA OS=Homo sapiens PE=2 SV=1 - [B2R8K8_HUMAN]	9.27	2	2	3	302	34.51442	8.250488	1.13171	1.165	0.992222	1.0963106	0.210399	0.2799531
B4DL14	ATP synthase subunit gamma OS=Homo sapiens PE=2 SV=1 - [B4DL14_HUMAN]	16.4	4	4	8	250	27.49525	7.415527	1.09869	1.15621	1.03364	1.0961794	0.113002	0.176354

Biology Open • Supplementary information

M0QYJ8	Glia maturation factor gamma OS=Homo sapiens GN=GMFG PE=1 SV=1 - [M0QYJ8_HUMAN]	31.88	3	4	5	138	16.12503	6.163574	1.18179	1.05723	1.04897	1.0959975	0.15502	0.2224773
B4DKX4	cDNA FLJ58014, highly similar to Homo sapiens programmed cell death 4, transcript variant 1, mRNA OS=Homo sapiens PE=2 SV=1 - [B4DKX4_HUMAN]	5.49	2	2	2	455	50.19743	5.465332	1.15954	1.15891	0.969199	1.0958833	0.269282	0.3413299
B4DLK2	cDNA FLJ55097, highly similar to Adenylate kinase isoenzyme 2, mitochondrial (EC 2.7.4.3) OS=Homo sapiens PE=2 SV=1 - [B4DLK2_HUMAN]	58.2	6	6	8	122	13.08968	5.57959	1.20714	1.03864	1.04115	1.0956471	0.228383	0.2981762
P25786	Proteasome subunit alpha type-1 OS=Homo sapiens GN=PSMA1 PE=1 SV=1 - [PSA1_HUMAN]	31.18	9	9	13	263	29.53686	6.609863	1.06626	1.11959	1.10089	1.0955835	0.0256871	0.070577
A8K787	cDNA FLJ75273, highly similar to Homo sapiens solute carrier family 25 (mitochondrial carrier; adenine nucleotide translocator), member 4, mRNA OS=Homo sapiens PE=2 SV=1 - [A8K787_HUMAN]	20.81	2	6	11	298	33.05921	9.759277	1.12076	1.07818	1.08722	1.0953871	0.0179492	0.0587034
Q9Y490	Talin-1 OS=Homo sapiens GN=TLN1 PE=1 SV=3 - [TLN1_HUMAN]	42.74	72	85	169	2541	269.5991	6.074707	1.08767	1.11051	1.08679	1.0949896	0.00661928	0.0432553
P23381	Tryptophan--tRNA ligase, cytoplasmic OS=Homo sapiens GN=WARS PE=1 SV=2 - [SYWC_HUMAN]	30.57	11	11	22	471	53.13161	6.227051	1.11026	1.09911	1.07384	1.0944049	0.0127724	0.0510309
Q9NR12	PDZ and LIM domain protein 7 OS=Homo sapiens GN=PDLIM7 PE=1 SV=1 - [PDLI7_HUMAN]	37.86	16	16	34	457	49.81261	8.411621	1.05589	1.11402	1.11207	1.0939928	0.03874	0.087951
B4DPN0	cDNA FLJ51265, moderately similar to Beta-2-glycoprotein 1 (Beta-2-glycoprotein I) OS=Homo sapiens PE=2 SV=1 - [B4DPN0_HUMAN]	32.85	6	6	11	274	30.31884	7.85498	1.07088	1.14146	1.06942	1.0939214	0.058498	0.1130872
P49411	Elongation factor Tu, mitochondrial OS=Homo sapiens GN=TUFM PE=1 SV=2 - [EFTU_HUMAN]	40.93	14	14	23	452	49.51018	7.605957	1.12732	1.00852	1.14575	1.093864	0.16075	0.2281504

Biology Open • Supplementary information

B4E2Z3	cDNA FLJ54090, highly similar to 4F2 cell-surface antigen heavy chain OS=Homo sapiens PE=2 SV=1 - [B4E2Z3_HUMAN]	12.92	4	4	4	511	55.90469	5.17334	1.09105	1.13428	1.0557	1.0936771	0.0541044	0.1073525
Q13418	Integrin-linked protein kinase OS=Homo sapiens GN=ILK PE=1 SV=2 - [ILK_HUMAN]	28.1	11	11	29	452	51.38593	8.074707	1.04943	1.14204	1.08748	1.0929841	0.0743435	0.1318935
H3BPC4	SUMO-conjugating enzyme UBC9 (Fragment) OS=Homo sapiens GN=UBE2I PE=1 SV=2 - [H3BPC4_HUMAN]	51.43	4	4	5	70	7.994026	9.715332	1.12231	1.0575	1.09603	1.0919483	0.0394318	0.0890226
P05164	Myeloperoxidase OS=Homo sapiens GN=MPO PE=1 SV=1 - [PERM_HUMAN]	24.83	14	17	29	745	83.81484	8.968262	1.07118	1.09772	1.10663	1.0918438	0.0131756	0.0513818
B4DNG2	cDNA FLJ59357, highly similar to Probable ATP-dependent RNA helicase DDX5 (EC 3.6.1.-) OS=Homo sapiens PE=2 SV=1 - [B4DNG2_HUMAN]	15.44	4	8	11	544	61.47789	8.851074	1.03882	1.15393	1.0826	1.0917829	0.111653	0.174732
P14618	Pyruvate kinase PKM OS=Homo sapiens GN=PKM PE=1 SV=4 - [KPYM_HUMAN]	65.91	4	35	80	531	57.90003	7.840332	1.11682	1.08403	1.07288	1.0912449	0.0202591	0.0623036
B4DY46	cDNA FLJ53447, highly similar to Syntaxin-binding protein 2 OS=Homo sapiens PE=2 SV=1 - [B4DY46_HUMAN]	10.91	5	5	5	559	62.65955	6.507324	1.09347	1.16992	1.01029	1.0912293	0.186373	0.255414
Q99714	3-hydroxyacyl-CoA dehydrogenase type-2 OS=Homo sapiens GN=HSD17B10 PE=1 SV=3 - [HCD2_HUMAN]	61.69	10	10	13	261	26.90611	7.781738	1.13892	1.01525	1.11949	1.0912229	0.140714	0.2060194
B4E0N6	cDNA FLJ56280, highly similar to Endoplasmic reticulum-Golgi intermediate compartment protein 1 OS=Homo sapiens PE=2 SV=1 - [B4E0N6_HUMAN]	7.66	2	2	2	235	26.24236	8.616699	1.09784	1.13548	1.04012	1.0911438	0.0814242	0.1409236
Q9HC35	Echinoderm microtubule-associated protein-like 4 OS=Homo sapiens GN=EML4 PE=1 SV=3 - [EMAL4_HUMAN]	2.14	2	2	2	981	108.8483	6.404785	1.07414	1.05092	1.14775	1.0909349	0.0894128	0.1502792
B4DPC7	cDNA FLJ55124, highly similar to Plasma serine protease inhibitor OS=Homo sapiens PE=2 SV=1 - [B4DPC7_HUMAN]	9.45	2	3	5	328	37.17031	8.98291	1.09996	0.991944	1.18069	1.0908641	0.238429	0.3073428

Q13243	Serine/arginine-rich splicing factor 5 OS=Homo sapiens GN=SRSF5 PE=1 SV=1 - [SRSF5_HUMAN]	17.28	3	4	5	272	31.24529	11.59033	1.14784	1.06543	1.05913	1.0907993	0.0864109	0.1468675
Q5HYL6	Putative uncharacterized protein DKFZp686E1899 OS=Homo sapiens GN=DKFZp686E1899 PE=2 SV=1 - [Q5HYL6_HUMAN]	19.89	4	5	7	352	39.53023	5.186035	1.01602	1.09668	1.1592	1.0906336	0.160287	0.22762
P31942	Heterogeneous nuclear ribonucleoprotein H3 OS=Homo sapiens GN=HNRNPH3 PE=1 SV=2 - [HNRH3_HUMAN]	18.79	4	5	7	346	36.90308	6.873535	1.05033	1.13259	1.08879	1.0905686	0.0624505	0.1171668
Q9Y277	Voltage-dependent anion-selective channel protein 3 OS=Homo sapiens GN=VDAC3 PE=1 SV=1 - [VDAC3_HUMAN]	16.96	4	5	12	283	30.63929	8.660645	1.08781	1.10046	1.08121	1.089825	0.00392953	0.0390072
A0A087WUQ6	Glutathione peroxidase OS=Homo sapiens GN=GPX1 PE=1 SV=1 - [A0A087WUQ6_HUMAN]	52.48	8	8	11	202	21.92416	6.55127	1.07929	1.05754	1.13235	1.0897303	0.0561896	0.1103259
B3KML1	cDNA FLJ11308 fis, clone PLACE1010074, highly similar to Sorting nexin-2 OS=Homo sapiens PE=2 SV=1 - [B3KML1_HUMAN]	18.11	6	9	14	519	58.43561	5.059082	1.01825	1.17364	1.07591	1.0892663	0.187875	0.2570561
Q08211	ATP-dependent RNA helicase A OS=Homo sapiens GN=DHX9 PE=1 SV=4 - [DHX9_HUMAN]	14.88	17	17	23	1270	140.8691	6.844238	1.08354	1.09538	1.08841	1.0891122	0.00148194	0.0290552
E7ENN3	Nesprin-1 OS=Homo sapiens GN=SYNE1 PE=1 SV=2 - [E7ENN3_HUMAN]	0.62	4	4	4	8392	964.2411	5.541504	1.1008	1.09924	1.0664	1.0888129	0.0155689	0.0547825
P13073	Cytochrome c oxidase subunit 4 isoform 1, mitochondrial OS=Homo sapiens GN=COX4I1 PE=1 SV=1 - [COX41_HUMAN]	24.26	4	4	9	169	19.56405	9.510254	1.14815	1.08071	1.03667	1.0885107	0.112038	0.1750796
Q9UMS4	Pre-mRNA-processing factor 19 OS=Homo sapiens GN=PRPF19 PE=1 SV=1 - [PRP19_HUMAN]	5.56	2	2	2	504	55.14635	6.609863	1.11436	1.07812	1.07275	1.0884102	0.0211563	0.063152
Q16698	2,4-dienoyl-CoA reductase, mitochondrial OS=Homo sapiens GN=DECR1 PE=1 SV=1 - [DECR_HUMAN]	25.67	6	6	10	335	36.0448	9.275879	1.07937	1.10769	1.07816	1.0884051	0.0116989	0.0494101

P61204	ADP-ribosylation factor 3 OS=Homo sapiens GN=ARF3 PE=1 SV=2 - [ARF3_HUMAN]	29.28	4	5	15	181	20.58773	7.430176	1.08874	1.07924	1.09434	1.0874412	0.00252919	0.0356721
Q9NZU5	LIM and cysteine-rich domains protein 1 OS=Homo sapiens GN=LMCD1 PE=1 SV=1 - [LMCD1_HUMAN]	18.36	5	5	9	365	40.80608	7.928223	1.13801	0.899369	1.22453	1.0873034	0.463994	0.5334985
B3KR50	cDNA FLJ33691 fis, clone BRAWH2002976, highly similar to GROWTH FACTOR RECEPTOR-BOUND PROTEIN 2 OS=Homo sapiens PE=2 SV=1 - [B3KR50_HUMAN]	31.34	6	6	7	217	25.1784	6.315918	1.16421	1.01169	1.08589	1.0872623	0.186015	0.2550266
A0A024RDA1	Exocyst complex component 1, isoform CRA_a OS=Homo sapiens GN=EXOC1 PE=4 SV=1 - [A0A024RDA1_HUMAN]	3.19	2	2	2	879	100.219	6.595215	1.048	1.10928	1.10444	1.0872401	0.0472495	0.0991979
B4DY09	Interleukin enhancer-binding factor 2 OS=Homo sapiens GN=ILF2 PE=1 SV=1 - [B4DY09_HUMAN]	25.57	7	7	12	352	38.88615	4.944824	1.05008	1.15909	1.0517	1.0869553	0.137454	0.2023844
Q9NUJ1	Mycophenolic acid acyl-glucuronide esterase, mitochondrial OS=Homo sapiens GN=ABHD10 PE=1 SV=1 - [ABHDA_HUMAN]	7.84	2	2	2	306	33.91075	8.572754	1.07146	1.08041	1.10883	1.0868997	0.0163927	0.0563479
P04839	Cytochrome b-245 heavy chain OS=Homo sapiens GN=CYBB PE=1 SV=2 - [CY24B_HUMAN]	8.77	5	5	5	570	65.29359	8.631348	1.14017	1.07392	1.04637	1.0868189	0.0892483	0.1501518
Q6IPN0	Reticulon OS=Homo sapiens GN=RTN4 PE=2 SV=1 - [Q6IPN0_HUMAN]	17.2	5	5	11	343	36.89529	4.805176	1.10339	1.0541	1.10199	1.0864952	0.0333368	0.0806036
Q70T18	BBF2H7/FUS protein (Fragment) OS=Homo sapiens PE=2 SV=1 - [Q70T18_HUMAN]	23.57	3	3	4	157	16.09679	8.997559	0.993599	1.13039	1.13394	1.0859751	0.203813	0.2740156
D6R9W4	Drebrin (Fragment) OS=Homo sapiens GN=DBN1 PE=1 SV=1 - [D6R9W4_HUMAN]	14.83	3	3	4	317	36.449	5.135254	0.950756	0.901297	1.40363	1.0852284	0.647208	0.7047082
P46778	60S ribosomal protein L21 OS=Homo sapiens GN=RPL21 PE=1 SV=2 - [RL21_HUMAN]	19.38	4	4	5	160	18.55306	10.4917	1.08642	1.06127	1.10778	1.0851579	0.0240182	0.0675863
B4DG62	Hexokinase OS=Homo sapiens PE=2 SV=1 - [B4DG62_HUMAN]	15.52	14	14	16	915	102.2461	6.800293	1.06167	1.11335	1.08034	1.0851236	0.0300893	0.0767538

A0A087WWB6	Transgelin OS=Homo sapiens GN=TAGLN3 PE=1 SV=1 - [A0A087WWB6_HUMAN]	16.29	2	3	4	221	24.99449	8.411621	0.99358	0.999288	1.2623	1.0850557	0.438516	0.5097447
P40925	Malate dehydrogenase, cytoplasmic OS=Homo sapiens GN=MDH1 PE=1 SV=4 - [MDHC_HUMAN]	30.84	9	10	15	334	36.40302	7.356934	1.07886	1.15478	1.0214	1.0850141	0.158713	0.2260175
P06899	Histone H2B type 1-J OS=Homo sapiens GN=HIST1H2BJ PE=1 SV=3 - [H2B1J_HUMAN]	57.94	2	9	33	126	13.89558	10.31592	1.10694	1.03851	1.10749	1.0843122	0.0665155	0.1219154
Q59EE7	Pro-alpha-1 type V collagen variant (Fragment) OS=Homo sapiens PE=2 SV=1 - [Q59EE7_HUMAN]	1.95	4	4	6	1792	178.4071	4.995605	1.08483	1.08833	1.079	1.0840551	0.00104693	0.0276207
A8K590	cDNA FLJ77456, highly similar to Homo sapiens interleukin enhancer binding factor 3, 90kDa (ILF3), transcript variant 2, mRNA OS=Homo sapiens PE=2 SV=1 - [A8K590_HUMAN]	12.68	7	7	16	702	76.00271	7.752441	1.11973	1.07247	1.05935	1.0838515	0.0446382	0.0957603
B4DUD6	cDNA FLJ54551, highly similar to Septin-7 OS=Homo sapiens PE=2 SV=1 - [B4DUD6_HUMAN]	21.95	9	10	19	410	47.85066	8.733887	1.09085	1.11829	1.04164	1.0835943	0.0650066	0.1201788
P50991	T-complex protein 1 subunit delta OS=Homo sapiens GN=CCT4 PE=1 SV=4 - [TCPD_HUMAN]	35.62	13	14	17	539	57.88776	7.825684	1.11806	1.06622	1.06573	1.0833325	0.0407752	0.0909849
P49748	Very long-chain specific acyl-CoA dehydrogenase, mitochondrial OS=Homo sapiens GN=ACADVL PE=1 SV=1 - [ACADV_HUMAN]	26.72	7	14	16	655	70.34543	8.748535	1.14321	1.06368	1.04247	1.0831188	0.113401	0.1768136
A0A0H4PIN7	Dendritic cell-specific intercellular adhesion molecule-3-grabbing non-integrin (Fragment) OS=Homo sapiens PE=4 SV=1 - [A0A0H4PIN7_HUMAN]	39.15	2	2	2	189	21.51455	5.554199	1.07384	1.08863	1.08646	1.0829766	0.00307646	0.0360868
A0A087X253	AP-2 complex subunit beta OS=Homo sapiens GN=AP2B1 PE=1 SV=1 - [A0A087X253_HUMAN]	18.73	8	16	25	913	101.2684	5.287598	1.0255	1.1548	1.06349	1.0812628	0.168361	0.2362778
O00483	Cytochrome c oxidase subunit NDUFA4 OS=Homo sapiens GN=NDUFA4 PE=1 SV=1 - [NDUA4_HUMAN]	27.16	2	2	2	81	9.363911	9.378418	1.08441	1.10354	1.05239	1.080113	0.0329917	0.0802733

Biology Open • Supplementary information

B7Z230	cDNA FLJ53833, highly similar to FK506-binding protein 9 (EC 5.2.1.8) OS=Homo sapiens PE=2 SV=1 - [B7Z230_HUMAN]	9.03	3	3	3	432	47.77867	4.906738	1.11284	1.15305	0.972795	1.0795619	0.282588	0.3554665
Q05DJ8	HTRA1 protein (Fragment) OS=Homo sapiens GN=HTRA1 PE=2 SV=1 - [Q05DJ8_HUMAN]	11.01	5	5	5	445	48.00659	9.788574	0.987382	1.11692	1.13416	1.0794849	0.228295	0.2981762
B3KN10	cDNA FLJ13176 fis, clone NT2RP3003846, highly similar to SEC23-interacting protein OS=Homo sapiens PE=2 SV=1 - [B3KN10_HUMAN]	5.32	2	2	3	376	42.53053	5.059082	1.08418	1.12825	1.02591	1.079445	0.115546	0.1795791
Q6UW02	Cytochrome P450 20A1 OS=Homo sapiens GN=CYP20A1 PE=1 SV=1 - [CP20A_HUMAN]	6.28	3	3	3	462	52.39902	6.214355	1.08145	1.11806	1.03809	1.0792012	0.0756222	0.1337395
Q6DD88	Atlastin-3 OS=Homo sapiens GN=ATL3 PE=1 SV=1 - [ATLA3_HUMAN]	27.17	11	11	15	541	60.50343	5.655762	1.07753	1.07553	1.08295	1.0786728	0.000793038	0.0242397
B3KM52	cDNA FLJ10327 fis, clone NT2RM2000581, highly similar to Transmembrane GTPase MFN2 (EC 3.6.5.-) OS=Homo sapiens PE=2 SV=1 - [B3KM52_HUMAN]	7.1	2	2	2	324	36.89773	7.503418	1.05612	1.1287	1.05087	1.0785624	0.0888059	0.1494818
Q7Z427	Putative MAPK activating protein OS=Homo sapiens PE=2 SV=1 - [Q7Z427_HUMAN]	14.25	3	3	5	358	38.67966	5.312988	1.0798	1.1069	1.04879	1.0784957	0.0428265	0.0935455
P07900	Heat shock protein HSP 90-alpha OS=Homo sapiens GN=HSP90AA1 PE=1 SV=5 - [HS90A_HUMAN]	36.34	19	30	64	732	84.60669	5.020996	1.06452	1.12158	1.04854	1.0782127	0.0717852	0.1288404
P09382	Galectin-1 OS=Homo sapiens GN=LGALS1 PE=1 SV=2 - [LEG1_HUMAN]	58.52	8	8	42	135	14.7062	5.503418	1.07446	1.08375	1.07629	1.0781639	0.00131903	0.0283421
A0A0A0MTR1	Cadherin-13 OS=Homo sapiens GN=CDH13 PE=1 SV=2 - [A0A0A0MTR1_HUMAN]	11.98	7	7	9	701	76.92116	4.95752	1.13023	1.05996	1.04382	1.0780004	0.0988024	0.1609044
O75935	Dynactin subunit 3 OS=Homo sapiens GN=DCTN3 PE=1 SV=1 - [DCTN3_HUMAN]	14.52	3	3	3	186	21.10625	5.465332	1.12639	1.03778	1.06891	1.0776936	0.0958265	0.1573082
B4DLP1	cDNA FLJ52560 OS=Homo sapiens PE=2 SV=1 - [B4DLP1_HUMAN]	13.8	5	5	7	355	38.77395	5.338379	1.12131	1.08535	1.02532	1.0773238	0.109916	0.1729733

Biology Open • Supplementary information

B7Z5L6	cDNA FLJ54723, highly similar to Poly (ADP-ribose) polymerase 9 (EC 2.4.2.30) OS=Homo sapiens PE=2 SV=1 - [B7Z5L6_HUMAN]	6.18	4	4	5	777	87.49412	8.147949	1.05956	1.15142	1.02099	1.0773224	0.183663	0.252518
O00629	Importin subunit alpha-3 OS=Homo sapiens GN=KPNA4 PE=1 SV=1 - [IMA3_HUMAN]	8.64	3	3	3	521	57.85064	4.95752	1.04184	1.11035	1.079	1.0770651	0.0601201	0.1148457
Q567R0	UQCRH protein OS=Homo sapiens GN=UQCRH PE=2 SV=1 - [Q567R0_HUMAN]	30.59	2	2	3	85	9.946829	5.452637	1.02644	1.0941	1.11056	1.0770319	0.095872	0.1573082
Q59H95	Flightless I homolog variant (Fragment) OS=Homo sapiens PE=2 SV=1 - [Q59H95_HUMAN]	2.36	2	2	2	1101	125.1981	5.262207	1.06503	1.13035	1.03544	1.0769378	0.111095	0.1741809
Q9Y315	Deoxyribose-phosphate aldolase OS=Homo sapiens GN=DERA PE=1 SV=2 - [DEOC_HUMAN]	11.64	3	3	3	318	35.20848	8.938965	1.0044	1.08443	1.14197	1.0769348	0.193566	0.2628243
Q15046	Lysine--tRNA ligase OS=Homo sapiens GN=KARS PE=1 SV=3 - [SYK_HUMAN]	9.21	5	5	7	597	68.0046	6.354004	1.04621	1.09132	1.09312	1.0768834	0.0376022	0.0868829
Q9P0K7	Ankycorbin OS=Homo sapiens GN=RAI14 PE=1 SV=2 - [RAI14_HUMAN]	6.43	4	4	4	980	109.9733	6.214355	1.12349	1.09276	1.01439	1.0768809	0.141546	0.206969
Q9Y394	Dehydrogenase/reductase SDR family member 7 OS=Homo sapiens GN=DHRS7 PE=1 SV=1 - [DHRS7_HUMAN]	6.19	2	2	3	339	38.27412	8.32373	1.05593	1.10926	1.06424	1.0764764	0.0438713	0.0947699
P18859	ATP synthase-coupling factor 6, mitochondrial OS=Homo sapiens GN=ATP5J PE=1 SV=1 - [ATP5J_HUMAN]	32.41	3	3	3	108	12.5796	9.524902	1.17078	1.02983	1.02719	1.0759327	0.250537	0.3207304
P49903	Selenide, water dikinase 1 OS=Homo sapiens GN=SEPHS1 PE=1 SV=2 - [SPS1_HUMAN]	7.14	2	2	3	392	42.88266	5.973145	1.07057	1.23547	0.9212	1.0757482	0.491754	0.5606073
P02671	Fibrinogen alpha chain OS=Homo sapiens GN=FGA PE=1 SV=2 - [FIBA_HUMAN]	32.1	31	31	105	866	94.91442	6.01123	1.06101	1.07967	1.08535	1.0753463	0.00938684	0.0472949
P14550	Alcohol dehydrogenase [NADP(+)] OS=Homo sapiens GN=AKR1A1 PE=1 SV=3 - [AK1A1_HUMAN]	46.15	11	12	16	325	36.54986	6.785645	1.09685	1.08912	1.03992	1.0752975	0.0517446	0.1043663

Biology Open • Supplementary information

B7Z2V8	cDNA FLJ54958, highly similar to Rap1 GTPase-GDP dissociation stimulator 1 OS=Homo sapiens PE=2 SV=1 - [B7Z2V8_HUMAN]	8.41	4	4	4	559	60.99803	5.439941	1.06157	1.01595	1.1483	1.0752747	0.192029	0.2610515
Q15582	Transforming growth factor-beta-induced protein ig-h3 OS=Homo sapiens GN=TGFBI PE=1 SV=1 - [BGH3_HUMAN]	32.06	20	20	61	683	74.63398	7.708496	1.08069	1.07379	1.07119	1.0752241	0.00141689	0.0287701
A0A087WTJ2	Protein GIMAP1-GIMAP5 OS=Homo sapiens GN=GIMAP1-GIMAP5 PE=4 SV=1 - [A0A087WTJ2_HUMAN]	9	3	3	3	511	57.0162	8.440918	1.06	1.12165	1.04378	1.0751438	0.0868437	0.1473513
B3KX11	T-complex protein 1 subunit gamma OS=Homo sapiens PE=2 SV=1 - [B3KX11_HUMAN]	30.46	13	13	19	522	57.90903	6.932129	1.09556	1.11343	1.01631	1.0750969	0.128238	0.193529
Q9H3K6	BolA-like protein 2 OS=Homo sapiens GN=BOLA2 PE=1 SV=1 - [BOLA2_HUMAN]	26.74	2	2	4	86	10.11018	6.521973	1.15353	1.0194	1.05166	1.0748648	0.205216	0.2748936
A0A0C4DFX3	EMILIN-1 OS=Homo sapiens GN=EMILIN1 PE=1 SV=1 - [A0A0C4DFX3_HUMAN]	25.1	20	20	41	1016	106.6295	5.17334	1.0668	1.09701	1.06058	1.0747965	0.0218848	0.0642498
Q53S54	Putative uncharacterized protein CUL3 (Fragment) OS=Homo sapiens GN=CUL3 PE=3 SV=1 - [Q53S54_HUMAN]	3.49	3	3	4	746	86.41116	7.986816	1.12427	0.988804	1.11119	1.0747559	0.225269	0.2953275
B4DQY1	cDNA FLJ56133, highly similar to Serine/threonine-protein phosphatase 2A 65 kDa regulatory subunit A alpha isoform OS=Homo sapiens PE=2 SV=1 - [B4DQY1_HUMAN]	14.23	7	7	11	555	61.48193	5.122559	1.05863	1.08321	1.08132	1.0743868	0.0110903	0.0493307
Q96AC1	Fermitin family homolog 2 OS=Homo sapiens GN=FERMT2 PE=1 SV=1 - [FERM2_HUMAN]	31.91	18	18	26	680	77.81061	6.697754	1.05032	1.14079	1.03203	1.0743808	0.157441	0.2249383
O75396	Vesicle-trafficking protein SEC22b OS=Homo sapiens GN=SEC22B PE=1 SV=4 - [SC22B_HUMAN]	38.14	7	7	14	215	24.57753	6.91748	1.03545	1.10333	1.08351	1.0740975	0.0666591	0.1220716
B2RE46	cDNA, FLJ96923, highly similar to Homo sapiens ribophorin II (RPN2), mRNA OS=Homo sapiens PE=2 SV=1 - [B2RE46_HUMAN]	25.67	11	11	16	631	69.29107	5.782715	1.05607	1.10743	1.05823	1.0739103	0.0478402	0.0996284
Q86UE4	Protein LYRIC OS=Homo sapiens GN=MTDH PE=1 SV=2 -	11.68	6	6	6	582	63.79881	9.319824	1.11835	0.982266	1.12074	1.0737851	0.248229	0.3182785

	[LYRIC_HUMAN]													
O95373	Importin-7 OS=Homo sapiens GN=IPO7 PE=1 SV=1 - [IPO7_HUMAN]	5.11	4	4	5	1038	119.4399	4.817871	1.03856	1.1476	1.03474	1.0736322	0.18487	0.2537652
P47755	F-actin-capping protein subunit alpha-2 OS=Homo sapiens GN=CAPZA2 PE=1 SV=3 - [CAZA2_HUMAN]	43.01	7	8	12	286	32.92861	5.846191	1.09312	1.07321	1.05345	1.0732616	0.0235808	0.06674
A1X283	SH3 and PX domain-containing protein 2B OS=Homo sapiens GN=SH3PXD2B PE=1 SV=3 - [SPD2B_HUMAN]	3.29	3	3	3	911	101.5161	8.689941	1.0899	1.04111	1.08866	1.0732226	0.0449045	0.0961428
A0A024R4X0	NADH-cytochrome b5 reductase OS=Homo sapiens GN=CYB5R3 PE=3 SV=1 - [A0A024R4X0_HUMAN]	44.33	10	10	22	291	33.21724	7.57666	1.08862	1.07554	1.05428	1.0728147	0.0183738	0.0594602
Q5D1D5	Guanylate binding protein 1 OS=Homo sapiens PE=2 SV=1 - [Q5D1D5_HUMAN]	15.71	9	9	11	592	67.8737	6.315918	1.07889	1.09845	1.04015	1.0724975	0.0515534	0.1041382
H3BSW3	Adenine phosphoribosyltransferase OS=Homo sapiens GN=APRT PE=1 SV=1 - [H3BSW3_HUMAN]	41.27	2	2	3	63	6.914693	8.748535	1.15682	1.08741	0.972213	1.0721449	0.312156	0.3847954
B2R6K4	cDNA, FLJ92996, highly similar to Homo sapiens guanine nucleotide binding protein (G protein), beta polypeptide 1 (GNB1), mRNA OS=Homo sapiens PE=2 SV=1 - [B2R6K4_HUMAN]	24.71	3	7	12	340	37.27704	6.17627	1.0463	1.15198	1.01809	1.0721272	0.218745	0.2883379
A0A0A0MTC7	Laminin subunit alpha-4 OS=Homo sapiens GN=LAMA4 PE=1 SV=1 - [A0A0A0MTC7_HUMAN]	15.69	20	20	35	1816	201.6933	6.290527	1.02445	1.09488	1.09633	1.0718873	0.0938156	0.1549858
K7EIN4	Transmembrane emp24 domain-containing protein 1 OS=Homo sapiens GN=TMED1 PE=1 SV=1 - [K7EIN4_HUMAN]	21.95	2	2	2	82	9.629161	8.968262	1.15966	1.06105	0.993633	1.0714456	0.276533	0.3490172
Q96IR1	RPS4X protein (Fragment) OS=Homo sapiens GN=RPS4X PE=2 SV=2 - [Q96IR1_HUMAN]	35.39	4	7	13	243	27.24272	9.935059	1.07844	1.08028	1.05536	1.0713591	0.0123852	0.0504499
A8K4G7	cDNA FLJ78528, highly similar to Homo sapiens vacuolar protein sorting 4B (yeast) (VPS4B), mRNA OS=Homo sapiens	15.54	3	5	5	444	49.24243	6.756348	0.98661	1.20571	1.02161	1.07131	0.40411	0.4764585

Biology Open • Supplementary information

	PE=2 SV=1 - [A8K4G7_HUMAN]													
H0YNG3	Signal peptidase complex catalytic subunit SEC11 OS=Homo sapiens GN=SEC11A PE=1 SV=1 - [H0YNG3_HUMAN]	22.09	4	4	4	163	18.63887	9.554199	1.13623	0.979794	1.09759	1.0712054	0.269333	0.3413299
Q5U071	High-mobility group box 2 OS=Homo sapiens PE=2 SV=1 - [Q5U071_HUMAN]	17.31	2	3	8	208	23.88968	8.133301	1.01845	1.18529	1.00965	1.071134	0.339226	0.4129018
Q15404	Ras suppressor protein 1 OS=Homo sapiens GN=RSU1 PE=1 SV=3 - [RSU1_HUMAN]	35.38	9	9	21	277	31.52069	8.645996	1.08466	1.09732	1.02888	1.0702867	0.0790184	0.1377329
B4DM33	cDNA FLJ52068, highly similar to Microtubule-associated protein RP/EB family member 1 OS=Homo sapiens PE=2 SV=1 - [B4DM33_HUMAN]	33.19	5	6	11	238	26.56558	5.287598	1.03675	1.09904	1.07496	1.0702508	0.0606422	0.1153342
P31946	14-3-3 protein beta/alpha OS=Homo sapiens GN=YWHAB PE=1 SV=3 - [1433B_HUMAN]	51.22	5	11	42	246	28.06483	4.830566	1.07784	1.08894	1.04382	1.0701969	0.0354161	0.0833458
A0A024R5A3	Calpain 1, (Mu/I) large subunit, isoform CRA_b OS=Homo sapiens GN=CAPN1 PE=4 SV=1 - [A0A024R5A3_HUMAN]	18.45	12	12	16	710	81.41598	5.744629	1.03718	1.12519	1.04796	1.0701075	0.127153	0.1922843
B4DJS7	Sorting nexin 6, isoform CRA_e OS=Homo sapiens GN=SNX6 PE=2 SV=1 - [B4DJS7_HUMAN]	25.85	7	7	9	294	33.79665	8.572754	1.06643	1.11346	1.03022	1.0700367	0.100783	0.1630008
A0A0A0MS41	Sideroflexin OS=Homo sapiens GN=SFXN3 PE=1 SV=1 - [A0A0A0MS41_HUMAN]	26.79	7	7	10	321	35.48053	9.100098	1.11861	1.06482	1.02528	1.0695725	0.123709	0.1890542
P56381	ATP synthase subunit epsilon, mitochondrial OS=Homo sapiens GN=ATP5E PE=1 SV=2 - [ATP5E_HUMAN]	29.41	2	2	2	51	5.776108	9.92041	1.08802	1.1008	1.01973	1.069515	0.109875	0.1729733
Q9Y5Z4	Heme-binding protein 2 OS=Homo sapiens GN=HEBP2 PE=1 SV=1 - [HEBP2_HUMAN]	23.41	5	5	8	205	22.86119	4.627441	1.03928	1.12558	1.04286	1.0692421	0.133358	0.198687
A0A024R7S3	Clathrin, light polypeptide (Lcb), isoform CRA_c OS=Homo sapiens GN=CLTB PE=4 SV=1 - [A0A024R7S3_HUMAN]	20.38	5	5	7	211	23.16714	4.690918	1.06767	1.11594	1.0241	1.0692374	0.120756	0.1854701
E5RHW4	Erlin-2 (Fragment) OS=Homo sapiens GN=ERLIN2 PE=1	19.82	6	6	7	338	37.70141	5.617676	1.00938	1.09404	1.1034	1.0689392	0.147608	0.2138011

Biology Open • Supplementary information

	SV=1 - [E5RHW4_HUMAN]														
E9PM69	26S protease regulatory subunit 6A OS=Homo sapiens GN=PSMC3 PE=1 SV=1 - [E9PM69_HUMAN]	28.21	10	10	14	397	44.29577	5.046387	1.06065	1.12573	1.02027	1.0688833	0.154194	0.2214583
Q86UP2	Kinectin OS=Homo sapiens GN=KTN1 PE=1 SV=1 - [KTN1_HUMAN]	9.43	11	12	13	1357	156.1793	5.643066	1.08923	1.09769	1.01865	1.0685236	0.111728	0.1747686
Q9BS26	Endoplasmic reticulum resident protein 44 OS=Homo sapiens GN=ERP44 PE=1 SV=1 - [ERP44_HUMAN]	22.66	7	7	12	406	46.9414	5.262207	1.12905	1.08431	0.991169	1.0681757	0.23522	0.3043021
Q6FID4	SARA1 protein OS=Homo sapiens GN=SARA1 PE=2 SV=1 - [Q6FID4_HUMAN]	27.27	4	4	7	198	22.32355	7.034668	1.16684	1.09592	0.941576	1.0681127	0.413421	0.4857446
P62888	60S ribosomal protein L30 OS=Homo sapiens GN=RPL30 PE=1 SV=2 - [RL30_HUMAN]	54.78	5	6	8	115	12.77574	9.627441	1.08563	1.07865	1.03989	1.0680564	0.0410332	0.0915003
B1AJY5	26S proteasome non-ATPase regulatory subunit 10 OS=Homo sapiens GN=PSMD10 PE=1 SV=1 - [B1AJY5_HUMAN]	22.7	3	3	3	185	20.20421	5.57959	1.0265	1.18661	0.990997	1.0680377	0.375497	0.4486613
Q5HYD9	Putative uncharacterized protein DKFZp686M0619 (Fragment) OS=Homo sapiens GN=DKFZp686M0619 PE=2 SV=1 - [Q5HYD9_HUMAN]	19.63	2	2	3	107	11.86578	4.79248	1.08008	1.08435	1.03944	1.0679558	0.0416064	0.092111
Q14677	Clathrin interactor 1 OS=Homo sapiens GN=CLINT1 PE=1 SV=1 - [EPN4_HUMAN]	3.52	2	2	2	625	68.2158	6.41748	1.01598	1.10209	1.08573	1.0679349	0.12365	0.1890494
P78527	DNA-dependent protein kinase catalytic subunit OS=Homo sapiens GN=PRKDC PE=1 SV=3 - [PRKDC_HUMAN]	7.27	23	24	32	4128	468.7879	7.122559	1.09836	1.05229	1.05122	1.0672887	0.0494116	0.1017576
P23368	NAD-dependent malic enzyme, mitochondrial OS=Homo sapiens GN=ME2 PE=1 SV=1 - [MAOM_HUMAN]	9.25	4	4	4	584	65.40178	7.605957	1.1951	0.995677	1.01083	1.0672026	0.404468	0.4767146
A0A087WTH0	Enolase-phosphatase E1 OS=Homo sapiens GN=ENOPH1 PE=1 SV=2 - [A0A087WTH0_HUMAN]	11.59	2	2	2	207	23.34971	5.097168	1.05443	1.04644	1.10003	1.0669668	0.056886	0.1113058

Biology Open • Supplementary information

B4DW07	cDNA FLJ50032, highly similar to Homo sapiens spectrin domain with coiled-coils 1 (SPECC1), transcript variant, mRNA OS=Homo sapiens PE=2 SV=1 - [B4DW07_HUMAN]	6.62	2	2	2	408	45.70269	8.338379	1.0179	1.07896	1.10337	1.0667436	0.119586	0.1839158
P55327	Tumor protein D52 OS=Homo sapiens GN=TPD52 PE=1 SV=2 - [TPD52_HUMAN]	12.95	2	2	3	224	24.31224	4.830566	1.16727	1.02144	1.01071	1.0664738	0.318616	0.3919023
B4DF77	Phosphofurin acidic cluster sorting protein 1 OS=Homo sapiens GN=PACS1 PE=1 SV=1 - [B4DF77_HUMAN]	10.22	4	4	4	499	54.42693	9.129395	1.08413	1.06792	1.04699	1.0663455	0.0252712	0.0697741
Q14554	Protein disulfide-isomerase A5 OS=Homo sapiens GN=PDIA5 PE=1 SV=1 - [PDIA5_HUMAN]	7.13	4	4	4	519	59.55611	7.913574	1.09311	1.10301	1.00272	1.0662793	0.173383	0.2410273
O75367	Core histone macro-H2A.1 OS=Homo sapiens GN=H2AFY PE=1 SV=4 - [H2AY_HUMAN]	37.1	12	13	25	372	39.59247	9.788574	1.08543	1.03587	1.07703	1.0661111	0.0496888	0.1018392
P07814	Bifunctional glutamate/proline--tRNA ligase OS=Homo sapiens GN=EPRS PE=1 SV=5 - [SYEP_HUMAN]	7.94	8	8	9	1512	170.4831	7.327637	1.08846	1.02578	1.08366	1.0659624	0.081936	0.1414347
P18124	60S ribosomal protein L7 OS=Homo sapiens GN=RPL7 PE=1 SV=1 - [RL7_HUMAN]	22.58	5	5	7	248	29.2072	10.65283	1.05061	1.12024	1.02678	1.0658732	0.143239	0.2090329
B7Z8G2	cDNA FLJ58213, highly similar to FK506-binding protein 5 (EC 5.2.1.8) OS=Homo sapiens PE=2 SV=1 - [B7Z8G2_HUMAN]	15.48	5	5	5	420	47.27253	5.871582	1.09148	1.0911	1.01403	1.0655344	0.125927	0.1909841
B4DEA3	cDNA FLJ56531, highly similar to UV excision repair protein RAD23 homolog B OS=Homo sapiens PE=2 SV=1 - [B4DEA3_HUMAN]	19.35	7	8	13	403	42.28336	5.427246	1.05465	1.06241	1.07944	1.0654987	0.0122654	0.0502036
B4DLB0	Upstream binding transcription factor, RNA polymerase I, isoform CRA_a OS=Homo sapiens GN=UBTF PE=2 SV=1 - [B4DLB0_HUMAN]	5.74	2	2	2	505	58.31619	4.995605	1.06673	0.998232	1.13072	1.0652289	0.230284	0.2995816
Q10567	AP-1 complex subunit beta-1 OS=Homo sapiens GN=AP1B1 PE=1 SV=2 - [AP1B1_HUMAN]	14.75	4	12	18	949	104.5701	5.059082	1.08984	1.10454	1.00096	1.065113	0.181835	0.2505134

Biology Open • Supplementary information

Q7L014	Probable ATP-dependent RNA helicase DDX46 OS=Homo sapiens GN=DDX46 PE=1 SV=2 - [DDX46_HUMAN]	3.1	3	3	3	1031	117.2895	9.290527	1.05041	1.19919	0.945293	1.064966	0.470803	0.5404097
A0A0C4DH07	Latent-transforming growth factor beta-binding protein 4 OS=Homo sapiens GN=LTBP4 PE=1 SV=1 - [A0A0C4DH07_HUMAN]	10.27	11	11	17	1587	169.3437	5.287598	1.10668	1.04381	1.04407	1.0648535	0.0901353	0.1510436
Q9UFN0	Protein NipSnap homolog 3A OS=Homo sapiens GN=NIPSNAP3A PE=1 SV=2 - [NPS3A_HUMAN]	21.05	4	4	4	247	28.44854	9.158691	1.07246	1.08938	1.03264	1.0648252	0.0611887	0.1157763
K7X1S0	SMAC-epsilon OS=Homo sapiens GN=DIABLO PE=2 SV=1 - [K7X1S0_HUMAN]	16.2	2	2	2	142	16.30402	4.754395	0.978312	1.10736	1.10843	1.0647013	0.272882	0.3451815
L0R6Q1	Alternative protein SLC35A4 OS=Homo sapiens GN=SLC35A4 PE=4 SV=1 - [L0R6Q1_HUMAN]	14.56	2	2	2	103	11.12589	8.104004	1.05364	1.09466	1.0456	1.0646322	0.0510558	0.1034254
P52943	Cysteine-rich protein 2 OS=Homo sapiens GN=CRIP2 PE=1 SV=1 - [CRIP2_HUMAN]	38.94	5	5	11	208	22.478	8.719238	1.1454	1.00478	1.04331	1.0644949	0.264029	0.3352292
Q8WX93	Palladin OS=Homo sapiens GN=PALLD PE=1 SV=3 - [PALLD_HUMAN]	9.54	12	12	22	1383	150.4702	7.093262	1.04389	1.11168	1.0376	1.0643911	0.113086	0.1764037
A8MXL6	Protein SEC13 homolog OS=Homo sapiens GN=SEC13 PE=1 SV=1 - [A8MXL6_HUMAN]	9.86	2	2	2	284	31.5753	5.820801	0.925299	1.13731	1.13048	1.0643615	0.452502	0.5227711
I1SRC5	UBE2L3/KRAS fusion protein OS=Homo sapiens PE=2 SV=1 - [I1SRC5_HUMAN]	26.01	3	6	8	296	33.96148	8.104004	1.07339	1.10885	1.01072	1.0643204	0.154203	0.2214583
A4D2D2	Procollagen C-endopeptidase enhancer OS=Homo sapiens GN=PCOLCE PE=4 SV=1 - [A4D2D2_HUMAN]	28.29	8	8	13	449	47.92797	7.430176	1.15969	0.982563	1.05031	1.0641872	0.339541	0.4131367
B4DU69	cDNA FLJ56583, highly similar to Argininosuccinate lyase (EC 4.3.2.1) OS=Homo sapiens PE=2 SV=1 - [B4DU69_HUMAN]	16.13	3	3	3	217	24.54978	8.470215	1.03353	0.991596	1.16679	1.0639726	0.349485	0.4229556
O15173	Membrane-associated progesterone receptor component 2 OS=Homo sapiens GN=PGRMC2 PE=1 SV=1 - [PGRC2_HUMAN]	15.7	4	4	6	223	23.80373	4.881348	1.06672	1.10755	1.01507	1.0631109	0.142355	0.2079722

Biology Open • Supplementary information

P02679	Fibrinogen gamma chain OS=Homo sapiens GN=FGG PE=1 SV=3 - [FIBG_HUMAN]	53.2	3	20	86	453	51.47887	5.617676	1.03595	1.10546	1.04534	1.0622509	0.103668	0.1663898
B4DM22	cDNA FLJ53357, highly similar to 26S proteasome non-ATPase regulatory subunit 2 OS=Homo sapiens PE=2 SV=1 - [B4DM22_HUMAN]	12	8	8	9	900	99.25032	5.160645	1.02873	1.08576	1.07184	1.0621097	0.0686376	0.1247119
Q9NPJ3	Acyl-coenzyme A thioesterase 13 OS=Homo sapiens GN=ACOT13 PE=1 SV=1 - [ACO13_HUMAN]	15.71	2	2	3	140	14.95086	9.144043	1.08916	1.11203	0.984636	1.0619419	0.254957	0.3253032
B4DIX4	cDNA FLJ61283, highly similar to Pleckstrin homology-like domain family B member1 OS=Homo sapiens PE=2 SV=1 - [B4DIX4_HUMAN]	8.04	2	2	2	373	38.68255	8.543457	1.03436	1.13345	1.0174	1.0617368	0.230135	0.2995315
P10606	Cytochrome c oxidase subunit 5B, mitochondrial OS=Homo sapiens GN=COX5B PE=1 SV=2 - [COX5B_HUMAN]	31.01	6	6	8	129	13.68694	8.807129	1.05885	1.11162	1.01424	1.0615725	0.160196	0.22762
E5KN59	Peptidyl-prolyl cis-trans isomerase D OS=Homo sapiens PE=4 SV=1 - [E5KN59_HUMAN]	4.59	2	2	2	370	40.7379	7.210449	1.05562	1.07711	1.05079	1.0611741	0.0170344	0.0572932
Q9Y5K6	CD2-associated protein OS=Homo sapiens GN=CD2AP PE=1 SV=1 - [CD2AP_HUMAN]	4.54	3	3	4	639	71.40733	6.404785	1.06199	1.09545	1.02593	1.0611229	0.0930468	0.154213
B0AZQ4	Structural maintenance of chromosomes protein OS=Homo sapiens PE=2 SV=1 - [B0AZQ4_HUMAN]	5.26	4	4	4	1217	141.4418	7.181152	1.04642	1.10974	1.02708	1.0610808	0.134184	0.1992162
Q562R1	Beta-actin-like protein 2 OS=Homo sapiens GN=ACTBL2 PE=1 SV=2 - [ACTBL_HUMAN]	22.34	3	8	131	376	41.97597	5.592285	1.09801	1.03616	1.04896	1.0610424	0.0835825	0.1435448
O76003	Glutaredoxin-3 OS=Homo sapiens GN=GLRX3 PE=1 SV=2 - [GLRX3_HUMAN]	7.16	2	2	2	335	37.40823	5.38916	1.15117	1.08076	0.951009	1.0609778	0.407484	0.4797355
B7ZKM7	UACA protein OS=Homo sapiens GN=UACA PE=2 SV=1 - [B7ZKM7_HUMAN]	3.52	3	4	4	1307	150.4693	7.02002	1.0721	1.02043	1.08983	1.060785	0.100021	0.1623842
B4DMT5	Eukaryotic translation initiation factor 3 subunit F OS=Homo sapiens GN=EIF3F PE=2 SV=1 - [B4DMT5_HUMAN]	14.01	3	3	5	307	33.21899	5.592285	1.08151	0.975529	1.1253	1.0607781	0.304981	0.3776739

Biology Open • Supplementary information

A0A096LNZ9	Ubiquitin-like protein ISG15 (Fragment) OS=Homo sapiens GN=ISG15 PE=1 SV=4 - [A0A096LNZ9_HUMAN]	12.59	2	2	2	143	15.55213	6.290527	1.02848	1.10708	1.04561	1.0603884	0.127051	0.192252
B7Z972	Protein-L-isoaspartate O-methyltransferase OS=Homo sapiens PE=2 SV=1 - [B7Z972_HUMAN]	22.92	3	3	3	192	20.66962	6.873535	1.0194	1.07721	1.08448	1.0603632	0.0993031	0.161451
A0A024R8V0	Septin 9, isoform CRA_a OS=Homo sapiens GN=SEPT9 PE=3 SV=1 - [A0A024R8V0_HUMAN]	13.38	7	7	11	568	63.59389	8.396973	0.974853	1.09724	1.10872	1.0602691	0.294715	0.3675793
Q86V81	THO complex subunit 4 OS=Homo sapiens GN=ALYREF PE=1 SV=3 - [THOC4_HUMAN]	11.28	2	2	3	257	26.87162	11.15088	0.967449	1.04927	1.16356	1.0600916	0.401458	0.4739921
Q14008	Cytoskeleton-associated protein 5 OS=Homo sapiens GN=CKAP5 PE=1 SV=3 - [CKAP5_HUMAN]	2.21	4	4	7	2032	225.3518	7.796387	1.04215	1.06291	1.07511	1.060058	0.0247154	0.068914
P26640	Valine--tRNA ligase OS=Homo sapiens GN=VARS PE=1 SV=4 - [SYVC_HUMAN]	7.83	8	8	12	1264	140.3874	7.591309	1.06013	1.03727	1.08163	1.0596761	0.0431007	0.0938359
P10155	60 kDa SS-A/Ro ribonucleoprotein OS=Homo sapiens GN=TROVE2 PE=1 SV=2 - [RO60_HUMAN]	8.36	4	4	5	538	60.63121	8.030762	1.09746	1.04411	1.03732	1.0596283	0.0884281	0.1494069
Q01469	Fatty acid-binding protein, epidermal OS=Homo sapiens GN=FABP5 PE=1 SV=3 - [FABP5_HUMAN]	48.89	6	6	8	135	15.15455	7.005371	1.05355	1.05284	1.07157	1.0593195	0.0104988	0.0489337
A0PJ92	NOP56 protein (Fragment) OS=Homo sapiens GN=NOP56 PE=2 SV=1 - [A0PJ92_HUMAN]	10.29	3	3	6	447	49.7254	9.100098	1.08356	1.0635	1.03045	1.0591706	0.062152	0.1168729
O94874	E3 UFM1-protein ligase 1 OS=Homo sapiens GN=UFL1 PE=1 SV=2 - [UFL1_HUMAN]	7.05	4	4	4	794	89.53976	6.785645	1.17054	0.985113	1.02156	1.0590731	0.407002	0.4795342
Q6P2S0	SRP9 protein OS=Homo sapiens GN=SRP9 PE=2 SV=1 - [Q6P2S0_HUMAN]	38.78	2	2	3	49	5.76486	6.521973	1.10274	1.08461	0.989378	1.0589069	0.235808	0.3047767
G3V1D4	Lin-7 homolog C (C. elegans), isoform CRA_b OS=Homo sapiens GN=LIN7C PE=1 SV=1 - [G3V1D4_HUMAN]	13.29	2	2	2	173	19.17417	9.070801	1.10861	1.02525	1.04273	1.0588649	0.146205	0.212132
A0A087X2E5	Isocitrate dehydrogenase [NAD] subunit, mitochondrial OS=Homo sapiens GN=IDH3B PE=1 SV=1 -	16.49	5	5	6	376	41.19322	8.089355	1.07452	1.02668	1.07506	1.0587535	0.0671121	0.1227307

Biology Open • Supplementary information

	[A0A087X2E5_HUMAN]														
A4QPB0	IQ motif containing GTPase activating protein 1 OS=Homo sapiens GN=IQGAP1 PE=1 SV=1 - [A4QPB0_HUMAN]	31.2	44	46	80	1657	189.1618	6.480957	1.04047	1.0949	1.0404	1.0585883	0.0840834	0.1442587
Q0ZGT2	Nexilin OS=Homo sapiens GN=NEXN PE=1 SV=1 - [NEXN_HUMAN]	10.37	2	6	8	675	80.6087	5.325684	1.05503	1.06275	1.05778	1.0585207	0.00148543	0.0290552
Q9NZM1	Myoferlin OS=Homo sapiens GN=MYOF PE=1 SV=1 - [MYOF_HUMAN]	21.2	36	38	51	2061	234.5606	6.17627	1.07205	1.0572	1.04531	1.0581873	0.017211	0.0576824
H7BY36	RNA-binding protein EWS (Fragment) OS=Homo sapiens GN=EWSR1 PE=1 SV=1 - [H7BY36_HUMAN]	7.47	2	2	2	308	32.1604	9.817871	0.976337	1.17577	1.02178	1.0579614	0.43816	0.509681
K7EP07	Tubulin-folding cofactor B (Fragment) OS=Homo sapiens GN=TBCB PE=1 SV=6 - [K7EP07_HUMAN]	23.08	3	3	4	169	19.23942	5.338379	1.12694	0.94978	1.09677	1.0578318	0.401398	0.4739921
P04792	Heat shock protein beta-1 OS=Homo sapiens GN=HSPB1 PE=1 SV=2 - [HSPB1_HUMAN]	66.83	12	12	40	205	22.76849	6.404785	1.01884	1.07627	1.07738	1.0575	0.0968742	0.1587218
O75116	Rho-associated protein kinase 2 OS=Homo sapiens GN=ROCK2 PE=1 SV=4 - [ROCK2_HUMAN]	6.48	7	7	7	1388	160.7988	6.023926	1.10401	1.0363	1.03011	1.0568094	0.13844	0.203659
B4DVE1	cDNA FLJ53478, highly similar to Galectin-3-binding protein OS=Homo sapiens PE=2 SV=1 - [B4DVE1_HUMAN]	13.26	6	6	12	573	64.05577	5.465332	1.00287	1.14692	1.02042	1.0567339	0.337629	0.4115499
A0A0K0K1H7	Epididymis secretory sperm binding protein Li 284 OS=Homo sapiens GN=HEL-S-284 PE=2 SV=1 - [A0A0K0K1H7_HUMAN]	26.28	17	17	24	780	85.51101	7.723145	1.03378	1.07744	1.05755	1.0562553	0.0468305	0.0985829
E7EVH9	Protein PUDP (Fragment) OS=Homo sapiens GN=PUDP PE=1 SV=1 - [E7EVH9_HUMAN]	14.66	2	2	2	191	21.45206	7.503418	1.03226	1.1178	1.01855	1.0562019	0.212019	0.2817763
B4DE91	cDNA FLJ55534, highly similar to 4-trimethylaminobutyraldehyde dehydrogenase (EC 1.2.1.47) OS=Homo sapiens PE=2 SV=1 - [B4DE91_HUMAN]	16.12	7	7	12	484	52.73549	6.062012	1.0822	1.05734	1.02861	1.0560508	0.0685492	0.1246182

Biology Open • Supplementary information

Q6UVK1	Chondroitin sulfate proteoglycan 4 OS=Homo sapiens GN=CSPG4 PE=1 SV=2 - [CSPG4_HUMAN]	4.61	7	7	7	2322	250.3824	5.465332	1.11126	1.00489	1.05171	1.0559557	0.210706	0.2801413
A0A087WZH7	Myristoylated alanine-rich C-kinase substrate OS=Homo sapiens GN=MARCKS PE=1 SV=1 - [A0A087WZH7_HUMAN]	29.39	5	5	9	330	31.57714	4.716309	1.1759	1.06569	0.926094	1.0558935	0.520228	0.5884958
Q09666	Neuroblast differentiation-associated protein AHNAK OS=Homo sapiens GN=AHNAK PE=1 SV=2 - [AHNK_HUMAN]	55.64	183	185	359	5890	628.6994	6.150879	1.05599	1.07726	1.03327	1.0555068	0.0485588	0.1006562
P25705	ATP synthase subunit alpha, mitochondrial OS=Homo sapiens GN=ATP5A1 PE=1 SV=1 - [ATPA_HUMAN]	43.04	21	21	49	553	59.7136	9.129395	1.05782	1.05972	1.04859	1.0553774	0.00382804	0.0387187
B4DH12	cDNA FLJ60514, highly similar to Fructosamine-3-kinase (EC 2.7.1.-) OS=Homo sapiens PE=2 SV=1 - [B4DH12_HUMAN]	14.36	2	2	3	202	22.31446	8.70459	1.04406	0.924806	1.19686	1.0552413	0.555571	0.6204579
P08758	Annexin A5 OS=Homo sapiens GN=ANXA5 PE=1 SV=2 - [ANXA5_HUMAN]	74.69	20	21	78	320	35.9144	5.046387	1.04199	1.071	1.05153	1.0548387	0.0233839	0.06649
A0A087WYF6	WASH complex subunit FAM21A OS=Homo sapiens GN=FAM21A PE=1 SV=1 - [A0A087WYF6_HUMAN]	7.47	5	5	5	1245	136.4883	4.754395	1.09085	1.0416	1.02958	1.0540102	0.102276	0.1649483
A0A088QCU6	Choline transporter-like protein 2 isoform 2 OS=Homo sapiens GN=SLC44A2 PE=4 SV=1 - [A0A088QCU6_HUMAN]	7.53	3	4	5	704	79.7934	8.528809	1.01235	1.12014	1.02815	1.0535488	0.252093	0.3223781
K0A7K7	TNF receptor-associated protein 1 (Fragment) OS=Homo sapiens GN=TRAP1 PE=2 SV=1 - [K0A7K7_HUMAN]	8.73	4	4	4	676	76.87975	7.269043	0.986968	1.11606	1.05658	1.0532049	0.289911	0.3626566
P27144	Adenylate kinase 4, mitochondrial OS=Homo sapiens GN=AK4 PE=1 SV=1 - [KAD4_HUMAN]	9.42	2	2	2	223	25.25221	8.396973	1.05345	1.04097	1.06489	1.0531023	0.0165044	0.0565435
H0Y9M9	C-terminal-binding protein 1 (Fragment) OS=Homo sapiens GN=CTBP1 PE=1 SV=1 - [H0Y9M9_HUMAN]	12.41	2	2	2	145	15.78102	7.781738	1.063	1.01432	1.08107	1.0527964	0.117887	0.1822305
Q96RF0	Sorting nexin-18 OS=Homo sapiens GN=SNX18 PE=1 SV=2 - [SNX18_HUMAN]	7.01	3	3	4	628	68.85117	5.681152	0.990753	1.03947	1.12751	1.0525753	0.319381	0.3927006

Biology Open • Supplementary information

H3BQZ7	HCG2044799 OS=Homo sapiens GN=HNRNPUL2-BSCL2 PE=4 SV=1 - [H3BQZ7_HUMAN]	12.06	6	6	7	746	84.6392	4.932129	1.06537	1.06311	1.02914	1.0525399	0.046302	0.0979388
P35527	Keratin, type I cytoskeletal 9 OS=Homo sapiens GN=KRT9 PE=1 SV=3 - [K1C9_HUMAN]	41.41	17	18	37	623	62.02682	5.236816	1.05773	1.06273	1.03618	1.0522132	0.0234962	0.0666678
Q99733	Nucleosome assembly protein 1-like 4 OS=Homo sapiens GN=NAP1L4 PE=1 SV=1 - [NP1L4_HUMAN]	14.4	3	4	5	375	42.79689	4.690918	1.00162	1.12622	1.02836	1.0520666	0.302951	0.3756371
C9JYN0	Synaptophysin-like protein 1 OS=Homo sapiens GN=SYPL1 PE=1 SV=1 - [C9JYN0_HUMAN]	11.61	2	2	5	224	24.76769	7.137207	1.02957	1.04872	1.07786	1.0520514	0.0656546	0.1210462
Q92747	Actin-related protein 2/3 complex subunit 1A OS=Homo sapiens GN=ARPC1A PE=1 SV=2 - [ARC1A_HUMAN]	4.05	2	2	3	370	41.54269	8.177246	1.06292	1.06344	1.02921	1.0518595	0.0445263	0.0957392
P17655	Calpain-2 catalytic subunit OS=Homo sapiens GN=CAPN2 PE=1 SV=6 - [CAN2_HUMAN]	23.43	11	11	17	700	79.9448	4.98291	1.06021	1.07427	1.02093	1.0518035	0.0832505	0.1433382
Q68D64	Putative uncharacterized protein DKFZp686E23276 (Fragment) OS=Homo sapiens GN=DKFZp686E23276 PE=2 SV=1 - [Q68D64_HUMAN]	11.89	3	3	3	328	37.0222	7.942871	1.09335	1.04124	1.02066	1.0517525	0.139161	0.2043008
P17858	ATP-dependent 6-phosphofructokinase, liver type OS=Homo sapiens GN=PFKL PE=1 SV=6 - [PFKAL_HUMAN]	13.59	8	9	10	780	84.96416	7.503418	1.11385	1.02822	1.01301	1.0516923	0.241335	0.310852
B7Z601	cDNA FLJ57187, highly similar to Glycerol-3-phosphate dehydrogenase, mitochondrial (EC 1.1.99.5) OS=Homo sapiens PE=2 SV=1 - [B7Z601_HUMAN]	3.4	2	2	2	500	55.70442	6.20166	1.05332	1.05436	1.04722	1.0516336	0.00185423	0.0312267
A0A024R5L7	Phosphatidylinositol binding clathrin assembly protein, isoform CRA_c OS=Homo sapiens GN=PICALM PE=4 SV=1 - [A0A024R5L7_HUMAN]	10.49	5	5	5	610	66.35082	8.807129	1.00612	1.11224	1.0359	1.0514211	0.245234	0.3151146
D3DRX6	Kinesin-like protein OS=Homo sapiens GN=KIF5B PE=3 SV=1 - [D3DRX6_HUMAN]	11.73	8	9	12	963	109.59	6.507324	1.03177	1.10145	1.02096	1.0513945	0.178465	0.2468754

Biology Open • Supplementary information

P05023	Sodium/potassium-transporting ATPase subunit alpha-1 OS=Homo sapiens GN=ATP1A1 PE=1 SV=1 - [AT1A1_HUMAN]	26.78	15	22	33	1023	112.8239	5.490723	1.06584	1.06927	1.01902	1.0513782	0.0867585	0.1472806
P05997	Collagen alpha-2(V) chain OS=Homo sapiens GN=COL5A2 PE=1 SV=3 - [CO5A2_HUMAN]	3.2	3	3	3	1499	144.821	6.455566	1.02941	1.0363	1.08729	1.0510015	0.107794	0.1707453
Q5VYK3	Proteasome-associated protein ECM29 homolog OS=Homo sapiens GN=ECM29 PE=1 SV=2 - [ECM29_HUMAN]	2.66	5	5	5	1845	204.1604	7.122559	0.984002	1.151	1.01747	1.0508254	0.424058	0.496519
Q14315	Filamin-C OS=Homo sapiens GN=FLNC PE=1 SV=3 - [FLNC_HUMAN]	29.32	45	60	117	2725	290.8406	5.973145	1.06053	1.03771	1.05404	1.0507603	0.0174226	0.05785
B2R8I2	cDNA, FLJ93914, highly similar to Homo sapiens histidine-rich glycoprotein (HRG), mRNA OS=Homo sapiens PE=2 SV=1 - [B2R8I2_HUMAN]	30.48	14	14	25	525	59.47478	7.444824	1.04067	1.04983	1.06171	1.0507372	0.0141111	0.0528386
D6RE99	Histidine triad nucleotide-binding protein 1 OS=Homo sapiens GN=HINT1 PE=1 SV=1 - [D6RE99_HUMAN]	43.59	2	2	4	78	8.628483	5.38916	1.04555	1.10009	1.00654	1.050724	0.202438	0.2729003
Q6DEN2	DPYSL3 protein OS=Homo sapiens GN=DPYSL3 PE=2 SV=1 - [Q6DEN2_HUMAN]	43.71	22	24	47	684	73.86395	6.55127	1.07626	1.03097	1.04494	1.0507238	0.0631537	0.1179775
P54652	Heat shock-related 70 kDa protein 2 OS=Homo sapiens GN=HSPA2 PE=1 SV=1 - [HSP72_HUMAN]	30.99	7	16	44	639	69.97787	5.744629	1.02097	1.14281	0.988186	1.0506549	0.394166	0.4670115
D6RGI3	Septin 11, isoform CRA_b OS=Homo sapiens GN=SEPT11 PE=1 SV=1 - [D6RGI3_HUMAN]	32.47	4	11	20	425	48.97506	6.961426	1.07606	1.02063	1.055	1.0505627	0.0887185	0.1494818
O15075	Serine/threonine-protein kinase DCLK1 OS=Homo sapiens GN=DCLK1 PE=1 SV=2 - [DCLK1_HUMAN]	9.32	6	6	6	740	82.17296	8.660645	1.04426	1.07145	1.03597	1.0505563	0.042105	0.0926217
Q68D38	Putative uncharacterized protein DKFZp686O15119 (Fragment) OS=Homo sapiens GN=DKFZp686O15119 PE=2 SV=1 - [Q68D38_HUMAN]	10.71	2	2	2	252	28.02046	5.79541	1	1.07299	1.07867	1.0505542	0.184077	0.2529844

A0A024R6D1	NIMA (Never in mitosis gene a)-related kinase 9, isoform CRA_a OS=Homo sapiens GN=NEK9 PE=4 SV=1 - [A0A024R6D1_HUMAN]	3.58	3	3	3	979	107.0812	5.731934	1.09074	1.03444	1.02632	1.0505013	0.130166	0.1951337
B4DMD3	cDNA FLJ58174, highly similar to WW domain-binding protein 11 OS=Homo sapiens PE=2 SV=1 - [B4DMD3_HUMAN]	4.24	2	2	2	589	64.86705	7.005371	0.964438	1.1203	1.06641	1.0503818	0.385195	0.4581466
Q15019	Septin-2 OS=Homo sapiens GN=SEPT2 PE=1 SV=1 - [SEPT2_HUMAN]	37.12	9	9	18	361	41.46125	6.595215	1.04298	1.04403	1.06334	1.0501151	0.0169914	0.0572932
B4DWM5	cDNA FLJ61436, highly similar to Melanoma-associated antigen D2 OS=Homo sapiens PE=2 SV=1 - [B4DWM5_HUMAN]	13.1	3	3	3	229	26.17244	10.15479	1.09794	1.02096	1.03123	1.0500436	0.173814	0.2414282
Q14204	Cytoplasmic dynein 1 heavy chain 1 OS=Homo sapiens GN=DYNC1H1 PE=1 SV=5 - [DYHC1_HUMAN]	17.89	68	68	88	4646	532.0719	6.404785	1.04264	1.06337	1.04411	1.0500394	0.0173574	0.05785
B4DJJ5	cDNA FLJ53423, highly similar to FK506-binding protein 10 (EC 5.2.1.8) OS=Homo sapiens PE=2 SV=1 - [B4DJJ5_HUMAN]	11.37	5	5	5	510	56.12333	5.681152	1.20423	1.01685	0.92547	1.0488514	0.611968	0.6725687
A5YM53	ITGAV protein OS=Homo sapiens GN=ITGAV PE=2 SV=1 - [A5YM53_HUMAN]	7.06	5	5	7	1048	116.0316	5.744629	1.08899	1.01052	1.04642	1.0486427	0.165156	0.2330359
A0A087WT27	Phosphoacetylglucosamine mutase OS=Homo sapiens GN=PGM3 PE=1 SV=1 - [A0A087WT27_HUMAN]	12.02	4	4	4	441	49.06372	6.366699	1.02808	1.09032	1.02737	1.0485888	0.145277	0.2110569
B3KMV8	cDNA FLJ12766 fis, clone NT2RP2001520, highly similar to Calcium-binding mitochondrial carrier protein Aralar1 OS=Homo sapiens PE=2 SV=1 - [B3KMV8_HUMAN]	13.13	5	7	12	678	74.73085	8.382324	1.02905	1.10157	1.0147	1.0484404	0.213387	0.2829279
E2QRB9	Thioredoxin reductase 1, cytoplasmic OS=Homo sapiens GN=TXNRD1 PE=1 SV=2 - [E2QRB9_HUMAN]	6.52	3	3	4	460	50.69092	6.624512	1.04685	1.0125	1.08566	1.0483342	0.149441	0.2159017
Q4G0N4	NAD kinase 2, mitochondrial OS=Homo sapiens GN=NADK2 PE=1 SV=2 - [NAKD2_HUMAN]	9.05	3	3	3	442	49.40217	8.177246	1.08181	1.1566	0.906331	1.0482467	0.582118	0.6445762

Biology Open • Supplementary information

P40939	Trifunctional enzyme subunit alpha, mitochondrial OS=Homo sapiens GN=HADHA PE=1 SV=2 - [ECHA_HUMAN]	33.16	22	22	40	763	82.94688	9.041504	1.04762	1.02936	1.06701	1.0479966	0.0476602	0.0994022
Q2M389	WASH complex subunit 7 OS=Homo sapiens GN=KIAA1033 PE=1 SV=2 - [WASH7_HUMAN]	2.13	2	2	2	1173	136.3162	7.444824	0.955109	1.10822	1.08031	1.0478824	0.416156	0.4886188
Q86TY5	Galectin OS=Homo sapiens PE=2 SV=1 - [Q86TY5_HUMAN]	52.89	5	5	16	121	13.88822	9.158691	1.02904	1.05552	1.0586	1.0477173	0.0365464	0.0854261
P19784	Casein kinase II subunit alpha' OS=Homo sapiens GN=CSNK2A2 PE=1 SV=1 - [CSK22_HUMAN]	10.29	2	3	4	350	41.18713	8.558105	1.05413	1.04412	1.04412	1.0474546	0.00490531	0.0401071
B7Z8K4	cDNA FLJ51772, highly similar to Ras-GTPase-activating protein-binding protein 1 (EC 3.6.1.-) OS=Homo sapiens PE=2 SV=1 - [B7Z8K4_HUMAN]	20.07	4	4	5	284	31.49211	5.973145	1.1479	0.997978	0.996425	1.0474348	0.444711	0.5152487
Q9NZN3	EH domain-containing protein 3 OS=Homo sapiens GN=EHD3 PE=1 SV=2 - [EHD3_HUMAN]	13.46	2	8	18	535	60.84872	6.565918	1.06997	1.0576	1.01462	1.0473969	0.105715	0.1685564
B4DKX2	cDNA FLJ52931, highly similar to Homo sapiens abl-interactor 1 (ABI1), transcript variant 3, mRNA OS=Homo sapiens PE=2 SV=1 - [B4DKX2_HUMAN]	11.32	2	2	2	318	34.13159	6.442871	1.02839	1.05089	1.06249	1.0472589	0.0420476	0.0926217
A0A087WZK9	Eukaryotic translation initiation factor 3 subunit H OS=Homo sapiens GN=EIF3H PE=1 SV=1 - [A0A087WZK9_HUMAN]	11.75	3	3	5	349	39.56481	6.39209	1.07213	1.04947	1.01986	1.0471544	0.0894007	0.1502792
A0A024R652	Methylenetetrahydrofolate dehydrogenase (NADP+ dependent) 1, methenyltetrahydrofolate cyclohydrolase, formyltetrahydrofolate synthetase, isoform CRA_a OS=Homo sapiens GN=MTHFD1 PE=3 SV=1 - [A0A024R652_HUMAN]	11.55	9	9	12	935	101.4673	7.181152	1.06714	1.02375	1.05047	1.0471198	0.0649818	0.1201788
A8K897	cDNA FLJ78686, highly similar to Homo sapiens nucleoporin 93kDa (NUP93), mRNA OS=Homo sapiens PE=2 SV=1 - [A8K897_HUMAN]	3.3	2	2	2	819	93.32362	5.77002	1.05389	1.06638	1.02003	1.0467685	0.0776025	0.1360078
P53007	Tricarboxylate transport protein, mitochondrial OS=Homo sapiens GN=SLC25A1 PE=1 SV=2 - [TXTP_HUMAN]	5.14	2	2	2	311	33.991	9.891113	1.0498	1.07619	1.01301	1.046334	0.127186	0.1922843

Biology Open • Supplementary information

P12270	Nucleoprotein TPR OS=Homo sapiens GN=TPR PE=1 SV=3 - [TPR_HUMAN]	6.39	11	11	11	2363	267.1311	5.020996	1.06318	1.05545	1.01999	1.0462079	0.073768	0.1310786
A0A024RAM4	Microtubule-associated protein 1B, isoform CRA_b OS=Homo sapiens GN=MAP1B PE=4 SV=1 - [A0A024RAM4_HUMAN]	12.07	20	21	24	2468	270.454	4.805176	1.04026	1.0767	1.02068	1.0458823	0.107674	0.1706351
P11215	Integrin alpha-M OS=Homo sapiens GN=ITGAM PE=1 SV=2 - [ITAM_HUMAN]	4.25	4	4	4	1152	127.0985	7.225098	1.1618	0.962535	1.01214	1.0454904	0.526838	0.5946471
Q59GA0	Thy-1 cell surface antigen variant (Fragment) OS=Homo sapiens PE=2 SV=1 - [Q59GA0_HUMAN]	27.59	4	4	12	145	15.89405	8.997559	1.00225	1.07088	1.06314	1.0454238	0.171407	0.2388111
P21333	Filamin-A OS=Homo sapiens GN=FLNA PE=1 SV=4 - [FLNA_HUMAN]	56.89	75	124	535	2647	280.5639	6.062012	1.03838	1.05224	1.04505	1.0452229	0.00774906	0.045203
P62736	Actin, aortic smooth muscle OS=Homo sapiens GN=ACTA2 PE=1 SV=1 - [ACTA_HUMAN]	63.66	2	22	444	377	41.98181	5.38916	1.07901	1.0085	1.0478	1.0451035	0.15759	0.2249392
B7SBB1	Aryl hydrocarbon receptor interacting protein OS=Homo sapiens GN=AIP PE=4 SV=1 - [B7SBB1_HUMAN]	24.01	6	6	8	329	37.47709	6.536621	1.11231	1.05852	0.964267	1.0450308	0.407259	0.4796701
H0YFI1	Ragulator complex protein LAMTOR1 (Fragment) OS=Homo sapiens GN=LAMTOR1 PE=1 SV=1 - [H0YFI1_HUMAN]	31.65	2	2	3	79	8.702243	5.38916	1.08609	1.04174	1.00706	1.0449602	0.188225	0.2572231
X6RFL8	Ras-related protein Rab-14 (Fragment) OS=Homo sapiens GN=RAB14 PE=1 SV=1 - [X6RFL8_HUMAN]	40.88	5	6	10	181	20.39631	6.328613	1.01129	1.0625	1.05991	1.0445656	0.115906	0.1800106
E5RFE7	Carbonic anhydrase 1 (Fragment) OS=Homo sapiens GN=CA1 PE=1 SV=1 - [E5RFE7_HUMAN]	47.94	7	7	20	194	21.38874	7.664551	1.08515	1.05369	0.994714	1.0445177	0.235075	0.3043021
Q7Z4I7	LIM and senescent cell antigen-like-containing domain protein 2 OS=Homo sapiens GN=LIMS2 PE=1 SV=1 - [LIMS2_HUMAN]	17.89	6	6	11	341	38.88957	8.04541	1.0508	1.00503	1.07756	1.0444662	0.170554	0.2383672
Q92841	Probable ATP-dependent RNA helicase DDX17 OS=Homo sapiens GN=DDX17 PE=1 SV=2 - [DDX17_HUMAN]	11.52	4	8	11	729	80.22202	8.265137	1.0885	1.00836	1.03628	1.0443778	0.19939	0.2691979

Biology Open • Supplementary information

Q9Y5M8	Signal recognition particle receptor subunit beta OS=Homo sapiens GN=SRPRB PE=1 SV=3 - [SRPRB_HUMAN]	21.77	4	4	6	271	29.68376	9.041504	1.18655	0.996136	0.949675	1.0441214	0.604574	0.6650903
Q6P2Q9	Pre-mRNA-processing-splicing factor 8 OS=Homo sapiens GN=PRPF8 PE=1 SV=2 - [PRP8_HUMAN]	3.08	7	7	7	2335	273.4266	8.836426	1.06419	1.01489	1.05253	1.0438734	0.0983024	0.1605179
O95782	AP-2 complex subunit alpha-1 OS=Homo sapiens GN=AP2A1 PE=1 SV=3 - [AP2A1_HUMAN]	17.81	9	16	19	977	107.4778	7.034668	1.08786	1.06322	0.9796	1.0435617	0.315021	0.3876213
R4GN98	Protein S100 (Fragment) OS=Homo sapiens GN=S100A6 PE=1 SV=1 - [R4GN98_HUMAN]	28.24	4	4	9	85	9.675063	5.452637	0.973991	1.07467	1.08198	1.0435484	0.337774	0.4115785
Q96I24	Far upstream element-binding protein 3 OS=Homo sapiens GN=FUBP3 PE=1 SV=2 - [FUBP3_HUMAN]	8.04	2	3	3	572	61.60222	8.382324	0.952901	1.08939	1.08814	1.0434756	0.43837	0.5097447
B2R5M8	Isocitrate dehydrogenase [NADP] OS=Homo sapiens PE=2 SV=1 - [B2R5M8_HUMAN]	22.71	9	9	13	414	46.6195	7.005371	1.10464	1.03074	0.994918	1.0434321	0.310968	0.3837502
B4DJQ8	cDNA FLJ55694, highly similar to Dipeptidyl-peptidase 1 (EC 3.4.14.1) OS=Homo sapiens PE=2 SV=1 - [B4DJQ8_HUMAN]	13.9	5	5	9	446	50.1192	6.990723	0.995769	1.06049	1.07353	1.0432621	0.21377	0.2831521
P35221	Catenin alpha-1 OS=Homo sapiens GN=CTNNA1 PE=1 SV=1 - [CTNA1_HUMAN]	13.47	11	11	11	906	100.0085	6.290527	1.07778	1.0364	1.01489	1.0430244	0.145004	0.2110226
P28066	Proteasome subunit alpha type-5 OS=Homo sapiens GN=PSMA5 PE=1 SV=3 - [PSA5_HUMAN]	35.27	5	5	9	241	26.39421	4.79248	1.08076	1.01652	1.03148	1.0429208	0.15749	0.2249383
A0A087X0K1	Calcium-binding protein 39 OS=Homo sapiens GN=CAB39 PE=1 SV=1 - [A0A087X0K1_HUMAN]	9.14	3	3	3	339	39.42766	7.10791	0.989253	1.13737	1.00194	1.0428539	0.461363	0.5310145
B4DXT2	cDNA FLJ59635, highly similar to Heat shock 70 kDa protein 4L OS=Homo sapiens PE=2 SV=1 - [B4DXT2_HUMAN]	6.15	2	4	4	813	91.88137	6.087402	0.995872	1.10094	1.02993	1.0422495	0.305499	0.378077
X5CMH5	Antigen peptide transporter 2 OS=Homo sapiens GN=TAP2 PE=1 SV=1 - [X5CMH5_HUMAN]	7.25	4	4	4	703	77.64581	7.85498	1.01106	1.02956	1.08388	1.0414999	0.197958	0.2677139
Q8N5K1	CDGSH iron-sulfur domain-containing protein 2 OS=Homo sapiens GN=CISD2 PE=1 SV=1 - [CISD2_HUMAN]	25.93	3	3	3	135	15.26845	9.612793	0.956024	1.12711	1.04088	1.0413388	0.490666	0.5597925

Biology Open • Supplementary information

B7Z4P9	cDNA FLJ51678, highly similar to Ras-related protein Rab-18 OS=Homo sapiens PE=2 SV=1 - [B7Z4P9_HUMAN]	10.66	2	2	2	197	22.44396	8.968262	1.08225	1.05634	0.985367	1.0413213	0.289757	0.362598
Q8N766	ER membrane protein complex subunit 1 OS=Homo sapiens GN=EMC1 PE=1 SV=1 - [EMC1_HUMAN]	3.02	2	2	2	993	111.6891	7.664551	1.04474	0.946081	1.13249	1.0411031	0.524979	0.5931422
A0A024R4E5	High density lipoprotein binding protein (Vigilin), isoform CRA_a OS=Homo sapiens GN=HDLBP PE=1 SV=1 - [A0A024R4E5_HUMAN]	10.33	9	10	11	1268	141.3522	6.873535	1.06003	1.01476	1.04815	1.0409791	0.0941701	0.1554197
P35237	Serpin B6 OS=Homo sapiens GN=SERPINB6 PE=1 SV=3 - [SPB6_HUMAN]	23.94	7	7	11	376	42.59406	5.274902	1.02461	1.06794	1.03022	1.0409231	0.0950207	0.1563678
C9J9K3	40S ribosomal protein SA (Fragment) OS=Homo sapiens GN=RPSA PE=1 SV=6 - [C9J9K3_HUMAN]	23.57	6	6	10	263	29.38595	5.249512	1.04973	0.962831	1.11019	1.0409161	0.439666	0.5109061
O43707	Alpha-actinin-4 OS=Homo sapiens GN=ACTN4 PE=1 SV=2 - [ACTN4_HUMAN]	64.32	37	51	167	911	104.7885	5.439941	1.02648	1.06719	1.02844	1.0407026	0.0916798	0.1526499
B4E1I8	cDNA FLJ54228, highly similar to Leucine-rich alpha-2-glycoprotein OS=Homo sapiens PE=2 SV=1 - [B4E1I8_HUMAN]	7.58	3	3	4	330	36.47122	7.210449	1.06587	1.0428	1.01278	1.0404837	0.118971	0.1834701
D3DTY9	Tripartite motif-containing 25, isoform CRA_a OS=Homo sapiens GN=TRIM25 PE=4 SV=1 - [D3DTY9_HUMAN]	14.69	5	5	5	422	47.84472	9.070801	0.970799	1.09338	1.05703	1.0404046	0.382055	0.4552116
B7Z792	cDNA FLJ53932, highly similar to NADH-ubiquinone oxidoreductase 49 kDa subunit, mitochondrial (EC 1.6.5.3) OS=Homo sapiens PE=2 SV=1 - [B7Z792_HUMAN]	10.84	4	4	6	406	46.36657	7.342285	0.993096	1.08309	1.04393	1.0400398	0.264123	0.3352292
B7Z2Z1	cDNA FLJ59523, highly similar to Scaffold attachment factor B OS=Homo sapiens PE=2 SV=1 - [B7Z2Z1_HUMAN]	7.03	4	4	5	811	91.03038	7.239746	0.968263	1.06981	1.08156	1.039879	0.383021	0.456202
B3KTQ2	cDNA FLJ38589 fis, clone HCHON2010074, highly similar to LACTADHERIN OS=Homo sapiens PE=2 SV=1 - [B3KTQ2_HUMAN]	19.79	6	6	8	379	42.43292	7.811035	1.00583	1.04857	1.0649	1.0397654	0.152505	0.2196721

Biology Open • Supplementary information

Q8IY17	Neuropathy target esterase OS=Homo sapiens GN=PNPLA6 PE=1 SV=2 - [PLPL6_HUMAN]	1.61	2	2	3	1366	149.9002	7.811035	1.09035	1.02951	0.998089	1.0393175	0.283687	0.3564516
A8K4Z4	cDNA FLJ75549, highly similar to Homo sapiens ribosomal protein, large, P0 (RPLP0), transcript variant 1, mRNA OS=Homo sapiens PE=2 SV=1 - [A8K4Z4_HUMAN]	44.48	10	10	20	317	34.21782	5.973145	0.99122	1.058	1.06816	1.039126	0.246396	0.3161677
E9PJS0	Acetolactate synthase-like protein (Fragment) OS=Homo sapiens GN=ILVBL PE=1 SV=1 - [E9PJS0_HUMAN]	10.69	2	2	2	262	27.53378	9.378418	1.08958	0.963665	1.06388	1.0390422	0.416387	0.4886638
P09960	Leukotriene A-4 hydrolase OS=Homo sapiens GN=LTA4H PE=1 SV=2 - [LKHA4_HUMAN]	19.64	11	11	17	611	69.24124	6.17627	1.06814	1.01733	1.03033	1.0385998	0.126889	0.1921786
F8W1R7	Myosin light polypeptide 6 OS=Homo sapiens GN=MYL6 PE=1 SV=1 - [F8W1R7_HUMAN]	54.48	8	8	32	145	16.2798	4.652832	1.06009	1.07109	0.984324	1.0384996	0.293543	0.3662525
Q13724	Mannosyl-oligosaccharide glucosidase OS=Homo sapiens GN=MOGS PE=1 SV=5 - [MOGS_HUMAN]	11.35	7	7	9	837	91.86059	8.89502	1.06978	1.00923	1.03625	1.0384173	0.159527	0.2268903
P27816	Microtubule-associated protein 4 OS=Homo sapiens GN=MAP4 PE=1 SV=3 - [MAP4_HUMAN]	17.71	16	17	25	1152	120.9298	5.427246	1.07063	1.03413	1.01021	1.0383239	0.16089	0.2282534
B3KUZ6	cDNA FLJ40980 fis, clone UTERU2014464, highly similar to ACID CERAMIDASE (EC 3.5.1.23) OS=Homo sapiens PE=2 SV=1 - [B3KUZ6_HUMAN]	23.93	6	6	8	305	34.57172	7.76709	1.02204	1.12586	0.965479	1.0377926	0.505434	0.5737405
A8K8U1	cDNA FLJ77762, highly similar to Homo sapiens cullin-associated and neddylation-dissociated 1 (CAND1), mRNA OS=Homo sapiens PE=2 SV=1 - [A8K8U1_HUMAN]	16.83	17	17	28	1230	136.2307	5.833496	1.01503	1.05922	1.03718	1.037147	0.100461	0.1627974
B3KTA3	Fascin OS=Homo sapiens PE=2 SV=1 - [B3KTA3_HUMAN]	15.89	7	7	14	472	52.24396	7.649902	1.05172	1.05032	1.00883	1.0369571	0.11948	0.1839158
A0PJ51	STK4 protein (Fragment) OS=Homo sapiens GN=STK4 PE=2 SV=1 - [A0PJ51_HUMAN]	12.84	2	4	4	405	45.89498	5.097168	1.12548	1.00342	0.981912	1.0369367	0.495531	0.5643918
P42704	Leucine-rich PPR motif-containing protein, mitochondrial OS=Homo sapiens GN=LRPPRC PE=1 SV=3 -	10.83	12	12	14	1394	157.8051	6.125488	1.05618	0.996086	1.05528	1.0358496	0.213168	0.2828345

Biology Open • Supplementary information

	[LPPRC_HUMAN]														
B7Z9C4	cDNA FLJ54863, highly similar to CTP synthase 1 (EC 6.3.4.2) OS=Homo sapiens PE=2 SV=1 - [B7Z9C4_HUMAN]	6.37	2	2	2	314	35.23118	5.909668	0.942202	1.1209	1.044	1.0357033	0.561567	0.6257091
Q5QNZ2	ATP synthase F(0) complex subunit B1, mitochondrial OS=Homo sapiens GN=ATP5F1 PE=1 SV=1 - [Q5QNZ2_HUMAN]	18.46	4	4	7	195	22.26079	9.26123	1.17158	1.04226	0.892752	1.035532	0.702271	0.7537871
A0A0A0MQY0	PDZ and LIM domain protein 3 OS=Homo sapiens GN=PDLIM3 PE=1 SV=1 - [A0A0A0MQY0_HUMAN]	35.87	7	7	16	276	30.13799	7.723145	1.026	0.989543	1.09087	1.03547	0.353927	0.4275671
M0R080	DnaJ homolog subfamily B member 1 (Fragment) OS=Homo sapiens GN=DNAJB1 PE=1 SV=1 - [M0R080_HUMAN]	25.14	2	3	4	183	20.48434	8.558105	1.12357	1.04317	0.939228	1.0353201	0.576075	0.6383024
Q6FG99	RPLP1 protein OS=Homo sapiens GN=RPLP1 PE=2 SV=1 - [Q6FG99_HUMAN]	51.75	2	2	5	114	11.56076	4.373535	1.02441	1.14349	0.936318	1.0347382	0.621272	0.6819085
P21912	Succinate dehydrogenase [ubiquinone] iron-sulfur subunit, mitochondrial OS=Homo sapiens GN=SDHB PE=1 SV=3 - [SDHB_HUMAN]	10.71	3	3	3	280	31.60884	8.763184	1.01708	1.03765	1.04811	1.0342778	0.0640011	0.1192316
Q9Y305	Acyl-coenzyme A thioesterase 9, mitochondrial OS=Homo sapiens GN=ACOT9 PE=1 SV=2 - [ACOT9_HUMAN]	15.26	5	5	8	439	49.86952	8.602051	0.94931	1.1768	0.97655	1.0342212	0.680325	0.7336413
P07357	Complement component C8 alpha chain OS=Homo sapiens GN=C8A PE=1 SV=2 - [CO8A_HUMAN]	15.58	7	7	8	584	65.12104	6.468262	1.05369	1.0535	0.993773	1.0336551	0.233516	0.302855
B7ZLW0	LPP protein OS=Homo sapiens GN=LPP PE=2 SV=1 - [B7ZLW0_HUMAN]	51.8	21	23	52	612	65.73187	7.371582	1.03463	1.0081	1.05802	1.0335843	0.145253	0.2110569
Q9NVD7	Alpha-parvin OS=Homo sapiens GN=PARVA PE=1 SV=1 - [PARVA_HUMAN]	22.85	6	8	12	372	42.21743	5.947754	1.02493	1.03221	1.0418	1.0329832	0.021245	0.0632079
B2RD27	cDNA, FLJ96428, highly similar to Homo sapiens proteasome (prosome, macropain) 26S subunit, non-ATPase, 7 (Mov34	9.26	2	2	2	324	36.98848	6.770996	1.09194	0.968761	1.03749	1.0327322	0.455345	0.5254341

Biology Open • Supplementary information

	homolog) (PSMD7), mRNA OS=Homo sapiens PE=2 SV=1 - [B2RD27_HUMAN]														
B4E0E1	Poly [ADP-ribose] polymerase OS=Homo sapiens PE=2 SV=1 - [B4E0E1_HUMAN]	12.79	9	9	13	993	111.0564	8.865723	1.02795	0.994137	1.07596	1.0326818	0.302436	0.3751359
O00264	Membrane-associated progesterone receptor component 1 OS=Homo sapiens GN=PGRMC1 PE=1 SV=3 - [PGRC1_HUMAN]	25.13	5	5	5	195	21.65776	4.703613	1.04012	1.03671	1.02111	1.032647	0.0306749	0.0773154
Q96HS1	Serine/threonine-protein phosphatase PGAM5, mitochondrial OS=Homo sapiens GN=PGAM5 PE=1 SV=2 - [PGAM5_HUMAN]	9	3	3	3	289	31.98461	8.675293	1.05365	0.997005	1.04651	1.0323872	0.210632	0.2801413
D3DWY2	Inhibitor of kappa light polypeptide gene enhancer in B-cells, kinase gamma, isoform CRA_a OS=Homo sapiens GN=IKBKG PE=4 SV=1 - [D3DWY2_HUMAN]	11.97	2	2	2	234	26.72158	5.566895	1.0662	0.986285	1.04403	1.0321726	0.309332	0.3821492
P46108	Adapter molecule crk OS=Homo sapiens GN=CRK PE=1 SV=2 - [CRK_HUMAN]	16.78	4	4	5	304	33.81002	5.554199	0.979574	0.999745	1.1162	1.0318396	0.532575	0.5997227
P13798	Acylamino-acid-releasing enzyme OS=Homo sapiens GN=APEH PE=1 SV=4 - [ACPH_HUMAN]	7.65	4	4	6	732	81.17262	5.478027	1.00085	1.0893	1.00534	1.0318337	0.383714	0.456706
P35606	Coatomer subunit beta' OS=Homo sapiens GN=COPB2 PE=1 SV=2 - [COPB2_HUMAN]	9.16	6	6	7	906	102.4225	5.274902	1.03969	1.00632	1.04848	1.0314964	0.133663	0.1987041
I3L4P6	Rho GTPase-activating protein 17 (Fragment) OS=Homo sapiens GN=ARHGAP17 PE=1 SV=1 - [I3L4P6_HUMAN]	36.84	2	2	2	76	8.619398	4.475098	1.04927	0.999722	1.04529	1.0314258	0.186631	0.2556641
Q9Y696	Chloride intracellular channel protein 4 OS=Homo sapiens GN=CLIC4 PE=1 SV=4 - [CLIC4_HUMAN]	49.01	8	8	11	253	28.75375	5.592285	0.962405	1.06773	1.06389	1.031342	0.459375	0.5292663
P43243	Matrin-3 OS=Homo sapiens GN=MATR3 PE=1 SV=2 - [MATR3_HUMAN]	10.27	8	8	11	847	94.5648	6.252441	1.01004	1.01373	1.06789	1.0305514	0.243893	0.3137886
O75369	Filamin-B OS=Homo sapiens GN=FLNB PE=1 SV=2 -	34.55	59	69	106	2602	277.9901	5.731934	1.05386	1.01437	1.02342	1.0305454	0.124899	0.1902239

Biology Open • Supplementary information

	[FLNB_HUMAN]														
Q59HB8	Nuclear mitotic apparatus protein 1 variant (Fragment) OS=Homo sapiens PE=2 SV=1 - [Q59HB8_HUMAN]	10.47	12	12	14	1585	177.9745	6.328613	1.05931	1.01735	1.01386	1.0301749	0.174782	0.2424742
P78371	T-complex protein 1 subunit beta OS=Homo sapiens GN=CCT2 PE=1 SV=4 - [TCPB_HUMAN]	42.99	17	17	22	535	57.45213	6.455566	1.00768	1.06552	1.01718	1.0301263	0.234579	0.3038844
A8KA41	cDNA FLJ75060, highly similar to Homo sapiens 2'-5'-oligoadenylate synthetase 2, 69/71kDa, mRNA OS=Homo sapiens PE=2 SV=1 - [A8KA41_HUMAN]	5.24	3	3	3	687	78.7668	7.913574	1.05781	1.02055	1.01165	1.0300023	0.167859	0.2359646
Q00610	Clathrin heavy chain 1 OS=Homo sapiens GN=CLTC PE=1 SV=5 - [CLH1_HUMAN]	32.66	45	45	76	1675	191.4925	5.693848	1.04369	1.02915	1.01532	1.0293856	0.0696911	0.1260847
P02776	Platelet factor 4 OS=Homo sapiens GN=PF4 PE=1 SV=2 - [PLF4_HUMAN]	35.64	4	4	5	101	10.83789	8.616699	1.03405	1.07479	0.979201	1.0293471	0.400352	0.4731814
B4E3S9	cDNA FLJ53443, highly similar to Leiomodin-1 OS=Homo sapiens PE=2 SV=1 - [B4E3S9_HUMAN]	23.13	10	10	17	549	61.49607	9.158691	1.05289	1.02356	1.01138	1.0292758	0.140692	0.2060194
Q53G58	Coronin (Fragment) OS=Homo sapiens PE=2 SV=1 - [Q53G58_HUMAN]	19.2	10	10	14	474	53.24707	7.078613	0.98707	1.08824	1.01112	1.0288087	0.444773	0.5152487
P35232	Prohibitin OS=Homo sapiens GN=PHB PE=1 SV=1 - [PHB_HUMAN]	32.72	8	8	15	272	29.7859	5.757324	1.01225	1.09954	0.973352	1.0283792	0.52632	0.5944589
A0A024R1T5	2',3'-cyclic-nucleotide 3'-phosphodiesterase OS=Homo sapiens GN=CNP PE=3 SV=1 - [A0A024R1T5_HUMAN]	10.47	4	4	5	401	45.07033	8.528809	0.960423	1.0815	1.04319	1.0283712	0.510396	0.5784032
P05546	Heparin cofactor 2 OS=Homo sapiens GN=SERPIND1 PE=1 SV=3 - [HEP2_HUMAN]	17.64	9	9	18	499	57.03421	6.902832	0.999596	1.04646	1.03876	1.0282751	0.190691	0.2599405
B3KUB9	SWAP-70 protein, isoform CRA_b OS=Homo sapiens GN=SWAP70 PE=2 SV=1 - [B3KUB9_HUMAN]	3.8	2	2	2	527	61.99491	6.01123	1.04129	1.02601	1.01713	1.0281414	0.0574964	0.1120445
B4DTY9	Integrin beta OS=Homo sapiens PE=2 SV=1 - [B4DTY9_HUMAN]	5.06	3	3	3	751	83.53159	5.059082	0.973896	1.01764	1.09242	1.0279874	0.503551	0.5721786

Biology Open • Supplementary information

P15924	Desmoplakin OS=Homo sapiens GN=DSP PE=1 SV=3 - [DESP_HUMAN]	2.23	5	5	5	2871	331.5687	6.814941	0.974144	0.976904	1.13244	1.0278289	0.647914	0.7052054
A0A096LNH5	Protein LOC102724023 OS=Homo sapiens GN=LOC102724023 PE=4 SV=1 - [A0A096LNH5_HUMAN]	42.31	5	5	6	182	19.40406	7.825684	0.995231	1.04323	1.04396	1.0274739	0.230486	0.2997292
Q9UHQ9	NADH-cytochrome b5 reductase 1 OS=Homo sapiens GN=CYB5R1 PE=1 SV=1 - [NB5R1_HUMAN]	26.56	7	7	7	305	34.07318	9.378418	1.10002	1.00343	0.978639	1.0273628	0.536867	0.6039531
Q8NBJ5	Procollagen galactosyltransferase 1 OS=Homo sapiens GN=COLGALT1 PE=1 SV=1 - [GT251_HUMAN]	9	5	5	8	622	71.59049	7.312988	1.02363	1.08279	0.975379	1.0272656	0.4726	0.5421047
Q07955	Serine/arginine-rich splicing factor 1 OS=Homo sapiens GN=SRSF1 PE=1 SV=2 - [SRSF1_HUMAN]	24.6	6	6	8	248	27.72782	10.35986	1.01688	0.991712	1.07071	1.026433	0.374268	0.4476669
A8K8K1	cDNA FLJ76936, highly similar to Homo sapiens RNA terminal phosphate cyclase domain 1 (RTCD1), mRNA OS=Homo sapiens PE=2 SV=1 - [A8K8K1_HUMAN]	6.56	2	2	2	366	39.29545	8.04541	1.0251	1.1438	0.910345	1.0264144	0.732932	0.7811634
B3KN82	cDNA FLJ13913 fis, clone Y79AA1000231, highly similar to Nucleolar protein NOP5 OS=Homo sapiens PE=2 SV=1 - [B3KN82_HUMAN]	7.94	3	3	3	466	52.25565	9.012207	1.0366	1.03851	1.00372	1.0262781	0.145441	0.2112045
Q9BUX5	ACAD9 protein (Fragment) OS=Homo sapiens GN=ACAD9 PE=2 SV=2 - [Q9BUX5_HUMAN]	5.25	2	2	2	343	38.54505	8.32373	1.01186	1.01688	1.04961	1.0261198	0.158065	0.2252842
P10153	Non-secretory ribonuclease OS=Homo sapiens GN=RNASE2 PE=1 SV=2 - [RNAS2_HUMAN]	19.88	3	3	10	161	18.34208	8.733887	1.0552	1.02756	0.995299	1.0260184	0.271672	0.3437793
Q5RLJ0	CLE OS=Homo sapiens PE=2 SV=1 - [Q5RLJ0_HUMAN]	19.26	4	4	7	244	27.95163	6.442871	1.00519	1.04247	1.02839	1.0253526	0.144867	0.2109139
A8K8F6	cDNA FLJ78417, highly similar to Homo sapiens low density lipoprotein receptor-related protein associated protein 1 (LRPAP1), mRNA OS=Homo sapiens PE=2 SV=1 - [A8K8F6_HUMAN]	6.72	3	3	4	357	41.38793	9.056152	1.00059	1.0715	1.00394	1.0253399	0.387061	0.4597198
A8K586	AP-3 complex subunit beta OS=Homo sapiens PE=2 SV=1 -	3.02	2	2	3	1094	121.2145	5.973145	1.03434	0.927713	1.11366	1.0252396	0.685516	0.7378288

Biology Open • Supplementary information

	[A8K586_HUMAN]														
P30049	ATP synthase subunit delta, mitochondrial OS=Homo sapiens GN=ATP5D PE=1 SV=2 - [ATPD_HUMAN]	13.69	2	2	5	168	17.47919	5.490723	1.03219	1.01203	1.03121	1.0251446	0.0618833	0.1164397
Q13707	ACTA2 protein (Fragment) OS=Homo sapiens GN=ACTA2 PE=3 SV=1 - [Q13707_HUMAN]	55.76	2	16	320	330	36.78319	5.351074	1.0277	0.992371	1.05536	1.025143	0.301769	0.3744681
B4DDD8	Histidine--tRNA ligase, cytoplasmic OS=Homo sapiens GN=HARS PE=1 SV=1 - [B4DDD8_HUMAN]	15.4	6	6	10	435	48.5387	5.300293	1.05399	1.04702	0.974038	1.0250154	0.431057	0.5029741
E9PNF7	Lysosomal Pro-X carboxypeptidase (Fragment) OS=Homo sapiens GN=PRCP PE=1 SV=1 - [E9PNF7_HUMAN]	28.45	3	3	4	116	13.45962	6.521973	0.98826	1.14901	0.937685	1.0249845	0.732775	0.7811634
D3YTB1	60S ribosomal protein L32 (Fragment) OS=Homo sapiens GN=RPL32 PE=1 SV=1 - [D3YTB1_HUMAN]	18.05	2	2	2	133	15.60672	11.44385	1.10162	1.07783	0.894883	1.02478	0.740875	0.7878925
Q9Y4J8	Dystrobrevin alpha OS=Homo sapiens GN=DTNA PE=1 SV=2 - [DTNA_HUMAN]	8.61	5	5	5	743	83.84795	6.888184	1.05004	1.00763	1.01665	1.0247729	0.194815	0.2641574
P56470	Galectin-4 OS=Homo sapiens GN=LGALS4 PE=1 SV=1 - [LEG4_HUMAN]	17.65	6	6	6	323	35.91819	9.158691	1.00796	1.03318	1.0329	1.0246839	0.0981248	0.1603826
Q6P452	Annexin OS=Homo sapiens GN=ANXA4 PE=1 SV=1 - [Q6P452_HUMAN]	46.49	13	14	24	299	33.53086	5.858887	0.994913	1.03624	1.04223	1.0244604	0.24181	0.3113453
Q9NTK5	Obg-like ATPase 1 OS=Homo sapiens GN=OLA1 PE=1 SV=2 - [OLA1_HUMAN]	6.06	2	2	3	396	44.7153	7.811035	1.10232	1.00762	0.962596	1.02418	0.616511	0.6771219
Q13423	NAD(P) transhydrogenase, mitochondrial OS=Homo sapiens GN=NNT PE=1 SV=3 - [NNTM_HUMAN]	20.44	17	17	22	1086	113.8226	8.089355	1.01685	0.99014	1.06464	1.0238781	0.387507	0.4600881
P62140	Serine/threonine-protein phosphatase PP1-beta catalytic subunit OS=Homo sapiens GN=PPP1CB PE=1 SV=3 - [PP1B_HUMAN]	27.52	2	7	15	327	37.16263	6.188965	1.00218	1.00458	1.06305	1.0232664	0.362866	0.4361866
Q9UF24	Putative uncharacterized protein DKFZp586K0821 (Fragment) OS=Homo sapiens GN=DKFZp586K0821 PE=2 SV=1 -	22.63	3	3	5	190	22.20434	5.478027	0.92481	1.11092	1.03263	1.0227886	0.713817	0.7648846

Biology Open • Supplementary information

	[Q9UF24_HUMAN]													
B4DX19	cDNA FLJ57644, highly similar to Serum paraoxonase/arylesterase 1 (EC 3.1.1.2) OS=Homo sapiens PE=2 SV=1 - [B4DX19_HUMAN]	5.07	2	2	2	355	39.69608	4.881348	0.990054	1.05819	1.01985	1.0227012	0.368742	0.4419942
Q5JTV8	Torsin-1A-interacting protein 1 OS=Homo sapiens GN=TOR1AIP1 PE=1 SV=2 - [TOIP1_HUMAN]	17.84	8	8	9	583	66.20827	8.177246	1.07118	1.00249	0.994346	1.0226734	0.450378	0.5208505
Q96N67	Dedicator of cytokinesis protein 7 OS=Homo sapiens GN=DOCK7 PE=1 SV=4 - [DOCK7_HUMAN]	0.79	2	2	3	2140	242.4074	6.800293	1.0277	1.03667	1.00361	1.0226627	0.148576	0.2148354
E9PJP1	Mth938 domain-containing protein OS=Homo sapiens GN=AAMDC PE=1 SV=1 - [E9PJP1_HUMAN]	26.88	2	2	2	93	10.1191	8.001465	0.979445	1.0309	1.05562	1.0219885	0.430407	0.5025593
B4E1L0	Adenylosuccinate synthetase isozyme 2 OS=Homo sapiens GN=ADSS PE=2 SV=1 - [B4E1L0_HUMAN]	11.72	4	4	5	435	47.90857	5.909668	1.02386	1.06025	0.981807	1.021973	0.434594	0.506403
H0YD13	CD44 antigen OS=Homo sapiens GN=CD44 PE=1 SV=2 - [H0YD13_HUMAN]	30.58	6	6	10	206	22.6686	8.191895	1.0319	1.03277	1.00093	1.0218664	0.171948	0.2394257
P23142	Fibulin-1 OS=Homo sapiens GN=FBLN1 PE=1 SV=4 - [FBLN1_HUMAN]	30.3	5	15	30	703	77.16235	5.224121	0.98856	1.05172	1.0242	1.0214908	0.360776	0.4339825
Q59ET3	Chaperonin containing TCP1, subunit 6A isoform a variant (Fragment) OS=Homo sapiens PE=2 SV=1 - [Q59ET3_HUMAN]	23.44	11	11	18	529	57.72547	6.668457	1.04293	1.10428	0.917204	1.02147	0.734187	0.7822546
E9PLA9	Caprin-1 (Fragment) OS=Homo sapiens GN=CAPRIN1 PE=1 SV=1 - [E9PLA9_HUMAN]	10.22	2	2	2	186	20.22343	7.400879	0.987217	1.02271	1.05445	1.0214601	0.384275	0.457213
A0A087WXS7	ATPase ASNA1 OS=Homo sapiens GN=ASNA1 PE=1 SV=1 - [A0A087WXS7_HUMAN]	13.9	5	5	7	331	37.09472	5.135254	0.972253	1.05494	1.03703	1.0214079	0.483786	0.552814
M0R0F0	40S ribosomal protein S5 (Fragment) OS=Homo sapiens GN=RPS5 PE=1 SV=1 - [M0R0F0_HUMAN]	18	3	3	4	200	22.37678	9.554199	0.749346	1.2067	1.10701	1.0210178	0.893573	0.9168671

Biology Open • Supplementary information

P55084	Trifunctional enzyme subunit beta, mitochondrial OS=Homo sapiens GN=HADHB PE=1 SV=3 - [ECHB_HUMAN]	29.96	14	14	23	474	51.26152	9.407715	1.0015	1.03594	1.02501	1.0208152	0.177018	0.2453751
Q9NZB2	Constitutive coactivator of PPAR-gamma-like protein 1 OS=Homo sapiens GN=FAM120A PE=1 SV=2 - [F120A_HUMAN]	4.2	4	4	4	1118	121.8106	8.880371	1.04598	1.03187	0.984422	1.0207559	0.380917	0.4540155
B3KRM8	Translin, isoform CRA_b OS=Homo sapiens GN=TSN PE=2 SV=1 - [B3KRM8_HUMAN]	13.26	2	2	2	181	20.93804	8.001465	1.02051	1.07985	0.961886	1.0207491	0.604312	0.6650182
B7Z550	Complement component 8, beta polypeptide, isoform CRA_b OS=Homo sapiens GN=C8B PE=2 SV=1 - [B7Z550_HUMAN]	14.56	7	7	10	529	60.10104	7.76709	1.01323	1.01393	1.03357	1.0202422	0.0935299	0.1546647
A8KAH7	cDNA FLJ75444, highly similar to Homo sapiens protein kinase, cAMP-dependent, regulatory, type II, alpha (PRKAR2A), mRNA OS=Homo sapiens PE=2 SV=1 - [A8KAH7_HUMAN]	33.17	9	12	14	404	45.47594	5.071777	1.03756	1.02573	0.995782	1.0196907	0.254077	0.3246699
Q15374	GARS protein OS=Homo sapiens PE=2 SV=1 - [Q15374_HUMAN]	11.78	4	4	4	433	45.95782	6.785645	1.01812	1.03004	1.00666	1.0182751	0.113579	0.1770096
B4DUD4	cDNA FLJ59988, highly similar to Ras-related protein Rab-2B OS=Homo sapiens PE=2 SV=1 - [B4DUD4_HUMAN]	22.35	3	3	4	170	18.99343	7.415527	0.970993	1.06145	1.0218	1.0180839	0.561123	0.6254203
Q53GD1	Guanine nucleotide-binding protein subunit gamma (Fragment) OS=Homo sapiens PE=2 SV=1 - [Q53GD1_HUMAN]	44.44	3	3	7	72	8.029202	8.968262	1.08005	0.934694	1.03931	1.0180205	0.717638	0.7680354
Q96D15	Reticulocalbin-3 OS=Homo sapiens GN=RCN3 PE=1 SV=1 - [RCN3_HUMAN]	16.77	4	4	5	328	37.47	4.894043	1.02238	0.98989	1.04124	1.0178368	0.356333	0.4297069
P14543	Nidogen-1 OS=Homo sapiens GN=NID1 PE=1 SV=3 - [NID1_HUMAN]	24.94	26	27	56	1247	136.2907	5.287598	1.04163	1.03301	0.978635	1.017759	0.462859	0.5323744
A0A024R5W6	Tropomyosin 1 (Alpha), isoform CRA_a OS=Homo sapiens GN=TPM1 PE=3 SV=1 - [A0A024R5W6_HUMAN]	58.45	2	29	116	284	32.65761	4.741699	1.00373	1.03719	1.01188	1.0175989	0.222762	0.2928347

Biology Open • Supplementary information

B4DKJ9	cDNA FLJ60639, highly similar to Mps one binder kinase activator-like 2 OS=Homo sapiens PE=2 SV=1 - [B4DKJ9_HUMAN]	10.84	2	2	2	166	19.48466	7.781738	0.978828	0.970229	1.10284	1.0172978	0.725464	0.7749119
C9JQ42	Glycogenin-1 (Fragment) OS=Homo sapiens GN=GYG1 PE=1 SV=6 - [C9JQ42_HUMAN]	22.64	6	6	8	265	29.85815	6.150879	0.993379	1.04069	1.01673	1.0169306	0.340819	0.4142385
B7Z964	Sarcolemmal membrane-associated protein OS=Homo sapiens GN=SLMAP PE=1 SV=1 - [B7Z964_HUMAN]	19.61	7	7	11	362	42.01078	5.325684	0.957103	1.0309	1.06241	1.0168074	0.644102	0.7017333
Q9H3U1	Protein unc-45 homolog A OS=Homo sapiens GN=UNC45A PE=1 SV=1 - [UN45A_HUMAN]	6.57	5	5	5	944	103.0115	6.074707	1.00676	0.956047	1.08604	1.0162846	0.708789	0.7599781
P51911	Calponin-1 OS=Homo sapiens GN=CNN1 PE=1 SV=2 - [CNN1_HUMAN]	54.21	14	15	46	297	33.14956	9.070801	1.00455	1.02285	1.02069	1.0160264	0.108999	0.1720903
A8K940	cDNA FLJ77630, highly similar to Homo sapiens BPY2 interacting protein 1, mRNA OS=Homo sapiens PE=1 SV=1 - [A8K940_HUMAN]	8.03	6	6	7	1059	112.158	7.29834	1.0341	1.00586	1.0076	1.0158507	0.224908	0.2949685
E5RFJ0	Dedicator of cytokinesis protein 2 OS=Homo sapiens GN=DOCK2 PE=1 SV=1 - [E5RFJ0_HUMAN]	2.52	2	2	2	1031	119.1442	7.181152	1.01082	0.995747	1.0404	1.0156561	0.354965	0.4283002
B4DZ87	cDNA FLJ57240, highly similar to Mitochondrial proteins import receptor OS=Homo sapiens PE=2 SV=1 - [B4DZ87_HUMAN]	8.18	4	4	4	501	56.89454	5.909668	1.02773	1.06425	0.954983	1.0156558	0.674119	0.7281088
C0JYY2	Apolipoprotein B (Including Ag(X) antigen) OS=Homo sapiens GN=APOB PE=4 SV=1 - [C0JYY2_HUMAN]	27.33	103	104	171	4563	515.2359	7.049316	1.02278	1.02867	0.995004	1.0154861	0.274289	0.3467021
A0A087WUS7	Ig delta chain C region OS=Homo sapiens GN=IGHD PE=1 SV=1 - [A0A087WUS7_HUMAN]	15.1	5	5	7	384	42.32632	8.118652	1.08032	1.015	0.950726	1.0153488	0.721378	0.7707906
A0A087WUN7	SRA stem-loop-interacting RNA-binding protein, mitochondrial OS=Homo sapiens GN=SLIRP PE=1 SV=1 - [A0A087WUN7_HUMAN]	26.09	2	2	2	92	10.34635	10.22803	1.0319	0.983119	1.03094	1.0153206	0.441809	0.5130441

Biology Open • Supplementary information

Q13561	Dynactin subunit 2 OS=Homo sapiens GN=DCTN2 PE=1 SV=4 - [DCTN2_HUMAN]	37.41	12	12	19	401	44.20386	5.211426	1.02905	1.00109	1.01469	1.0149436	0.205334	0.2749429
O75533	Splicing factor 3B subunit 1 OS=Homo sapiens GN=SF3B1 PE=1 SV=3 - [SF3B1_HUMAN]	5.21	5	5	6	1304	145.7381	7.093262	1.06184	1.02447	0.958435	1.0149163	0.670584	0.725448
P11766	Alcohol dehydrogenase class-3 OS=Homo sapiens GN=ADH5 PE=1 SV=4 - [ADHX_HUMAN]	30.21	10	10	16	374	39.69838	7.48877	1.04112	1.02221	0.980267	1.0145341	0.503803	0.5722729
B4DZD5	cDNA FLJ60991, highly similar to FKBP12-rapamycin complex-associated protein OS=Homo sapiens PE=2 SV=1 - [B4DZD5_HUMAN]	4.7	5	5	6	1404	157.6421	7.76709	0.979081	0.992706	1.07097	1.0142511	0.667981	0.7233086
A0A024QZE7	Transforming growth factor beta 1 induced transcript 1, isoform CRA_a OS=Homo sapiens GN=TGFB1I1 PE=4 SV=1 - [A0A024QZE7_HUMAN]	30.86	10	10	18	444	47.90994	7.57666	1.00465	0.987417	1.05064	1.0142372	0.529292	0.5966212
J3K000	PEPD protein OS=Homo sapiens GN=PEPD PE=2 SV=1 - [J3K000_HUMAN]	12.98	5	5	6	493	54.54689	5.998535	0.994523	0.979809	1.06744	1.0139257	0.658429	0.7141244
P48668	Keratin, type II cytoskeletal 6C OS=Homo sapiens GN=KRT6C PE=1 SV=3 - [K2C6C_HUMAN]	40.78	2	23	34	564	59.98831	8.001465	1.0072	1.06422	0.969197	1.0135388	0.672442	0.7269934
Q5T626	Nuclear autoantigenic sperm protein (Histone-binding) OS=Homo sapiens GN=NASP PE=2 SV=1 - [Q5T626_HUMAN]	6.01	2	2	2	449	48.77457	4.398926	0.988741	1.10323	0.947512	1.0131616	0.804079	0.842404
E9PGT6	COP9 signalosome complex subunit 8 OS=Homo sapiens GN=COPS8 PE=1 SV=1 - [E9PGT6_HUMAN]	16.18	2	2	2	173	19.33194	5.528809	1.01617	0.901763	1.12135	1.0130918	0.855533	0.8863595
M0R2S2	Epidermal growth factor receptor substrate 15-like 1 OS=Homo sapiens GN=EPS15L1 PE=1 SV=1 - [M0R2S2_HUMAN]	13.87	4	4	5	447	49.82629	5.135254	1.08397	0.962303	0.992857	1.0130437	0.755286	0.8005726
B7ZAC5	cDNA, FLJ79139, highly similar to Alkyldihydroxyacetonephosphate synthase, peroxisomal (EC	11.25	2	2	2	320	35.57765	6.455566	1.00088	1.08005	0.957501	1.0128107	0.755187	0.8005726

Biology Open • Supplementary information

	2.5.1.26) OS=Homo sapiens PE=2 SV=1 - [B7ZAC5_HUMAN]													
B2RB23	cDNA, FLJ95265, highly similar to Homo sapiens acetyl-Coenzyme A acyltransferase 2 (mitochondrial 3-oxoacyl-Coenzyme A thiolase) (ACAA2), nuclear gene encoding mitochondrial protein, mRNA OS=Homo sapiens PE=2 SV=1 - [B2RB23_HUMAN]	19.9	6	6	8	397	41.98271	8.250488	1.00128	1.00307	1.03399	1.0127806	0.351792	0.4253567
E5RIW3	Tubulin-specific chaperone A OS=Homo sapiens GN=TBCA PE=1 SV=1 - [E5RIW3_HUMAN]	23.81	2	2	2	84	10.07401	4.627441	0.973665	1.03539	1.029	1.0126838	0.583843	0.6458525
Q6NVY0	Calcyclin binding protein OS=Homo sapiens GN=CACYBP PE=2 SV=1 - [Q6NVY0_HUMAN]	7.02	2	2	2	228	26.1656	7.869629	0.897862	1.07887	1.06083	1.0125209	0.847987	0.880905
B4E1Z4	Uncharacterized protein OS=Homo sapiens PE=1 SV=1 - [B4E1Z4_HUMAN]	20.62	20	23	29	1266	140.8528	7.181152	1.00384	1.06345	0.969678	1.0123227	0.696926	0.7486812
Q9Y281	Cofilin-2 OS=Homo sapiens GN=CFL2 PE=1 SV=1 - [COF2_HUMAN]	54.82	4	8	25	166	18.72485	7.884277	1.03385	1.00741	0.995203	1.0121524	0.398215	0.4709846
Q7Z524	HUMEEP OS=Homo sapiens PE=2 SV=1 - [Q7Z524_HUMAN]	3.75	2	2	2	453	50.47903	6.252441	1.0642	1.30652	0.665497	1.012073	0.954365	0.9637606
B4DHV5	cDNA FLJ51852, highly similar to Homo sapiens ADP-ribosylhydrolase like 2 (ADPRHL2), mRNA OS=Homo sapiens PE=2 SV=1 - [B4DHV5_HUMAN]	8.13	2	2	2	283	30.85434	5.186035	1.02453	1.01899	0.992068	1.011863	0.358298	0.4314617
E9PJ77	Galectin (Fragment) OS=Homo sapiens GN=LGALS8 PE=1 SV=1 - [E9PJ77_HUMAN]	21.7	2	2	2	106	12.09919	7.29834	0.981633	1.01506	1.03762	1.0114383	0.554721	0.6197131
A0A0B4J1W3	N-alpha-acetyltransferase 15, NatA auxiliary subunit OS=Homo sapiens GN=NAA15 PE=1 SV=1 - [A0A0B4J1W3_HUMAN]	2.08	2	2	2	865	101.1367	7.415527	1.02742	0.979689	1.0269	1.0113363	0.548108	0.6137745
Q9NX63	MICOS complex subunit MIC19 OS=Homo sapiens	19.38	4	4	4	227	26.13623	8.279785	1.04229	1.0344	0.957024	1.0112385	0.719588	0.7693637

Biology Open • Supplementary information

	GN=CHCHD3 PE=1 SV=1 - [MIC19_HUMAN]													
Q6U2L6	C4B (Fragment) OS=Homo sapiens GN=C4B PE=4 SV=1 - [Q6U2L6_HUMAN]	30	2	4	5	100	10.24625	6.521973	1.03719	1.05272	0.943659	1.0111904	0.773718	0.8156448
Q2NLD4	PURA protein (Fragment) OS=Homo sapiens GN=PURA PE=2 SV=1 - [Q2NLD4_HUMAN]	31.58	6	7	8	285	31.99523	7.049316	0.976369	1.07556	0.9816	1.0111776	0.761817	0.8056554
P61970	Nuclear transport factor 2 OS=Homo sapiens GN=NUTF2 PE=1 SV=1 - [NTF2_HUMAN]	51.18	4	4	8	127	14.46905	5.376465	1.05967	1.05338	0.920264	1.011104	0.829789	0.8650557
A8K7B1	cDNA FLJ78384, highly similar to Homo sapiens RUN and FYVE domain containing 1 (RUFY1), mRNA OS=Homo sapiens PE=2 SV=1 - [A8K7B1_HUMAN]	6	3	3	3	600	69.02005	5.909668	0.917454	0.964061	1.15167	1.0110625	0.891366	0.9148799
A8MZF9	Developmentally-regulated GTP-binding protein 2 OS=Homo sapiens GN=DRG2 PE=1 SV=1 - [A8MZF9_HUMAN]	13.7	3	4	4	343	38.10504	8.909668	1.12715	0.946558	0.95936	1.0110228	0.86722	0.8947088
H3BNR4	AP-1 complex subunit gamma-1 OS=Homo sapiens GN=AP1G1 PE=1 SV=1 - [H3BNR4_HUMAN]	8.05	2	2	3	236	27.04989	8.836426	0.911268	0.985375	1.13596	1.0108672	0.88453	0.9095182
Q4QQP8	PTGFRN protein (Fragment) OS=Homo sapiens GN=PTGFRN PE=2 SV=1 - [Q4QQP8_HUMAN]	7.36	5	5	5	788	88.22317	6.653809	1.08646	0.961665	0.984323	1.0108158	0.804589	0.8426775
Q05CM9	PSIP1 protein (Fragment) OS=Homo sapiens GN=PSIP1 PE=2 SV=1 - [Q05CM9_HUMAN]	25.1	5	6	7	263	29.65243	9.393066	1.01925	0.982888	1.02998	1.0107055	0.530855	0.5979848
P30050	60S ribosomal protein L12 OS=Homo sapiens GN=RPL12 PE=1 SV=1 - [RL12_HUMAN]	49.7	6	6	11	165	17.80754	9.422363	0.991356	0.967003	1.07355	1.010638	0.772746	0.8148739
V9HW35	Epididymis secretory protein Li 55 OS=Homo sapiens GN=HEL-S-55 PE=2 SV=1 - [V9HW35_HUMAN]	41.36	5	5	14	162	17.02001	7.239746	0.999372	1.06913	0.96249	1.0103297	0.772519	0.8148739
F6V6Z1	Mitochondrial amidoxime reducing component 2 OS=Homo sapiens GN=MARC2 PE=1 SV=1 - [F6V6Z1_HUMAN]	32.98	3	3	3	94	10.19348	11.81006	1.02188	1.00699	1.00171	1.0101913	0.233608	0.3028583
G3V0I5	NADH dehydrogenase (Ubiquinone) flavoprotein 1, 51kDa,	15.97	6	6	7	457	50.02243	8.206543	0.990328	1.02552	1.01353	1.0097948	0.443138	0.5142345

	isoform CRA_c OS=Homo sapiens GN=NDUFV1 PE=1 SV=1 - [G3V0I5_HUMAN]													
Q12797	Aspartyl/asparaginyl beta-hydroxylase OS=Homo sapiens GN=ASPH PE=1 SV=3 - [ASPH_HUMAN]	18.07	10	10	14	758	85.80945	5.008301	1.01327	1.07664	0.939362	1.0097571	0.828639	0.8641229
E9PDF6	Unconventional myosin-Ib OS=Homo sapiens GN=MYO1B PE=1 SV=1 - [E9PDF6_HUMAN]	6.78	5	5	6	1107	128.4	9.305176	1.0067	1.0364	0.984474	1.0091924	0.603326	0.6641491
P51148	Ras-related protein Rab-5C OS=Homo sapiens GN=RAB5C PE=1 SV=2 - [RAB5C_HUMAN]	48.61	5	7	14	216	23.46779	8.411621	0.989817	1.03767	0.999711	1.0090664	0.597602	0.6585842
P26447	Protein S100-A4 OS=Homo sapiens GN=S100A4 PE=1 SV=1 - [S10A4_HUMAN]	45.54	6	6	15	101	11.72073	6.112793	0.967275	1.05578	1.00401	1.0090219	0.75883	0.8034531
Q6YHK3	CD109 antigen OS=Homo sapiens GN=CD109 PE=1 SV=2 - [CD109_HUMAN]	3.74	4	4	5	1445	161.5873	5.846191	0.965367	1.01977	1.04038	1.0085047	0.740431	0.7876678
O43143	Pre-mRNA-splicing factor ATP-dependent RNA helicase DHX15 OS=Homo sapiens GN=DHX15 PE=1 SV=2 - [DHX15_HUMAN]	10.82	7	7	9	795	90.87517	7.459473	1.06443	0.976428	0.983873	1.0082435	0.797406	0.8367078
P11177	Pyruvate dehydrogenase E1 component subunit beta, mitochondrial OS=Homo sapiens GN=PDHB PE=1 SV=3 - [ODPB_HUMAN]	19.22	6	6	7	359	39.20803	6.653809	0.984889	1.06379	0.975028	1.0079015	0.804903	0.8427456
Q08257	Quinone oxidoreductase OS=Homo sapiens GN=CRYZ PE=1 SV=1 - [QOR_HUMAN]	28.88	7	7	10	329	35.18455	8.440918	1.0111	0.998253	1.01434	1.0078982	0.249135	0.319279
C9J0K6	Sorcin OS=Homo sapiens GN=SRI PE=1 SV=1 - [C9J0K6_HUMAN]	46.45	6	6	6	155	17.59351	5.60498	0.974376	1.05951	0.989596	1.007828	0.793391	0.8334492
A6NKB8	Aminopeptidase B OS=Homo sapiens GN=RNPEP PE=1 SV=1 - [A6NKB8_HUMAN]	20.29	10	10	13	611	68.12527	6.01123	0.981319	0.999182	1.04227	1.0075916	0.71554	0.7664883
Q9UJU6	Drebrin-like protein OS=Homo sapiens GN=DBNL PE=1 SV=1 - [DBNL_HUMAN]	16.98	5	5	7	430	48.17757	5.046387	1.07305	0.879353	1.06824	1.0068808	0.923935	0.9400421

Biology Open • Supplementary information

Q14240	Eukaryotic initiation factor 4A-II OS=Homo sapiens GN=EIF4A2 PE=1 SV=2 - [IF4A2_HUMAN]	30.22	4	10	24	407	46.37278	5.478027	0.974682	1.00432	1.04079	1.0065994	0.762885	0.8064852
B4DMP4	cDNA FLJ53136, highly similar to Homo sapiens Vac14 homolog (VAC14), mRNA OS=Homo sapiens PE=2 SV=1 - [B4DMP4_HUMAN]	7.02	3	3	6	712	80.09036	5.706543	1.01563	1.03182	0.972047	1.0064967	0.750731	0.7971218
B4E0H8	cDNA FLJ60385, highly similar to Integrin alpha-3 OS=Homo sapiens PE=2 SV=1 - [B4E0H8_HUMAN]	8.29	7	7	8	1037	115.0942	6.565918	0.964504	0.98446	1.07007	1.0063435	0.862774	0.8909365
O15400	Syntaxin-7 OS=Homo sapiens GN=STX7 PE=1 SV=4 - [STX7_HUMAN]	16.09	3	3	4	261	29.79732	5.554199	1.06821	0.932603	1.01788	1.0062308	0.889359	0.9139284
P84157	Matrix-remodeling-associated protein 7 OS=Homo sapiens GN=MXRA7 PE=1 SV=1 - [MXRA7_HUMAN]	23.04	4	4	6	204	21.4524	4.259277	1.04969	1.03932	0.929094	1.0060343	0.890088	0.9142478
A8K492	cDNA FLJ76789, highly similar to Homo sapiens methionine-tRNA synthetase (MARS), mRNA OS=Homo sapiens PE=2 SV=1 - [A8K492_HUMAN]	13.78	8	8	10	900	101.0799	6.163574	1.05125	1.01289	0.953896	1.0060109	0.851545	0.8836542
P46783	40S ribosomal protein S10 OS=Homo sapiens GN=RPS10 PE=1 SV=1 - [RS10_HUMAN]	18.79	2	4	4	165	18.88587	10.15479	1.02263	1.00715	0.987385	1.0057238	0.631168	0.6903269
P63000	Ras-related C3 botulinum toxin substrate 1 OS=Homo sapiens GN=RAC1 PE=1 SV=1 - [RAC1_HUMAN]	34.9	4	7	19	192	21.43624	8.499512	0.972484	1.06249	0.981913	1.0056274	0.862002	0.890411
Q9HDC9	Adipocyte plasma membrane-associated protein OS=Homo sapiens GN=APMAP PE=1 SV=2 - [APMAP_HUMAN]	22.84	8	8	9	416	46.45085	6.163574	1.07245	0.903493	1.04088	1.0056078	0.923768	0.9400421
A0A024RDQ0	Heat shock 105kDa/110kDa protein 1, isoform CRA_a OS=Homo sapiens GN=HSPH1 PE=3 SV=1 - [A0A024RDQ0_HUMAN]	13.27	7	8	10	814	92.05745	5.554199	0.975341	1.02443	1.01663	1.0054652	0.754041	0.8001344
H7BYX6	Neural cell adhesion molecule 1 OS=Homo sapiens GN=NCAM1 PE=1 SV=2 - [H7BYX6_HUMAN]	4.14	3	3	5	725	80.23313	4.881348	0.992032	1.04025	0.983515	1.0052656	0.793744	0.8334492
Q9Y5B2	Junction adhesion molecule OS=Homo sapiens PE=2 SV=1 -	13.13	2	2	2	259	28.10426	8.294434	1.06709	1.01612	0.930841	1.0046831	0.916971	0.9357693

Biology Open • Supplementary information

	[Q9Y5B2_HUMAN]													
Q13011	Delta(3,5)-Delta(2,4)-dienoyl-CoA isomerase, mitochondrial OS=Homo sapiens GN=ECH1 PE=1 SV=2 - [ECH1_HUMAN]	28.35	8	8	13	328	35.79338	8.001465	1.07916	0.965535	0.969073	1.0045884	0.913341	0.9329087
O94973	AP-2 complex subunit alpha-2 OS=Homo sapiens GN=AP2A2 PE=1 SV=2 - [AP2A2_HUMAN]	15.34	5	12	15	939	103.895	6.961426	0.987554	1.00159	1.02311	1.0040848	0.730947	0.7797843
B4E1D8	cDNA FLJ51597, highly similar to C4b-binding protein alpha chain OS=Homo sapiens PE=2 SV=1 - [B4E1D8_HUMAN]	18.66	9	9	18	536	60.3658	6.653809	1.00408	1.0526	0.95554	1.0040728	0.897757	0.9200446
P62857	40S ribosomal protein S28 OS=Homo sapiens GN=RPS28 PE=1 SV=1 - [RS28_HUMAN]	36.23	3	3	6	69	7.836202	10.69678	1.01445	0.92418	1.07323	1.0039544	0.935626	0.9482317
A0A024R972	Laminin, gamma 1 (Formerly LAMB2), isoform CRA_a OS=Homo sapiens GN=LAMC1 PE=4 SV=1 - [A0A024R972_HUMAN]	32.49	46	46	87	1573	173.8996	5.046387	0.999947	1.02888	0.98283	1.0038859	0.799676	0.8388296
P49327	Fatty acid synthase OS=Homo sapiens GN=FASN PE=1 SV=3 - [FAS_HUMAN]	13.02	25	25	28	2511	273.2543	6.442871	1.02177	1.0396	0.949818	1.0037285	0.904367	0.9256977
Q5XKP0	MICOS complex subunit MIC13 OS=Homo sapiens GN=MIC13 PE=1 SV=1 - [MIC13_HUMAN]	35.59	2	2	3	118	13.07876	9.422363	0.935655	1.10956	0.965551	1.0035894	0.952775	0.9624421
P14868	Aspartate--tRNA ligase, cytoplasmic OS=Homo sapiens GN=DARS PE=1 SV=2 - [SYDC_HUMAN]	27.15	10	10	12	501	57.10004	6.55127	1.01441	0.973884	1.02233	1.0035433	0.835302	0.8699992
Q15008	26S proteasome non-ATPase regulatory subunit 6 OS=Homo sapiens GN=PSMD6 PE=1 SV=1 - [PSMD6_HUMAN]	9.77	3	3	3	389	45.50228	5.617676	1.02119	1.0561	0.932954	1.0034136	0.934269	0.9481775
P23526	Adenosylhomocysteinase OS=Homo sapiens GN=AHCY PE=1 SV=4 - [SAHH_HUMAN]	16.2	6	6	9	432	47.68521	6.341309	1.02565	1.03744	0.946569	1.003219	0.920467	0.9383646
A8K525	cDNA FLJ76817, highly similar to Homo sapiens non-POU domain containing, octamer-binding (NONO), mRNA OS=Homo sapiens PE=2 SV=1 - [A8K525_HUMAN]	20.59	7	8	15	471	54.25337	9.202637	0.988778	1.02301	0.997505	1.0030961	0.791489	0.8317884
P30085	UMP-CMP kinase OS=Homo sapiens GN=CMPK1 PE=1	18.88	3	3	6	196	22.20828	5.566895	0.936864	1.09333	0.978322	1.0028394	0.957141	0.9656996

Biology Open • Supplementary information

	SV=3 - [KCY_HUMAN]														
P53618	Coatomer subunit beta OS=Homo sapiens GN=COPB1 PE=1 SV=3 - [COPB_HUMAN]	16.47	11	11	16	953	107.0738	6.049316	1.01509	1.01979	0.973077	1.0026519	0.874714	0.9007931
Q9BTV4	Transmembrane protein 43 OS=Homo sapiens GN=TMEM43 PE=1 SV=1 - [TMM43_HUMAN]	19.75	7	7	14	400	44.8472	8.133301	0.9799	1.00327	1.02453	1.0025655	0.860624	0.8895305
B7Z5S1	cDNA FLJ61699, highly similar to Homo sapiens androgen-induced proliferation inhibitor (APRIN), transcript variant 1, mRNA OS=Homo sapiens PE=2 SV=1 - [B7Z5S1_HUMAN]	4.22	5	5	6	1302	148.6452	8.060059	1.00504	0.926604	1.07541	1.0023505	0.961356	0.9687973
B9EJA8	Mannose receptor, C type 1-like 1 OS=Homo sapiens GN=MRC1L1 PE=2 SV=1 - [B9EJA8_HUMAN]	6.87	8	8	9	1456	165.9177	6.507324	1.03567	1.02428	0.946995	1.0023142	0.941354	0.9528957
Q99584	Protein S100-A13 OS=Homo sapiens GN=S100A13 PE=1 SV=1 - [S10AD_HUMAN]	23.47	2	2	4	98	11.46409	6.163574	1.06334	0.978776	0.964145	1.0020875	0.952311	0.9622605
Q7Z503	Succinyl-CoA ligase subunit beta OS=Homo sapiens PE=2 SV=1 - [Q7Z503_HUMAN]	16.87	5	5	7	403	43.58274	5.57959	1.0269	0.979019	1.00029	1.0020703	0.894895	0.9176672
B2RAH5	Protein phosphatase 1 regulatory subunit OS=Homo sapiens PE=2 SV=1 - [B2RAH5_HUMAN]	9.42	7	7	8	1030	115.2594	5.427246	0.975276	1.04065	0.98991	1.001946	0.930701	0.9449379
A0M8Q2	Phosphatase 2C motif OS=Homo sapiens PE=3 SV=1 - [A0M8Q2_HUMAN]	9.87	3	3	3	385	42.5776	5.516113	1.074	0.927105	1.00406	1.0017212	0.971322	0.9776762
Q15124	Phosphoglucomutase-like protein 5 OS=Homo sapiens GN=PGM5 PE=1 SV=2 - [PGM5_HUMAN]	39.86	19	19	37	567	62.1857	7.210449	0.971682	1.03578	0.997156	1.0015387	0.941706	0.9529671
E9PKG6	Nucleobindin-2 OS=Homo sapiens GN=NUCB2 PE=1 SV=2 - [E9PKG6_HUMAN]	8.28	2	2	2	338	40.34334	5.17334	1.01523	1.00907	0.979429	1.0012446	0.920623	0.9383646
A8K3A8	cDNA FLJ75085, highly similar to Homo sapiens glutaminyl-tRNA synthetase (QARS), mRNA OS=Homo sapiens PE=2 SV=1 - [A8K3A8_HUMAN]	12.65	8	8	11	775	87.7529	7.151855	1.00776	0.927704	1.06812	1.0011934	0.979254	0.9841968

Biology Open • Supplementary information

A7E2D8	Calcium-transporting ATPase OS=Homo sapiens GN=ATP2B4 PE=2 SV=1 - [A7E2D8_HUMAN]	12.71	8	12	14	1164	128.7494	7.38623	0.980482	0.928799	1.09346	1.0009126	0.986728	0.9902385
Q03154	Aminoacylase-1 OS=Homo sapiens GN=ACY1 PE=1 SV=1 - [ACY1_HUMAN]	6.86	2	2	2	408	45.85599	6.17627	1.04412	1.02353	0.934274	1.0006417	0.98654	0.9902385
E9PR16	Nuclear pore complex protein Nup160 (Fragment) OS=Homo sapiens GN=NUP160 PE=1 SV=2 - [E9PR16_HUMAN]	2.05	2	2	2	1123	127.3571	5.427246	0.926944	0.924685	1.15027	1.0006341	0.994007	0.995183
A8K477	Sulfhydryl oxidase OS=Homo sapiens PE=2 SV=1 - [A8K477_HUMAN]	4.8	2	2	3	604	66.76833	8.602051	1.04584	0.813545	1.14245	1.0006138	0.995553	0.9964361
E9PDQ5	Rho guanine nucleotide exchange factor 7 OS=Homo sapiens GN=ARHGEF7 PE=1 SV=1 - [E9PDQ5_HUMAN]	8.04	3	3	4	547	61.59085	7.532715	1.003	1.0576	0.941147	1.0005835	0.987736	0.9905908
B4DQ92	cDNA FLJ59379, highly similar to Hematopoietic lineage cell-specific protein OS=Homo sapiens PE=2 SV=1 - [B4DQ92_HUMAN]	7.35	2	2	2	449	49.58592	4.754395	1.0165	0.939649	1.04495	1.0003662	0.991766	0.9932331
F5H0N8	Tyrosine-protein phosphatase non-receptor type 6 (Fragment) OS=Homo sapiens GN=PTPN6 PE=1 SV=1 - [F5H0N8_HUMAN]	24.24	3	3	4	165	18.45429	6.800293	0.974101	1.04	0.986254	1.000117	0.995916	0.9965048
P49961	Ectonucleoside triphosphate diphosphohydrolase 1 OS=Homo sapiens GN=ENTPD1 PE=1 SV=1 - [ENTP1_HUMAN]	10.98	6	6	10	510	57.92711	6.341309	0.975148	1.00136	1.02334	0.9999491	0.997417	0.997417
Q4W5L2	Alpha-synuclein (Fragment) OS=Homo sapiens GN=SNCA PE=3 SV=1 - [Q4W5L2_HUMAN]	46.08	3	3	3	102	10.1835	9.524902	0.979831	1.00032	1.01927	0.999806	0.987957	0.9905908
P50995	Annexin A11 OS=Homo sapiens GN=ANXA11 PE=1 SV=1 - [ANX11_HUMAN]	25.54	10	10	16	505	54.35507	7.649902	0.968296	1.07611	0.954992	0.9997998	0.996309	0.9966034
Q5VZR0	Golgi-associated plant pathogenesis-related protein 1 OS=Homo sapiens GN=GLIPR2 PE=1 SV=1 - [Q5VZR0_HUMAN]	33.59	3	3	3	128	14.20411	9.510254	1.01007	1.10205	0.883655	0.998591	0.984263	0.988644

Biology Open • Supplementary information

Q9NZ45	CDGSH iron-sulfur domain-containing protein 1 OS=Homo sapiens GN=CISD1 PE=1 SV=1 - [CISD1_HUMAN]	34.26	3	3	6	108	12.19121	9.085449	1.01492	1.0231	0.957198	0.9984067	0.945758	0.9564956
Q14112	Nidogen-2 OS=Homo sapiens GN=NID2 PE=1 SV=3 - [NID2_HUMAN]	13.53	16	17	25	1375	151.1583	5.287598	0.990884	0.97639	1.02706	0.998112	0.911744	0.9317721
Q5NV90	V2-17 protein (Fragment) OS=Homo sapiens GN=V2-17 PE=1 SV=1 - [Q5NV90_HUMAN]	41.24	3	3	7	97	10.39894	4.589355	0.994496	0.963516	1.03511	0.9977091	0.922099	0.9393034
Q53GW1	Vesicle transport-related protein isoform a variant (Fragment) OS=Homo sapiens PE=2 SV=1 - [Q53GW1_HUMAN]	10.44	4	4	4	642	72.26203	6.379395	0.974827	1.01563	1.00084	0.9970999	0.83055	0.8655824
Q96FJ2	Dynein light chain 2, cytoplasmic OS=Homo sapiens GN=DYNLL2 PE=1 SV=1 - [DYL2_HUMAN]	50.56	3	4	6	89	10.34307	7.371582	1.0049	1.04366	0.942478	0.9970115	0.928487	0.94365
P07108	Acyl-CoA-binding protein OS=Homo sapiens GN=DBI PE=1 SV=2 - [ACBP_HUMAN]	41.38	2	2	5	87	10.03802	6.565918	1.01286	0.901651	1.07607	0.9968631	0.956534	0.965375
Q8NBX0	Saccharopine dehydrogenase-like oxidoreductase OS=Homo sapiens GN=SCCPDH PE=1 SV=1 - [SCPDL_HUMAN]	9.32	3	3	4	429	47.12145	9.144043	0.987262	0.993495	1.00908	0.9966125	0.653672	0.7103306
P35749	Myosin-11 OS=Homo sapiens GN=MYH11 PE=1 SV=3 - [MYH11_HUMAN]	60.5	45	141	475	1972	227.1989	5.503418	0.967256	1.02901	0.993487	0.9965828	0.866169	0.893897
P05141	ADP/ATP translocase 2 OS=Homo sapiens GN=SLC25A5 PE=1 SV=7 - [ADT2_HUMAN]	22.82	2	8	15	298	32.83115	9.686035	1.0072	0.980521	1.00154	0.9964214	0.702347	0.7537871
Q8NCW5	NAD(P)H-hydrate epimerase OS=Homo sapiens GN=APOA1BP PE=1 SV=2 - [NNRE_HUMAN]	17.01	4	4	6	288	31.65425	7.664551	0.935427	1.02064	1.03293	0.9963323	0.91571	0.9347643
E7EX90	Dynactin subunit 1 OS=Homo sapiens GN=DCTN1 PE=1 SV=1 - [E7EX90_HUMAN]	13.38	15	15	18	1256	139.0078	5.668457	0.97828	0.993482	1.01678	0.996181	0.765525	0.808771
O75131	Copine-3 OS=Homo sapiens GN=CPNE3 PE=1 SV=1 - [CPNE3_HUMAN]	18.44	6	7	9	537	60.09213	5.846191	1.05172	0.981426	0.954795	0.9959792	0.902127	0.9236842
B2R825	Alpha-1,4 glucan phosphorylase OS=Homo sapiens PE=2 SV=1 - [B2R825_HUMAN]	15.11	10	12	14	847	97.00596	7.29834	0.989621	1.00576	0.992381	0.9959207	0.499036	0.5676199

Biology Open • Supplementary information

H7BZN1	Choline-phosphate cytidylyltransferase A (Fragment) OS=Homo sapiens GN=PCYT1A PE=1 SV=1 - [H7BZN1_HUMAN]	19.71	2	2	2	137	15.75975	6.277832	1.04548	0.991866	0.949728	0.9956909	0.890686	0.9144592
A0A0G3IFT0	Mutant methylmalonyl CoA mutase (Fragment) OS=Homo sapiens GN=MUT PE=4 SV=1 - [A0A0G3IFT0_HUMAN]	21.14	2	2	3	123	13.44781	4.79248	0.913758	1.009	1.06395	0.9955685	0.928759	0.94365
P35900	Keratin, type I cytoskeletal 20 OS=Homo sapiens GN=KRT20 PE=1 SV=1 - [K1C20_HUMAN]	13.92	4	6	8	424	48.45692	5.693848	0.99688	0.936418	1.05175	0.9950153	0.894758	0.9176672
B3KM48	cDNA FLJ10286 fis, clone HEMBB1001384, highly similar to COP9 signalosome complex subunit 4 OS=Homo sapiens PE=2 SV=1 - [B3KM48_HUMAN]	5.91	2	2	2	406	46.22566	5.833496	1.01067	1.02542	0.948035	0.9947075	0.844163	0.8778777
Q6XQN6	Nicotinate phosphoribosyltransferase OS=Homo sapiens GN=NAPRT PE=1 SV=2 - [PNCB_HUMAN]	11.15	4	4	5	538	57.54202	5.681152	0.892297	1.03105	1.0598	0.9943843	0.92344	0.9400421
P63173	60S ribosomal protein L38 OS=Homo sapiens GN=RPL38 PE=1 SV=2 - [RL38_HUMAN]	31.43	2	2	2	70	8.212732	10.09619	1.05338	0.978561	0.950818	0.9942525	0.868463	0.8957182
H3BLU7	Aflatoxin B1 aldehyde reductase member 2 (Fragment) OS=Homo sapiens GN=AKR7A2 PE=1 SV=1 - [H3BLU7_HUMAN]	16.24	4	4	8	314	34.66225	7.181152	0.983175	0.994704	1.00456	0.9941454	0.443382	0.5143414
B3KNJ3	cDNA FLJ14684 fis, clone NT2RP2004933, highly similar to Death-associated protein kinase 3 (EC 2.7.11.1) OS=Homo sapiens PE=2 SV=1 - [B3KNJ3_HUMAN]	4.85	2	2	2	454	52.47337	6.888184	0.996723	0.933819	1.05118	0.9939084	0.873984	0.9005893
A0A024RBR1	Restin (Reed-Steinberg cell-expressed intermediate filament-associated protein), isoform CRA_b OS=Homo sapiens GN=RSN PE=4 SV=1 - [A0A024RBR1_HUMAN]	3.85	4	4	4	1427	160.8911	5.38916	0.955101	1.04567	0.98083	0.9938675	0.841115	0.8749767
P38919	Eukaryotic initiation factor 4A-III OS=Homo sapiens GN=EIF4A3 PE=1 SV=4 - [IF4A3_HUMAN]	15.09	3	5	9	411	46.84118	6.727051	1.02072	0.877023	1.08368	0.9938064	0.928569	0.94365

P55001	Microfibrillar-associated protein 2 OS=Homo sapiens GN=MFAP2 PE=2 SV=1 - [MFAP2_HUMAN]	20.22	3	3	6	183	20.81185	4.970215	1.02183	0.96496	0.994354	0.9937135	0.738669	0.7860404
P01833	Polymeric immunoglobulin receptor OS=Homo sapiens GN=PIGR PE=1 SV=4 - [PIGR_HUMAN]	17.02	10	10	11	764	83.23166	5.744629	0.934861	1.00297	1.04247	0.9934323	0.853819	0.885471
O94776	Metastasis-associated protein MTA2 OS=Homo sapiens GN=MTA2 PE=1 SV=1 - [MTA2_HUMAN]	2.84	2	2	2	668	74.97574	9.656738	0.967481	1.03956	0.973085	0.9933744	0.801632	0.8403606
P25311	Zinc-alpha-2-glycoprotein OS=Homo sapiens GN=AZGP1 PE=1 SV=2 - [ZA2G_HUMAN]	45.64	11	11	19	298	34.23711	6.049316	1.0041	0.999666	0.976349	0.9933703	0.521653	0.589778
P25398	40S ribosomal protein S12 OS=Homo sapiens GN=RPS12 PE=1 SV=3 - [RS12_HUMAN]	53.79	7	7	16	132	14.50548	7.210449	0.990077	0.996011	0.993707	0.9932651	0.059923	0.1145985
P07305	Histone H1.0 OS=Homo sapiens GN=H1F0 PE=1 SV=3 - [H10_HUMAN]	21.13	5	5	16	194	20.8502	10.84326	0.985433	1.02728	0.967082	0.9932647	0.74171	0.7885328
O14828	Secretory carrier-associated membrane protein 3 OS=Homo sapiens GN=SCAMP3 PE=1 SV=3 - [SCAM3_HUMAN]	8.65	2	2	2	347	38.26233	7.635254	1.08916	0.847504	1.04289	0.9931852	0.935067	0.9482317
A0A0A1TSG4	CD47 OS=Homo sapiens GN=CD47 PE=2 SV=1 - [A0A0A1TSG4_HUMAN]	6.48	2	2	3	293	31.85107	6.741699	0.972077	0.982081	1.02507	0.9930755	0.711575	0.7627237
B4DJ63	Serine hydroxymethyltransferase OS=Homo sapiens PE=2 SV=1 - [B4DJ63_HUMAN]	11.9	4	4	4	378	41.6308	9.524902	1.06236	0.976296	0.93875	0.9924695	0.855985	0.8863595
A0A024R035	Complement component 9, isoform CRA_a OS=Homo sapiens GN=C9 PE=4 SV=1 - [A0A024R035_HUMAN]	18.07	9	9	17	559	63.16268	5.516113	0.972466	1.04349	0.961103	0.9923522	0.794677	0.8341028
Q92973	Transportin-1 OS=Homo sapiens GN=TNPO1 PE=1 SV=2 - [TNPO1_HUMAN]	12.25	5	8	10	898	102.2888	4.98291	0.96577	1.04922	0.962027	0.9923397	0.813042	0.8502154
P05090	Apolipoprotein D OS=Homo sapiens GN=APOD PE=1 SV=1 - [APOD_HUMAN]	18.52	3	3	6	189	21.26176	5.147949	1.04292	1.03505	0.897987	0.9919862	0.880437	0.9061354
P21964	Catechol O-methyltransferase OS=Homo sapiens GN=COMT PE=1 SV=2 - [COMT_HUMAN]	23.62	5	5	7	271	30.01763	5.465332	1.05446	0.924068	0.99728	0.9919364	0.850597	0.8832119

Biology Open • Supplementary information

A8K6V3	cDNA FLJ78677, highly similar to Homo sapiens splicing factor 3b, subunit 3, 130kDa (SF3B3), mRNA OS=Homo sapiens PE=2 SV=1 - [A8K6V3_HUMAN]	3.7	3	4	5	1217	135.4927	5.211426	1.0727	1.08771	0.81537	0.9919258	0.935537	0.9482317
Q8N1G4	Leucine-rich repeat-containing protein 47 OS=Homo sapiens GN=LRRC47 PE=1 SV=1 - [LRC47_HUMAN]	17.15	8	8	10	583	63.43374	8.279785	1.05886	0.997087	0.919203	0.991715	0.856504	0.8866257
Q9BQI0	Allograft inflammatory factor 1-like OS=Homo sapiens GN=AIF1L PE=1 SV=1 - [AIF1L_HUMAN]	22	3	3	3	150	17.05662	7.195801	1.07318	0.939977	0.961001	0.9913872	0.854276	0.8856736
P46109	Crk-like protein OS=Homo sapiens GN=CRKL PE=1 SV=1 - [CRKL_HUMAN]	16.83	4	4	5	303	33.75592	6.741699	0.947617	0.973445	1.05254	0.9912017	0.806619	0.8442812
P54920	Alpha-soluble NSF attachment protein OS=Homo sapiens GN=NAPA PE=1 SV=3 - [SNAA_HUMAN]	26.78	6	6	6	295	33.21128	5.36377	0.928675	1.07875	0.965333	0.9909181	0.859248	0.8889225
Q6ZMI0	Protein phosphatase 1 regulatory subunit 21 OS=Homo sapiens GN=PPP1R21 PE=1 SV=1 - [PPR21_HUMAN]	6.67	3	3	3	780	88.25953	6.844238	0.950307	0.91331	1.10835	0.990657	0.89021	0.9142478
P68402	Platelet-activating factor acetylhydrolase IB subunit beta OS=Homo sapiens GN=PAFAH1B2 PE=1 SV=1 - [PA1B2_HUMAN]	14.85	3	3	5	229	25.55312	5.922363	0.942682	0.960996	1.06721	0.9902959	0.825933	0.8615665
B9EG60	Synaptopodin 2 OS=Homo sapiens GN=SYNPO2 PE=2 SV=1 - [B9EG60_HUMAN]	23.71	4	22	37	1261	136.2365	8.79248	1.02223	0.992976	0.955498	0.9902364	0.663396	0.7188206
A6NJA2	Ubiquitin carboxyl-terminal hydrolase 14 OS=Homo sapiens GN=USP14 PE=1 SV=2 - [A6NJA2_HUMAN]	22.32	7	7	12	448	51.0537	5.922363	1.00614	1.00117	0.962221	0.9898445	0.540638	0.6077913
V9GYG9	Apolipoprotein A-II (Fragment) OS=Homo sapiens GN=APOA2 PE=1 SV=1 - [V9GYG9_HUMAN]	42.11	5	5	16	95	10.64264	6.624512	0.990533	0.963405	1.01555	0.9898279	0.568931	0.6318345
B4DQZ7	cDNA FLJ50838, highly similar to Apoptotic chromatin condensation inducer in thenucleus (Fragment) OS=Homo sapiens PE=2 SV=1 - [B4DQZ7_HUMAN]	2.55	2	2	2	1096	122.4847	5.655762	1.0086	0.990228	0.969677	0.9895024	0.449003	0.5196154

Biology Open • Supplementary information

B7Z7R8	cDNA FLJ55622, highly similar to Multimerin-1 OS=Homo sapiens PE=2 SV=1 - [B7Z7R8_HUMAN]	4.02	3	3	4	970	110.6946	7.02002	0.987747	0.986382	0.993982	0.9893706	0.0451788	0.0965469
P05160	Coagulation factor XIII B chain OS=Homo sapiens GN=F13B PE=1 SV=3 - [F13B_HUMAN]	3.18	2	2	2	661	75.46117	6.39209	0.931049	1.09877	0.937765	0.9891953	0.861975	0.890411
P06753	Tropomyosin alpha-3 chain OS=Homo sapiens GN=TPM3 PE=1 SV=2 - [TPM3_HUMAN]	52.63	3	21	61	285	32.92978	4.716309	1.09322	0.90125	0.973104	0.9891917	0.864773	0.8927285
H0Y984	ADP-ribosyl cyclase/cyclic ADP-ribose hydrolase 2 (Fragment) OS=Homo sapiens GN=BST1 PE=1 SV=1 - [H0Y984_HUMAN]	12.9	2	2	3	186	21.06801	5.57959	0.968596	1.03118	0.967255	0.9890104	0.65424	0.7104916
B1AKG0	Complement factor H-related protein 1 OS=Homo sapiens GN=CFHR1 PE=1 SV=1 - [B1AKG0_HUMAN]	22.88	3	6	9	271	30.83791	7.811035	0.984461	1.00609	0.976309	0.9889537	0.339772	0.4132692
P45974	Ubiquitin carboxyl-terminal hydrolase 5 OS=Homo sapiens GN=USP5 PE=1 SV=2 - [UBP5_HUMAN]	16.32	9	10	12	858	95.72534	5.033691	1.00443	0.966606	0.995696	0.9889102	0.434386	0.5063349
Q96HE7	ERO1-like protein alpha OS=Homo sapiens GN=ERO1A PE=1 SV=2 - [ERO1A_HUMAN]	6.2	2	2	2	468	54.35805	5.681152	1.01141	0.893097	1.06214	0.9888822	0.844927	0.8784023
H0YMU3	Isocitrate dehydrogenase [NAD] subunit alpha, mitochondrial (Fragment) OS=Homo sapiens GN=IDH3A PE=1 SV=1 - [H0YMU3_HUMAN]	32	4	4	4	175	19.23498	9.466309	0.977014	1.02384	0.965677	0.9888422	0.594778	0.6560404
Q5SSJ5	Heterochromatin protein 1-binding protein 3 OS=Homo sapiens GN=HP1BP3 PE=1 SV=1 - [HP1B3_HUMAN]	20.25	11	11	18	553	61.16927	9.671387	0.961157	1.05293	0.951762	0.9886153	0.757953	0.8030182
A0A024RBH7	Thymopoietin, isoform CRA_a OS=Homo sapiens GN=TMPO PE=4 SV=1 - [A0A024RBH7_HUMAN]	33.33	8	8	12	345	38.71426	9.158691	1.00439	0.924858	1.03629	0.9885115	0.761862	0.8056554
O95479	GDH/6PGL endoplasmic bifunctional protein OS=Homo sapiens GN=H6PD PE=1 SV=2 - [G6PE_HUMAN]	6.07	4	4	4	791	88.83641	7.29834	1.0097	0.968837	0.985198	0.9879117	0.415757	0.4883197
Q15746	Myosin light chain kinase, smooth muscle OS=Homo sapiens GN=MYLK PE=1 SV=4 - [MYLK_HUMAN]	16.51	25	26	47	1914	210.5828	6.150879	0.991459	0.999817	0.972153	0.9878096	0.275123	0.3473672

Biology Open • Supplementary information

A8K079	cDNA FLJ75240, highly similar to Homo sapiens methyl CpG binding protein 2 (MECP2) mRNA OS=Homo sapiens PE=2 SV=1 - [A8K079_HUMAN]	16.87	6	6	9	486	52.4667	9.949707	1.02797	0.985355	0.949944	0.9877569	0.641685	0.6997757
A0A087WUA5	EH domain-containing protein 4 OS=Homo sapiens GN=EHD4 PE=1 SV=1 - [A0A087WUA5_HUMAN]	28.89	10	16	33	540	61.0747	8.265137	0.997805	1.02587	0.939375	0.9876821	0.676489	0.7303008
E9PRU1	EGF-containing fibulin-like extracellular matrix protein 2 OS=Homo sapiens GN=EFEMP2 PE=1 SV=1 - [E9PRU1_HUMAN]	5.28	2	2	2	436	48.15965	5.008301	1.06734	0.869433	1.02568	0.9874849	0.854653	0.8857931
B4DV79	Eukaryotic translation initiation factor 3 subunit B OS=Homo sapiens GN=EIF3B PE=2 SV=1 - [B4DV79_HUMAN]	5.42	3	3	4	738	85.08138	5.262207	0.985244	1.01886	0.957697	0.987266	0.546264	0.6124888
P46939	Utrophin OS=Homo sapiens GN=UTRN PE=1 SV=2 - [UTRO_HUMAN]	5.71	17	18	23	3433	394.2203	5.325684	0.975338	1.06248	0.923933	0.9872499	0.782382	0.823496
Q59H91	Inositol 1,4,5-trisphosphate receptor type 1 variant (Fragment) OS=Homo sapiens PE=2 SV=1 - [Q59H91_HUMAN]	4.14	3	4	4	1207	136.3444	5.744629	1.03681	0.97832	0.945842	0.9869914	0.673362	0.7277556
C9J0J7	Profilin-2 OS=Homo sapiens GN=PFN2 PE=1 SV=1 - [C9J0J7_HUMAN]	24.18	2	2	2	91	9.834085	9.17334	0.919188	1.06135	0.980117	0.9868849	0.780291	0.8218062
P00390	Glutathione reductase, mitochondrial OS=Homo sapiens GN=GSR PE=1 SV=2 - [GSHR_HUMAN]	15.9	5	5	5	522	56.22095	8.499512	0.962594	0.974563	1.02274	0.9866324	0.54269	0.6094909
A8K9K1	Calcium-transporting ATPase OS=Homo sapiens PE=2 SV=1 - [A8K9K1_HUMAN]	8.92	3	6	10	998	109.1101	5.617676	0.990469	1.01035	0.95877	0.9865309	0.4645	0.533899
B2RAK1	cDNA, FLJ94965, highly similar to Homo sapiens leucyl/cystinyl aminopeptidase (LNPEP), mRNA OS=Homo sapiens PE=2 SV=1 - [B2RAK1_HUMAN]	4.59	4	4	4	1025	117.2703	5.731934	0.979173	1.03511	0.944281	0.9861879	0.65365	0.7103306
Q8TCF0	LBP protein OS=Homo sapiens GN=LBP PE=2 SV=1 - [Q8TCF0_HUMAN]	8.18	4	4	7	477	52.90066	6.756348	0.990205	1.02681	0.94129	0.9861013	0.631233	0.6903269

Biology Open • Supplementary information

D3DWB6	Ubiquitin specific peptidase 9, X-linked, isoform CRA_b OS=Homo sapiens GN=USP9X PE=4 SV=1 - [D3DWB6_HUMAN]	2.31	4	4	4	2379	271.0811	5.973145	1.00826	0.955119	0.99334	0.9855731	0.458179	0.5280681
P10644	cAMP-dependent protein kinase type I-alpha regulatory subunit OS=Homo sapiens GN=PRKAR1A PE=1 SV=1 - [KAP0_HUMAN]	29.66	9	10	13	381	42.95497	5.351074	1.00808	1.00542	0.942812	0.9854361	0.565146	0.6294808
B7Z899	cDNA FLJ55506, highly similar to Puromycin-sensitive aminopeptidase (EC3.4.11.-) OS=Homo sapiens PE=2 SV=1 - [B7Z899_HUMAN]	17.6	14	14	17	915	102.8662	5.655762	0.959154	1.00407	0.992709	0.9853094	0.389676	0.4620152
K7ERI9	Apolipoprotein C-I (Fragment) OS=Homo sapiens GN=APOC1 PE=1 SV=1 - [K7ERI9_HUMAN]	37.66	5	5	14	77	8.64167	6.712402	1.00873	0.977481	0.967987	0.9847332	0.340601	0.4141287
B4DTY8	cDNA FLJ61587, highly similar to Integrin alpha-1 (Fragment) OS=Homo sapiens PE=2 SV=1 - [B4DTY8_HUMAN]	11	12	12	17	1173	129.9202	6.17627	0.931333	0.992068	1.03074	0.9847144	0.65003	0.7070538
B4DTU7	10-formyltetrahydrofolate dehydrogenase OS=Homo sapiens PE=2 SV=1 - [B4DTU7_HUMAN]	7.37	5	5	6	923	101.6512	6.521973	0.939784	0.996842	1.0165	0.9843752	0.567097	0.6303635
Q53GF0	Cytidine 5'-monophosphate N-acetylneuraminic acid synthetase variant (Fragment) OS=Homo sapiens PE=2 SV=1 - [Q53GF0_HUMAN]	9.22	2	2	2	434	48.29862	7.928223	0.985088	0.998632	0.969331	0.9843502	0.205789	0.2750082
Q93034	Cullin-5 OS=Homo sapiens GN=CUL5 PE=1 SV=4 - [CUL5_HUMAN]	4.36	3	3	3	780	90.89741	7.942871	0.994332	1.03571	0.922466	0.9841708	0.67953	0.7330175
C9JEV6	N-acetyl-D-glucosamine kinase OS=Homo sapiens GN=NAGK PE=1 SV=3 - [C9JEV6_HUMAN]	24.91	7	7	9	293	31.99727	6.990723	0.977193	0.902085	1.07169	0.9836567	0.770752	0.81328
Q9UBG0	C-type mannose receptor 2 OS=Homo sapiens GN=MRC2 PE=1 SV=2 - [MRC2_HUMAN]	5.81	6	6	7	1479	166.568	5.833496	0.997633	0.95758	0.995232	0.9834814	0.33078	0.4046586
H9KV28	Protein diaphanous homolog 1 OS=Homo sapiens GN=DIAPH1 PE=1 SV=2 - [H9KV28_HUMAN]	2.69	3	3	3	1228	136.7651	5.236816	0.903604	1.03078	1.01563	0.9833386	0.718154	0.7683158

B3KM86	cDNA FLJ10524 fis, clone NT2RP2000880, highly similar to Eukaryotic translation initiation factor 5B OS=Homo sapiens PE=2 SV=1 - [B3KM86_HUMAN]	8.2	2	2	2	634	71.44772	6.844238	1.06106	0.965026	0.923835	0.9833072	0.721177	0.7707906
E9PN51	NADH dehydrogenase [ubiquinone] iron-sulfur protein 8, mitochondrial (Fragment) OS=Homo sapiens GN=NDUFS8 PE=1 SV=1 - [E9PN51_HUMAN]	17.27	2	2	2	110	12.39731	9.979004	0.949356	0.96857	1.03153	0.9831521	0.567234	0.6303635
P53004	Biliverdin reductase A OS=Homo sapiens GN=BLVRA PE=1 SV=2 - [BIEA_HUMAN]	22.64	6	6	10	296	33.40733	6.442871	1.01731	0.98355	0.94792	0.9829279	0.483896	0.552814
P62195	26S protease regulatory subunit 8 OS=Homo sapiens GN=PSMC5 PE=1 SV=1 - [PRS8_HUMAN]	13.05	4	4	4	406	45.59706	7.547363	0.952577	0.972833	1.0229	0.9827688	0.496332	0.564924
H0Y9Y1	Transcription factor BTF3 (Fragment) OS=Homo sapiens GN=BTF3 PE=1 SV=2 - [H0Y9Y1_HUMAN]	32.56	2	2	3	86	9.493752	4.995605	1.04361	0.952353	0.951897	0.9826198	0.626213	0.6860004
B7Z3Z9	Long-chain-fatty-acid--CoA ligase 1 OS=Homo sapiens GN=ACSL1 PE=1 SV=1 - [B7Z3Z9_HUMAN]	10.44	4	5	7	527	58.54831	7.532715	0.9706	0.962177	1.01393	0.9822359	0.383266	0.4563332
Q92522	Histone H1x OS=Homo sapiens GN=H1FX PE=1 SV=1 - [H1X_HUMAN]	11.27	3	3	4	213	22.47353	10.75537	0.99338	0.983014	0.96861	0.9816679	0.125291	0.1904401
D6RE83	Ubiquitin carboxyl-terminal hydrolase OS=Homo sapiens GN=UCHL1 PE=1 SV=1 - [D6RE83_HUMAN]	22.22	4	4	6	207	23.15968	5.478027	0.978928	1.02832	0.937618	0.9816228	0.555927	0.6206507
P38117	Electron transfer flavoprotein subunit beta OS=Homo sapiens GN=ETFB PE=1 SV=3 - [ETFB_HUMAN]	25.88	5	5	7	255	27.82615	8.104004	0.967338	0.990407	0.986722	0.9814888	0.12254	0.1876065
E7EU96	Casein kinase II subunit alpha OS=Homo sapiens GN=CSNK2A1 PE=1 SV=1 - [E7EU96_HUMAN]	15.06	4	5	8	385	45.28189	7.942871	0.950585	0.99794	0.995925	0.9814833	0.353722	0.427472
F5GZZ9	Scavenger receptor cysteine-rich type 1 protein M130 OS=Homo sapiens GN=CD163 PE=1 SV=1 - [F5GZZ9_HUMAN]	6.67	6	6	6	1109	120.2436	6.100098	1.00409	0.985058	0.954932	0.9813597	0.322525	0.395991

Biology Open • Supplementary information

B4E0X8	cDNA FLJ61021, highly similar to Far upstream element-binding protein 1 OS=Homo sapiens PE=2 SV=1 - [B4E0X8_HUMAN]	14.47	5	8	8	629	66.19083	7.562012	0.964214	0.939943	1.03904	0.9810646	0.590403	0.6524695
G3V3I1	Proteasome subunit alpha type OS=Homo sapiens GN=PSMA6 PE=1 SV=1 - [G3V3I1_HUMAN]	42.57	5	5	7	148	16.63421	8.631348	0.944212	1.00939	0.989274	0.9809587	0.427231	0.499543
B3KY54	cDNA FLJ46886 fis, clone UTERU3016308, highly similar to Smoothelin OS=Homo sapiens PE=2 SV=1 - [B3KY54_HUMAN]	19.86	8	8	8	584	61.57261	7.752441	0.945229	0.971599	1.02601	0.9809469	0.507127	0.575374
H7BXI1	Extended synaptotagmin-2 (Fragment) OS=Homo sapiens GN=ESYT2 PE=1 SV=1 - [H7BXI1_HUMAN]	7.69	5	5	7	884	97.94866	8.572754	1.10886	0.884551	0.949357	0.9809228	0.801623	0.8403606
P62258	14-3-3 protein epsilon OS=Homo sapiens GN=YWHAE PE=1 SV=1 - [1433E_HUMAN]	56.86	9	12	40	255	29.15542	4.741699	0.983076	0.993975	0.96571	0.98092	0.146306	0.2121876
D3DT44	Glutamate-cysteine ligase, modifier subunit, isoform CRA_a OS=Homo sapiens GN=GCLM PE=4 SV=1 - [D3DT44_HUMAN]	11.42	2	2	2	254	28.59775	6.023926	0.972211	1.00471	0.965586	0.9808349	0.253759	0.324386
Q16527	Cysteine and glycine-rich protein 2 OS=Homo sapiens GN=CSRP2 PE=1 SV=3 - [CSRP2_HUMAN]	47.15	7	7	14	193	20.93988	8.616699	0.977251	0.997974	0.966822	0.9806822	0.169319	0.2370326
A8MWD9	Putative small nuclear ribonucleoprotein G-like protein 15 OS=Homo sapiens GN=SNRPGP15 PE=5 SV=2 - [RUXGL_HUMAN]	34.21	3	3	4	76	8.538445	8.836426	0.949245	1.01075	0.981862	0.9806199	0.389248	0.4619932
B4DEQ0	cDNA FLJ59482, highly similar to Electron transfer flavoprotein-ubiquinone oxidoreductase, mitochondrial (EC 1.5.5.1) OS=Homo sapiens PE=2 SV=1 - [B4DEQ0_HUMAN]	4.14	2	2	2	556	61.34613	6.800293	0.920576	1.01193	1.00918	0.9805604	0.583477	0.6458525
B4DMF5	Glutamate dehydrogenase OS=Homo sapiens PE=2 SV=1 - [B4DMF5_HUMAN]	27.7	12	12	23	509	56.56758	7.210449	0.977288	0.994015	0.970056	0.980453	0.110366	0.1733192

Biology Open • Supplementary information

Q13838	Spliceosome RNA helicase DDX39B OS=Homo sapiens GN=DDX39B PE=1 SV=1 - [DX39B_HUMAN]	23.13	4	9	14	428	48.95994	5.668457	0.92816	1.13287	0.879906	0.9803127	0.823294	0.8590784
Q71UH7	Galactokinase (Fragment) OS=Homo sapiens GN=GALK1 PE=3 SV=1 - [Q71UH7_HUMAN]	12.12	2	2	3	264	28.47862	5.300293	1.00542	0.996966	0.938088	0.9801578	0.447671	0.5184284
O95571	Persulfide dioxygenase ETHE1, mitochondrial OS=Homo sapiens GN=ETHE1 PE=1 SV=2 - [ETHE1_HUMAN]	16.14	3	3	4	254	27.85511	6.82959	1.01421	0.895408	1.02905	0.9795576	0.67658	0.7303008
B2R960	cDNA, FLJ94230, highly similar to Homo sapiens thioredoxin-like 1 (TXNL1), mRNA OS=Homo sapiens PE=2 SV=1 - [B2R960_HUMAN]	34.26	6	6	6	289	32.21671	4.95752	0.949838	0.989332	0.999244	0.9794712	0.306742	0.3793649
E9PCY5	DNA topoisomerase 2 (Fragment) OS=Homo sapiens GN=TOP2B PE=1 SV=1 - [E9PCY5_HUMAN]	4.09	4	4	4	1150	130.3978	8.470215	0.999086	0.900693	1.0375	0.9790927	0.658872	0.714376
A0A0A0MSV6	Complement C1q subcomponent subunit B (Fragment) OS=Homo sapiens GN=C1QB PE=1 SV=4 - [A0A0A0MSV6_HUMAN]	12.72	2	2	3	228	24.01526	9.158691	0.973692	1.01971	0.943129	0.9788447	0.442189	0.5133093
Q99536	Synaptic vesicle membrane protein VAT-1 homolog OS=Homo sapiens GN=VAT1 PE=1 SV=2 - [VAT1_HUMAN]	31.04	10	10	24	393	41.89342	6.290527	1.00933	0.969494	0.957539	0.9787887	0.308237	0.3809355
B2R8W4	cDNA, FLJ94092 OS=Homo sapiens PE=2 SV=1 - [B2R8W4_HUMAN]	7.71	2	2	4	402	46.90468	6.55127	1.11282	0.821913	0.999439	0.9780583	0.81973	0.8561512
P31150	Rab GDP dissociation inhibitor alpha OS=Homo sapiens GN=GDI1 PE=1 SV=2 - [GDIA_HUMAN]	33.56	8	12	19	447	50.55014	5.135254	1.03178	1.00088	0.901437	0.9780336	0.632626	0.6912327
P00568	Adenylate kinase isoenzyme 1 OS=Homo sapiens GN=AK1 PE=1 SV=3 - [KAD1_HUMAN]	42.27	8	8	11	194	21.62131	8.631348	1.0064	1.01506	0.912427	0.9779628	0.571554	0.6343312
Q96PK6	RNA-binding protein 14 OS=Homo sapiens GN=RBM14 PE=1 SV=2 - [RBM14_HUMAN]	5.68	3	3	3	669	69.44891	9.671387	0.989028	0.931844	1.01261	0.9778275	0.452745	0.5228734
A0A024R1S5	GTP binding protein 1, isoform CRA_c OS=Homo sapiens GN=GTPBP1 PE=4 SV=1 - [A0A024R1S5_HUMAN]	4.55	2	2	2	660	71.41855	8.221191	0.895044	0.982458	1.05566	0.9777198	0.678648	0.7322995

Biology Open • Supplementary information

Q92905	COP9 signalosome complex subunit 5 OS=Homo sapiens GN=COPS5 PE=1 SV=4 - [CSN5_HUMAN]	10.48	3	3	4	334	37.55472	6.536621	0.920843	1.03701	0.975095	0.9776507	0.573972	0.6363889
Q02952	A-kinase anchor protein 12 OS=Homo sapiens GN=AKAP12 PE=1 SV=4 - [AKA12_HUMAN]	17.12	18	18	25	1782	191.3667	4.411621	0.955859	1.00236	0.974543	0.977587	0.238969	0.3079216
A8K4W7	cDNA FLJ76284, highly similar to Homo sapiens succinate-CoA ligase, GDP-forming, alpha subunit (SUCLG1), mRNA OS=Homo sapiens PE=2 SV=1 - [A8K4W7_HUMAN]	15.32	4	4	4	333	35.02623	8.79248	0.983966	0.9379	1.01079	0.9775505	0.40213	0.47462
P20591	Interferon-induced GTP-binding protein Mx1 OS=Homo sapiens GN=MX1 PE=1 SV=4 - [MX1_HUMAN]	12.39	4	6	6	662	75.47322	5.833496	0.978861	1.04525	0.908511	0.9775422	0.626824	0.6860004
P16435	NADPH--cytochrome P450 reductase OS=Homo sapiens GN=POR PE=1 SV=2 - [NCPR_HUMAN]	4.43	3	3	3	677	76.64125	5.57959	1.00017	0.968667	0.963286	0.977373	0.188031	0.2571028
B4DDB9	cDNA FLJ56339, highly similar to Signal peptidase complex subunit 2 (EC 3.4.-.-) OS=Homo sapiens PE=2 SV=1 - [B4DDB9_HUMAN]	16.81	3	3	4	226	25.02073	8.470215	0.980895	0.961641	0.989072	0.9772027	0.107151	0.1700451
P25189	Myelin protein P0 OS=Homo sapiens GN=MPZ PE=1 SV=1 - [MYP0_HUMAN]	8.06	2	2	2	248	27.53748	9.539551	0.993165	0.961097	0.976841	0.9770341	0.131256	0.1962687
B7Z7P8	Eukaryotic peptide chain release factor subunit 1 OS=Homo sapiens GN=ETF1 PE=1 SV=1 - [B7Z7P8_HUMAN]	16.55	5	5	7	423	47.44619	5.566895	0.933865	0.95983	1.03698	0.976893	0.533339	0.6003833
E9PNW4	CD59 glycoprotein OS=Homo sapiens GN=CD59 PE=1 SV=1 - [E9PNW4_HUMAN]	29.63	4	4	8	108	11.97652	5.744629	0.944031	1.0491	0.937	0.976711	0.586417	0.6484879
P09327	Villin-1 OS=Homo sapiens GN=VIL1 PE=1 SV=4 - [VILI_HUMAN]	4.96	3	3	3	827	92.63675	6.39209	0.978706	0.866468	1.08461	0.9765933	0.745831	0.7924162
B3KSG9	cDNA FLJ36188 fis, clone TESTI2027179, highly similar to Transmembrane 9 superfamily protein member 2 OS=Homo sapiens PE=2 SV=1 - [B3KSG9_HUMAN]	5.06	2	2	2	573	65.38712	7.283691	0.986975	0.962196	0.979709	0.9762935	0.0842509	0.1443266

Biology Open • Supplementary information

M0R0P1	rRNA 2'-O-methyltransferase fibrillarin (Fragment) OS=Homo sapiens GN=FBL PE=1 SV=1 - [M0R0P1_HUMAN]	24.12	4	4	5	228	24.52885	10.11084	0.981623	1.03022	0.915266	0.9757012	0.541631	0.6087055
B5BUB1	RuvB-like 1 (Fragment) OS=Homo sapiens GN=RUVBL1 PE=2 SV=1 - [B5BUB1_HUMAN]	26.1	9	9	16	456	50.18428	6.41748	1.01099	0.993409	0.922366	0.975587	0.462602	0.5322596
Q5VU21	PAI-1 mRNA-binding protein variant OS=Homo sapiens PE=2 SV=1 - [Q5VU21_HUMAN]	13.44	4	4	4	387	42.40128	8.440918	1.01423	0.956203	0.955121	0.9751853	0.331595	0.4052163
P52272	Heterogeneous nuclear ribonucleoprotein M OS=Homo sapiens GN=HNRNPM PE=1 SV=3 - [HNRPM_HUMAN]	18.49	11	11	18	730	77.46432	8.70459	0.993458	0.906672	1.02539	0.9751736	0.556426	0.621003
B7Z953	cDNA FLJ53758 OS=Homo sapiens PE=2 SV=1 - [B7Z953_HUMAN]	10.36	2	2	2	338	38.12943	8.484863	1.04843	0.968044	0.907711	0.9747283	0.598473	0.6592356
P22676	Calretinin OS=Homo sapiens GN=CALB2 PE=2 SV=2 - [CALB2_HUMAN]	9.23	2	2	3	271	31.51958	5.147949	0.960058	0.932708	1.03064	0.9744675	0.473758	0.543065
P61088	Ubiquitin-conjugating enzyme E2 N OS=Homo sapiens GN=UBE2N PE=1 SV=1 - [UBE2N_HUMAN]	36.84	4	4	12	152	17.12698	6.565918	0.957528	1.05619	0.909594	0.9744377	0.613674	0.6742248
B4DNT5	cDNA FLJ60316, highly similar to Apolipoprotein-L1 OS=Homo sapiens PE=2 SV=1 - [B4DNT5_HUMAN]	8.3	2	2	3	277	30.79135	8.660645	0.991705	0.978693	0.95246	0.974286	0.15572	0.2231635
B2R858	cDNA, FLJ93750, Homo sapiens DEAD (Asp-Glu-Ala-Asp) box polypeptide 6 (DDX6), mRNA OS=Homo sapiens PE=2 SV=1 - [B2R858_HUMAN]	19.7	7	8	9	472	53.18271	8.660645	0.955809	1.04394	0.922284	0.9740115	0.548136	0.6137745
B3KXZ9	cDNA FLJ46477 fis, clone THYMU3025118, highly similar to Cell surface glycoprotein MUC18 OS=Homo sapiens PE=2 SV=1 - [B3KXZ9_HUMAN]	18.08	7	7	13	531	58.98268	5.186035	0.987411	0.981992	0.950491	0.9732978	0.146149	0.212132
P56385	ATP synthase subunit e, mitochondrial OS=Homo sapiens GN=ATP5I PE=1 SV=2 - [ATP5I_HUMAN]	30.43	2	2	3	69	7.928326	9.349121	1.01662	1.00918	0.893094	0.9729671	0.568776	0.6318345
Q05707	Collagen alpha-1(XIV) chain OS=Homo sapiens GN=COL14A1 PE=1 SV=3 - [COEA1_HUMAN]	33.91	51	51	169	1796	193.3945	5.300293	0.958022	0.988288	0.971469	0.9725933	0.0886843	0.1494818

Biology Open • Supplementary information

Q06124	Tyrosine-protein phosphatase non-receptor type 11 OS=Homo sapiens GN=PTPN11 PE=1 SV=2 - [PTN11_HUMAN]	3.52	2	2	2	597	68.39333	7.29834	0.959672	0.962625	0.995405	0.9725674	0.138844	0.2039874
P02545	Prelamin-A/C OS=Homo sapiens GN=LMNA PE=1 SV=1 - [LMNA_HUMAN]	56.17	37	37	110	664	74.09471	7.02002	0.972981	0.975002	0.969303	0.9724288	0.00364146	0.0381621
A8K5J8	cDNA FLJ75066, highly similar to Homo sapiens complement component 1, r subcomponent (C1R), mRNA OS=Homo sapiens PE=2 SV=1 - [A8K5J8_HUMAN]	17.02	7	7	8	705	80.14698	6.442871	0.9644	0.909674	1.043	0.9723567	0.549081	0.6146294
Q53EL3	Tyrosine-protein kinase (Fragment) OS=Homo sapiens PE=2 SV=1 - [Q53EL3_HUMAN]	9.13	4	4	4	449	50.5847	7.063965	0.984529	0.952393	0.979788	0.9722368	0.109233	0.1722459
F5H5V4	26S proteasome non-ATPase regulatory subunit 9 (Fragment) OS=Homo sapiens GN=PSMD9 PE=1 SV=1 - [F5H5V4_HUMAN]	30.72	4	4	6	153	16.9024	6.112793	0.961027	0.964383	0.990367	0.9719257	0.093948	0.1551288
Q15459	Splicing factor 3A subunit 1 OS=Homo sapiens GN=SF3A1 PE=1 SV=1 - [SF3A1_HUMAN]	4.67	3	3	3	793	88.83052	5.224121	1.06003	0.966611	0.888916	0.9718537	0.626741	0.6860004
A0A087WTP3	Far upstream element-binding protein 2 OS=Homo sapiens GN=KHSRP PE=1 SV=1 - [A0A087WTP3_HUMAN]	14.06	5	7	8	711	72.98228	7.708496	0.986536	0.912754	1.01607	0.9717879	0.455428	0.5254341
B7Z2K7	cDNA FLJ53289, highly similar to Ribosomal protein S6 kinase alpha-1 (EC 2.7.11.1) OS=Homo sapiens PE=2 SV=1 - [B7Z2K7_HUMAN]	4.55	3	3	4	594	67.33675	7.635254	0.946409	1.00114	0.967455	0.9716684	0.21747	0.2871045
B4DDM6	cDNA FLJ57899, highly similar to Mitotic checkpoint protein BUB3 OS=Homo sapiens PE=2 SV=1 - [B4DDM6_HUMAN]	8.87	2	2	2	248	28.22088	7.10791	1.01877	0.966528	0.928834	0.9713759	0.386803	0.4595746
P14324	Farnesyl pyrophosphate synthase OS=Homo sapiens GN=FDPS PE=1 SV=4 - [FPPS_HUMAN]	9.79	3	3	5	419	48.24463	6.150879	1.04935	0.863192	1.00127	0.9712674	0.657824	0.7136969
P12259	Coagulation factor V OS=Homo sapiens GN=F5 PE=1 SV=4 - [FA5_HUMAN]	1.98	3	3	3	2224	251.5455	6.049316	1.00982	0.970978	0.93177	0.9708577	0.325133	0.3984704

Biology Open • Supplementary information

Q9H2D6	TRIO and F-actin-binding protein OS=Homo sapiens GN=TRIOBP PE=1 SV=3 - [TARA_HUMAN]	2.16	4	4	4	2365	261.2169	8.484863	0.989434	1.03132	0.891508	0.9707531	0.553344	0.6183788
B2RDD7	Protein arginine N-methyltransferase 5 OS=Homo sapiens PE=2 SV=1 - [B2RDD7_HUMAN]	9.11	4	4	4	637	72.6636	6.290527	0.918969	1.01908	0.974129	0.9707261	0.418366	0.4905331
A6PVJ3	ERGIC and golgi 3 (Fragment) OS=Homo sapiens GN=ERGIC3 PE=4 SV=1 - [A6PVJ3_HUMAN]	11.38	3	3	3	325	36.82816	5.338379	0.999473	0.975663	0.936379	0.9705047	0.250031	0.3202251
Q9BXP5	Serrate RNA effector molecule homolog OS=Homo sapiens GN=SRRT PE=1 SV=1 - [SRRT_HUMAN]	4	3	3	3	876	100.6043	5.960449	0.982655	1.01145	0.91689	0.9703329	0.400196	0.4731622
E9PJK1	Tetraspanin OS=Homo sapiens GN=CD81 PE=1 SV=1 - [E9PJK1_HUMAN]	40.61	4	4	7	165	17.95093	4.995605	0.973449	1.00976	0.927771	0.970326	0.337423	0.411447
B7Z7C0	cDNA FLJ53109, highly similar to Serine/threonine-protein phosphatase 2A 56 kDa regulatory subunit epsilon isoform OS=Homo sapiens PE=2 SV=1 - [B7Z7C0_HUMAN]	5.88	2	2	3	391	46.08996	5.922363	1.04156	0.948679	0.920168	0.9701345	0.500712	0.5691438
Q6FG43	FLOT2 protein OS=Homo sapiens GN=FLOT2 PE=2 SV=1 - [Q6FG43_HUMAN]	19.26	6	6	6	379	41.65924	5.287598	0.895659	0.931449	1.0801	0.9690696	0.638829	0.6973351
Q9NWV4	UPF0587 protein C1orf123 OS=Homo sapiens GN=C1orf123 PE=1 SV=1 - [CA123_HUMAN]	30	3	3	4	160	18.03681	5.008301	0.996875	1.02209	0.888171	0.9690446	0.529816	0.597013
P01031	Complement C5 OS=Homo sapiens GN=C5 PE=1 SV=4 - [CO5_HUMAN]	9.19	14	14	15	1676	188.1861	6.521973	0.944394	0.975593	0.987121	0.9690358	0.136058	0.2010994
B3KS64	cDNA FLJ35580 fis, clone SPLEN2006389, highly similar to FIBROMODULIN OS=Homo sapiens PE=2 SV=1 - [B3KS64_HUMAN]	8.71	2	2	2	287	32.09673	9.187988	0.886726	0.975735	1.04419	0.9688842	0.565324	0.6294808
P98095	Fibulin-2 OS=Homo sapiens GN=FBLN2 PE=1 SV=2 - [FBLN2_HUMAN]	24.49	22	22	40	1184	126.4891	4.817871	0.964776	0.97537	0.966306	0.9688171	0.0110533	0.0493307
A0A024RDM2	Crystallin, lambda 1, isoform CRA_a OS=Homo sapiens GN=CRYL1 PE=4 SV=1 - [A0A024RDM2_HUMAN]	19.19	5	5	6	297	33.33702	5.998535	0.928728	0.947489	1.03015	0.9687882	0.421932	0.4944162

Biology Open • Supplementary information

A0A087X142	Septin-8 OS=Homo sapiens GN=SEPT8 PE=1 SV=1 - [A0A087X142_HUMAN]	14.79	2	5	15	426	49.32507	6.20166	0.912293	1.07937	0.912766	0.9681422	0.624565	0.6848567
A0A024R2W4	Dystroglycan 1 (Dystrophin-associated glycoprotein 1), isoform CRA_a OS=Homo sapiens GN=DAG1 PE=4 SV=1 - [A0A024R2W4_HUMAN]	4.36	3	3	4	895	97.47971	8.558105	0.93813	0.94317	1.02293	0.9680772	0.365065	0.4385185
B4DWV9	cDNA FLJ53108, highly similar to Guanine nucleotide-binding protein alpha-13 subunit OS=Homo sapiens PE=2 SV=1 - [B4DWV9_HUMAN]	17.33	4	5	10	352	40.94913	7.986816	0.940197	0.969221	0.993985	0.967801	0.174105	0.2417332
Q9HBR7	Propionyl Coenzyme A carboxylase, beta polypeptide, isoform CRA_a OS=Homo sapiens GN=PCCB PE=2 SV=1 - [Q9HBR7_HUMAN]	12.25	3	3	4	302	32.85632	4.76709	0.975347	0.93832	0.987923	0.9671966	0.158431	0.2257108
G8JLD5	Dynamin-1-like protein OS=Homo sapiens GN=DNM1L PE=1 SV=1 - [G8JLD5_HUMAN]	9.13	5	5	6	712	79.57199	7.078613	0.939164	0.993598	0.968382	0.9670482	0.171153	0.2388111
A8K968	Band 4.1-like protein 3 OS=Homo sapiens GN=EPB41L3 PE=1 SV=1 - [A8K968_HUMAN]	20.24	9	13	17	756	84.65941	5.439941	0.939723	0.929008	1.03083	0.9665195	0.408866	0.4808935
A8K6A5	cDNA FLJ77742, highly similar to Homo sapiens integrin, alpha 5 (fibronectin receptor, alpha polypeptide), mRNA OS=Homo sapiens PE=2 SV=1 - [A8K6A5_HUMAN]	3.05	2	2	2	1049	114.4117	5.706543	0.948187	0.964332	0.98669	0.9664029	0.0949366	0.1563678
G3XAM2	Complement factor I OS=Homo sapiens GN=CFI PE=1 SV=1 - [G3XAM2_HUMAN]	17.36	3	8	11	576	65.01628	7.503418	0.934927	0.920341	1.04346	0.9662437	0.476406	0.5459155
P05556	Integrin beta-1 OS=Homo sapiens GN=ITGB1 PE=1 SV=2 - [ITB1_HUMAN]	25.31	17	17	34	798	88.35701	5.38916	0.945303	0.965303	0.988093	0.9662329	0.111949	0.1750334
P08237	ATP-dependent 6-phosphofructokinase, muscle type OS=Homo sapiens GN=PFKM PE=1 SV=2 - [PFKAM_HUMAN]	11.03	5	7	9	780	85.12839	7.986816	0.968694	1.01554	0.914244	0.9661599	0.367068	0.4404556

Biology Open • Supplementary information

B4DUG5	cDNA FLJ57006, highly similar to Cadherin-17 OS=Homo sapiens PE=2 SV=1 - [B4DUG5_HUMAN]	13.32	4	4	6	443	48.3118	5.338379	0.95915	0.981766	0.956118	0.9656779	0.0513376	0.1038679
Q9NXF1	Testis-expressed sequence 10 protein OS=Homo sapiens GN=TEX10 PE=1 SV=2 - [TEX10_HUMAN]	3.34	2	2	2	929	105.6076	9.36377	1.0059	0.822849	1.06805	0.9656011	0.686193	0.7383227
O00410	Importin-5 OS=Homo sapiens GN=IPO5 PE=1 SV=4 - [IPO5_HUMAN]	7.29	6	6	6	1097	123.5499	4.944824	0.990299	0.951738	0.953552	0.965196	0.109328	0.1722459
K7EJE8	Lon protease homolog OS=Homo sapiens GN=LONP1 PE=1 SV=1 - [K7EJE8_HUMAN]	6.76	6	6	6	829	93.23797	6.493652	0.955867	0.971345	0.968155	0.9651224	0.017814	0.058414
Q53GG0	Epithelial protein lost in neoplasm beta variant (Fragment) OS=Homo sapiens PE=2 SV=1 - [Q53GG0_HUMAN]	8.56	5	5	5	759	85.20139	6.844238	1.00679	0.965445	0.922433	0.9648909	0.286148	0.3590107
F5H0C8	Enolase OS=Homo sapiens GN=ENO2 PE=1 SV=1 - [F5H0C8_HUMAN]	28.25	4	5	8	315	34.74056	4.868652	0.945481	1.02468	0.923778	0.9646476	0.368151	0.4415986
K7EPP6	ELKS/Rab6-interacting/CAST family member 1 (Fragment) OS=Homo sapiens GN=ERC1 PE=1 SV=1 - [K7EPP6_HUMAN]	7.85	2	2	2	344	40.58078	6.036621	1.0732	0.885556	0.935113	0.9646229	0.592999	0.6546972
Q59GZ2	PLEK protein variant (Fragment) OS=Homo sapiens PE=2 SV=1 - [Q59GZ2_HUMAN]	13.64	3	3	3	308	35.36996	8.733887	0.911267	1.03606	0.944717	0.9640143	0.436388	0.5081436
B4DPP8	cDNA FLJ53075, highly similar to Kininogen-1 OS=Homo sapiens PE=2 SV=1 - [B4DPP8_HUMAN]	36.63	14	14	24	415	46.4668	6.430176	0.976021	0.968482	0.947404	0.963969	0.0521111	0.1048105
B4DH19	cDNA FLJ54615, highly similar to Vesicle-fusing ATPase (EC 3.6.4.6) OS=Homo sapiens PE=2 SV=1 - [B4DH19_HUMAN]	16.77	11	11	13	650	72.21288	6.379395	0.985061	0.949955	0.95618	0.9637319	0.0785782	0.1371775
Q96K97	cDNA FLJ14416 fis, clone HEMBA1005202, highly similar to SIGNAL RECOGNITION PARTICLE 68 KD PROTEIN OS=Homo sapiens PE=2 SV=1 - [Q96K97_HUMAN]	7.76	2	2	3	335	38.20004	6.303223	0.957703	1.06392	0.868868	0.9634983	0.583761	0.6458525
B7Z478	Proteasome (Prosome, macropain) subunit, beta type, 2, isoform CRA_b OS=Homo sapiens GN=PSMB2 PE=2 SV=1 -	10.23	2	2	3	176	20.19434	7.444824	0.870941	0.983711	1.03502	0.9632256	0.527187	0.5948427

Biology Open • Supplementary information

	[B7Z478_HUMAN]													
P68133	Actin, alpha skeletal muscle OS=Homo sapiens GN=ACTA1 PE=1 SV=1 - [ACTS_HUMAN]	63.66	2	22	412	377	42.02385	5.38916	1.00622	0.943115	0.940164	0.9631674	0.229473	0.2989862
P02790	Hemopexin OS=Homo sapiens GN=HPX PE=1 SV=2 - [HEMO_HUMAN]	37.88	14	15	45	462	51.64328	7.02002	0.941456	0.971222	0.975359	0.9626789	0.0730114	0.1300072
A8K6N3	cDNA FLJ76886, highly similar to Homo sapiens loss of heterozygosity, 11, chromosomal region 2, gene A (LOH11CR2A), transcript variant 1, mRNA OS=Homo sapiens PE=2 SV=1 - [A8K6N3_HUMAN]	6.23	4	4	5	786	86.46003	6.580566	0.989247	0.911335	0.987366	0.9626495	0.282811	0.3556149
A0A024RC87	Ribonuclease/angiogenin inhibitor 1, isoform CRA_a OS=Homo sapiens GN=RNH1 PE=4 SV=1 - [A0A024RC87_HUMAN]	28.29	10	10	15	456	49.3848	4.843262	0.989856	0.942573	0.955439	0.9626225	0.117898	0.1822305
B2R694	Terpene cyclase/mutase family member OS=Homo sapiens PE=2 SV=1 - [B2R694_HUMAN]	2.87	2	2	2	732	83.37016	6.609863	0.926576	0.864545	1.09655	0.9625583	0.643342	0.7011309
B4DS43	Acetyltransferase component of pyruvate dehydrogenase complex OS=Homo sapiens PE=2 SV=1 - [B4DS43_HUMAN]	12.92	5	5	7	418	44.57155	6.20166	0.987398	0.987281	0.912967	0.9625485	0.269964	0.3419281
P14854	Cytochrome c oxidase subunit 6B1 OS=Homo sapiens GN=COX6B1 PE=1 SV=2 - [CX6B1_HUMAN]	26.74	2	2	2	86	10.18572	7.049316	0.99042	0.960539	0.935483	0.9621475	0.139961	0.2051832
B4E290	cDNA FLJ50039, highly similar to Homo sapiens solute carrier family 25, member 24, transcript variant 1, mRNA OS=Homo sapiens PE=2 SV=1 - [B4E290_HUMAN]	7.13	4	4	4	477	53.26234	6.609863	0.971812	0.976238	0.938245	0.9620983	0.0872507	0.1476718
P50135	Histamine N-methyltransferase OS=Homo sapiens GN=HNMT PE=1 SV=1 - [HNMT_HUMAN]	11.99	2	2	2	292	33.27358	5.338379	0.895291	1.06103	0.929143	0.9618224	0.528991	0.5964805
P35754	Glutaredoxin-1 OS=Homo sapiens GN=GLRX PE=1 SV=2 - [GLRX1_HUMAN]	11.32	2	2	4	106	11.76818	8.089355	0.945866	1.00802	0.931211	0.9616985	0.245296	0.3151146

D6RFM5	Succinate dehydrogenase [ubiquinone] flavoprotein subunit, mitochondrial OS=Homo sapiens GN=SDHA PE=1 SV=1 - [D6RFM5_HUMAN]	20.75	7	7	7	583	63.52669	7.239746	0.894458	1.09861	0.89176	0.9616086	0.631593	0.6903269
P0DMV8	Heat shock 70 kDa protein 1A OS=Homo sapiens GN=HSPA1A PE=1 SV=1 - [HS71A_HUMAN]	43.06	13	23	73	641	70.00904	5.655762	0.970711	0.962207	0.951411	0.9614429	0.0203384	0.0623036
B4E357	cDNA FLJ53282, highly similar to Dipeptidyl-peptidase 3 (EC 3.4.14.4) OS=Homo sapiens PE=2 SV=1 - [B4E357_HUMAN]	17.95	7	7	8	635	71.03398	5.17334	0.90776	0.981897	0.994619	0.9614251	0.290379	0.3629738
P02656	Apolipoprotein C-III OS=Homo sapiens GN=APOC3 PE=1 SV=1 - [APOC3_HUMAN]	27.27	2	2	8	99	10.8455	5.414551	0.963715	0.952824	0.96715	0.9612294	0.012179	0.0499708
O00232	26S proteasome non-ATPase regulatory subunit 12 OS=Homo sapiens GN=PSMD12 PE=1 SV=3 - [PSD12_HUMAN]	16.23	5	5	6	456	52.87058	7.649902	0.895419	1.04769	0.939	0.9607042	0.476955	0.5463596
Q96FQ6	Protein S100-A16 OS=Homo sapiens GN=S100A16 PE=1 SV=1 - [S10AG_HUMAN]	22.33	2	2	2	103	11.79397	6.785645	0.950683	0.999459	0.931455	0.9605325	0.190477	0.2597763
P27361	Mitogen-activated protein kinase 3 OS=Homo sapiens GN=MAPK3 PE=1 SV=4 - [MK03_HUMAN]	31.13	6	10	13	379	43.10812	6.741699	0.993916	0.893097	0.993507	0.9601733	0.356947	0.4301408
Q9Y3Q8	TSC22 domain family protein 4 OS=Homo sapiens GN=TSC22D4 PE=1 SV=2 - [T22D4_HUMAN]	6.08	2	2	2	395	41.0009	7.210449	0.905967	0.931135	1.0428	0.9599661	0.44153	0.512896
Q7Z4Y4	GTP:AMP phosphotransferase AK3, mitochondrial OS=Homo sapiens GN=AK3 PE=2 SV=1 - [Q7Z4Y4_HUMAN]	33.48	6	6	9	227	25.6046	9.305176	0.963232	0.937102	0.979327	0.9598868	0.0825895	0.1424175
Q96C86	m7GpppX diphosphatase OS=Homo sapiens GN=DCPS PE=1 SV=2 - [DCPS_HUMAN]	13.95	3	3	5	337	38.58502	6.379395	0.993147	0.929261	0.953436	0.9586146	0.156349	0.223947
B3KMV5	cDNA FLJ12728 fis, clone NT2RP2000040, highly similar to Protein FAM62A OS=Homo sapiens PE=2 SV=1 - [B3KMV5_HUMAN]	17.57	14	14	23	1104	122.829	5.846191	0.925488	0.980053	0.970114	0.9585517	0.132158	0.1972464

Biology Open • Supplementary information

P28331	NADH-ubiquinone oxidoreductase 75 kDa subunit, mitochondrial OS=Homo sapiens GN=NDUFS1 PE=1 SV=3 - [NDUS1_HUMAN]	21.6	11	11	17	727	79.41655	6.227051	1.04893	0.92522	0.899901	0.9580185	0.458119	0.5280681
B1ALA9	Ribose-phosphate pyrophosphokinase 1 OS=Homo sapiens GN=PRPS1 PE=1 SV=1 - [B1ALA9_HUMAN]	42.66	3	6	9	218	24.06846	7.693848	1.00059	0.949776	0.923073	0.9578124	0.204646	0.2746492
P54868	Hydroxymethylglutaryl-CoA synthase, mitochondrial OS=Homo sapiens GN=HMGCS2 PE=1 SV=1 - [HMCS2_HUMAN]	3.94	2	2	2	508	56.59942	8.162598	0.97275	0.987415	0.912237	0.9574673	0.205764	0.2750082
D3DVC4	Nestin, isoform CRA_c OS=Homo sapiens GN=NES PE=3 SV=1 - [D3DVC4_HUMAN]	11.1	15	15	19	1621	177.32	4.36084	0.98271	0.939889	0.949365	0.9573216	0.0814318	0.1409236
B7Z5H9	Beta-galactosidase OS=Homo sapiens PE=2 SV=1 - [B7Z5H9_HUMAN]	7.33	3	3	5	546	60.51366	6.990723	0.998764	0.900428	0.971131	0.9567744	0.277883	0.3505904
B7Z768	cDNA FLJ52138, highly similar to NADH dehydrogenase (ubiquinone) 1 alpha subcomplex subunit 8 (EC 1.6.5.3) OS=Homo sapiens PE=2 SV=1 - [B7Z768_HUMAN]	14.06	2	2	2	128	14.95342	7.254395	0.95115	1.01152	0.907471	0.9567124	0.287736	0.3603654
F5H4Z6	Folate receptor beta (Fragment) OS=Homo sapiens GN=FOLR2 PE=1 SV=1 - [F5H4Z6_HUMAN]	21.64	4	4	5	171	20.01332	7.840332	0.925058	0.951475	0.993533	0.9566887	0.16195	0.2291809
Q6FIA3	PACSIN2 protein OS=Homo sapiens GN=PACSIN2 PE=2 SV=1 - [Q6FIA3_HUMAN]	13.26	5	5	5	445	51.32099	5.38916	0.976458	0.909622	0.983242	0.9564406	0.204861	0.2747443
J9R021	Eukaryotic translation initiation factor 3 subunit A OS=Homo sapiens GN=eIF3a PE=2 SV=1 - [J9R021_HUMAN]	11.36	13	13	16	1382	166.3813	6.785645	0.972171	0.951751	0.944655	0.9561923	0.0336656	0.0810225
P31939	Bifunctional purine biosynthesis protein PURH OS=Homo sapiens GN=ATIC PE=1 SV=3 - [PUR9_HUMAN]	26.52	12	12	18	592	64.57535	6.712402	0.957533	1.0195	0.890995	0.956011	0.357578	0.4307479
Q96JB5	CDK5 regulatory subunit-associated protein 3 OS=Homo sapiens GN=CDK5RAP3 PE=1 SV=2 - [CK5P3_HUMAN]	9.09	4	5	5	506	56.88502	4.754395	0.960259	0.946306	0.960557	0.9557071	0.0110802	0.0493307

Biology Open • Supplementary information

Q6MZX7	Putative uncharacterized protein DKFZp686M24218 OS=Homo sapiens GN=DKFZp686M24218 PE=2 SV=1 - [Q6MZX7_HUMAN]	32.14	5	10	43	476	52.38711	7.76709	0.939315	1.01703	0.910597	0.9556463	0.297693	0.3707472
B1AUU8	Epidermal growth factor receptor substrate 15 OS=Homo sapiens GN=EPS15 PE=1 SV=1 - [B1AUU8_HUMAN]	4.46	3	3	3	762	83.60277	4.703613	0.972273	0.97569	0.918939	0.955634	0.137109	0.2021402
O14776	Transcription elongation regulator 1 OS=Homo sapiens GN=TCERG1 PE=1 SV=2 - [TCRG1_HUMAN]	1.73	2	2	2	1098	123.8233	8.645996	0.98733	0.866056	1.01346	0.9556169	0.431464	0.5032756
Q13884	Beta-1-syntrophin OS=Homo sapiens GN=SNTB1 PE=1 SV=3 - [SNTB1_HUMAN]	4.65	2	3	3	538	58.02506	8.631348	0.926095	0.895306	1.0445	0.9553015	0.429305	0.5014484
B2R983	cDNA, FLJ94267, highly similar to Homo sapiens glutathione S-transferase omega 1 (GSTO1), mRNA OS=Homo sapiens PE=2 SV=1 - [B2R983_HUMAN]	23.24	6	6	11	241	27.52103	6.595215	0.927854	1.03528	0.902523	0.9552177	0.385859	0.4587751
B3KPU0	PDZ and LIM domain protein 2 OS=Homo sapiens GN=PDLIM2 PE=1 SV=1 - [B3KPU0_HUMAN]	13.7	2	2	2	146	16.09284	7.312988	1.02818	0.910429	0.925977	0.954861	0.346118	0.4196309
O00505	Importin subunit alpha-4 OS=Homo sapiens GN=KPNA3 PE=1 SV=2 - [IMA4_HUMAN]	5.18	2	2	3	521	57.77485	4.944824	0.957369	0.894877	1.01209	0.9547782	0.313412	0.386203
B4DRW8	cDNA FLJ54537, highly similar to Homo sapiens pitrilysin metallopeptidase 1 (PITRM1), mRNA OS=Homo sapiens PE=2 SV=1 - [B4DRW8_HUMAN]	3.19	2	2	2	972	109.7094	7.283691	0.934094	0.918141	1.01156	0.9545982	0.256214	0.3265378
E9PF84	Isocitrate dehydrogenase [NAD] subunit, mitochondrial (Fragment) OS=Homo sapiens GN=IDH3G PE=1 SV=1 - [E9PF84_HUMAN]	10.88	2	2	2	285	31.40221	8.104004	0.918965	0.909654	1.03489	0.9545031	0.375962	0.4490584
P62917	60S ribosomal protein L8 OS=Homo sapiens GN=RPL8 PE=1 SV=2 - [RL8_HUMAN]	16.73	5	5	7	257	28.00729	11.03369	0.9898	0.955791	0.917594	0.9543949	0.160309	0.22762
P17174	Aspartate aminotransferase, cytoplasmic OS=Homo sapiens GN=GOT1 PE=1 SV=3 - [AATC_HUMAN]	16.22	6	6	6	413	46.21853	7.005371	0.936167	0.982505	0.943643	0.9541048	0.0855591	0.1459031

Biology Open • Supplementary information

Q9GZM7	Tubulointerstitial nephritis antigen-like OS=Homo sapiens GN=TINAGL1 PE=1 SV=1 - [TINAL_HUMAN]	32.76	12	12	22	467	52.35296	6.990723	0.927908	0.980372	0.953884	0.9540545	0.093649	0.1547861
P30084	Enoyl-CoA hydratase, mitochondrial OS=Homo sapiens GN=ECHS1 PE=1 SV=4 - [ECHM_HUMAN]	30.69	7	7	10	290	31.36713	8.074707	0.923179	0.993561	0.944759	0.9538331	0.156854	0.2242634
Q9H4M9	EH domain-containing protein 1 OS=Homo sapiens GN=EHD1 PE=1 SV=2 - [EHD1_HUMAN]	20.6	6	9	18	534	60.58867	6.82959	0.956443	0.998315	0.906309	0.953689	0.223755	0.2936839
Q9BVJ8	HEXA protein (Fragment) OS=Homo sapiens GN=HEXA PE=2 SV=2 - [Q9BVJ8_HUMAN]	11.74	3	4	6	409	47.06444	4.995605	0.98264	0.936349	0.941812	0.9536006	0.0864284	0.1468675
P62312	U6 snRNA-associated Sm-like protein LSm6 OS=Homo sapiens GN=LSM6 PE=1 SV=1 - [LSM6_HUMAN]	23.75	2	2	2	80	9.121768	9.583496	0.96953	0.976363	0.91419	0.953361	0.141331	0.2067439
B2R780	cDNA, FLJ93320, highly similar to Homo sapiens angiopoietin-like 2 (ANGPTL2), mRNA OS=Homo sapiens PE=2 SV=1 - [B2R780_HUMAN]	4.67	2	2	2	493	57.01442	7.518066	0.923604	1.02486	0.91149	0.9533179	0.323577	0.3970139
G5E9D8	Inter-alpha (Globulin) inhibitor H5, isoform CRA_e OS=Homo sapiens GN=ITIH5 PE=1 SV=1 - [G5E9D8_HUMAN]	9.26	5	5	5	702	78.38908	9.012207	0.992112	1.02967	0.837564	0.9531158	0.508774	0.5767582
P50453	Serpin B9 OS=Homo sapiens GN=SERPINB9 PE=1 SV=1 - [SPB9_HUMAN]	23.14	6	7	8	376	42.37631	5.858887	0.92825	0.949021	0.978942	0.9520707	0.0827085	0.14255
Q5U000	Cathepsin Z OS=Homo sapiens PE=2 SV=1 - [Q5U000_HUMAN]	7.26	2	2	3	303	33.84622	7.10791	0.948295	0.936691	0.971061	0.9520154	0.0415197	0.0920394
O00291	Huntingtin-interacting protein 1 OS=Homo sapiens GN=HIP1 PE=1 SV=5 - [HIP1_HUMAN]	4.53	4	4	5	1037	116.1485	5.300293	0.928469	0.9816	0.945917	0.9519952	0.0917252	0.1526499
E7ETJ9	Nucleolysin TIAR (Fragment) OS=Homo sapiens GN=TIAL1 PE=1 SV=1 - [E7ETJ9_HUMAN]	13.64	2	2	2	132	14.60725	8.147949	0.933323	0.994931	0.927625	0.9519597	0.155568	0.2230401
Q6MZU1	Putative uncharacterized protein DKFZp686A1195 OS=Homo sapiens GN=DKFZp686A1195 PE=2 SV=1 - [Q6MZU1_HUMAN]	4.43	5	6	6	1692	189.502	6.800293	0.917645	0.91613	1.02168	0.9518178	0.301787	0.3744681

Biology Open • Supplementary information

G3V2T4	Legumain (Fragment) OS=Homo sapiens GN=LGMN PE=1 SV=1 - [G3V2T4_HUMAN]	22.96	2	2	3	135	14.62645	6.049316	0.96257	0.899785	0.992299	0.9515516	0.217599	0.2871628
Q9Y5S2	Serine/threonine-protein kinase MRCK beta OS=Homo sapiens GN=CDC42BPB PE=1 SV=2 - [MRCKB_HUMAN]	2.28	3	3	3	1711	194.1928	6.366699	0.988333	1.00129	0.864382	0.9513341	0.380776	0.4540073
P38606	V-type proton ATPase catalytic subunit A OS=Homo sapiens GN=ATP6V1A PE=1 SV=2 - [VATA_HUMAN]	15.4	6	6	8	617	68.2605	5.516113	0.907431	0.956625	0.989475	0.9511772	0.177135	0.2454368
Q9Y2A7	Nck-associated protein 1 OS=Homo sapiens GN=NCKAP1 PE=1 SV=1 - [NCKP1_HUMAN]	6.74	5	5	7	1128	128.7065	6.624512	0.997483	0.909982	0.944289	0.9505847	0.191732	0.2608572
A0A0D9SFB3	ATP-dependent RNA helicase DDX3X OS=Homo sapiens GN=DDX3X PE=1 SV=1 - [A0A0D9SFB3_HUMAN]	17.97	8	9	10	640	70.7961	7.356934	0.946135	0.929292	0.975387	0.9502713	0.066138	0.1216063
Q53H26	Transferrin variant (Fragment) OS=Homo sapiens PE=2 SV=1 - [Q53H26_HUMAN]	58.74	47	47	191	698	77.02959	7.034668	0.938713	0.967457	0.942343	0.9495043	0.0305689	0.0773154
P05165	Propionyl-CoA carboxylase alpha chain, mitochondrial OS=Homo sapiens GN=PCCA PE=1 SV=4 - [PCCA_HUMAN]	4.81	3	3	3	728	80.0081	7.518066	0.977385	0.908816	0.959721	0.9486409	0.129727	0.194648
Q63ZY3	KN motif and ankyrin repeat domain-containing protein 2 OS=Homo sapiens GN=KANK2 PE=1 SV=1 - [KANK2_HUMAN]	17.27	12	12	17	851	91.11788	5.630371	0.971106	0.926656	0.948085	0.9486155	0.0570945	0.111536
P55036	26S proteasome non-ATPase regulatory subunit 4 OS=Homo sapiens GN=PSMD4 PE=1 SV=1 - [PSMD4_HUMAN]	23.87	6	6	6	377	40.7112	4.79248	0.930411	0.967384	0.946143	0.9479794	0.0398854	0.0894712
Q96GX3	KIAA0118 protein (Fragment) OS=Homo sapiens PE=2 SV=1 - [Q96GX3_HUMAN]	13.14	2	2	2	175	19.33573	6.902832	0.900447	0.947391	0.994186	0.9473411	0.191055	0.2602297
G5EA31	Protein transport protein Sec24C OS=Homo sapiens GN=SEC24C PE=1 SV=1 - [G5EA31_HUMAN]	5.85	4	4	4	1042	111.9136	6.366699	0.925977	0.88695	1.02876	0.947228	0.338398	0.4121904
B2R932	cDNA, FLJ94187, highly similar to Homo sapiens CD99 antigen (CD99), mRNA OS=Homo sapiens PE=2 SV=1 - [B2R932_HUMAN]	12.97	2	2	3	185	18.93541	4.817871	0.778774	0.984968	1.07781	0.9471836	0.610706	0.6713997

Biology Open • Supplementary information

B4DGP4	cDNA FLJ53288, moderately similar to LIM domain-binding protein 3 OS=Homo sapiens PE=2 SV=1 - [B4DGP4_HUMAN]	22.38	10	10	14	648	69.60721	8.279785	0.925048	0.910559	1.00582	0.9471433	0.216441	0.2860807
B3KY11	cDNA FLJ46571 fis, clone THYMU3041428, highly similar to Probable ATP-dependent RNA helicase DDX23 (EC 3.6.1.-) OS=Homo sapiens PE=2 SV=1 - [B3KY11_HUMAN]	2.75	2	2	3	800	93.17667	9.495605	0.968529	0.981702	0.889946	0.9467256	0.203979	0.2741044
A0A087WYF1	Laminin subunit alpha-2 OS=Homo sapiens GN=LAMA2 PE=1 SV=1 - [A0A087WYF1_HUMAN]	3.91	8	8	8	3118	343.1963	6.366699	0.938144	0.936913	0.96467	0.9465758	0.0275404	0.0734628
J3QRU4	Vesicle-associated membrane protein 2 OS=Homo sapiens GN=VAMP2 PE=4 SV=2 - [J3QRU4_HUMAN]	35.4	3	3	4	113	12.24347	6.697754	0.924807	0.905759	1.00827	0.9462791	0.230035	0.2995315
B4E3L7	cDNA FLJ52058, highly similar to Transforming acidic coiled-coil-containing protein 1 OS=Homo sapiens PE=2 SV=1 - [B4E3L7_HUMAN]	11.93	2	2	2	243	27.62813	4.995605	0.943092	0.912748	0.982931	0.946257	0.118171	0.1825691
B2R9F2	cDNA, FLJ94361, highly similar to Homo sapiens serine (or cysteine) proteinase inhibitor, clade A(alpha-1 antiproteinase, antitrypsin), member 6 (SERPINA6), mRNA OS=Homo sapiens PE=2 SV=1 - [B2R9F2_HUMAN]	6.91	3	3	4	405	45.0659	6.036621	0.935269	0.986831	0.91626	0.9461198	0.125027	0.1902951
A0A0G2JL54	Complement C4-B OS=Homo sapiens GN=C4B_2 PE=1 SV=1 - [A0A0G2JL54_HUMAN]	28.03	33	43	81	1698	187.5979	7.327637	0.928816	0.995889	0.913338	0.9460142	0.166797	0.2347642
P35052	Glypican-1 OS=Homo sapiens GN=GPC1 PE=1 SV=2 - [GPC1_HUMAN]	6.45	2	2	2	558	61.64102	7.29834	0.885567	0.970448	0.981771	0.9459287	0.216836	0.286491
O15061	Synemin OS=Homo sapiens GN=SYNM PE=1 SV=2 - [SYNEM_HUMAN]	14.57	20	20	23	1565	172.6633	5.160645	0.959857	0.919426	0.958051	0.945778	0.0543639	0.1077411
P02765	Alpha-2-HS-glycoprotein OS=Homo sapiens GN=AHSG PE=1 SV=1 - [FETUA_HUMAN]	22.62	9	9	17	367	39.29971	5.719238	0.915831	0.953213	0.968205	0.9457497	0.0734367	0.130627
P08514	Integrin alpha-IIb OS=Homo sapiens GN=ITGA2B PE=1 SV=3 - [ITA2B_HUMAN]	7.22	6	6	6	1039	113.3055	5.376465	0.938185	0.925402	0.973409	0.9456654	0.0632452	0.1180833

A0A024R962	HCG40889, isoform CRA_b OS=Homo sapiens GN=hCG_40889 PE=4 SV=1 - [A0A024R962_HUMAN]	29.41	2	28	46	1231	138.9787	6.624512	0.944563	0.900915	0.991078	0.9455187	0.171436	0.2388111
Q8IVF2	Protein AHNAK2 OS=Homo sapiens GN=AHNAK2 PE=1 SV=2 - [AHNK2_HUMAN]	1.62	3	3	3	5795	616.2424	5.36377	0.916463	0.997408	0.922468	0.9454463	0.171155	0.2388111
O94760	N(G),N(G)-dimethylarginine dimethylaminohydrolase 1 OS=Homo sapiens GN=DDAH1 PE=1 SV=3 - [DDAH1_HUMAN]	21.4	4	5	8	285	31.10193	5.808105	0.945752	0.943577	0.946849	0.9453927	0.000309993	0.0212234
B4DRA5	cDNA FLJ61346, highly similar to Protein transport protein Sec23B OS=Homo sapiens PE=2 SV=1 - [B4DRA5_HUMAN]	4.04	2	2	2	742	83.75619	7.093262	1.10906	0.849521	0.877385	0.9453215	0.574638	0.6369187
Q13617	Cullin-2 OS=Homo sapiens GN=CUL2 PE=1 SV=2 - [CUL2_HUMAN]	5.23	3	4	5	745	86.92677	6.91748	0.949815	0.987459	0.898252	0.9451752	0.168062	0.2361519
Q75L23	Putative uncharacterized protein PSMC2 (Fragment) OS=Homo sapiens GN=PSMC2 PE=3 SV=1 - [Q75L23_HUMAN]	20.05	7	7	8	409	45.80274	6.100098	0.96279	0.914122	0.958459	0.9451241	0.0717702	0.1288404
B4DDM5	cDNA FLJ53298, highly similar to Peroxisomal multifunctional enzyme type 2 OS=Homo sapiens PE=2 SV=1 - [B4DDM5_HUMAN]	13.67	6	6	9	717	77.37907	8.689941	0.928636	0.840382	1.06526	0.944759	0.487317	0.5563467
Q59GC9	Syntaxin binding protein 1 variant (Fragment) OS=Homo sapiens PE=2 SV=1 - [Q59GC9_HUMAN]	6.04	2	2	2	414	47.30532	6.55127	0.944131	0.954355	0.934512	0.9443324	0.010426	0.0489337
B7Z544	cDNA FLJ51742, highly similar to Inter-alpha-trypsin inhibitor heavy chain H4 OS=Homo sapiens PE=2 SV=1 - [B7Z544_HUMAN]	18.81	16	16	25	888	98.28629	6.595215	0.945829	0.962871	0.924016	0.9442387	0.0383423	0.0876358
Q9Y570	Protein phosphatase methylesterase 1 OS=Homo sapiens GN=PPME1 PE=1 SV=3 - [PPME1_HUMAN]	5.7	2	2	2	386	42.2882	5.973145	0.925203	0.919757	0.987204	0.9440547	0.122611	0.1876303
O43665	Regulator of G-protein signaling 10 OS=Homo sapiens GN=RGS10 PE=1 SV=2 - [RGS10_HUMAN]	20.81	3	3	3	173	20.22296	5.490723	1.00957	0.947569	0.874855	0.9439977	0.286885	0.3598021

Biology Open • Supplementary information

B2R9T9	cDNA, FLJ94551 OS=Homo sapiens PE=2 SV=1 - [B2R9T9_HUMAN]	9.05	3	3	5	243	26.17763	10.66748	1.00756	0.907748	0.916254	0.9438545	0.22093	0.2907955
P62879	Guanine nucleotide-binding protein G(I)/G(S)/G(T) subunit beta-2 OS=Homo sapiens GN=GNB2 PE=1 SV=3 - [GBB2_HUMAN]	24.41	2	7	10	340	37.30706	5.998535	0.991074	0.822714	1.01776	0.9438484	0.454823	0.5250941
Q16762	Thiosulfate sulfurtransferase OS=Homo sapiens GN=TST PE=1 SV=4 - [THTR_HUMAN]	18.52	4	4	5	297	33.40784	7.254395	0.928221	0.957888	0.945016	0.9437085	0.0224995	0.0650391
Q12768	WASH complex subunit strumpellin OS=Homo sapiens GN=KIAA0196 PE=1 SV=1 - [STRUM_HUMAN]	5.26	5	5	5	1159	134.2009	6.976074	0.986873	0.884851	0.958867	0.9435302	0.204665	0.2746492
Q9NZZ3	Charged multivesicular body protein 5 OS=Homo sapiens GN=CHMP5 PE=1 SV=1 - [CHMP5_HUMAN]	17.81	2	2	2	219	24.55536	4.830566	0.949423	0.979177	0.901414	0.9433383	0.129508	0.1945639
P49756	RNA-binding protein 25 OS=Homo sapiens GN=RBM25 PE=1 SV=3 - [RBM25_HUMAN]	4.51	3	3	4	843	100.1244	6.315918	0.908836	0.957755	0.962005	0.9428655	0.0787583	0.1373503
B4DNN2	DnaJ (Hsp40) homolog, subfamily B, member 4, isoform CRA_b OS=Homo sapiens GN=DNAJB4 PE=2 SV=1 - [B4DNN2_HUMAN]	20.27	2	4	5	222	24.93984	8.953613	0.884401	1.01848	0.923771	0.9422187	0.283599	0.3564516
P08697	Alpha-2-antiplasmin OS=Homo sapiens GN=SERPINF2 PE=1 SV=3 - [A2AP_HUMAN]	19.96	7	7	9	491	54.53107	6.290527	0.938687	0.952754	0.935191	0.9422108	0.00851685	0.0456887
P42126	Enoyl-CoA delta isomerase 1, mitochondrial OS=Homo sapiens GN=ECI1 PE=1 SV=1 - [ECI1_HUMAN]	12.58	3	3	4	302	32.79524	8.543457	0.965829	0.931114	0.92926	0.9420679	0.0396522	0.08917
B3KQM8	cDNA FLJ90752 fis, clone PLACE3000181, highly similar to Homo sapiens protocadherin 1 (cadherin-like 1) (PCDH1), transcript variant 2, mRNA OS=Homo sapiens PE=2 SV=1 - [B3KQM8_HUMAN]	3.49	2	2	2	1003	108.5414	5.262207	0.936709	0.906713	0.982466	0.9419628	0.11888	0.183421
Q9NUV9	GTPase IMAP family member 4 OS=Homo sapiens GN=GIMAP4 PE=1 SV=1 - [GIMA4_HUMAN]	17.63	4	4	4	329	37.51038	7.811035	0.918341	0.837421	1.06989	0.9418837	0.483516	0.552753

Biology Open • Supplementary information

Q04760	Lactoylglutathione lyase OS=Homo sapiens GN=GLO1 PE=1 SV=4 - [LGUL_HUMAN]	29.35	5	5	5	184	20.76425	5.312988	0.973534	0.94837	0.903714	0.9418729	0.104394	0.1672379
Q15628	Tumor necrosis factor receptor type 1-associated DEATH domain protein OS=Homo sapiens GN=TRADD PE=1 SV=2 - [TRADD_HUMAN]	8.33	2	2	2	312	34.22581	6.265137	0.983305	0.912066	0.927006	0.9407924	0.112082	0.1750796
B4DS66	cDNA FLJ54290, highly similar to Mitochondrial inner membrane protein OS=Homo sapiens PE=2 SV=1 - [B4DS66_HUMAN]	21.82	12	12	14	660	73.33804	5.98584	0.938785	0.939533	0.943889	0.9407358	0.000720263	0.0242397
P30041	Peroxiredoxin-6 OS=Homo sapiens GN=PRDX6 PE=1 SV=3 - [PRDX6_HUMAN]	55.8	13	13	26	224	25.01919	6.379395	0.931579	0.959386	0.931017	0.9406603	0.024009	0.0675863
A0A024R3R6	Nucleoporin 133kDa, isoform CRA_a OS=Homo sapiens GN=NUP133 PE=4 SV=1 - [A0A024R3R6_HUMAN]	2.85	2	2	2	981	110.4465	4.906738	0.949179	0.921209	0.950064	0.9401505	0.0241549	0.0677418
J3KT25	Protein IMPACT (Fragment) OS=Homo sapiens GN=IMPACT PE=1 SV=2 - [J3KT25_HUMAN]	16.67	2	2	2	180	20.49314	4.411621	1.01867	0.859088	0.942688	0.9401502	0.323628	0.3970139
B3KMP6	cDNA FLJ11861 fis, clone HEMBA1006885, highly similar to Proline synthetase co-transcribed bacterial homolog protein OS=Homo sapiens PE=2 SV=1 - [B3KMP6_HUMAN]	15.32	3	3	3	235	25.92115	6.430176	0.960789	0.947808	0.91172	0.9401056	0.0551448	0.1086526
A8K4T6	cDNA FLJ76282, highly similar to Homo sapiens proteasome (prosome, macropain) 26S subunit, non-ATPase, 5 (PSMD5), mRNA OS=Homo sapiens PE=2 SV=1 - [A8K4T6_HUMAN]	19.25	7	7	9	504	56.1884	5.566895	0.920411	0.930338	0.968798	0.939849	0.055236	0.108769
Q14651	Plastin-1 OS=Homo sapiens GN=PLS1 PE=1 SV=2 - [PLSI_HUMAN]	5.88	3	3	4	629	70.20926	5.414551	0.957588	0.882003	0.979576	0.9397224	0.178181	0.2466841
O19712	MHC class II HLA-DQ-beta-1 (Fragment) OS=Homo sapiens GN=HLA-DQB1 PE=2 SV=1 - [O19712_HUMAN]	17.47	3	3	4	229	26.3475	7.884277	0.975847	0.927822	0.913989	0.9392196	0.0833829	0.1434203
B3KPC7	Actin-related protein 2/3 complex subunit 5 OS=Homo sapiens PE=2 SV=1 - [B3KPC7_HUMAN]	16.34	2	2	2	153	16.96079	6.023926	0.978128	0.960586	0.878867	0.9391936	0.185134	0.2540246

Biology Open • Supplementary information

A6NLG9	cDNA FLJ36740 fis, clone UTERU2013322, highly similar to Biglycan OS=Homo sapiens PE=2 SV=1 - [A6NLG9_HUMAN]	55.05	8	14	45	307	34.85337	8.865723	0.923492	0.953676	0.938974	0.9387142	0.0196254	0.0612255
Q01813	ATP-dependent 6-phosphofructokinase, platelet type OS=Homo sapiens GN=PFKP PE=1 SV=2 - [PFKAP_HUMAN]	23.47	15	15	19	784	85.54154	7.547363	0.929809	0.978474	0.907074	0.9384521	0.0998493	0.1621832
B5MCV4	Complement C1s subcomponent (Fragment) OS=Homo sapiens GN=C1S PE=1 SV=1 - [B5MCV4_HUMAN]	8.33	2	2	2	168	19.30182	4.271973	0.978539	0.871346	0.965067	0.9383174	0.208781	0.2786765
P30043	Flavin reductase (NADPH) OS=Homo sapiens GN=BLVRB PE=1 SV=3 - [BLVRB_HUMAN]	50.49	7	7	18	206	22.10543	7.649902	0.939844	0.944664	0.929208	0.9379052	0.00536294	0.0401071
B3KPN7	cDNA FLJ32002 fis, clone NT2RP7009394, highly similar to Exocyst complex component 2 OS=Homo sapiens PE=2 SV=1 - [B3KPN7_HUMAN]	3.25	2	2	2	924	104.0584	6.82959	0.843864	0.975109	0.994681	0.9378846	0.319918	0.393218
A0A024R1M8	Apolipoprotein L, 2, isoform CRA_a OS=Homo sapiens GN=APOL2 PE=4 SV=1 - [A0A024R1M8_HUMAN]	11.57	3	3	4	337	37.05538	6.741699	0.911556	0.934479	0.966885	0.9376399	0.0603114	0.1149713
A6NL93	Non-histone chromosomal protein HMG-14 OS=Homo sapiens GN=HMGN1 PE=1 SV=1 - [A6NL93_HUMAN]	25.56	2	2	2	90	9.52992	9.070801	0.968585	0.957909	0.886178	0.9375574	0.137223	0.2022147
B7Z8Y6	cDNA FLJ58394, highly similar to Platelet endothelial cell adhesion molecule OS=Homo sapiens PE=2 SV=1 - [B7Z8Y6_HUMAN]	17.05	8	8	10	733	81.72831	6.712402	0.908023	0.963601	0.941014	0.9375457	0.0607439	0.1153342
Q16647	Prostacyclin synthase OS=Homo sapiens GN=PTGIS PE=1 SV=1 - [PTGIS_HUMAN]	17.4	7	7	8	500	57.06782	7.312988	0.858619	0.979623	0.974276	0.9375062	0.254217	0.3247262
B7ZMC1	Uncharacterized protein OS=Homo sapiens PE=2 SV=1 - [B7ZMC1_HUMAN]	4.81	2	2	2	395	43.73369	6.990723	0.94109	0.891507	0.979727	0.937441	0.133912	0.1989869
B3KMJ1	cDNA FLJ11167 fis, clone PLACE1007257, highly similar to Protein diaphanous homolog 2 OS=Homo sapiens PE=2 SV=1 - [B3KMJ1_HUMAN]	3.47	2	2	2	634	72.06053	7.884277	0.963807	0.957605	0.890537	0.9373163	0.11615	0.1801834

Biology Open • Supplementary information

F6WIT2	Serine/threonine-protein phosphatase 2A activator OS=Homo sapiens GN=PPP2R4 PE=1 SV=1 - [F6WIT2_HUMAN]	15.28	4	4	6	288	32.91484	6.932129	0.902611	0.98598	0.922991	0.9371942	0.129361	0.1944436
Q16519	Protein S (Fragment) OS=Homo sapiens GN=PROS1 PE=4 SV=1 - [Q16519_HUMAN]	6	5	5	6	650	72.41553	5.731934	0.928452	0.943955	0.938247	0.9368849	0.00510495	0.0401071
Q92888	Rho guanine nucleotide exchange factor 1 OS=Homo sapiens GN=ARHGEF1 PE=1 SV=2 - [ARHG1_HUMAN]	5.81	4	4	4	912	102.3714	5.655762	0.920782	0.952457	0.937074	0.936771	0.0202844	0.0623036
B5LY71	Uridine monophosphate synthetase isoform I OS=Homo sapiens GN=UMPS PE=2 SV=1 - [B5LY71_HUMAN]	6.25	2	2	2	480	52.24364	7.239746	0.971297	0.889086	0.949775	0.9367193	0.123811	0.1891247
P43652	Afamin OS=Homo sapiens GN=AFM PE=1 SV=1 - [AFAM_HUMAN]	16.53	11	11	14	599	69.02402	5.896973	0.937294	0.933472	0.939305	0.9366906	0.000729433	0.0242397
E9PN17	ATP synthase subunit g, mitochondrial OS=Homo sapiens GN=ATP5L PE=1 SV=1 - [E9PN17_HUMAN]	64.47	4	4	5	76	8.446677	10.28662	1.00768	0.883544	0.918753	0.9366584	0.228499	0.2981762
P07355	Annexin A2 OS=Homo sapiens GN=ANXA2 PE=1 SV=2 - [ANXA2_HUMAN]	70.5	32	32	130	339	38.57982	7.752441	0.949636	0.932039	0.928102	0.9365921	0.0107256	0.0491288
E5RJR5	S-phase kinase-associated protein 1 OS=Homo sapiens GN=SKP1 PE=1 SV=1 - [E5RJR5_HUMAN]	15.95	2	2	2	163	18.7083	4.703613	0.973798	0.964486	0.871179	0.9364878	0.192146	0.2611057
B4DQX8	cDNA FLJ51723, highly similar to DCC-interacting protein 13 alpha (Fragment) OS=Homo sapiens PE=2 SV=1 - [B4DQX8_HUMAN]	9	4	5	5	633	71.29444	5.79541	0.890323	0.993509	0.925496	0.9364431	0.170727	0.2385001
P00739	Haptoglobin-related protein OS=Homo sapiens GN=HPR PE=2 SV=2 - [HPTR_HUMAN]	35.63	2	13	44	348	39.00468	7.093262	1.07184	0.920707	0.815054	0.935867	0.480117	0.5492383
B4DPP0	cDNA FLJ51032, highly similar to CD9 antigen OS=Homo sapiens PE=2 SV=1 - [B4DPP0_HUMAN]	12.06	2	2	3	141	15.89521	6.741699	0.973857	0.902121	0.931287	0.9357549	0.0909889	0.1520224
P11532	Dystrophin OS=Homo sapiens GN=DMD PE=1 SV=3 - [DMD_HUMAN]	4.29	12	13	16	3685	426.4843	5.884277	0.943859	0.91783	0.94552	0.9357364	0.0189153	0.0603321

B4DQ47	cDNA FLJ58036, highly similar to Homo sapiens sterile alpha motif and leucine zipper containing kinase AZK (ZAK), transcript variant 2, mRNA OS=Homo sapiens PE=2 SV=1 - [B4DQ47_HUMAN]	7.63	2	2	2	354	40.18231	5.224121	0.940278	0.877355	0.989435	0.935689	0.185877	0.2549407
Q13308	Inactive tyrosine-protein kinase 7 OS=Homo sapiens GN=PTK7 PE=1 SV=2 - [PTK7_HUMAN]	4.21	3	3	3	1070	118.3169	7.093262	0.918169	0.901238	0.987589	0.9356653	0.135242	0.2003476
P50897	Palmitoyl-protein thioesterase 1 OS=Homo sapiens GN=PPT1 PE=1 SV=1 - [PPT1_HUMAN]	10.78	2	2	4	306	34.17125	6.521973	0.926381	0.90531	0.97434	0.9353439	0.0869724	0.1474958
B3KWQ6	cDNA FLJ43599 fis, clone SMINT2017781, highly similar to PERIPHERIN OS=Homo sapiens PE=2 SV=1 - [B3KWQ6_HUMAN]	48.51	19	23	48	470	53.64843	5.465332	0.918794	0.965523	0.921669	0.9353287	0.0505506	0.102771
P25788	Proteasome subunit alpha type-3 OS=Homo sapiens GN=PSMA3 PE=1 SV=2 - [PSA3_HUMAN]	11.37	3	3	4	255	28.41508	5.325684	0.905794	1.00783	0.89219	0.9352714	0.218093	0.2877026
B4DY50	cDNA FLJ55528, highly similar to Transducin beta-like 2 protein OS=Homo sapiens PE=2 SV=1 - [B4DY50_HUMAN]	7.79	3	3	3	411	45.92017	9.480957	0.974299	0.967174	0.861273	0.9342486	0.213807	0.2831521
E9PF10	Nuclear pore complex protein Nup155 OS=Homo sapiens GN=NUP155 PE=1 SV=1 - [E9PF10_HUMAN]	4.75	4	4	4	1327	147.9996	6.303223	0.971564	0.879282	0.951537	0.9341277	0.143143	0.2090329
Q9C0C2	182 kDa tankyrase-1-binding protein OS=Homo sapiens GN=TNKS1BP1 PE=1 SV=4 - [TB182_HUMAN]	7.17	8	8	11	1729	181.685	4.855957	0.914008	0.967696	0.919868	0.9338574	0.0601848	0.1148843
P05091	Aldehyde dehydrogenase, mitochondrial OS=Homo sapiens GN=ALDH2 PE=1 SV=2 - [ALDH2_HUMAN]	38.3	14	16	27	517	56.34563	7.049316	0.934698	0.928596	0.937944	0.9337462	0.00170622	0.0307118
Q96DP0	cDNA FLJ31479 fis, clone NT2NE2001634, moderately similar to NADH-UBIQUINONE OXIDOREDUCTASE 9 KD SUBUNIT (EC 1.6.5.3) OS=Homo sapiens PE=2 SV=1 - [Q96DP0_HUMAN]	8.99	3	3	3	456	49.21527	8.98291	0.934517	0.837817	1.02681	0.9330483	0.344642	0.4179911

Biology Open • Supplementary information

D6RGE2	Isochorismatase domain-containing protein 1 (Fragment) OS=Homo sapiens GN=ISOC1 PE=1 SV=1 - [D6RGE2_HUMAN]	22.34	3	3	4	188	20.22658	7.210449	0.966543	0.946463	0.885685	0.9328971	0.109988	0.1730062
Q9HAB8	Phosphopantothenate--cysteine ligase OS=Homo sapiens GN=PPCS PE=1 SV=2 - [PPCS_HUMAN]	6.75	2	2	2	311	33.98379	6.712402	0.952785	0.827616	1.01554	0.9319817	0.343331	0.4166997
K7EL81	Protein phosphatase 1 regulatory subunit 12C (Fragment) OS=Homo sapiens GN=PPP1R12C PE=1 SV=1 - [K7EL81_HUMAN]	4.07	2	2	3	737	80.26157	5.452637	0.862362	0.896787	1.0361	0.9317487	0.327502	0.4010833
B4DDH8	cDNA FLJ55184, highly similar to Homo sapiens leukocyte receptor cluster (LRC) member 4 (LENG4), mRNA OS=Homo sapiens PE=2 SV=1 - [B4DDH8_HUMAN]	6.17	2	2	2	454	50.53007	8.98291	0.962676	0.878411	0.953747	0.9316113	0.124763	0.1902239
B2RAW0	cDNA, FLJ95154, highly similar to Homo sapiens disabled homolog 2, mitogen-responsive phosphoprotein (Drosophila) (DAB2), mRNA OS=Homo sapiens PE=2 SV=1 - [B2RAW0_HUMAN]	4.16	2	2	2	770	82.41906	5.528809	1.03866	0.913321	0.839568	0.9305177	0.354377	0.427958
A0A024R7L5	UPF1 regulator of nonsense transcripts homolog (Yeast), isoform CRA_b OS=Homo sapiens GN=UPF1 PE=4 SV=1 - [A0A024R7L5_HUMAN]	10.2	9	9	11	1118	122.958	6.683105	0.945117	0.931097	0.91531	0.930508	0.0150054	0.0541506
B2RBR9	cDNA, FLJ95650, highly similar to Homo sapiens karyopherin (importin) beta 1 (KPNB1), mRNA OS=Homo sapiens PE=2 SV=1 - [B2RBR9_HUMAN]	18.49	11	11	17	876	97.12203	4.779785	0.921311	0.912147	0.957691	0.9303833	0.0376704	0.0869204
A2NB44	Cold agglutinin FS-2 H-chain (Fragment) OS=Homo sapiens GN=IGH@ PE=2 SV=1 - [A2NB44_HUMAN]	21.93	2	2	3	114	12.75322	7.10791	0.833847	0.995155	0.961507	0.9301699	0.291127	0.3635061
P28289	Tropomodulin-1 OS=Homo sapiens GN=TMOD1 PE=1 SV=1 - [TMOD1_HUMAN]	16.71	5	6	8	359	40.54389	5.097168	0.93945	0.945917	0.904005	0.9297906	0.0327466	0.0800919

B2R815	cDNA, FLJ93695, highly similar to Homo sapiens serpin peptidase inhibitor, clade A (alpha-1 antiproteinase, antitrypsin), member 4 (SERPINA4), mRNA OS=Homo sapiens PE=2 SV=1 - [B2R815_HUMAN]	22.95	8	8	8	427	48.49513	7.752441	0.899543	0.894372	0.99523	0.929715	0.165316	0.2331644
F2RM35	Serine protease OS=Homo sapiens GN=factor IX F9 PE=2 SV=1 - [F2RM35_HUMAN]	6.51	2	2	2	461	51.67885	5.38916	0.941985	0.899784	0.947304	0.9296909	0.0427966	0.0935455
Q9BUF5	Tubulin beta-6 chain OS=Homo sapiens GN=TUBB6 PE=1 SV=1 - [TBB6_HUMAN]	36.77	6	14	41	446	49.825	4.881348	1.01004	0.903833	0.872357	0.9287425	0.22924	0.2989127
X5D2J9	General transcription factor IIi isoform D (Fragment) OS=Homo sapiens GN=GTF2I PE=2 SV=1 - [X5D2J9_HUMAN]	7.11	5	5	5	957	107.902	7.942871	0.921076	0.920899	0.943925	0.9286331	0.011285	0.0493721
A0A0G2JMS7	Protein scribble homolog OS=Homo sapiens GN=SCRIB PE=1 SV=1 - [A0A0G2JMS7_HUMAN]	1.61	2	2	2	1549	165.1721	4.995605	0.95387	0.90574	0.925355	0.9283221	0.0359642	0.084367
Q0VG54	TNS1 protein OS=Homo sapiens GN=TNS1 PE=2 SV=1 - [Q0VG54_HUMAN]	18.62	23	25	37	1713	182.9559	7.825684	0.934352	0.94397	0.906591	0.9283046	0.0235704	0.06674
Q5JNW7	Proteasome subunit beta type-8 OS=Homo sapiens GN=PSMB8 PE=1 SV=1 - [Q5JNW7_HUMAN]	10.32	2	2	4	252	27.83227	7.459473	0.904241	0.901288	0.978633	0.9280542	0.10464	0.1674735
Q96TC7	Regulator of microtubule dynamics protein 3 OS=Homo sapiens GN=RMDN3 PE=1 SV=2 - [RMD3_HUMAN]	10.85	3	3	3	470	52.08604	5.097168	0.864177	0.852627	1.06652	0.9277756	0.407598	0.4797355
O14617	AP-3 complex subunit delta-1 OS=Homo sapiens GN=AP3D1 PE=1 SV=1 - [AP3D1_HUMAN]	7.2	5	5	5	1153	130.0758	8.484863	0.864713	0.860882	1.05654	0.9273784	0.377681	0.4507934
O95219	Sorting nexin-4 OS=Homo sapiens GN=SNX4 PE=1 SV=1 - [SNX4_HUMAN]	7.11	2	2	2	450	51.8761	5.98584	0.944467	0.867893	0.968895	0.9270853	0.138767	0.2039628
P62333	26S protease regulatory subunit 10B OS=Homo sapiens GN=PSMC6 PE=1 SV=1 - [PRS10_HUMAN]	18.25	6	6	8	389	44.1451	7.48877	0.959573	0.910612	0.911012	0.9270655	0.0462475	0.0979349

Q9BQD2	Signal transducer and activator of transcription OS=Homo sapiens PE=2 SV=1 - [Q9BQD2_HUMAN]	6.33	4	4	4	679	76.0777	7.85498	0.974829	0.875494	0.929659	0.9266609	0.125158	0.1903234
A0A0C4DGV7	Retinol-binding protein 4 OS=Homo sapiens GN=RBP4 PE=1 SV=1 - [A0A0C4DGV7_HUMAN]	35.18	6	6	6	199	22.85315	5.719238	0.9519	0.885523	0.942291	0.9265712	0.0711653	0.1280953
B4E1E2	cDNA FLJ61530, highly similar to Hepatocyte growth factor-regulated tyrosine kinase substrate OS=Homo sapiens PE=2 SV=1 - [B4E1E2_HUMAN]	6.05	4	4	4	661	74.80763	7.986816	0.955491	0.904598	0.918686	0.9262582	0.0398186	0.0893806
P00747	Plasminogen OS=Homo sapiens GN=PLG PE=1 SV=2 - [PLMN_HUMAN]	31.6	17	18	28	810	90.51016	7.239746	0.924077	0.907253	0.947278	0.9262027	0.0238395	0.0674158
B4DWS6	cDNA FLJ61181, highly similar to Homo sapiens hydroxysteroid (17-beta) dehydrogenase 12 (HSD17B12), mRNA OS=Homo sapiens PE=2 SV=1 - [B4DWS6_HUMAN]	13.49	3	3	5	304	33.5068	9.393066	0.901852	0.916033	0.959561	0.9258153	0.0506506	0.1028322
O14494	Lipid phosphate phosphohydrolase 1 OS=Homo sapiens GN=PPAP2A PE=1 SV=1 - [LPP1_HUMAN]	10.21	2	2	4	284	32.13545	7.972168	0.934331	0.89866	0.943961	0.9256507	0.0326702	0.0799629
A0A024RAB6	Heparan sulfate proteoglycan 2 (Perlecan), isoform CRA_b OS=Homo sapiens GN=HSPG2 PE=4 SV=1 - [A0A024RAB6_HUMAN]	33.06	112	112	267	4346	463.7229	6.507324	0.923685	0.916303	0.936012	0.9253332	0.00587612	0.0416123
A0A024R442	Aspartyl aminopeptidase, isoform CRA_b OS=Homo sapiens GN=DNPEP PE=3 SV=1 - [A0A024R442_HUMAN]	11.68	4	4	5	471	51.96572	7.415527	0.986468	1.01569	0.773781	0.9253144	0.430553	0.5025593
D9ZGF8	Rho-associated protein kinase OS=Homo sapiens GN=ROCK1 PE=3 SV=1 - [D9ZGF8_HUMAN]	6.5	8	8	8	1354	158.1448	5.935059	0.963556	0.932882	0.877552	0.9246634	0.0958113	0.1573082
Q96DG6	Carboxymethylenebutenolidase homolog OS=Homo sapiens GN=CMBL PE=1 SV=1 - [CMBL_HUMAN]	11.43	3	3	3	245	28.03018	7.181152	0.867312	1.05813	0.846926	0.9241217	0.376401	0.4494241
B4DMR3	cDNA FLJ51896, highly similar to Glia-derived nexin OS=Homo sapiens PE=2 SV=1 - [B4DMR3_HUMAN]	9.58	3	3	3	334	37.13365	9.524902	0.869585	0.978737	0.92266	0.9236606	0.136394	0.2013492

O94919	Endonuclease domain-containing 1 protein OS=Homo sapiens GN=ENDOD1 PE=1 SV=2 - [ENDD1_HUMAN]	12.2	4	4	5	500	54.98127	5.706543	0.871956	0.887347	1.00864	0.9226486	0.215434	0.2849723
Q07954	Prolow-density lipoprotein receptor-related protein 1 OS=Homo sapiens GN=LRP1 PE=1 SV=2 - [LRP1_HUMAN]	6.82	25	25	32	4544	504.2759	5.38916	0.912716	0.904914	0.949138	0.922256	0.0293809	0.0759612
P07360	Complement component C8 gamma chain OS=Homo sapiens GN=C8G PE=1 SV=3 - [CO8G_HUMAN]	40.1	5	5	8	202	22.26354	8.309082	0.961757	0.894706	0.910156	0.9222065	0.0616898	0.1162829
B7Z715	cDNA FLJ59771, moderately similar to Homo sapiens murine retrovirus integration site 1 homolog (MRVI1), transcript variant 2, mRNA OS=Homo sapiens PE=2 SV=1 - [B7Z715_HUMAN]	5.84	2	2	3	428	46.63443	5.808105	1.08022	0.651702	1.03444	0.9221193	0.624328	0.6848186
P02647	Apolipoprotein A-I OS=Homo sapiens GN=APOA1 PE=1 SV=1 - [APOA1_HUMAN]	67.79	23	23	113	267	30.75893	5.757324	0.917833	0.931393	0.916059	0.9217617	0.00380951	0.0387187
Q9BR63	FARSB protein (Fragment) OS=Homo sapiens GN=FARSB PE=2 SV=2 - [Q9BR63_HUMAN]	15.73	7	7	9	585	65.65936	6.844238	0.909101	0.8844	0.971684	0.9217283	0.0947431	0.156137
O95865	N(G),N(G)-dimethylarginine dimethylaminohydrolase 2 OS=Homo sapiens GN=DDAH2 PE=1 SV=1 - [DDAH2_HUMAN]	47.37	9	10	18	285	29.62546	6.01123	0.9214	0.925146	0.918166	0.9215709	0.000660597	0.0241782
Q5T985	Inter-alpha-trypsin inhibitor heavy chain H2 OS=Homo sapiens GN=ITIH2 PE=1 SV=1 - [Q5T985_HUMAN]	19.14	16	16	31	935	105.1501	7.034668	0.872396	0.942632	0.949301	0.9214432	0.0856449	0.1459758
O75955	Flotillin-1 OS=Homo sapiens GN=FLOT1 PE=1 SV=3 - [FLOT1_HUMAN]	20.84	7	7	8	427	47.32562	7.48877	0.954223	0.899993	0.910113	0.9214429	0.0420981	0.0926217
O75923	Dysferlin OS=Homo sapiens GN=DYSF PE=1 SV=1 - [DYSF_HUMAN]	5.63	8	10	10	2080	237.1442	5.643066	0.958501	0.89776	0.90791	0.9213905	0.0526372	0.1054926
Q53HP9	Chromosome 14 open reading frame 159 variant (Fragment) OS=Homo sapiens PE=2 SV=1 - [Q53HP9_HUMAN]	15.74	5	5	5	413	44.20274	6.468262	0.916237	0.830561	1.01712	0.9213051	0.281805	0.3548772
O43866	CD5 antigen-like OS=Homo sapiens GN=CD5L PE=1 SV=1 -	15.85	5	5	7	347	38.06295	5.465332	0.921545	0.889509	0.952168	0.9210739	0.0487232	0.1008751

Biology Open • Supplementary information

	[CD5L_HUMAN]													
O15116	U6 snRNA-associated Sm-like protein LSm1 OS=Homo sapiens GN=LSM1 PE=1 SV=1 - [LSM1_HUMAN]	16.54	2	2	2	133	15.17012	5.224121	0.846658	1.00335	0.912135	0.9207138	0.223097	0.2930475
E5RFP0	NudC domain-containing protein 2 OS=Homo sapiens GN=NUDCD2 PE=1 SV=1 - [E5RFP0_HUMAN]	20.45	2	2	2	132	14.73617	4.678223	0.847741	0.808022	1.10637	0.9207101	0.485869	0.5548808
Q99747	Gamma-soluble NSF attachment protein OS=Homo sapiens GN=NAPG PE=1 SV=1 - [SNAG_HUMAN]	15.71	4	4	6	312	34.72434	5.414551	0.90723	0.955382	0.897756	0.9201224	0.0464369	0.0980592
Q99598	Translin-associated protein X OS=Homo sapiens GN=TSNAX PE=1 SV=1 - [TSNAX_HUMAN]	18.28	4	4	4	290	33.09171	6.55127	0.894499	0.959563	0.905805	0.9199558	0.0575048	0.1120445
Q9Y2U8	Inner nuclear membrane protein Man1 OS=Homo sapiens GN=LEMD3 PE=1 SV=2 - [MAN1_HUMAN]	2.85	2	2	3	911	99.9351	7.547363	0.942744	0.855035	0.959144	0.9189744	0.129012	0.1943505
A0A024R9B9	Matrilin 2, isoform CRA_b OS=Homo sapiens GN=MATN2 PE=4 SV=1 - [A0A024R9B9_HUMAN]	8.79	6	6	6	922	102.806	6.468262	0.908102	0.945373	0.902564	0.9186796	0.0262521	0.0715486
B3KNE7	cDNA FLJ14468 fis, clone MAMMA1000734, highly similar to Translocation protein SEC63 homolog OS=Homo sapiens PE=2 SV=1 - [B3KNE7_HUMAN]	6.22	2	2	2	418	47.8999	8.89502	0.909175	0.83938	1.00682	0.9184587	0.235116	0.3043021
B4DNK3	cDNA FLJ52127, highly similar to Multisynthetase complex auxiliary component p43 OS=Homo sapiens PE=2 SV=1 - [B4DNK3_HUMAN]	11.15	2	2	3	269	29.72676	9.070801	0.853905	0.878972	1.02249	0.9184555	0.260694	0.3317478
P31937	3-hydroxyisobutyrate dehydrogenase, mitochondrial OS=Homo sapiens GN=HIBADH PE=1 SV=2 - [3HIDH_HUMAN]	9.23	2	2	3	336	35.30576	8.133301	0.841182	0.94706	0.966797	0.9183461	0.171335	0.2388111
E5KLK2	Mitochondrial dynamin-like 120 kDa protein OS=Homo sapiens GN=OPA1 PE=4 SV=1 - [E5KLK2_HUMAN]	7.47	6	6	6	924	107.5006	7.986816	0.92257	0.928925	0.900525	0.91734	0.0106645	0.0490917
A8MW49	Fatty acid-binding protein, liver OS=Homo sapiens GN=FABP1 PE=1 SV=1 - [A8MW49_HUMAN]	45.16	4	4	9	124	13.799	5.262207	0.931778	0.90627	0.913876	0.9173078	0.00825704	0.0456887

B4DRF0	cDNA FLJ56418, highly similar to WD repeat protein 26 OS=Homo sapiens PE=2 SV=1 - [B4DRF0_HUMAN]	4.88	2	2	2	451	51.50046	6.214355	0.889113	0.890397	0.971516	0.9170088	0.0930499	0.154213
Q9H8D6	cDNA FLJ13729 fis, clone PLACE3000121, weakly similar to VESICULAR TRAFFIC CONTROL PROTEIN SEC15 OS=Homo sapiens PE=2 SV=1 - [Q9H8D6_HUMAN]	6.9	2	2	2	348	40.16077	7.459473	0.900966	0.917045	0.932435	0.9168152	0.0117188	0.0494101
P02747	Complement C1q subcomponent subunit C OS=Homo sapiens GN=C1QC PE=1 SV=3 - [C1QC_HUMAN]	16.33	3	3	6	245	25.75714	8.411621	0.937547	0.875222	0.935066	0.915945	0.054035	0.1072777
B4DJB3	cDNA FLJ55979, highly similar to Homo sapiens AE binding protein 1 (AEBP1), mRNA OS=Homo sapiens PE=2 SV=1 - [B4DJB3_HUMAN]	17.49	7	7	9	589	66.55874	4.855957	0.910216	0.839548	0.997199	0.9156542	0.205532	0.2749727
Q969G5	Protein kinase C delta-binding protein OS=Homo sapiens GN=PRKCDBP PE=1 SV=3 - [PRDBP_HUMAN]	30.27	9	9	13	261	27.68454	6.430176	0.963852	0.918909	0.862953	0.9152381	0.100923	0.1631444
Q7L1Q6	Basic leucine zipper and W2 domain-containing protein 1 OS=Homo sapiens GN=BZW1 PE=1 SV=1 - [BZW1_HUMAN]	8.11	2	4	5	419	48.01275	5.922363	0.875908	0.950889	0.918606	0.9151344	0.0596709	0.1143971
Q15121	Astrocytic phosphoprotein PEA-15 OS=Homo sapiens GN=PEA15 PE=1 SV=2 - [PEA15_HUMAN]	53.08	5	5	7	130	15.03079	5.020996	0.909038	0.909919	0.925969	0.9149754	0.00416225	0.0391996
B7Z2A1	cDNA FLJ56068, highly similar to DNA damage-binding protein 1 OS=Homo sapiens PE=2 SV=1 - [B7Z2A1_HUMAN]	7.42	7	7	9	1092	121.6598	5.541504	0.931355	0.910059	0.903098	0.9148371	0.00981485	0.0478721
A0A090N8G0	Glycyl-tRNA synthetase OS=Homo sapiens GN=GARS PE=3 SV=1 - [A0A090N8G0_HUMAN]	6.28	4	4	5	685	77.48131	6.239746	0.893106	1.03008	0.820664	0.9146178	0.298895	0.3719645
A2NUT2	Lambda-chain (AA -20 to 215) OS=Homo sapiens PE=1 SV=1 - [A2NUT2_HUMAN]	40.43	2	10	76	235	24.63914	7.620605	0.927714	0.919935	0.896199	0.9146158	0.0120994	0.0498254
Q9UPN3	Microtubule-actin cross-linking factor 1, isoforms 1/2/3/5 OS=Homo sapiens GN=MACF1 PE=1 SV=4 - [MACF1_HUMAN]	1.73	9	11	11	7388	837.7871	5.38916	0.903505	0.85733	0.982283	0.9143729	0.14344	0.2091061

P41226	Ubiquitin-like modifier-activating enzyme 7 OS=Homo sapiens GN=UBA7 PE=1 SV=2 - [UBA7_HUMAN]	4.15	3	3	3	1012	111.6241	6.036621	0.940133	0.870815	0.931324	0.9140906	0.058707	0.1132972
A7E2Y5	DnaJ (Hsp40) homolog, subfamily C, member 13 OS=Homo sapiens GN=DNAJC13 PE=2 SV=1 - [A7E2Y5_HUMAN]	3.74	8	8	9	2243	254.2915	6.756348	0.906147	0.935207	0.900732	0.9140289	0.0151496	0.0543533
P00734	Prothrombin OS=Homo sapiens GN=F2 PE=1 SV=2 - [THRB_HUMAN]	34.08	17	17	33	622	69.99213	5.896973	0.913181	0.929843	0.895794	0.9129396	0.0125098	0.050533
A0A024R1X8	Junction plakoglobin, isoform CRA_a OS=Homo sapiens GN=JUP PE=4 SV=1 - [A0A024R1X8_HUMAN]	14.63	5	7	7	745	81.67478	6.138184	0.901984	0.875031	0.960595	0.9125366	0.0742277	0.1317571
Q13636	Ras-related protein Rab-31 OS=Homo sapiens GN=RAB31 PE=1 SV=1 - [RAB31_HUMAN]	13.92	2	2	2	194	21.55491	7.063965	0.944544	0.925989	0.866231	0.9122546	0.0654644	0.1208371
Q7Z406	Myosin-14 OS=Homo sapiens GN=MYH14 PE=1 SV=2 - [MYH14_HUMAN]	13.83	12	24	42	1995	227.7318	5.60498	0.889121	0.956459	0.890548	0.9120426	0.058256	0.1127482
P24844	Myosin regulatory light polypeptide 9 OS=Homo sapiens GN=MYL9 PE=1 SV=4 - [MYL9_HUMAN]	55.81	4	9	52	172	19.81445	4.919434	0.947902	0.869366	0.918534	0.9119341	0.0615002	0.1161724
E9PCT3	Caveolin OS=Homo sapiens GN=CAV2 PE=1 SV=1 - [E9PCT3_HUMAN]	26.55	2	2	2	113	12.94437	5.059082	0.915429	0.907175	0.913018	0.9118739	0.00077233	0.0242397
B7Z539	cDNA FLJ56954, highly similar to Inter-alpha-trypsin inhibitor heavy chain H1 OS=Homo sapiens PE=2 SV=1 - [B7Z539_HUMAN]	20	10	10	21	645	72.074	7.679199	0.876679	0.921635	0.935742	0.9113522	0.038067	0.0873014
B7Z2F7	cDNA FLJ54655, highly similar to Heat shock 70 kDa protein 12A OS=Homo sapiens PE=2 SV=1 - [B7Z2F7_HUMAN]	13.85	6	6	9	592	66.37448	8.250488	0.936668	0.943647	0.853536	0.9112837	0.0919901	0.1529403
B7Z8A2	cDNA FLJ51671, highly similar to Prenylcysteine oxidase (EC 1.8.3.5) OS=Homo sapiens PE=2 SV=1 - [B7Z8A2_HUMAN]	18.48	7	7	20	487	54.51858	5.731934	0.91251	0.89628	0.924722	0.9111709	0.00849063	0.0456887
Q9NVE7	Pantothenate kinase 4 OS=Homo sapiens GN=PANK4 PE=1 SV=1 - [PANK4_HUMAN]	5.95	3	4	4	773	85.937	6.277832	0.811118	0.89875	1.02176	0.9105431	0.280702	0.3537514

Biology Open • Supplementary information

B2R806	Eukaryotic translation initiation factor 3 subunit E OS=Homo sapiens GN=EIF3E PE=2 SV=1 - [B2R806_HUMAN]	6.29	3	3	3	445	52.22335	6.036621	1.01105	0.899496	0.820353	0.9102997	0.246333	0.3161677
Q9H3N1	Thioredoxin-related transmembrane protein 1 OS=Homo sapiens GN=TMX1 PE=1 SV=1 - [TMX1_HUMAN]	8.93	2	2	3	280	31.7708	4.98291	0.884093	0.957123	0.888257	0.9098243	0.0625558	0.1172488
P55899	IgG receptor FcRn large subunit p51 OS=Homo sapiens GN=FCGRT PE=1 SV=1 - [FCGRN_HUMAN]	10.96	3	3	3	365	39.71828	6.536621	0.840261	0.987602	0.90124	0.909701	0.169009	0.236873
Q9Y4F1	FERM, RhoGEF and pleckstrin domain-containing protein 1 OS=Homo sapiens GN=FARP1 PE=1 SV=1 - [FARP1_HUMAN]	3.16	3	3	4	1045	118.5587	8.147949	0.913948	0.875012	0.939085	0.9093481	0.0397696	0.0893299
B7Z650	cDNA FLJ58685, highly similar to Homo sapiens echinoderm microtubule associated protein like 1 (EML1), transcript variant 1, mRNA OS=Homo sapiens PE=2 SV=1 - [B7Z650_HUMAN]	8.59	6	6	7	803	88.69963	7.95752	0.962383	0.885382	0.877024	0.9082627	0.0776269	0.1360078
P05155	Plasma protease C1 inhibitor OS=Homo sapiens GN=SERPING1 PE=1 SV=2 - [IC1_HUMAN]	24.6	12	12	20	500	55.1194	6.55127	0.888393	0.920458	0.915256	0.9080358	0.0114718	0.0494101
P69849	Nodal modulator 3 OS=Homo sapiens GN=NOMO3 PE=3 SV=2 - [NOMO3_HUMAN]	3.52	3	3	3	1222	134.0493	5.668457	0.923862	0.867648	0.932467	0.9079921	0.0454931	0.0967908
Q96FS1	CTNND1 protein (Fragment) OS=Homo sapiens GN=CTNND1 PE=2 SV=2 - [Q96FS1_HUMAN]	10.84	7	7	13	830	92.33073	7.151855	0.918801	0.877716	0.927102	0.907873	0.0263824	0.0717881
P47985	Cytochrome b-c1 complex subunit Rieske, mitochondrial OS=Homo sapiens GN=UQCRFS1 PE=1 SV=2 - [UCRI_HUMAN]	15.69	3	3	3	274	29.64937	8.32373	0.868133	0.941509	0.912508	0.9073832	0.0491877	0.1015866
Q6NZI2	Polymerase I and transcript release factor OS=Homo sapiens GN=PTRF PE=1 SV=1 - [PTRF_HUMAN]	33.08	15	15	41	390	43.44982	5.60498	0.885966	0.915243	0.92081	0.9073395	0.0133316	0.0516924
Q9P253	Vacuolar protein sorting-associated protein 18 homolog OS=Homo sapiens GN=VPS18 PE=1 SV=2 - [VPS18_HUMAN]	2.57	2	2	2	973	110.1158	6.074707	0.972717	0.845132	0.903991	0.9072798	0.128357	0.1936223

Biology Open • Supplementary information

D3DVQ1	Leucine zipper-EF-hand containing transmembrane protein 1, isoform CRA_a OS=Homo sapiens GN=LETM1 PE=4 SV=1 - [D3DVQ1_HUMAN]	6.98	3	3	3	559	63.50159	5.236816	0.878088	0.902175	0.941269	0.9071771	0.0371557	0.0863586
A8K6I4	cDNA FLJ76877, highly similar to Homo sapiens superkiller viralicidic activity 2-like 2 (SKIV2L2), mRNA OS=Homo sapiens PE=2 SV=1 - [A8K6I4_HUMAN]	2.4	2	2	2	1042	117.8234	6.536621	0.927898	0.918491	0.874546	0.9069783	0.0298503	0.0765061
Q5H924	HECT, UBA and WWE domain containing 1 (Fragment) OS=Homo sapiens GN=HUWE1 PE=4 SV=1 - [Q5H924_HUMAN]	1.29	4	4	4	3407	373.9625	5.097168	0.999021	0.769653	0.949634	0.9061025	0.310258	0.3830136
B4DTB9	cDNA FLJ50666, weakly similar to Long-chain-fatty-acid--CoA ligase (EC 6.2.1.3) OS=Homo sapiens PE=2 SV=1 - [B4DTB9_HUMAN]	7.87	3	3	4	572	63.57744	7.415527	0.865264	0.891516	0.960374	0.9057178	0.0797962	0.1388027
G1UI17	Glycogen debranching enzyme (Fragment) OS=Homo sapiens GN=AGL PE=2 SV=1 - [G1UI17_HUMAN]	4.68	4	4	4	1262	143.6301	6.63916	0.888477	0.839347	0.988325	0.905383	0.163511	0.2310037
B2RDR4	cDNA, FLJ96732, highly similar to Homo sapiens testis derived transcript (3 LIM domains) (TES), transcript variant 1, mRNA OS=Homo sapiens PE=2 SV=1 - [B2RDR4_HUMAN]	30.4	10	10	19	421	47.93388	7.679199	0.928812	0.892753	0.893798	0.9051211	0.0152418	0.0543533
P06727	Apolipoprotein A-IV OS=Homo sapiens GN=APOA4 PE=1 SV=3 - [APOA4_HUMAN]	47.47	18	18	33	396	45.37147	5.376465	0.908441	0.887491	0.919064	0.9049987	0.00939828	0.0472949
Q01118	Sodium channel protein type 7 subunit alpha OS=Homo sapiens GN=SCN7A PE=1 SV=2 - [SCN7A_HUMAN]	1.19	2	2	2	1682	193.3661	7.95752	0.896392	0.858087	0.960437	0.9049719	0.0861438	0.146531
Q0QEN7	ATP synthase subunit beta (Fragment) OS=Homo sapiens GN=ATP5B PE=2 SV=1 - [Q0QEN7_HUMAN]	60.22	18	18	67	445	48.08304	5.071777	0.912713	0.884992	0.916747	0.9048176	0.0108179	0.0492111
Q59HE5	Cysteine-tRNA ligase isoform b variant (Fragment) OS=Homo sapiens PE=2 SV=1 - [Q59HE5_HUMAN]	8.19	2	2	2	281	32.05226	5.541504	0.900099	0.906729	0.907419	0.9047493	0.000599626	0.0241782

Biology Open • Supplementary information

E9PC71	Hippocalcin-like protein 1 OS=Homo sapiens GN=HPCAL1 PE=1 SV=1 - [E9PC71_HUMAN]	9.71	2	2	2	175	20.2588	5.681152	0.904635	0.928378	0.881057	0.90469	0.0199305	0.0616166
Q9H9J2	39S ribosomal protein L44, mitochondrial OS=Homo sapiens GN=MRPL44 PE=1 SV=1 - [RM44_HUMAN]	9.04	2	2	2	332	37.51171	8.396973	0.828996	0.959855	0.923805	0.9042187	0.133543	0.1987002
A0A024R248	Chromosome 9 open reading frame 89, isoform CRA_b OS=Homo sapiens GN=C9orf89 PE=4 SV=1 - [A0A024R248_HUMAN]	13.11	2	2	2	183	20.61261	8.104004	0.891458	0.907595	0.912524	0.903859	0.00434994	0.039476
Q5JQ44	Putative uncharacterized protein DKFZp547A0616 (Fragment) OS=Homo sapiens GN=DKFZp547A0616 PE=2 SV=1 - [Q5JQ44_HUMAN]	30.39	3	5	7	181	20.28107	4.805176	0.920596	0.885124	0.905837	0.9038524	0.0112564	0.0493721
P62263	40S ribosomal protein S14 OS=Homo sapiens GN=RPS14 PE=1 SV=3 - [RS14_HUMAN]	37.75	5	5	9	151	16.26253	10.05225	0.983776	0.808922	0.918006	0.9035683	0.199149	0.2690021
A0A0A0MSB8	Exocyst complex component 7 OS=Homo sapiens GN=EXOC7 PE=1 SV=1 - [A0A0A0MSB8_HUMAN]	9.24	5	5	6	693	78.75968	6.55127	0.982536	0.868885	0.856428	0.9026167	0.135964	0.2010652
A0A024R091	von Willebrand factor A domain containing 1, isoform CRA_c OS=Homo sapiens GN=VWA1 PE=4 SV=1 - [A0A024R091_HUMAN]	19.02	5	5	7	410	43.20934	7.078613	0.881755	0.845558	0.980434	0.9025827	0.136884	0.2018964
B3KMV2	Glucosamine-6-phosphate isomerase OS=Homo sapiens PE=2 SV=1 - [B3KMV2_HUMAN]	16.47	4	4	5	255	28.62262	6.756348	0.946899	0.915904	0.844281	0.9023611	0.0847304	0.1450372
Q6AZE3	CAST protein (Fragment) OS=Homo sapiens GN=CAST PE=2 SV=1 - [Q6AZE3_HUMAN]	19.59	9	9	15	592	63.89195	4.944824	0.857441	0.900635	0.948847	0.9023079	0.0658917	0.121279
Q9H2M9	Rab3 GTPase-activating protein non-catalytic subunit OS=Homo sapiens GN=RAB3GAP2 PE=1 SV=1 - [RBGPR_HUMAN]	2.08	2	2	2	1393	155.8858	5.617676	0.959684	0.921253	0.825957	0.9022979	0.133232	0.1985867
D6RF35	Vitamin D-binding protein OS=Homo sapiens GN=GC PE=1 SV=1 - [D6RF35_HUMAN]	53.57	25	25	47	476	52.98592	5.516113	0.913771	0.910056	0.881499	0.9017752	0.0106016	0.0490917

P01024	Complement C3 OS=Homo sapiens GN=C3 PE=1 SV=2 - [CO3_HUMAN]	52.56	81	81	220	1663	187.0299	6.404785	0.907334	0.912932	0.884043	0.9014365	0.00795816	0.0454273
Q9UEH5	24-kDa subunit of complex I (Fragment) OS=Homo sapiens GN=NDUFV2 PE=4 SV=1 - [Q9UEH5_HUMAN]	10.39	2	2	5	231	25.41398	7.327637	0.916727	0.88344	0.903529	0.9012318	0.00946468	0.0472949
F8VV64	Tensin-2 OS=Homo sapiens GN=TNS2 PE=1 SV=1 - [F8VV64_HUMAN]	6.4	5	7	10	1344	145.8855	8.265137	0.889873	0.944927	0.867619	0.9008065	0.0496904	0.1018392
B4E184	cDNA FLJ53267, highly similar to Optineurin OS=Homo sapiens PE=2 SV=1 - [B4E184_HUMAN]	4.62	2	2	2	520	59.57624	5.274902	0.879934	0.904751	0.916551	0.9004119	0.0115382	0.0494101
Q53G79	Carnitine O-palmitoyltransferase II, mitochondrial variant (Fragment) OS=Homo sapiens PE=2 SV=1 - [Q53G79_HUMAN]	3.99	2	2	2	577	64.68361	7.415527	0.86968	0.94554	0.885108	0.9001093	0.0497281	0.1018392
B4E2V5	cDNA FLJ52062, highly similar to Erythrocyte band 7 integral membrane protein OS=Homo sapiens PE=2 SV=1 - [B4E2V5_HUMAN]	47.26	8	8	19	237	25.94779	7.38623	0.925753	0.861136	0.912507	0.8997984	0.0365679	0.0854261
B2RA94	cDNA, FLJ94779 OS=Homo sapiens PE=2 SV=1 - [B2RA94_HUMAN]	14.18	5	5	7	402	45.70546	8.660645	0.877331	0.946081	0.873183	0.8988649	0.0505251	0.102771
B7Z1N6	Fructose-bisphosphate aldolase OS=Homo sapiens PE=2 SV=1 - [B7Z1N6_HUMAN]	25.85	5	7	13	325	35.40112	7.063965	0.982505	0.783646	0.929561	0.8985706	0.230157	0.2995315
B7Z2R3	cDNA FLJ59510, highly similar to Homo sapiens tight junction protein 2 (TJP2), transcript variant 2, mRNA OS=Homo sapiens PE=2 SV=1 - [B7Z2R3_HUMAN]	4.02	3	3	4	1020	115.1118	6.712402	0.961695	0.857254	0.874882	0.8979439	0.0871584	0.147625
O75531	Barrier-to-autointegration factor OS=Homo sapiens GN=BANF1 PE=1 SV=1 - [BAF_HUMAN]	40.45	2	2	4	89	10.05202	6.087402	0.937649	0.969498	0.785963	0.8977033	0.212557	0.2822697
Q8TCE5	GBP2 protein OS=Homo sapiens GN=GBP2 PE=2 SV=1 - [Q8TCE5_HUMAN]	4.99	2	2	3	481	54.0006	5.820801	0.87841	0.987261	0.827218	0.8976297	0.162285	0.229559

Biology Open • Supplementary information

B2RCP1	cDNA, FLJ96185, highly similar to Homo sapiens guanylate cyclase 1, soluble, beta 3 (GUCY1B3), mRNA OS=Homo sapiens PE=2 SV=1 - [B2RCP1_HUMAN]	5.82	3	3	3	619	70.44152	5.351074	0.952474	0.852601	0.887373	0.8974829	0.0727387	0.1297947
P01008	Antithrombin-III OS=Homo sapiens GN=SERPINC1 PE=1 SV=1 - [ANT3_HUMAN]	39.22	19	19	39	464	52.56887	6.712402	0.89544	0.931591	0.865243	0.8974247	0.0332253	0.0806036
Q9UDQ3	Calcium channel; match to P54289 (PID:g1705852) (Fragment) OS=Homo sapiens GN=WUGSC:H_DJ0560O14.1 PE=4 SV=1 - [Q9UDQ3_HUMAN]	5.64	3	3	3	745	84.34288	5.312988	0.842382	0.677962	1.17169	0.8973457	0.552732	0.6178989
Q59FM5	Guanine nucleotide-binding protein G, alpha subunit variant (Fragment) OS=Homo sapiens PE=2 SV=1 - [Q59FM5_HUMAN]	15.41	2	5	11	357	41.86443	5.693848	0.850824	0.877802	0.962905	0.8971768	0.0930745	0.154213
P12273	Prolactin-inducible protein OS=Homo sapiens GN=PIP PE=1 SV=1 - [PIP_HUMAN]	18.49	2	2	2	146	16.5618	8.04541	0.951922	0.808012	0.931003	0.8969791	0.14867	0.2148796
B4DUL5	cDNA FLJ51625, highly similar to Ubiquinol-cytochrome-c reductase complex coreprotein I, mitochondrial (EC 1.10.2.2) OS=Homo sapiens PE=2 SV=1 - [B4DUL5_HUMAN]	22.47	5	5	9	365	40.3471	5.884277	0.903603	0.853131	0.93413	0.8969547	0.048724	0.1008751
P36269	Gamma-glutamyltransferase 5 OS=Homo sapiens GN=GGT5 PE=1 SV=2 - [GGT5_HUMAN]	18.6	9	9	15	586	62.22232	7.547363	0.913132	0.875477	0.90196	0.8968564	0.0115166	0.0494101
P08754	Guanine nucleotide-binding protein G(k) subunit alpha OS=Homo sapiens GN=GNAI3 PE=1 SV=3 - [GNAI3_HUMAN]	25.14	3	8	19	354	40.50627	5.693848	0.981779	0.893799	0.813292	0.89629	0.166717	0.2347492
Q16610	Extracellular matrix protein 1 OS=Homo sapiens GN=ECM1 PE=1 SV=2 - [ECM1_HUMAN]	16.11	7	7	7	540	60.63528	6.712402	0.822097	0.77486	1.09159	0.8961806	0.403	0.4753153
H7C131	3-ketoacyl-CoA thiolase, peroxisomal (Fragment) OS=Homo sapiens GN=ACAA1 PE=1 SV=1 - [H7C131_HUMAN]	12.41	2	3	3	290	30.31385	8.631348	0.922499	1.0032	0.762612	0.896105	0.279444	0.3522972

Biology Open • Supplementary information

F8VXI9	ARF GTPase-activating protein GIT2 OS=Homo sapiens GN=GIT2 PE=1 SV=1 - [F8VXI9_HUMAN]	3.53	2	2	2	708	78.81836	7.459473	0.817749	0.795525	1.07136	0.894877	0.356727	0.4300288
B4DZ20	cDNA FLJ56422, highly similar to Engulfment and cell motility protein 2 OS=Homo sapiens PE=2 SV=1 - [B4DZ20_HUMAN]	13.27	3	3	3	452	52.57063	6.239746	0.878524	0.896313	0.909095	0.8946442	0.00700492	0.0438293
P02766	Transthyretin OS=Homo sapiens GN=TTR PE=1 SV=1 - [TTHY_HUMAN]	49.66	6	6	23	147	15.87705	5.757324	0.857417	0.921357	0.904692	0.8944888	0.0313984	0.0782718
Q9UL88	Myosin-reactive immunoglobulin heavy chain variable region (Fragment) OS=Homo sapiens PE=2 SV=1 - [Q9UL88_HUMAN]	29.01	2	3	9	131	14.13325	9.627441	0.903748	0.907349	0.872318	0.8944716	0.0109327	0.0493157
B7Z1F8	cDNA FLJ53025, highly similar to Complement C4-B OS=Homo sapiens PE=2 SV=1 - [B7Z1F8_HUMAN]	32.22	2	10	21	270	30.42477	7.986816	0.873733	0.891326	0.918335	0.8944649	0.0147715	0.053881
P84095	Rho-related GTP-binding protein RhoG OS=Homo sapiens GN=RHOG PE=1 SV=1 - [RHOG_HUMAN]	36.65	5	6	10	191	21.29493	8.118652	0.891388	0.882468	0.908685	0.8941804	0.00524731	0.0401071
P28074	Proteasome subunit beta type-5 OS=Homo sapiens GN=PSMB5 PE=1 SV=3 - [PSB5_HUMAN]	14.45	3	3	4	263	28.46221	6.91748	0.869346	0.893111	0.91938	0.8939457	0.0180617	0.0588439
P20339	Ras-related protein Rab-5A OS=Homo sapiens GN=RAB5A PE=1 SV=2 - [RAB5A_HUMAN]	33.49	2	5	7	215	23.64382	8.147949	0.93348	0.842483	0.905041	0.8936679	0.0583537	0.1128727
P00742	Coagulation factor X OS=Homo sapiens GN=F10 PE=1 SV=2 - [FA10_HUMAN]	5.33	2	2	2	488	54.69652	5.935059	0.873961	0.893337	0.913103	0.8934668	0.0110634	0.0493307
Q8ND71	GTPase IMAP family member 8 OS=Homo sapiens GN=GIMAP8 PE=2 SV=2 - [GIMA8_HUMAN]	5.71	4	4	4	665	74.84341	8.338379	0.88856	0.930645	0.860834	0.8933464	0.0343521	0.0817258
Q5JR95	40S ribosomal protein S8 OS=Homo sapiens GN=RPS8 PE=1 SV=1 - [Q5JR95_HUMAN]	20.74	3	3	3	188	21.86602	10.35986	0.879924	0.91528	0.883603	0.8929356	0.0108094	0.0492111
P07099	Epoxide hydrolase 1 OS=Homo sapiens GN=EPHX1 PE=1 SV=1 - [HYEP_HUMAN]	21.76	8	8	9	455	52.91497	7.254395	0.885634	0.883924	0.909206	0.8929211	0.00575337	0.0411737

Biology Open • Supplementary information

B2RB06	cDNA, FLJ95242, highly similar to Homo sapiens L-3-hydroxyacyl-Coenzyme A dehydrogenase, short chain (HADHSC), mRNA OS=Homo sapiens PE=2 SV=1 - [B2RB06_HUMAN]	15.61	5	5	11	314	34.23988	8.851074	0.807663	0.965291	0.90494	0.8926314	0.144326	0.2103072
Q13425	Beta-2-syntrophin OS=Homo sapiens GN=SNTB2 PE=1 SV=1 - [SNTB2_HUMAN]	10.37	4	6	8	540	57.91303	8.821777	0.894344	0.873928	0.90819	0.892154	0.0084066	0.0456887
P01011	Alpha-1-antichymotrypsin OS=Homo sapiens GN=SERPINA3 PE=1 SV=2 - [AACT_HUMAN]	34.52	15	15	57	423	47.62054	5.516113	0.88779	0.894684	0.893295	0.8919233	0.00037914	0.0212234
Q5QPQ1	Acyl-protein thioesterase 2 (Fragment) OS=Homo sapiens GN=LYPLA2 PE=1 SV=1 - [Q5QPQ1_HUMAN]	16.05	2	2	2	162	17.50505	8.118652	1.0026	0.797112	0.876014	0.8919096	0.212666	0.2823037
Q5TB53	Transmembrane 9 superfamily member 3 (Fragment) OS=Homo sapiens GN=TM9SF3 PE=1 SV=6 - [Q5TB53_HUMAN]	7.51	2	2	3	253	29.8617	5.820801	0.993647	0.829965	0.849046	0.8908862	0.169135	0.236873
O95336	6-phosphogluconolactonase OS=Homo sapiens GN=PGLS PE=1 SV=2 - [6PGL_HUMAN]	33.33	7	7	10	258	27.52955	6.049316	0.862018	0.845649	0.964842	0.8908363	0.0996273	0.1619003
P27487	Dipeptidyl peptidase 4 OS=Homo sapiens GN=DPP4 PE=1 SV=2 - [DPP4_HUMAN]	4.05	3	3	3	766	88.22249	6.036621	0.923945	0.915183	0.831662	0.8902635	0.0649106	0.1201788
B4DR71	cDNA FLJ57078, highly similar to Homo sapiens opioid receptor, sigma 1 (OPRS1), transcript variant 1, mRNA OS=Homo sapiens PE=2 SV=1 - [B4DR71_HUMAN]	12.26	2	2	2	155	17.48598	6.785645	0.818572	0.964394	0.887523	0.8901631	0.120929	0.1855597
P04040	Catalase OS=Homo sapiens GN=CAT PE=1 SV=3 - [CATA_HUMAN]	34.54	16	16	34	527	59.71876	7.38623	0.884008	0.90189	0.883815	0.8899043	0.00294996	0.0360523
Q03135	Caveolin-1 OS=Homo sapiens GN=CAV1 PE=1 SV=4 - [CAV1_HUMAN]	39.33	6	6	16	178	20.45848	6.023926	0.843675	0.935597	0.890054	0.8897753	0.0533607	0.1066179
A7MD96	SYNPO protein (Fragment) OS=Homo sapiens GN=SYNPO PE=2 SV=1 - [A7MD96_HUMAN]	13.53	7	7	8	887	94.65309	9.744629	0.898801	0.911141	0.858671	0.8895376	0.0199478	0.0616166

Biology Open • Supplementary information

Q9NTX5	Ethylmalonyl-CoA decarboxylase OS=Homo sapiens GN=ECHDC1 PE=1 SV=2 - [ECHD1_HUMAN]	9.45	3	3	4	307	33.67653	8.206543	0.926905	0.845751	0.894763	0.8891397	0.0424376	0.0931591
B2R514	cDNA, FLJ92300, Homo sapiens COP9 subunit 6 (MOV34 homolog, 34 kD) (COPS6), mRNA OS=Homo sapiens PE=2 SV=1 - [B2R514_HUMAN]	18.18	4	4	4	297	33.55399	5.998535	0.889497	0.840059	0.93718	0.8889116	0.0581972	0.1126988
I3L1P8	Mitochondrial 2-oxoglutarate/malate carrier protein (Fragment) OS=Homo sapiens GN=SLC25A11 PE=1 SV=1 - [I3L1P8_HUMAN]	8.11	2	2	2	296	32.16176	9.773926	0.875411	0.909433	0.881606	0.8888167	0.00873877	0.0461478
B3KT14	Sulfotransferase OS=Homo sapiens PE=2 SV=1 - [B3KT14_HUMAN]	12.88	2	3	4	295	34.19012	6.01123	0.914878	0.903053	0.848245	0.8887252	0.0323823	0.0797193
J3QSB7	S-methyl-5'-thioadenosine phosphorylase OS=Homo sapiens GN=MTAP PE=1 SV=1 - [J3QSB7_HUMAN]	25.62	4	4	5	242	26.75922	6.844238	0.889122	0.844458	0.931621	0.8884003	0.0472692	0.0991979
A8K9A9	cDNA FLJ77744, highly similar to Homo sapiens kallikrein B, plasma (Fletcher factor) 1 (KLKB1), mRNA OS=Homo sapiens PE=2 SV=1 - [A8K9A9_HUMAN]	4.08	2	2	2	638	71.30681	8.221191	0.860297	0.928361	0.876186	0.8882817	0.032234	0.079702
B4E1C4	cDNA FLJ51179, highly similar to Vitamin K-dependent protein C (EC 3.4.21.69) OS=Homo sapiens PE=2 SV=1 - [B4E1C4_HUMAN]	6.41	2	2	2	421	46.0296	7.928223	0.91645	0.833274	0.914433	0.8880525	0.0549921	0.1085413
P23946	Chymase OS=Homo sapiens GN=CMA1 PE=1 SV=1 - [CMA1_HUMAN]	38.87	8	8	17	247	27.30719	9.290527	0.926325	0.899109	0.838552	0.8879953	0.0496757	0.1018392
P21266	Glutathione S-transferase Mu 3 OS=Homo sapiens GN=GSTM3 PE=1 SV=3 - [GSTM3_HUMAN]	32	6	7	14	225	26.54214	5.541504	0.857898	0.938035	0.868028	0.887987	0.0470484	0.0989185
B4DM97	cDNA FLJ55002, highly similar to Alpha-centractin OS=Homo sapiens PE=2 SV=1 - [B4DM97_HUMAN]	23.65	3	6	10	334	38.25072	6.961426	0.92186	0.896457	0.844613	0.8876432	0.0385748	0.0877525
A8K7H3	cDNA FLJ77670, highly similar to Homo sapiens ribosomal protein S15a (RPS15A), mRNA OS=Homo sapiens PE=2	46.15	2	5	8	130	14.81394	10.12549	0.82061	0.910027	0.930929	0.8871882	0.0793766	0.1382149

Biology Open • Supplementary information

	SV=1 - [A8K7H3_HUMAN]														
Q9UBV8	Peflin OS=Homo sapiens GN=PEF1 PE=1 SV=1 - [PEF1_HUMAN]	7.39	2	2	2	284	30.36131	6.536621	0.860888	0.831697	0.967726	0.8867705	0.111477	0.1745373
P24752	Acetyl-CoA acetyltransferase, mitochondrial OS=Homo sapiens GN=ACAT1 PE=1 SV=1 - [THIL_HUMAN]	25.06	8	8	11	427	45.17065	8.851074	0.896654	0.866164	0.897009	0.886609	0.00803034	0.0455523
P08572	Collagen alpha-2(IV) chain OS=Homo sapiens GN=COL4A2 PE=1 SV=4 - [CO4A2_HUMAN]	10.4	12	12	47	1712	167.4486	8.660645	0.890801	0.855445	0.913014	0.88642	0.0210935	0.063152
Q49AE1	CLIC5 protein OS=Homo sapiens GN=CLIC5 PE=2 SV=1 - [Q49AE1_HUMAN]	25	2	2	3	72	8.015204	5.452637	0.899125	1.00519	0.754525	0.8862809	0.257973	0.3285322
Q9UBQ7	Glyoxylate reductase/hydroxypyruvate reductase OS=Homo sapiens GN=GRHPR PE=1 SV=1 - [GRHPR_HUMAN]	26.22	6	6	10	328	35.64574	7.38623	0.926013	0.846395	0.884649	0.8856857	0.038146	0.0874233
A8MPZ0	cDNA FLJ53828, highly similar to Guanine nucleotide-binding protein-like 1 OS=Homo sapiens PE=2 SV=3 - [A8MPZ0_HUMAN]	17.56	3	3	4	262	28.68698	4.690918	0.971524	0.911851	0.771947	0.8851075	0.191548	0.2607117
A0A087WUX8	MICOS complex subunit MIC27 OS=Homo sapiens GN=APOOL PE=1 SV=1 - [A0A087WUX8_HUMAN]	15.21	3	3	3	263	28.74305	9.598145	1.01341	0.797433	0.842208	0.8843511	0.220953	0.2907955
B4DZ36	cDNA FLJ58441, highly similar to Attractin OS=Homo sapiens PE=2 SV=1 - [B4DZ36_HUMAN]	2.16	2	2	2	1156	129.7484	6.873535	0.906713	0.802402	0.942732	0.8839487	0.110166	0.173163
B4DQI1	Complement C2 OS=Homo sapiens GN=C2 PE=1 SV=1 - [B4DQI1_HUMAN]	12.65	2	5	6	506	56.43045	7.840332	0.945254	0.802984	0.90312	0.883786	0.110405	0.1733192
A0A087WZZ5	Splicing factor 3B subunit 2 OS=Homo sapiens GN=SF3B2 PE=1 SV=1 - [A0A087WZZ5_HUMAN]	7.46	5	5	5	871	97.52467	5.668457	0.889876	0.777034	0.98396	0.8836236	0.191117	0.2602297
P63151	Serine/threonine-protein phosphatase 2A 55 kDa regulatory subunit B alpha isoform OS=Homo sapiens GN=PPP2R2A PE=1 SV=1 - [2ABA_HUMAN]	4.92	2	2	2	447	51.65949	6.20166	0.892938	0.843681	0.913384	0.8833344	0.030031	0.0767182

Biology Open • Supplementary information

Q9GZN8	UPF0687 protein C20orf27 OS=Homo sapiens GN=C20orf27 PE=1 SV=3 - [CT027_HUMAN]	20.11	2	2	2	174	19.27875	6.844238	0.890751	0.843725	0.915272	0.8832495	0.0308395	0.0773766
A8K607	cDNA FLJ76855, highly similar to Homo sapiens exportin 7 (XPO7), mRNA OS=Homo sapiens PE=2 SV=1 - [A8K607_HUMAN]	4.32	3	3	3	1087	123.847	6.341309	0.88051	0.904494	0.863953	0.8829859	0.0099642	0.0482782
B7Z8T9	cDNA FLJ52526, highly similar to Lysosomal acid phosphatase (EC 3.1.3.2) OS=Homo sapiens PE=2 SV=1 - [B7Z8T9_HUMAN]	8.05	2	2	2	236	26.55745	5.376465	0.911997	0.859774	0.876224	0.8826652	0.0168272	0.0569966
B4DLT1	cDNA FLJ59716, highly similar to Vacuolar protein sorting 26A OS=Homo sapiens PE=2 SV=1 - [B4DLT1_HUMAN]	18.39	4	4	7	310	36.54783	6.888184	0.881801	0.857668	0.906459	0.8819761	0.0139447	0.0526817
B2R7R5	cDNA, FLJ93570, highly similar to Homo sapiens phosphoribosyl pyrophosphate synthetase-associated protein 2 (PRPSAP2), mRNA OS=Homo sapiens PE=2 SV=1 - [B2R7R5_HUMAN]	11.65	2	3	4	369	40.85731	7.444824	0.742727	0.893424	1.0088	0.8816504	0.264259	0.3352761
E5RIR1	Protein NDRG1 (Fragment) OS=Homo sapiens GN=NDRG1 PE=1 SV=1 - [E5RIR1_HUMAN]	20.25	2	2	5	158	17.43739	4.703613	0.916059	0.838575	0.889512	0.8813822	0.0348252	0.0823753
Q53F35	Acidic (Leucine-rich) nuclear phosphoprotein 32 family, member B variant (Fragment) OS=Homo sapiens PE=2 SV=1 - [Q53F35_HUMAN]	28.4	2	8	11	250	28.65528	4.056152	0.798369	0.945485	0.899141	0.8809981	0.111362	0.1744379
P27694	Replication protein A 70 kDa DNA-binding subunit OS=Homo sapiens GN=RPA1 PE=1 SV=2 - [RFA1_HUMAN]	7.63	4	4	4	616	68.09527	7.210449	0.882764	0.841578	0.917879	0.8807403	0.0325237	0.0798294
B4DVC8	cDNA FLJ56250 OS=Homo sapiens PE=2 SV=1 - [B4DVC8_HUMAN]	8.86	3	3	4	542	58.78518	5.452637	0.913598	0.83347	0.894358	0.8804751	0.0384836	0.0877472
P21281	V-type proton ATPase subunit B, brain isoform OS=Homo sapiens GN=ATP6V1B2 PE=1 SV=3 - [VATB2_HUMAN]	15.07	6	6	6	511	56.46488	5.808105	0.920943	0.828835	0.890441	0.880073	0.0474247	0.0993395

Biology Open • Supplementary information

Q9BYX7	Putative beta-actin-like protein 3 OS=Homo sapiens GN=POTEKP PE=5 SV=1 - [ACTBM_HUMAN]	19.73	2	5	69	375	41.98884	6.328613	0.893639	0.862073	0.883871	0.8798612	0.00597718	0.0419659
B7ZKY2	Calcium/calmodulin-dependent serine protein kinase (MAGUK family) OS=Homo sapiens GN=CASK PE=2 SV=1 - [B7ZKY2_HUMAN]	3.68	3	3	3	897	102.0187	6.404785	0.981877	0.791964	0.864159	0.8793335	0.161041	0.2283719
B3KTJ9	cDNA FLJ38393 fis, clone FEBRA2007212 OS=Homo sapiens PE=2 SV=1 - [B3KTJ9_HUMAN]	6.18	4	4	5	923	102.8658	5.224121	0.860924	0.897415	0.878376	0.8789052	0.00748693	0.0446031
B3KSS1	cDNA FLJ36845 fis, clone ASTRO2013050, weakly similar to UBIQUITIN-ACTIVATING ENZYME E1 OS=Homo sapiens PE=2 SV=1 - [B3KSS1_HUMAN]	3.62	2	2	2	800	90.01228	6.507324	0.919583	0.841778	0.875076	0.8788124	0.0328901	0.0802262
A2A2M0	Regulation of nuclear pre-mRNA domain-containing protein 1B (Fragment) OS=Homo sapiens GN=RPRD1B PE=1 SV=1 - [A2A2M0_HUMAN]	16.24	2	2	2	197	22.11438	4.932129	0.899356	0.848664	0.888096	0.8787057	0.0156771	0.055022
P01619	Ig kappa chain V-III region B6 OS=Homo sapiens PE=1 SV=1 - [KV301_HUMAN]	16.67	2	2	9	108	11.62777	9.246582	0.833273	0.809034	0.993721	0.8786763	0.171326	0.2388111
P04899	Guanine nucleotide-binding protein G(i) subunit alpha-2 OS=Homo sapiens GN=GNAI2 PE=1 SV=3 - [GNAI2_HUMAN]	46.48	9	14	30	355	40.42506	5.541504	0.904512	0.91197	0.819248	0.8785767	0.0550879	0.1086526
Q8NC56	LEM domain-containing protein 2 OS=Homo sapiens GN=LEMD2 PE=1 SV=1 - [LEMD2_HUMAN]	11.73	6	6	7	503	56.93953	8.997559	0.867951	0.844401	0.923287	0.8785461	0.0351176	0.0828962
E9PR44	Alpha-crystallin B chain (Fragment) OS=Homo sapiens GN=CRYAB PE=1 SV=1 - [E9PR44_HUMAN]	38.51	8	8	10	174	20.01833	7.034668	0.894109	0.829035	0.912221	0.8784551	0.0405714	0.0906037
Q16822	Phosphoenolpyruvate carboxykinase [GTP], mitochondrial OS=Homo sapiens GN=PCK2 PE=1 SV=3 - [PCKGM_HUMAN]	11.56	6	6	7	640	70.68465	7.620605	0.911395	0.849353	0.872959	0.8779023	0.0212306	0.0632079
B4DX01	cDNA FLJ59092 OS=Homo sapiens PE=2 SV=1 -	18.25	4	4	6	274	30.5013	5.947754	0.850642	0.923566	0.858951	0.8777195	0.0337395	0.0810562

Biology Open • Supplementary information

	[B4DX01_HUMAN]													
B3KSI3	Branched-chain-amino-acid aminotransferase OS=Homo sapiens GN=BCAT2 PE=1 SV=1 - [B3KSI3_HUMAN]	11.65	3	3	4	352	39.88973	8.23584	0.803099	0.970205	0.858511	0.8772719	0.129834	0.1947222
B3KS18	cDNA FLJ35285 fis, clone PROST2008079, highly similar to Golgi phosphoprotein 3 OS=Homo sapiens PE=2 SV=1 - [B3KS18_HUMAN]	11.86	2	2	2	236	27.12635	7.38623	0.841299	0.919263	0.871229	0.8772639	0.0325668	0.0798294
B7Z5Q2	cDNA FLJ58075, highly similar to Ceruloplasmin (EC 1.16.3.1) OS=Homo sapiens PE=2 SV=1 - [B7Z5Q2_HUMAN]	25.48	22	22	49	946	108.7534	5.77002	0.879398	0.904919	0.846477	0.8769317	0.0183736	0.0594602
E9PIB2	Gasdermin-D (Fragment) OS=Homo sapiens GN=GSDMD PE=1 SV=1 - [E9PIB2_HUMAN]	5.05	2	2	2	277	30.80573	9.041504	0.903085	0.833225	0.894188	0.8768324	0.0303345	0.0772047
F5H7S7	Ras GTPase-activating-like protein IQGAP2 OS=Homo sapiens GN=IQGAP2 PE=1 SV=2 - [F5H7S7_HUMAN]	7.74	8	10	11	1525	174.3405	5.693848	0.920665	0.844172	0.865145	0.8766604	0.0325685	0.0798294
B7Z9B7	cDNA FLJ54732, moderately similar to Sorbin and SH3 domain-containing protein 1 OS=Homo sapiens PE=2 SV=1 - [B7Z9B7_HUMAN]	22.21	4	12	17	860	93.2321	8.558105	0.919063	0.813602	0.89432	0.8756615	0.0597612	0.1144184
P80748	Ig lambda chain V-III region LOI OS=Homo sapiens PE=1 SV=1 - [LV302_HUMAN]	37.84	2	3	6	111	11.92782	5.084473	0.837676	0.863656	0.924195	0.8751757	0.0396721	0.08917
B4DSE2	cDNA FLJ57277, highly similar to Tripeptidyl-peptidase 1 (EC 3.4.14.9) OS=Homo sapiens PE=2 SV=1 - [B4DSE2_HUMAN]	22.86	6	6	16	385	41.64952	5.452637	0.828371	0.915885	0.880635	0.8749637	0.0389392	0.088344
P36957	Dihydrolipoyllysine-residue succinyltransferase component of 2-oxoglutarate dehydrogenase complex, mitochondrial OS=Homo sapiens GN=DLST PE=1 SV=4 - [ODO2_HUMAN]	19.21	7	7	8	453	48.72446	8.953613	0.898967	0.803065	0.922826	0.8749525	0.0760177	0.1341442
B4DF70	cDNA FLJ60461, highly similar to Peroxiredoxin-2 (EC 1.11.1.15) OS=Homo sapiens PE=2 SV=1 - [B4DF70_HUMAN]	45.9	10	11	30	183	20.09462	8.777832	0.86844	0.885977	0.86786	0.8740923	0.00222188	0.033949

Biology Open • Supplementary information

A8MT40	Pyruvate dehydrogenase phosphatase regulatory subunit, mitochondrial OS=Homo sapiens GN=PDPR PE=1 SV=2 - [A8MT40_HUMAN]	5.26	3	3	3	779	88.51308	5.808105	0.900683	0.914643	0.80551	0.873612	0.0663599	0.1218099
A0A0A0MSA0	Laminin subunit alpha-3 OS=Homo sapiens GN=LAMA3 PE=1 SV=1 - [A0A0A0MSA0_HUMAN]	2.29	4	6	6	3277	359.9508	7.239746	0.897388	0.82987	0.891178	0.8728116	0.0275171	0.0734628
P14735	Insulin-degrading enzyme OS=Homo sapiens GN=IDE PE=1 SV=4 - [IDE_HUMAN]	3.24	3	3	3	1019	117.8933	6.609863	0.942293	0.882042	0.792677	0.8723375	0.0989922	0.1611003
A8K964	cDNA FLJ75071, highly similar to Homo sapiens pinin, desmosome associated protein (PNN), mRNA OS=Homo sapiens PE=2 SV=1 - [A8K964_HUMAN]	3.63	2	2	2	717	81.49295	7.371582	0.940427	0.860398	0.815943	0.872256	0.0725533	0.1297549
P55268	Laminin subunit beta-2 OS=Homo sapiens GN=LAMB2 PE=1 SV=2 - [LAMB2_HUMAN]	30.42	43	43	80	1798	195.8544	6.521973	0.850515	0.866034	0.898521	0.8716901	0.0119335	0.0495204
Q9BTT5	Similar to NADH dehydrogenase (Ubiquinone) 1 alpha subcomplex, 9 (39kD) (Fragment) OS=Homo sapiens PE=2 SV=1 - [Q9BTT5_HUMAN]	21.89	5	5	8	338	38.41749	9.671387	0.845446	0.866619	0.901968	0.8713443	0.0160275	0.0555872
A0A024R872	Chromosome 9 open reading frame 88, isoform CRA_a OS=Homo sapiens GN=C9orf88 PE=4 SV=1 - [A0A024R872_HUMAN]	5.46	3	3	5	733	82.63093	6.150879	0.992114	0.799258	0.819411	0.8702614	0.168135	0.2361564
P23193	Transcription elongation factor A protein 1 OS=Homo sapiens GN=TCEA1 PE=1 SV=2 - [TCEA1_HUMAN]	8.31	2	2	2	301	33.94803	8.382324	0.932327	0.837985	0.840448	0.8702533	0.0527615	0.1056166
P61020	Ras-related protein Rab-5B OS=Homo sapiens GN=RAB5B PE=1 SV=1 - [RAB5B_HUMAN]	32.09	3	5	8	215	23.6919	8.133301	0.898652	0.805604	0.9045	0.8695854	0.055375	0.108916
E5RGS4	Prefoldin subunit 1 OS=Homo sapiens GN=PFDN1 PE=1 SV=1 - [E5RGS4_HUMAN]	17.95	2	2	2	117	13.45711	5.516113	0.875276	0.871634	0.861826	0.8695789	0.000947057	0.0260633
A0A087WYJ9	Ig mu chain C region OS=Homo sapiens GN=IGHM PE=1 SV=1 - [A0A087WYJ9_HUMAN]	37.4	4	20	75	599	65.65961	6.946777	0.879091	0.897433	0.829408	0.8686441	0.0231041	0.0660535

Biology Open • Supplementary information

P00738	Haptoglobin OS=Homo sapiens GN=HP PE=1 SV=1 - [HPT_HUMAN]	53.2	12	23	107	406	45.17657	6.580566	0.860516	0.870398	0.869639	0.8668508	0.000568175	0.0241782
E9PIE4	Mitochondrial carrier homolog 2 (Fragment) OS=Homo sapiens GN=MTCH2 PE=1 SV=6 - [E9PIE4_HUMAN]	20.23	3	3	4	262	28.53054	7.605957	0.84466	0.75398	1.00117	0.8666044	0.205913	0.2750653
C9JJT5	Protein ATP5J2-PTCD1 OS=Homo sapiens GN=ATP5J2-PTCD1 PE=4 SV=2 - [C9JJT5_HUMAN]	44.44	2	2	3	54	5.911132	9.319824	0.934707	0.862407	0.801837	0.8663171	0.0735473	0.1307551
P00352	Retinal dehydrogenase 1 OS=Homo sapiens GN=ALDH1A1 PE=1 SV=2 - [AL1A1_HUMAN]	38.32	14	15	32	501	54.82695	6.727051	0.883388	0.845788	0.869633	0.8662698	0.00667822	0.0433606
Q92538	Golgi-specific brefeldin A-resistance guanine nucleotide exchange factor 1 OS=Homo sapiens GN=GBF1 PE=1 SV=2 - [GBF1_HUMAN]	1.61	2	2	3	1859	206.3146	5.731934	0.784318	0.832389	0.979251	0.8653194	0.148447	0.2147406
O75339	Cartilage intermediate layer protein 1 OS=Homo sapiens GN=CILP PE=1 SV=4 - [CILP1_HUMAN]	2.62	3	3	3	1184	132.48	8.411621	0.911335	0.832532	0.851646	0.8651712	0.0296134	0.0761831
J3KNV4	Integrin alpha-7 OS=Homo sapiens GN=ITGA7 PE=1 SV=1 - [J3KNV4_HUMAN]	7.69	7	7	8	1131	123.5539	5.973145	0.854173	0.873785	0.865789	0.8645821	0.00176302	0.0311259
P09417	Dihydropteridine reductase OS=Homo sapiens GN=QDPR PE=1 SV=2 - [DHPR_HUMAN]	11.48	2	2	2	244	25.77303	7.371582	0.8787	0.901701	0.81308	0.8644939	0.0363128	0.0850649
B4E367	cDNA FLJ61564, highly similar to Plexin domain-containing protein 2 OS=Homo sapiens PE=2 SV=1 - [B4E367_HUMAN]	4.47	2	2	2	515	58.1468	6.366699	0.860092	0.926293	0.806221	0.8642017	0.0595946	0.1143971
A0A087WXM8	Basal cell adhesion molecule OS=Homo sapiens GN=BCAM PE=1 SV=1 - [A0A087WXM8_HUMAN]	32.14	14	14	24	588	63.65398	6.100098	0.875975	0.845791	0.870731	0.8641656	0.00466603	0.0398629
Q0PNF2	FEX1 OS=Homo sapiens PE=2 SV=1 - [Q0PNF2_HUMAN]	7.32	13	13	15	2570	275.2651	6.493652	0.885229	0.85527	0.851706	0.8640685	0.00606021	0.0421458
P23141	Liver carboxylesterase 1 OS=Homo sapiens GN=CES1 PE=1 SV=2 - [EST1_HUMAN]	18.52	9	9	12	567	62.48116	6.595215	0.849512	0.873956	0.867918	0.8637954	0.00290038	0.0360252
F6X2W2	Neuronal growth regulator 1 OS=Homo sapiens GN=NEGR1 PE=1 SV=1 - [F6X2W2_HUMAN]	14.38	3	3	3	299	32.82947	6.873535	0.892736	0.840049	0.857713	0.8634993	0.0126217	0.0508625

Biology Open • Supplementary information

Q59F44	Cytochrome b-5 isoform 1 variant (Fragment) OS=Homo sapiens PE=2 SV=1 - [Q59F44_HUMAN]	16.67	2	2	3	132	14.56314	5.719238	0.881726	0.80687	0.9012	0.8632655	0.0414861	0.0920252
Q9BQI9	Nuclear receptor-interacting protein 2 OS=Homo sapiens GN=NRIP2 PE=1 SV=3 - [NRIP2_HUMAN]	14.59	3	3	3	281	31.31117	8.396973	0.801365	0.841718	0.945291	0.8627912	0.0852903	0.1455914
O14558	Heat shock protein beta-6 OS=Homo sapiens GN=HSPB6 PE=1 SV=2 - [HSPB6_HUMAN]	30	5	5	10	160	17.12498	6.404785	0.857343	0.814496	0.91595	0.8625966	0.0428734	0.0935696
Q9BZQ8	Protein Niban OS=Homo sapiens GN=FAM129A PE=1 SV=1 - [NIBAN_HUMAN]	6.03	4	4	6	928	103.07	4.779785	0.917789	0.820426	0.849161	0.8624586	0.0413771	0.0919642
O15230	Laminin subunit alpha-5 OS=Homo sapiens GN=LAMA5 PE=1 SV=8 - [LAMA5_HUMAN]	20.54	58	60	108	3695	399.4786	7.02002	0.847949	0.881771	0.856528	0.8620828	0.00537341	0.0401071
A0A024R3M0	Signal recognition particle receptor ('docking protein'), isoform CRA_a OS=Homo sapiens GN=SRPR PE=4 SV=1 - [A0A024R3M0_HUMAN]	4.59	2	2	2	414	44.41783	8.191895	0.851723	0.876768	0.856757	0.861749	0.00304682	0.0360868
Q9Y4G6	Talin-2 OS=Homo sapiens GN=TLN2 PE=1 SV=4 - [TLN2_HUMAN]	11.96	12	24	35	2542	271.4434	5.566895	0.829425	0.856092	0.898361	0.8612929	0.0202994	0.0623036
B7Z609	cDNA FLJ51747, highly similar to Succinyl-CoA:3-ketoacid-coenzyme A transferase1, mitochondrial (EC 2.8.3.5) OS=Homo sapiens PE=2 SV=1 - [B7Z609_HUMAN]	5.39	2	2	2	334	36.63687	6.741699	0.838702	0.844688	0.90016	0.8611832	0.0192909	0.0608852
A9UFC0	Caspase 14 OS=Homo sapiens GN=CASP14 PE=2 SV=1 - [A9UFC0_HUMAN]	12.81	2	2	3	242	27.64888	5.338379	0.792721	0.834769	0.954715	0.860735	0.103019	0.1657428
B4DNR3	cDNA FLJ52710, highly similar to Abhydrolase domain-containing protein 14B OS=Homo sapiens PE=2 SV=1 - [B4DNR3_HUMAN]	37.3	5	5	11	185	19.78425	6.404785	0.877876	0.842586	0.861329	0.8605969	0.00530483	0.0401071
Q8WYJ5	Protein kinase C inhibitor-2 OS=Homo sapiens PE=2 SV=1 - [Q8WYJ5_HUMAN]	31.25	3	3	6	128	13.90945	7.049316	0.850774	0.846484	0.883175	0.8601439	0.00678806	0.0433606

Q13228	Selenium-binding protein 1 OS=Homo sapiens GN=SELENBP1 PE=1 SV=2 - [SBP1_HUMAN]	45.55	20	20	30	472	52.35763	6.366699	0.860625	0.810999	0.907928	0.8598509	0.0376324	0.0868934
B2RAQ8	cDNA, FLJ95058, highly similar to Homo sapiens carnitine palmitoyltransferase 1A (liver) (CPT1A),nuclear gene encoding mitochondrial protein, mRNA OS=Homo sapiens PE=2 SV=1 - [B2RAQ8_HUMAN]	6.08	4	4	6	773	88.28269	8.587402	0.850875	0.891861	0.836456	0.8597307	0.0137107	0.0523233
F6S6P2	Large proline-rich protein BAG6 (Fragment) OS=Homo sapiens GN=BAG6 PE=1 SV=1 - [F6S6P2_HUMAN]	3.3	2	2	2	515	54.63625	5.77002	0.840388	0.907209	0.83052	0.8593722	0.0281075	0.0742152
C9JJV6	Myeloid-associated differentiation marker (Fragment) OS=Homo sapiens GN=MYADM PE=1 SV=6 - [C9JJV6_HUMAN]	22.6	2	2	3	146	15.85308	8.645996	0.950182	0.847882	0.778898	0.8589871	0.105217	0.1679998
Q5T4S7	E3 ubiquitin-protein ligase UBR4 OS=Homo sapiens GN=UBR4 PE=1 SV=1 - [UBR4_HUMAN]	1.22	5	5	5	5183	573.4765	6.036621	0.840222	0.862049	0.872757	0.8583428	0.00453606	0.0398479
P40123	Adenylyl cyclase-associated protein 2 OS=Homo sapiens GN=CAP2 PE=1 SV=1 - [CAP2_HUMAN]	19.29	6	7	7	477	52.79082	6.366699	0.853205	0.795029	0.924107	0.8574471	0.0622171	0.1168729
P08559	Pyruvate dehydrogenase E1 component subunit alpha, somatic form, mitochondrial OS=Homo sapiens GN=PDHA1 PE=1 SV=3 - [ODPA_HUMAN]	11.54	5	5	7	390	43.26761	8.060059	0.95032	0.820711	0.801242	0.8574244	0.0929193	0.154213
A0A024R493	EF-hand domain family, member D1, isoform CRA_a OS=Homo sapiens GN=EFHD1 PE=4 SV=1 - [A0A024R493_HUMAN]	32.22	5	6	9	239	26.93883	5.38916	0.841797	0.850011	0.879576	0.8571278	0.0063853	0.0423724
Q59HC1	Beige-like protein variant (Fragment) OS=Homo sapiens PE=2 SV=1 - [Q59HC1_HUMAN]	1.66	2	2	2	1504	166.7533	5.643066	0.899466	0.87936	0.791782	0.8568693	0.0494208	0.1017576
Q9UGT4	Sushi domain-containing protein 2 OS=Homo sapiens GN=SUSD2 PE=1 SV=1 - [SUSD2_HUMAN]	8.52	5	5	9	822	90.14969	6.277832	0.891358	0.840798	0.837872	0.8566763	0.0143586	0.0530821
Q9H4B7	Tubulin beta-1 chain OS=Homo sapiens GN=TUBB1 PE=1	14.63	3	7	18	451	50.29456	5.17334	0.698693	0.901947	0.968544	0.8563948	0.218851	0.2883654

Biology Open • Supplementary information

	SV=1 - [TBB1_HUMAN]													
Q7Z7G0	Target of Nesh-SH3 OS=Homo sapiens GN=ABI3BP PE=1 SV=1 - [TARSH_HUMAN]	5.4	6	6	6	1075	118.5688	9.437012	0.851943	0.856834	0.860118	0.8562983	0.000273049	0.0212234
Q96CS3	FAS-associated factor 2 OS=Homo sapiens GN=FAF2 PE=1 SV=2 - [FAF2_HUMAN]	9.44	2	2	2	445	52.59055	5.617676	0.852667	0.725983	0.989727	0.8561254	0.199453	0.2691979
Q03252	Lamin-B2 OS=Homo sapiens GN=LMNB2 PE=1 SV=4 - [LMNB2_HUMAN]	26.61	16	18	23	620	69.9057	5.592285	0.821551	0.882388	0.86313	0.8556896	0.0151242	0.0543476
X6RDA4	Paraspeckle component 1 (Fragment) OS=Homo sapiens GN=PSPC1 PE=1 SV=1 - [X6RDA4_HUMAN]	14.11	2	2	2	248	27.25719	5.452637	0.775066	0.83619	0.954807	0.8553544	0.111289	0.1744043
V9GYR2	Sodium/potassium-transporting ATPase subunit beta (Fragment) OS=Homo sapiens GN=ATP1B1 PE=1 SV=1 - [V9GYR2_HUMAN]	19.23	2	2	5	130	15.1156	6.163574	0.87534	0.832557	0.857084	0.8549936	0.00722696	0.0439255
Q14558	Phosphoribosyl pyrophosphate synthase-associated protein 1 OS=Homo sapiens GN=PRPSAP1 PE=1 SV=2 - [KPRA_HUMAN]	11.24	2	3	4	356	39.36863	7.195801	0.74032	0.880006	0.943311	0.8545458	0.136106	0.2010994
Q14126	Desmoglein-2 OS=Homo sapiens GN=DSG2 PE=1 SV=2 - [DSG2_HUMAN]	3.13	2	2	2	1118	122.2176	5.236816	0.857836	0.767872	0.937026	0.8542447	0.0964095	0.1581135
Q5TFQ8	Signal-regulatory protein beta-1 isoform 3 OS=Homo sapiens GN=SIRPB1 PE=1 SV=1 - [SIRBL_HUMAN]	10.55	3	3	3	398	43.33227	7.825684	0.883611	0.812058	0.865528	0.8537322	0.0208946	0.0628695
Q27J81	Inverted formin-2 OS=Homo sapiens GN=INF2 PE=1 SV=2 - [INF2_HUMAN]	7.29	7	7	7	1249	135.5397	5.376465	0.847096	0.768226	0.944762	0.8533612	0.102866	0.1656524
A0A0D9SFS3	2-oxoglutarate dehydrogenase, mitochondrial OS=Homo sapiens GN=OGDH PE=1 SV=1 - [A0A0D9SFS3_HUMAN]	17.58	12	12	15	1001	113.2411	7.078613	0.853888	0.842882	0.861876	0.8528819	0.00139786	0.0286773
B4DJI2	cDNA FLJ53342, highly similar to Granulins OS=Homo sapiens PE=2 SV=1 - [B4DJI2_HUMAN]	4.53	2	2	2	530	56.81268	6.873535	0.889849	0.808333	0.858321	0.8521679	0.0248157	0.0690048

Biology Open • Supplementary information

O00186	Syntaxin-binding protein 3 OS=Homo sapiens GN=STXBP3 PE=1 SV=2 - [STXB3_HUMAN]	5.91	3	3	3	592	67.72085	7.796387	0.853999	0.921485	0.781003	0.8521623	0.0677262	0.12352
B4DNJ3	cDNA FLJ57129, highly similar to 1,4-alpha-glucan branching enzyme (EC 2.4.1.18) OS=Homo sapiens PE=2 SV=1 - [B4DNJ3_HUMAN]	7.31	3	3	3	465	53.32089	5.541504	0.815086	0.918702	0.822446	0.8520782	0.0473348	0.099274
B7Z1C5	Glutathione synthetase OS=Homo sapiens PE=2 SV=1 - [B7Z1C5_HUMAN]	11.36	4	4	4	405	44.81038	6.565918	0.897048	0.790396	0.867808	0.8517508	0.0431063	0.0938359
P78559	Microtubule-associated protein 1A OS=Homo sapiens GN=MAP1A PE=1 SV=6 - [MAP1A_HUMAN]	2.85	4	5	5	2803	305.2982	4.919434	0.839859	0.84078	0.874327	0.8516554	0.00579195	0.0412753
B3KRA1	cDNA FLJ33914 fis, clone CTONG2016575, highly similar to SON PROTEIN OS=Homo sapiens PE=2 SV=1 - [B3KRA1_HUMAN]	5.87	2	2	2	579	63.50062	8.074707	0.901733	0.82826	0.823735	0.8512427	0.0276838	0.0736361
Q01082	Spectrin beta chain, non-erythrocytic 1 OS=Homo sapiens GN=SPTBN1 PE=1 SV=2 - [SPTB2_HUMAN]	35.83	70	73	106	2364	274.4387	5.566895	0.853415	0.840388	0.859817	0.8512069	0.00147264	0.0290552
E7EV99	Alpha-adducin OS=Homo sapiens GN=ADD1 PE=1 SV=1 - [E7EV99_HUMAN]	14.56	6	6	8	632	70.01236	6.455566	0.850487	0.847993	0.854218	0.8508992	0.000147161	0.0212234
E9PHY5	Band 4.1-like protein 2 OS=Homo sapiens GN=EPB41L2 PE=1 SV=1 - [E9PHY5_HUMAN]	21.6	14	17	21	935	104.296	5.38916	0.889982	0.790766	0.869539	0.850096	0.0383825	0.0876425
Q14789	Golgin subfamily B member 1 OS=Homo sapiens GN=GOLGB1 PE=1 SV=2 - [GOGB1_HUMAN]	1.07	2	3	5	3259	375.7897	4.995605	0.879399	0.822671	0.847517	0.8498622	0.0117478	0.0494101
R4GNE8	Dipeptidyl peptidase 2 (Fragment) OS=Homo sapiens GN=DPP7 PE=1 SV=1 - [R4GNE8_HUMAN]	6.62	2	2	2	272	29.34	9.45166	0.911856	0.832962	0.804765	0.849861	0.0426691	0.0935455
A0A087WUI8	Mannose-1-phosphate guanyltransferase alpha OS=Homo sapiens GN=GMPPA PE=1 SV=1 - [A0A087WUI8_HUMAN]	6.77	2	2	2	399	43.64143	7.166504	0.910055	0.88227	0.756617	0.8496472	0.0860299	0.1464109
P16152	Carbonyl reductase [NADPH] 1 OS=Homo sapiens GN=CBR1 PE=1 SV=3 - [CBR1_HUMAN]	35.38	3	6	11	277	30.35586	8.32373	0.916818	0.84048	0.790963	0.8494204	0.0543249	0.1077269

Biology Open • Supplementary information

Q9BQ69	O-acetyl-ADP-ribose deacetylase MACROD1 OS=Homo sapiens GN=MACROD1 PE=1 SV=2 - [MACD1_HUMAN]	7.69	2	2	2	325	35.48272	9.510254	0.803747	0.83216	0.912246	0.8493842	0.043501	0.0942707
C9JLU1	DNA-directed RNA polymerases I, II, and III subunit RPABC3 (Fragment) OS=Homo sapiens GN=POLR2H PE=1 SV=6 - [C9JLU1_HUMAN]	16.22	2	2	2	148	16.91434	4.678223	0.862835	0.861237	0.82346	0.8491775	0.00719953	0.0439255
Q59H46	Integrin beta (Fragment) OS=Homo sapiens PE=2 SV=1 - [Q59H46_HUMAN]	7.26	8	8	9	1515	169.0392	6.493652	0.877027	0.844229	0.82623	0.8491623	0.00957892	0.0475435
B4DSW9	cDNA FLJ59415, highly similar to Beta-catenin OS=Homo sapiens PE=2 SV=1 - [B4DSW9_HUMAN]	19.04	8	10	13	709	77.46962	6.379395	0.86447	0.856053	0.825145	0.848556	0.00617393	0.0423147
Q9NZN4	EH domain-containing protein 2 OS=Homo sapiens GN=EHD2 PE=1 SV=2 - [EHD2_HUMAN]	51.01	19	24	60	543	61.12271	6.455566	0.837003	0.875703	0.829379	0.8473615	0.00871157	0.0461063
E9PNF3	L-aminoadipate-semialdehyde dehydrogenase-phosphopantetheinyl transferase (Fragment) OS=Homo sapiens GN=AASDHPPT PE=1 SV=1 - [E9PNF3_HUMAN]	12.5	2	2	2	160	18.68468	7.503418	0.797154	0.870703	0.871385	0.8464144	0.0247676	0.0689744
P14555	Phospholipase A2, membrane associated OS=Homo sapiens GN=PLA2G2A PE=1 SV=2 - [PA2GA_HUMAN]	29.17	5	5	9	144	16.07179	9.231934	0.859254	0.889527	0.788745	0.8458419	0.0355228	0.0835033
D6RCD0	Estradiol 17-beta-dehydrogenase 11 OS=Homo sapiens GN=HSD17B11 PE=1 SV=2 - [D6RCD0_HUMAN]	26.17	5	5	6	256	28.08429	9.539551	0.806303	0.881228	0.848176	0.8452356	0.0190624	0.0605312
P00918	Carbonic anhydrase 2 OS=Homo sapiens GN=CA2 PE=1 SV=2 - [CAH2_HUMAN]	33.85	7	7	14	260	29.22792	7.400879	0.824867	0.871491	0.839199	0.8451856	0.00783874	0.045203
Q9BTE3	Mini-chromosome maintenance complex-binding protein OS=Homo sapiens GN=MCMBP PE=1 SV=2 - [MCMBP_HUMAN]	5.14	3	3	3	642	72.93379	5.871582	0.782187	0.824722	0.927669	0.8448591	0.0695094	0.1259579
B3KUR3	cDNA FLJ40459 fis, clone TESTI2041800, highly similar to BISPHOSPHOGLYCERATE MUTASE (EC 5.4.2.4)	17.77	3	3	4	242	27.97443	5.846191	0.875876	0.836218	0.82043	0.8441748	0.0110176	0.0493307

Biology Open • Supplementary information

	OS=Homo sapiens PE=2 SV=1 - [B3KUR3_HUMAN]													
B4DWD0	cDNA FLJ50716 OS=Homo sapiens PE=2 SV=1 - [B4DWD0_HUMAN]	18.35	2	2	2	109	13.17766	8.338379	0.840232	0.915993	0.772999	0.8430746	0.0628192	0.1176123
A0A024RDI4	Ankyrin 2, neuronal, isoform CRA_a OS=Homo sapiens GN=ANK2 PE=4 SV=1 - [A0A024RDI4_HUMAN]	6.86	7	10	14	1851	203.3086	6.227051	0.882946	0.761778	0.884154	0.8429591	0.0607847	0.1153342
K7EQ02	DAZ-associated protein 1 (Fragment) OS=Homo sapiens GN=DAZAP1 PE=1 SV=2 - [K7EQ02_HUMAN]	9.79	2	2	3	327	34.99812	7.85498	0.81074	0.850617	0.867489	0.8429486	0.0112828	0.0493721
B3KSC3	cDNA FLJ35987 fis, clone TESTI2014269, highly similar to D-3-phosphoglycerate dehydrogenase (EC 1.1.1.95) OS=Homo sapiens PE=2 SV=1 - [B3KSC3_HUMAN]	8.82	3	3	4	499	52.97942	7.122559	0.803778	0.869435	0.855393	0.8428689	0.0157577	0.0550258
O60565	Gremlin-1 OS=Homo sapiens GN=GREM1 PE=1 SV=1 - [GREM1_HUMAN]	20.11	2	2	2	184	20.68345	9.393066	0.853067	0.900145	0.775389	0.842867	0.0496282	0.1018392
B4E1K8	Eukaryotic translation initiation factor 3 subunit D OS=Homo sapiens GN=EIF3D PE=2 SV=1 - [B4E1K8_HUMAN]	10.86	3	3	3	451	52.79421	5.79541	0.883972	0.737387	0.904295	0.8418845	0.0950672	0.1563678
B7Z3E5	cDNA FLJ53573, highly similar to Myosin Ic OS=Homo sapiens PE=2 SV=1 - [B7Z3E5_HUMAN]	29.93	24	24	45	1039	118.9824	9.407715	0.859051	0.838298	0.828053	0.8418004	0.00330565	0.036808
A0A024R529	Dihydroxyacetone kinase 2 homolog (Yeast), isoform CRA_a OS=Homo sapiens GN=DAK PE=4 SV=1 - [A0A024R529_HUMAN]	11.48	4	4	5	575	58.94005	7.48877	0.833149	0.805073	0.88593	0.8413841	0.0216078	0.0637577
P10909	Clusterin OS=Homo sapiens GN=CLU PE=1 SV=1 - [CLUS_HUMAN]	20.49	10	10	26	449	52.46101	6.265137	0.817556	0.893896	0.812645	0.8413656	0.026409	0.0717972
B4E2F9	cDNA FLJ57038, highly similar to Filamin-A OS=Homo sapiens PE=2 SV=1 - [B4E2F9_HUMAN]	60.77	2	41	193	780	82.50049	6.55127	0.803427	0.832732	0.887378	0.8411788	0.0231605	0.0660677

Biology Open • Supplementary information

B3KY63	cDNA FLJ16830 fis, clone UTERU3022536, highly similar to Chromodomain helicase-DNA-binding protein 4 (EC 3.6.1.-) OS=Homo sapiens PE=2 SV=1 - [B3KY63_HUMAN]	1.96	3	3	3	1886	215.1489	5.896973	0.830214	0.813047	0.878812	0.8406908	0.0149408	0.0540327
Q9BUT1	3-hydroxybutyrate dehydrogenase type 2 OS=Homo sapiens GN=BDH2 PE=1 SV=2 - [BDH2_HUMAN]	11.02	2	2	3	245	26.70672	7.649902	0.913906	0.79354	0.812798	0.8400814	0.0504028	0.10263
P06396	Gelsolin OS=Homo sapiens GN=GSN PE=1 SV=1 - [GELS_HUMAN]	39.51	2	26	52	782	85.64419	6.277832	0.858899	0.824031	0.835695	0.8395415	0.00405388	0.0390309
B3KU69	cDNA FLJ39269 fis, clone OCBBF2010420, highly similar to Guanylate cyclase soluble subunit alpha-3 (EC 4.6.1.2) OS=Homo sapiens PE=2 SV=1 - [B3KU69_HUMAN]	4.49	2	2	3	690	77.30324	6.990723	0.913308	0.738412	0.866507	0.839409	0.0916282	0.1526499
P08294	Extracellular superoxide dismutase [Cu-Zn] OS=Homo sapiens GN=SOD3 PE=1 SV=2 - [SODE_HUMAN]	37.08	7	7	18	240	25.83468	6.609863	0.823854	0.827362	0.866549	0.8392548	0.00716949	0.0439255
Q14669	E3 ubiquitin-protein ligase TRIP12 OS=Homo sapiens GN=TRIP12 PE=1 SV=1 - [TRIPC_HUMAN]	2.11	3	3	3	1992	220.2958	8.484863	0.888046	0.758599	0.869913	0.8388528	0.0576597	0.1121076
P12110	Collagen alpha-2(VI) chain OS=Homo sapiens GN=COL6A2 PE=1 SV=4 - [CO6A2_HUMAN]	30.52	15	30	113	1019	108.5119	6.214355	0.852422	0.829299	0.831752	0.8378241	0.00203828	0.0323797
H7C4V4	Nitric oxide synthase, endothelial (Fragment) OS=Homo sapiens GN=NOS3 PE=1 SV=1 - [H7C4V4_HUMAN]	10.46	2	2	2	306	33.92566	7.76709	0.863457	0.872184	0.77603	0.8372234	0.0337795	0.0810769
Q59F20	Neuropilin-1 variant (Fragment) OS=Homo sapiens PE=2 SV=1 - [Q59F20_HUMAN]	5.51	3	3	3	890	99.63078	5.935059	0.950427	0.760351	0.800595	0.8371243	0.106334	0.169145
X5D932	Glutathione S-transferase mu 1 isoform C (Fragment) OS=Homo sapiens GN=GSTM1 PE=2 SV=1 - [X5D932_HUMAN]	22.65	2	4	4	181	21.23876	7.85498	0.911652	0.812154	0.78716	0.8369886	0.0503339	0.1026144
C9JM82	Septin-5 (Fragment) OS=Homo sapiens GN=SEPT5 PE=1 SV=1 - [C9JM82_HUMAN]	12.62	2	2	2	301	34.6506	6.20166	0.820835	0.750308	0.936957	0.8360335	0.0947403	0.156137

Biology Open • Supplementary information

Q59F15	COL4A1 protein variant (Fragment) OS=Homo sapiens PE=2 SV=1 - [Q59F15_HUMAN]	9.21	6	6	15	847	82.92704	8.70459	0.817547	0.831787	0.857606	0.8356467	0.00504993	0.0401071
K7EKL8	Intercellular adhesion molecule 1 (Fragment) OS=Homo sapiens GN=ICAM1 PE=1 SV=1 - [K7EKL8_HUMAN]	15.56	2	2	3	180	19.4311	7.181152	0.98898	0.730625	0.786269	0.8352916	0.17079	0.2385001
Q15661	Tryptase alpha/beta-1 OS=Homo sapiens GN=TPSAB1 PE=1 SV=1 - [TRYB1_HUMAN]	35.64	2	9	38	275	30.49547	7.10791	0.839028	0.823534	0.839386	0.8339823	0.000989143	0.0269712
Q9NPH2	Inositol-3-phosphate synthase 1 OS=Homo sapiens GN=ISYNA1 PE=1 SV=1 - [INO1_HUMAN]	6.63	4	4	4	558	61.0291	5.757324	0.870647	0.82959	0.800975	0.8337373	0.0144688	0.0532358
Q86T65	Disheveled-associated activator of morphogenesis 2 OS=Homo sapiens GN=DAAM2 PE=1 SV=3 - [DAAM2_HUMAN]	2.53	2	2	3	1068	123.4204	6.800293	0.918805	0.721589	0.857871	0.8327552	0.103069	0.1657428
B7Z321	cDNA FLJ53330, highly similar to Exocyst complex component 4 OS=Homo sapiens PE=2 SV=1 - [B7Z321_HUMAN]	3.09	2	2	3	873	98.99974	6.521973	0.762355	0.908507	0.826022	0.8322947	0.0581446	0.1126707
A8K7I4	Calcium-activated chloride channel regulator 1 OS=Homo sapiens GN=CLCA1 PE=1 SV=3 - [CLCA1_HUMAN]	7	6	6	9	914	100.1631	6.366699	0.909944	0.746553	0.840216	0.8322377	0.0712124	0.1280953
H3BM67	Nucleolar protein 3 (Fragment) OS=Homo sapiens GN=NOL3 PE=1 SV=6 - [H3BM67_HUMAN]	19.57	2	2	2	138	14.9154	5.046387	0.850353	0.836906	0.809445	0.8322347	0.00510925	0.0401071
H0YIB4	Serine/arginine-rich-splicing factor 9 (Fragment) OS=Homo sapiens GN=SRSF9 PE=1 SV=6 - [H0YIB4_HUMAN]	20.18	2	2	2	109	12.71628	5.376465	0.847899	0.874461	0.773621	0.8319937	0.0307807	0.0773766
Q96PD5	N-acetylmuramoyl-L-alanine amidase OS=Homo sapiens GN=PGLYRP2 PE=1 SV=1 - [PGRP2_HUMAN]	4.86	2	2	4	576	62.17789	7.547363	0.906857	0.807862	0.780027	0.8315818	0.048454	0.1005287
Q86XP3	ATP-dependent RNA helicase DDX42 OS=Homo sapiens GN=DDX42 PE=1 SV=1 - [DDX42_HUMAN]	2.35	2	2	2	938	102.9119	7.02002	0.809557	0.872556	0.810403	0.8308385	0.014868	0.0540327
Q9ULC3	Ras-related protein Rab-23 OS=Homo sapiens GN=RAB23 PE=1 SV=1 - [RAB23_HUMAN]	11.81	2	2	2	237	26.64255	6.595215	0.868966	0.795361	0.828072	0.8307996	0.0154683	0.0546557

Biology Open • Supplementary information

Biology Open (2019): doi:10.1242/bio.042838: Supplementary information

P00491	Purine nucleoside phosphorylase OS=Homo sapiens GN=PNP PE=1 SV=2 - [PNPH_HUMAN]	29.07	6	6	13	289	32.09716	6.946777	0.806598	0.777099	0.906935	0.8302105	0.0496123	0.1018392
Q96C19	EF-hand domain-containing protein D2 OS=Homo sapiens GN=EFHD2 PE=1 SV=1 - [EFHD2_HUMAN]	18.33	2	3	5	240	26.68046	5.19873	0.823135	0.778176	0.887916	0.8297424	0.0332606	0.0806036
P20645	Cation-dependent mannose-6-phosphate receptor OS=Homo sapiens GN=M6PR PE=1 SV=1 - [MPRD_HUMAN]	15.52	3	3	5	277	30.97338	5.833496	0.818658	0.875645	0.793536	0.8292797	0.0196498	0.0612255
B7Z6E8	cDNA FLJ59425, highly similar to SH3-domain kinase-binding protein 1 OS=Homo sapiens PE=2 SV=1 - [B7Z6E8_HUMAN]	7.43	2	2	2	404	44.20802	8.98291	0.854405	0.832819	0.796669	0.8279644	0.00944919	0.0472949
I6MBV4	Receptor protein-tyrosine kinase OS=Homo sapiens PE=2 SV=1 - [I6MBV4_HUMAN]	2.5	2	3	3	1161	128.3311	6.404785	0.868233	0.8439	0.77023	0.8274544	0.0279385	0.0740148
P55083	Microfibril-associated glycoprotein 4 OS=Homo sapiens GN=MFAP4 PE=1 SV=2 - [MFAP4_HUMAN]	9.8	2	2	6	255	28.62989	5.630371	0.81758	0.86276	0.800718	0.8270193	0.0112708	0.0493721
B4E0K5	Mitogen-activated protein kinase 14 OS=Homo sapiens GN=MAPK14 PE=1 SV=1 - [B4E0K5_HUMAN]	16.61	3	4	4	283	32.33627	4.919434	1.00341	0.712674	0.76486	0.8269828	0.192889	0.2620101
A0A087WXI2	IgGFc-binding protein OS=Homo sapiens GN=FCGBP PE=1 SV=1 - [A0A087WXI2_HUMAN]	7.94	19	19	27	4204	444.9177	5.351074	0.842674	0.769004	0.867474	0.8263841	0.0278035	0.0738735
P30038	Delta-1-pyrroline-5-carboxylate dehydrogenase, mitochondrial OS=Homo sapiens GN=ALDH4A1 PE=1 SV=3 - [AL4A1_HUMAN]	5.15	3	3	3	563	61.6806	8.074707	0.790334	0.792784	0.896018	0.8263787	0.0379604	0.0871159
F8W9J4	Dystonin OS=Homo sapiens GN=DST PE=1 SV=1 - [F8W9J4_HUMAN]	0.54	2	3	3	7461	847.444	5.249512	0.821141	0.878728	0.774892	0.8249202	0.0281896	0.074374
A0A024QZB4	Sulfotransferase OS=Homo sapiens GN=hCG_1993905 PE=3 SV=1 - [A0A024QZB4_HUMAN]	12.88	2	3	6	295	34.17515	6.624512	0.769914	0.900637	0.80156	0.8240371	0.046599	0.0982789
B7Z5Z9	cDNA FLJ51066, highly similar to Ras-related protein Rab-6A OS=Homo sapiens PE=2 SV=1 - [B7Z5Z9_HUMAN]	33.33	3	4	6	144	16.60754	5.439941	0.846133	0.675199	0.949087	0.8234729	0.157684	0.2249392

Biology Open • Supplementary information

Q16853	Membrane primary amine oxidase OS=Homo sapiens GN=AOC3 PE=1 SV=3 - [AOC3_HUMAN]	22.28	13	13	37	763	84.56829	6.521973	0.820809	0.797496	0.8501	0.8228017	0.00729493	0.0440167
Q9NRN5	Olfactomedin-like protein 3 OS=Homo sapiens GN=OLFML3 PE=2 SV=1 - [OLFL3_HUMAN]	26.85	9	9	10	406	45.98127	6.565918	0.833901	0.85493	0.776143	0.8216579	0.0169985	0.0572932
Q6N092	Putative uncharacterized protein DKFZp686K18196 (Fragment) OS=Homo sapiens GN=DKFZp686K18196 PE=2 SV=1 - [Q6N092_HUMAN]	34.3	2	14	69	519	56.38786	6.932129	0.838833	0.831205	0.793875	0.8213041	0.00598805	0.0419659
Q5JSH3	WD repeat-containing protein 44 OS=Homo sapiens GN=WDR44 PE=1 SV=1 - [WDR44_HUMAN]	3.29	2	2	2	913	101.3037	5.452637	0.87316	0.715429	0.875093	0.8212271	0.0775215	0.1360078
I1VZV6	Hemoglobin alpha 1 OS=Homo sapiens GN=HBA1 PE=3 SV=1 - [I1VZV6_HUMAN]	71.83	4	12	271	142	15.26996	9.026855	0.848989	0.777564	0.834283	0.8202784	0.0143643	0.0530821
B7Z6Y2	cDNA FLJ54942, highly similar to Homo sapiens bridging integrator 1 (BIN1), transcript variant 10, mRNA OS=Homo sapiens PE=2 SV=1 - [B7Z6Y2_HUMAN]	6.23	2	2	2	385	43.13854	5.325684	0.843467	0.80282	0.81403	0.8201057	0.00450874	0.0398479
P09471	Guanine nucleotide-binding protein G(o) subunit alpha OS=Homo sapiens GN=GNAO1 PE=1 SV=4 - [GNAO_HUMAN]	22.88	4	6	12	354	40.0249	5.528809	0.814909	0.827745	0.814911	0.8191883	0.000559489	0.0241782
P01023	Alpha-2-macroglobulin OS=Homo sapiens GN=A2M PE=1 SV=3 - [A2MG_HUMAN]	36.64	37	43	128	1474	163.1879	6.455566	0.810408	0.837403	0.808916	0.8189091	0.00260298	0.0357839
Q6DKJ4	Nucleoredoxin OS=Homo sapiens GN=NXN PE=1 SV=2 - [NXN_HUMAN]	9.66	3	3	3	435	48.36164	4.970215	0.86835	0.785229	0.797259	0.816946	0.0194891	0.0611972
H0YKI0	Deoxyuridine 5'-triphosphate nucleotidohydrolase, mitochondrial OS=Homo sapiens GN=DUT PE=1 SV=1 - [H0YKI0_HUMAN]	15.04	2	2	2	226	23.69714	9.656738	0.887785	0.858292	0.703449	0.8165085	0.0848888	0.1450523
P63092	Guanine nucleotide-binding protein G(s) subunit alpha isoforms short OS=Homo sapiens GN=GNAS PE=1 SV=1 -	17.01	5	6	15	394	45.6359	5.820801	0.793674	0.849157	0.805836	0.8162222	0.00828965	0.0456887

Biology Open • Supplementary information

	[GNAS2_HUMAN]													
B7Z3I9	Delta-aminolevulinic acid dehydratase OS=Homo sapiens PE=2 SV=1 - [B7Z3I9_HUMAN]	26.84	6	6	12	313	34.49749	7.195801	0.790999	0.829448	0.826663	0.8157037	0.00448084	0.0398479
Q8IV08	Phospholipase D3 OS=Homo sapiens GN=PLD3 PE=1 SV=1 - [PLD3_HUMAN]	9.8	3	3	4	490	54.67051	6.468262	0.839181	0.810849	0.796895	0.8156417	0.00452228	0.0398479
P27658	Collagen alpha-1(VIII) chain OS=Homo sapiens GN=COL8A1 PE=1 SV=2 - [CO8A1_HUMAN]	3.9	2	2	3	744	73.31713	9.612793	0.732118	0.92206	0.79203	0.8154028	0.081165	0.1407498
Q96PE2	Rho guanine nucleotide exchange factor 17 OS=Homo sapiens GN=ARHGEF17 PE=1 SV=1 - [ARHGH_HUMAN]	1.36	2	2	2	2063	221.5348	6.290527	0.846484	0.760257	0.837201	0.8146472	0.0210518	0.063146
D6RIZ4	Major facilitator superfamily domain-containing protein 10 OS=Homo sapiens GN=MFSD10 PE=1 SV=1 - [D6RIZ4_HUMAN]	6.46	2	2	4	356	38.2232	9.72998	0.811575	0.76682	0.864521	0.8143056	0.0223503	0.0647738
P02763	Alpha-1-acid glycoprotein 1 OS=Homo sapiens GN=ORM1 PE=1 SV=1 - [A1AG1_HUMAN]	47.76	5	9	29	201	23.49676	5.020996	0.813966	0.811697	0.813484	0.813049	1.36E-05	0.0119321
F5GYN4	Ubiquitin thioesterase OTUB1 OS=Homo sapiens GN=OTUB1 PE=1 SV=1 - [F5GYN4_HUMAN]	21.16	4	4	7	241	28.03298	5.287598	0.815787	0.764715	0.858199	0.8129005	0.0202327	0.0623036
Q9NQT8	Kinesin-like protein KIF13B OS=Homo sapiens GN=KIF13B PE=1 SV=2 - [KI13B_HUMAN]	2.3	3	3	3	1826	202.6626	5.884277	0.882402	0.755111	0.798098	0.8118704	0.0372954	0.0864865
Q86YQ4	Alpha-1 globin (Fragment) OS=Homo sapiens GN=HBA1 PE=3 SV=1 - [Q86YQ4_HUMAN]	57.95	3	6	100	88	9.476097	9.202637	0.846747	0.713877	0.874776	0.8118003	0.0630277	0.1178723
P10301	Ras-related protein R-Ras OS=Homo sapiens GN=RRAS PE=1 SV=1 - [RRAS_HUMAN]	32.11	4	6	12	218	23.46589	6.932129	0.795109	0.83094	0.808949	0.8116658	0.00305416	0.0360868
P48163	NADP-dependent malic enzyme OS=Homo sapiens GN=ME1 PE=1 SV=1 - [MAOX_HUMAN]	6.99	3	3	3	572	64.10907	6.125488	0.869374	0.849748	0.712838	0.8106532	0.0614465	0.1161724
O60504	Vinexin OS=Homo sapiens GN=SORBS3 PE=1 SV=2 -	19.37	3	9	13	671	75.29491	9.45166	0.839479	0.806095	0.785771	0.8104483	0.00675305	0.0433606

	[VINEX_HUMAN]														
B7Z709	cDNA FLJ50250, highly similar to Structural maintenance of chromosome 1-like 1 protein OS=Homo sapiens PE=2 SV=1 - [B7Z709_HUMAN]	7.17	3	3	3	516	59.05335	9.231934	0.777074	0.882167	0.771447	0.8102294	0.0341639	0.0816124
B7ZC39	Endophilin-B2 OS=Homo sapiens GN=SH3GLB2 PE=1 SV=1 - [B7ZC39_HUMAN]	10.96	4	4	4	374	41.64245	5.973145	0.806185	0.818318	0.802802	0.8091017	0.000608275	0.0241782
P22059	Oxysterol-binding protein 1 OS=Homo sapiens GN=OSBP PE=1 SV=1 - [OSBP1_HUMAN]	4.83	2	2	2	807	89.36456	7.29834	0.798977	0.816891	0.807824	0.8078973	0.000723933	0.0242397
B2R941	cDNA, FLJ94198, highly similar to Homo sapiens carboxypeptidase A3 (mast cell) (CPA3), mRNA OS=Homo sapiens PE=2 SV=1 - [B2R941_HUMAN]	25.18	13	13	35	417	48.6788	8.997559	0.806666	0.810322	0.805357	0.8074482	5.95E-05	0.0173763
B7Z242	Amine oxidase [flavin-containing] OS=Homo sapiens PE=2 SV=1 - [B7Z242_HUMAN]	20.04	7	10	18	504	57.22244	7.518066	0.861979	0.74603	0.814281	0.8074302	0.029198	0.0756786
B4DVV1	cDNA FLJ51526, highly similar to Homo sapiens aldehyde dehydrogenase 16 family, member A1 (ALDH16A1), mRNA OS=Homo sapiens PE=2 SV=1 - [B4DVV1_HUMAN]	4.08	2	2	3	637	66.84957	7.063965	0.782696	0.765566	0.873042	0.8071011	0.0285956	0.0748616
Q5QNY5	Peroxisomal biogenesis factor 19 (Fragment) OS=Homo sapiens GN=PEX19 PE=1 SV=1 - [Q5QNY5_HUMAN]	16.6	2	2	2	235	26.12039	4.437012	0.834422	0.762796	0.82339	0.8068695	0.013032	0.0512946
Q5U0F2	Proteolipid protein 1 (Pelizaeus-Merzbacher disease, spastic paraplegia 2, uncomplicated) OS=Homo sapiens PE=2 SV=1 - [Q5U0F2_HUMAN]	8.68	2	2	2	242	26.27021	7.913574	0.844189	0.81205	0.763634	0.8066243	0.0143432	0.0530821
A0A0G2JI89	Abhydrolase domain-containing protein 16A (Fragment) OS=Homo sapiens GN=ABHD16A PE=1 SV=2 - [A0A0G2JI89_HUMAN]	8.75	2	2	2	263	29.62419	9.290527	0.763465	0.756187	0.899653	0.8064351	0.0534808	0.106678
P07585	Decorin OS=Homo sapiens GN=DCN PE=1 SV=1 - [PGS2_HUMAN]	54.04	15	18	56	359	39.72184	8.543457	0.790433	0.848206	0.779184	0.8059412	0.0119221	0.0495204

Biology Open • Supplementary information

K7ER90	Eukaryotic translation initiation factor 3 subunit G (Fragment) OS=Homo sapiens GN=EIF3G PE=1 SV=1 - [K7ER90_HUMAN]	18.06	2	2	2	227	25.42584	5.490723	0.83253	0.760819	0.823538	0.8056289	0.0131994	0.051412
O60216	Double-strand-break repair protein rad21 homolog OS=Homo sapiens GN=RAD21 PE=1 SV=2 - [RAD21_HUMAN]	5.71	2	2	2	631	71.64466	4.652832	0.82642	0.765454	0.822707	0.8048606	0.0100707	0.0483662
A8K129	cDNA FLJ75032, highly similar to Homo sapiens unc-84 homolog B (C. elegans) (UNC84B), mRNA OS=Homo sapiens PE=2 SV=1 - [A8K129_HUMAN]	10.04	5	5	6	717	80.31856	6.814941	0.79699	0.736676	0.879521	0.8043957	0.0419985	0.0926217
P19652	Alpha-1-acid glycoprotein 2 OS=Homo sapiens GN=ORM2 PE=1 SV=2 - [A1AG2_HUMAN]	40.8	4	8	15	201	23.58763	5.109863	0.832435	0.816126	0.76392	0.8041601	0.0109504	0.0493157
O75953	DnaJ homolog subfamily B member 5 OS=Homo sapiens GN=DNAJB5 PE=1 SV=1 - [DNJB5_HUMAN]	9.2	2	2	2	348	39.10911	9.041504	0.896306	0.926389	0.589269	0.8039883	0.210397	0.2799531
Q92556	Engulfment and cell motility protein 1 OS=Homo sapiens GN=ELMO1 PE=1 SV=2 - [ELMO1_HUMAN]	4.54	2	2	2	727	83.77561	6.277832	0.805004	0.799691	0.805694	0.8034628	9.31E-05	0.0195135
P12109	Collagen alpha-1(VI) chain OS=Homo sapiens GN=COL6A1 PE=1 SV=3 - [CO6A1_HUMAN]	40.76	37	37	150	1028	108.462	5.427246	0.800981	0.798097	0.809948	0.8030085	0.000327913	0.0212234
P05543	Thyroxine-binding globulin OS=Homo sapiens GN=SERPINA7 PE=1 SV=2 - [THBG_HUMAN]	6.75	3	3	3	415	46.29461	6.303223	0.818692	0.800913	0.788157	0.8025875	0.00200566	0.0323029
P01767	Ig heavy chain V-III region BUT OS=Homo sapiens PE=1 SV=1 - [HV306_HUMAN]	35.65	3	4	16	115	12.37115	9.246582	0.791695	0.735928	0.872299	0.799974	0.0370018	0.0860832
A0A0A0MSS8	Aldo-keto reductase family 1 member C3 OS=Homo sapiens GN=AKR1C3 PE=1 SV=1 - [A0A0A0MSS8_HUMAN]	21.98	3	8	9	323	36.84386	7.942871	0.82087	0.812341	0.763478	0.7988963	0.00781191	0.045203
D6RG15	Twinfilin-2 OS=Homo sapiens GN=TWF2 PE=1 SV=1 - [D6RG15_HUMAN]	21.65	3	3	4	254	28.86483	6.125488	0.825237	0.7695	0.801896	0.7988778	0.0063946	0.0423724
P16930	Fumarylacetoacetase OS=Homo sapiens GN=FAH PE=1 SV=2 - [FAAA_HUMAN]	7.4	3	3	4	419	46.34421	6.946777	0.767237	0.807217	0.821544	0.7986657	0.00645103	0.0424839

Biology Open • Supplementary information

Q2UY09	Collagen alpha-1(XXVIII) chain OS=Homo sapiens GN=COL28A1 PE=2 SV=2 - [COSA1_HUMAN]	3.91	3	3	3	1125	116.5849	6.404785	0.816802	0.690849	0.88724	0.798297	0.072407	0.129643
Q5JX83	Heat shock 70 kDa protein 12B OS=Homo sapiens GN=HSPA12B PE=1 SV=2 - [Q5JX83_HUMAN]	9.83	5	5	6	600	66.76059	8.865723	0.764686	0.773064	0.856784	0.7981783	0.0205717	0.0626566
B9EG90	Topoisomerase (DNA) I OS=Homo sapiens GN=TOP1 PE=2 SV=1 - [B9EG90_HUMAN]	3.4	2	2	2	765	90.64291	9.305176	0.835896	0.758246	0.795855	0.7966658	0.0119396	0.0495204
B2R6D0	cDNA, FLJ92896, highly similar to Homo sapiens proteasome (prosome, macropain) 26S subunit, non-ATPase, 1 (PSMD1), mRNA OS=Homo sapiens PE=2 SV=1 - [B2R6D0_HUMAN]	15.84	10	10	13	953	105.7707	5.38916	0.84812	0.739402	0.801329	0.7962835	0.0230641	0.0659949
B7Z6C3	cDNA FLJ56151, highly similar to Platelet glycoprotein 4 OS=Homo sapiens PE=2 SV=1 - [B7Z6C3_HUMAN]	8.33	3	3	4	396	44.71791	8.221191	0.809647	0.786287	0.792717	0.7962169	0.00116672	0.0276207
A0A087X1J7	Glutathione peroxidase OS=Homo sapiens GN=GPX3 PE=1 SV=1 - [A0A087X1J7_HUMAN]	35.56	7	7	14	225	25.38595	8.133301	0.786748	0.774371	0.823725	0.7949478	0.00518691	0.0401071
Q96N83	Podocalyxin OS=Homo sapiens PE=2 SV=1 - [Q96N83_HUMAN]	6.37	3	3	5	518	54.54299	5.57959	0.753816	0.79327	0.834282	0.7937891	0.0124537	0.0505179
Q6Y1H2	Very-long-chain (3R)-3-hydroxyacyl-CoA dehydratase 2 OS=Homo sapiens GN=HACD2 PE=1 SV=1 - [HACD2_HUMAN]	7.87	2	2	2	254	28.35014	9.554199	0.770055	0.752218	0.856935	0.7930692	0.0235724	0.06674
A1L377	CABC1 protein OS=Homo sapiens GN=CABC1 PE=2 SV=1 - [A1L377_HUMAN]	7.8	2	2	2	410	44.4497	8.645996	0.777706	0.764162	0.83615	0.7926723	0.0111599	0.0493721
Q4VB86	EPB41 protein OS=Homo sapiens GN=EPB41 PE=1 SV=2 - [Q4VB86_HUMAN]	15.92	6	8	11	622	69.847	6.785645	0.80035	0.826684	0.748695	0.7919094	0.0119007	0.0495204
A8K1J3	cDNA FLJ78534, highly similar to Homo sapiens RAD23 homolog A (S. cerevisiae), mRNA OS=Homo sapiens PE=2 SV=1 - [A8K1J3_HUMAN]	12.98	2	3	4	362	39.44159	4.602051	0.772598	0.851521	0.751595	0.7919047	0.0207059	0.0626973

Biology Open • Supplementary information

E9PHY8	Maestro heat-like repeat-containing protein family member 1 OS=Homo sapiens GN=MROH1 PE=1 SV=1 - [E9PHY8_HUMAN]	1.9	2	2	2	1632	180.1661	6.858887	0.872543	0.728367	0.774483	0.7917977	0.0392514	0.0887548
Q07507	Dermatopontin OS=Homo sapiens GN=DPT PE=1 SV=2 - [DERM_HUMAN]	21.89	4	4	9	201	23.9888	4.817871	0.870063	0.762131	0.74215	0.7914477	0.0344258	0.0818338
Q709C8	Vacuolar protein sorting-associated protein 13C OS=Homo sapiens GN=VPS13C PE=1 SV=1 - [VP13C_HUMAN]	1.92	6	6	6	3753	422.1238	6.82959	0.722867	0.76604	0.881888	0.7902653	0.0476111	0.0994011
O76038	Secretagogin OS=Homo sapiens GN=SCGN PE=2 SV=2 - [SEGN_HUMAN]	10.51	2	2	2	276	32.01911	5.414551	0.842399	0.791317	0.734721	0.7894789	0.021131	0.063152
P02743	Serum amyloid P-component OS=Homo sapiens GN=APCS PE=1 SV=2 - [SAMP_HUMAN]	30.94	8	8	31	223	25.37113	6.536621	0.778862	0.752222	0.837123	0.789402	0.0138756	0.0525379
S6BGD6	IgG L chain OS=Homo sapiens PE=1 SV=1 - [S6BGD6_HUMAN]	45.96	2	10	74	235	24.80813	7.239746	0.746741	0.759319	0.861837	0.7892988	0.0286478	0.0749403
A0A087WVG2	Ras-interacting protein 1 OS=Homo sapiens GN=RASIP1 PE=1 SV=1 - [A0A087WVG2_HUMAN]	5.2	4	4	4	962	103.4682	8.074707	0.760915	0.759008	0.847255	0.7890592	0.0185081	0.0595532
F8WA11	CLIP-associating protein 1 OS=Homo sapiens GN=CLASP1 PE=1 SV=2 - [F8WA11_HUMAN]	2.91	3	3	3	1478	162.6578	8.719238	0.749216	0.810429	0.806839	0.788828	0.00870577	0.0461063
Q05DK5	ADD2 protein (Fragment) OS=Homo sapiens GN=ADD2 PE=2 SV=1 - [Q05DK5_HUMAN]	3.95	3	3	4	709	78.78329	5.630371	0.826901	0.759159	0.778963	0.7883407	0.00890662	0.0463829
P12277	Creatine kinase B-type OS=Homo sapiens GN=CKB PE=1 SV=1 - [KCRB_HUMAN]	42.52	12	12	27	381	42.61732	5.592285	0.871365	0.718748	0.774463	0.7881919	0.041572	0.092095
Q6IQ22	Ras-related protein Rab-12 OS=Homo sapiens GN=RAB12 PE=1 SV=3 - [RAB12_HUMAN]	13.11	2	3	3	244	27.23109	8.411621	0.734881	0.781331	0.846009	0.787407	0.0222119	0.0647174
H0YII4	Protein DDI1 homolog 2 (Fragment) OS=Homo sapiens GN=DDI2 PE=1 SV=1 - [H0YII4_HUMAN]	52.53	4	4	5	99	11.24883	5.097168	0.802202	0.767439	0.791626	0.787089	0.00232694	0.034396

Biology Open • Supplementary information

G5E9C5	Phosphodiesterase 5A, cGMP-specific, isoform CRA_a OS=Homo sapiens GN=PDE5A PE=1 SV=1 - [G5E9C5_HUMAN]	3.04	2	2	2	823	93.57013	6.100098	0.86178	0.861448	0.63758	0.7869362	0.104029	0.1668111
M0QXU7	Mitochondrial import inner membrane translocase subunit TIM44 (Fragment) OS=Homo sapiens GN=TIMM44 PE=1 SV=1 - [M0QXU7_HUMAN]	9.89	2	2	2	273	31.14125	9.480957	0.839254	0.675109	0.842903	0.785755	0.0606942	0.1153342
Q13325	Interferon-induced protein with tetratricopeptide repeats 5 OS=Homo sapiens GN=IFIT5 PE=1 SV=1 - [IFIT5_HUMAN]	6.22	2	2	2	482	55.81161	7.400879	0.818726	0.733786	0.804159	0.7855568	0.0146281	0.0534731
P21397	Amine oxidase [flavin-containing] A OS=Homo sapiens GN=MAOA PE=1 SV=1 - [AOFA_HUMAN]	16.7	5	8	9	527	59.64355	7.85498	0.82322	0.804037	0.727423	0.7848934	0.0180096	0.0587307
C9JJX6	Armadillo repeat protein deleted in velo-cardio-facial syndrome OS=Homo sapiens GN=ARVCF PE=1 SV=1 - [C9JJX6_HUMAN]	4.93	3	3	3	893	96.76859	7.049316	0.945852	0.930701	0.476367	0.784307	0.296394	0.3694012
B4DHN0	Mitogen-activated protein kinase OS=Homo sapiens PE=2 SV=1 - [B4DHN0_HUMAN]	19.54	2	6	8	348	40.39479	7.151855	0.78008	0.771187	0.797289	0.7828519	0.00124253	0.0276882
P04217	Alpha-1B-glycoprotein OS=Homo sapiens GN=A1BG PE=1 SV=4 - [A1BG_HUMAN]	22.63	9	9	16	495	54.21954	5.858887	0.813865	0.715709	0.817343	0.782306	0.0226261	0.065238
H0YI16	PRKC apoptosis WT1 regulator protein (Fragment) OS=Homo sapiens GN=PAWR PE=1 SV=1 - [H0YI16_HUMAN]	22.16	2	2	2	194	20.21415	7.928223	0.827028	0.761308	0.757865	0.7820669	0.0104937	0.0489337
Q6P163	APOC2 protein OS=Homo sapiens GN=APOC2 PE=1 SV=1 - [Q6P163_HUMAN]	27.78	2	2	2	72	8.141222	4.894043	0.789651	0.803564	0.752922	0.7820461	0.004769	0.0400965
B3KM43	cDNA FLJ10228 fis, clone HEMBB1000119, highly similar to N-acetylserotonin O-methyltransferase-like protein OS=Homo sapiens PE=2 SV=1 - [B3KM43_HUMAN]	4.19	2	2	2	621	68.75386	6.125488	0.778561	0.735804	0.831463	0.7819426	0.0157187	0.0550236
H7C3V9	Selenocysteine lyase (Fragment) OS=Homo sapiens GN=SCLY PE=1 SV=1 - [H7C3V9_HUMAN]	12.86	2	2	2	241	26.18124	6.91748	0.832727	0.657329	0.855329	0.781795	0.0733072	0.1304652

B0AZL7	cDNA, FLJ79457, highly similar to Insulin-like growth factor-binding proteincomplex acid labile chain OS=Homo sapiens PE=2 SV=1 - [B0AZL7_HUMAN]	9.09	4	4	5	605	65.95982	6.785645	0.718435	0.875149	0.751757	0.7817803	0.0445463	0.0957392
A0A087WZX2	NADH dehydrogenase [ubiquinone] 1 beta subcomplex subunit 6 OS=Homo sapiens GN=NDUFB6 PE=1 SV=1 - [A0A087WZX2_HUMAN]	26.8	2	2	2	97	11.7081	9.480957	0.857492	0.790969	0.696108	0.7815233	0.0429966	0.093778
Q8N335	Glycerol-3-phosphate dehydrogenase 1-like protein OS=Homo sapiens GN=GPD1L PE=1 SV=1 - [GPD1L_HUMAN]	5.7	2	2	3	351	38.39393	7.02002	0.80955	0.743295	0.789873	0.780906	0.00794388	0.0454224
P35555	Fibrillin-1 OS=Homo sapiens GN=FBN1 PE=1 SV=3 - [FBN1_HUMAN]	33.96	75	81	189	2871	312.0225	4.932129	0.779312	0.78251	0.780453	0.7807584	1.82E-05	0.0123214
A8K8F9	Phosphoinositide phospholipase C OS=Homo sapiens PE=2 SV=1 - [A8K8F9_HUMAN]	8.07	4	4	4	756	85.55097	6.697754	0.782106	0.720994	0.833777	0.7789587	0.0210611	0.063146
A0A087WXL8	Ig gamma-3 chain C region OS=Homo sapiens GN=IGHG3 PE=1 SV=1 - [A0A087WXL8_HUMAN]	37.14	6	14	111	517	56.85294	8.250488	0.809796	0.764185	0.759429	0.7778032	0.00518062	0.0401071
A8K2W3	cDNA FLJ78516 OS=Homo sapiens PE=2 SV=1 - [A8K2W3_HUMAN]	21.18	7	7	15	425	47.07257	5.249512	0.844161	0.752615	0.735588	0.7774546	0.0221881	0.0647174
O75569	Interferon-inducible double-stranded RNA-dependent protein kinase activator A OS=Homo sapiens GN=PRKRA PE=1 SV=1 - [PRKRA_HUMAN]	13.1	3	3	3	313	34.38255	8.411621	0.755235	0.812498	0.764338	0.777357	0.00630714	0.0423147
E7ENL6	Collagen alpha-3(VI) chain OS=Homo sapiens GN=COL6A3 PE=1 SV=2 - [E7ENL6_HUMAN]	43.6	2	106	481	2569	277.9463	8.177246	0.709332	0.808175	0.812658	0.7767217	0.0220553	0.0645265
Q05DL3	CSNK1A1 protein (Fragment) OS=Homo sapiens GN=CSNK1A1 PE=2 SV=1 - [Q05DL3_HUMAN]	10.34	2	2	3	232	26.68497	9.744629	0.781008	0.822651	0.726482	0.7767137	0.0151975	0.0543533
P02042	Hemoglobin subunit delta OS=Homo sapiens GN=HBD PE=1 SV=2 - [HBD_HUMAN]	95.24	8	15	384	147	16.04529	8.04541	0.791178	0.786182	0.75146	0.7762734	0.00310229	0.0360868

Q0ZCF6	Immunglobulin heavy chain variable region (Fragment) OS=Homo sapiens PE=4 SV=1 - [Q0ZCF6_HUMAN]	23.26	2	3	8	129	14.53821	8.777832	0.784605	0.69986	0.844267	0.7762441	0.033316	0.0806036
A5PLK7	RCC2 protein (Fragment) OS=Homo sapiens GN=RCC2 PE=2 SV=1 - [A5PLK7_HUMAN]	10.5	3	3	5	457	49.64713	8.689941	0.738367	0.704982	0.880561	0.7746368	0.0525964	0.1054926
Q5T1M7	60 kDa U4/U6 snRNP-specific spliceosomal protein OS=Homo sapiens GN=PRPF4 PE=2 SV=1 - [Q5T1M7_HUMAN]	5.57	2	2	2	521	58.28434	7.415527	0.813361	0.788026	0.721975	0.7744541	0.0142743	0.0529228
F8W9X7	Coiled-coil domain-containing protein 93 OS=Homo sapiens GN=CCDC93 PE=1 SV=1 - [F8W9X7_HUMAN]	3.02	2	2	2	630	72.9958	7.986816	0.815794	0.717224	0.786305	0.7731075	0.0161727	0.0558618
Q96A33	Coiled-coil domain-containing protein 47 OS=Homo sapiens GN=CCDC47 PE=1 SV=1 - [CCD47_HUMAN]	4.14	2	2	2	483	55.83831	4.868652	0.740617	0.782781	0.794951	0.7727832	0.00520841	0.0401071
B4DQT8	cDNA FLJ61158, highly similar to ADP-ribosylation factor-like protein 8B OS=Homo sapiens PE=2 SV=1 - [B4DQT8_HUMAN]	22.6	2	3	6	177	20.58457	7.781738	0.759909	0.868783	0.688217	0.7723031	0.049253	0.1016594
O15031	Plexin-B2 OS=Homo sapiens GN=PLXNB2 PE=1 SV=3 - [PLXB2_HUMAN]	3.92	5	6	6	1838	204.9972	6.239746	0.764751	0.736921	0.81359	0.7717538	0.00950089	0.0472949
Q6NUQ2	Calmin (Calponin-like, transmembrane) OS=Homo sapiens GN=CLMN PE=2 SV=1 - [Q6NUQ2_HUMAN]	2.3	2	2	2	1002	111.571	4.932129	0.761183	0.814806	0.738514	0.7715009	0.009658	0.047762
B3VL31	Beta globin (Fragment) OS=Homo sapiens PE=3 SV=1 - [B3VL31_HUMAN]	93.33	2	12	493	105	11.49185	6.683105	0.818523	0.739245	0.756242	0.7713364	0.0109246	0.0493157
E9PC52	Histone-binding protein RBBP7 OS=Homo sapiens GN=RBBP7 PE=1 SV=1 - [E9PC52_HUMAN]	11.06	2	3	3	416	46.90879	5.071777	0.743231	0.794413	0.774692	0.7707786	0.00420094	0.0391996
D6RFL4	Monocyte differentiation antigen CD14 (Fragment) OS=Homo sapiens GN=CD14 PE=1 SV=1 - [D6RFL4_HUMAN]	32.39	5	5	6	213	23.29813	5.262207	0.825792	0.712347	0.774071	0.7707366	0.0198502	0.061532
Q9HAV0	Guanine nucleotide-binding protein subunit beta-4 OS=Homo sapiens GN=GNB4 PE=1 SV=3 - [GBB4_HUMAN]	15.59	2	5	8	340	37.54302	5.998535	0.750138	0.712229	0.849654	0.7706738	0.0304783	0.0772223

Biology Open • Supplementary information

Q8WUW1	Protein BRICK1 OS=Homo sapiens GN=BRK1 PE=1 SV=1 - [BRK1_HUMAN]	25.33	2	2	2	75	8.739464	5.452637	0.806862	0.769107	0.734077	0.7700152	0.00824721	0.0456887
B3KSV9	cDNA FLJ37148 fis, clone BRACE2025333, highly similar to Homo sapiens Na+/H+ exchange regulatory co-factor (NHERF) mRNA OS=Homo sapiens PE=2 SV=1 - [B3KSV9_HUMAN]	8.79	2	2	2	273	29.4191	6.800293	0.844866	0.679386	0.785316	0.7698563	0.0414803	0.0920252
G4V2I8	Anion exchange protein OS=Homo sapiens PE=2 SV=1 - [G4V2I8_HUMAN]	21.51	16	16	36	911	101.6834	5.211426	0.811557	0.764721	0.730997	0.7690916	0.0100785	0.0483662
Q96Q06	Perilipin-4 OS=Homo sapiens GN=PLIN4 PE=1 SV=2 - [PLIN4_HUMAN]	29.26	10	23	28	1357	134.3489	8.733887	0.776122	0.77155	0.756008	0.7678933	0.000687165	0.0241782
A9XXE1	Gamma-synuclein OS=Homo sapiens GN=SNCG PE=2 SV=1 - [A9XXE1_HUMAN]	63.29	3	3	6	79	8.319126	4.525879	0.816675	0.718478	0.76845	0.7678678	0.0145883	0.0534431
A0A0J9YXM6	WD repeat-containing protein 81 OS=Homo sapiens GN=WDR81 PE=1 SV=1 - [A0A0J9YXM6_HUMAN]	1.86	3	3	3	1940	211.4352	5.60498	0.870935	0.604529	0.827745	0.7677363	0.106517	0.1693565
P01611	Ig kappa chain V-I region Wes OS=Homo sapiens PE=1 SV=1 - [KV119_HUMAN]	31.48	2	2	4	108	11.60071	7.283691	0.843155	0.641203	0.816727	0.7670282	0.0666796	0.1220716
O94911	ATP-binding cassette sub-family A member 8 OS=Homo sapiens GN=ABCA8 PE=1 SV=3 - [ABCA8_HUMAN]	5	6	6	8	1581	179.1296	7.181152	0.81226	0.733782	0.754341	0.7667942	0.00999793	0.0482782
B3KY04	cDNA FLJ46506 fis, clone THYMU3030752, highly similar to BTB/POZ domain-containing protein KCTD12 OS=Homo sapiens PE=2 SV=1 - [B3KY04_HUMAN]	24.31	7	7	15	325	35.7087	5.643066	0.797635	0.711401	0.789825	0.7662868	0.0135986	0.0522489
P62191	26S protease regulatory subunit 4 OS=Homo sapiens GN=PSMC1 PE=1 SV=1 - [PRS4_HUMAN]	7.95	3	3	3	440	49.1537	6.214355	0.821126	0.652622	0.823723	0.7658241	0.0537616	0.1069859
A6NHR9	Structural maintenance of chromosomes flexible hinge domain-containing protein 1 OS=Homo sapiens GN=SMCHD1 PE=1 SV=2 - [SMHD1_HUMAN]	1.45	2	2	2	2005	226.2311	7.29834	0.734948	0.763072	0.798643	0.7655543	0.00612224	0.0421458

Q12882	Dihydropyrimidine dehydrogenase [NADP(+)] OS=Homo sapiens GN=DPYD PE=1 SV=2 - [DPYD_HUMAN]	2.54	2	2	2	1025	111.3293	7.049316	0.706147	0.658526	0.930068	0.7649134	0.106861	0.1698237
C9J9J4	55 kDa erythrocyte membrane protein (Fragment) OS=Homo sapiens GN=MPP1 PE=1 SV=1 - [C9J9J4_HUMAN]	30.24	5	5	7	205	23.04176	8.821777	0.781275	0.688043	0.825338	0.7648852	0.0283761	0.0745175
Q9BT22	Chitobiosyldiphosphodolichol beta-mannosyltransferase OS=Homo sapiens GN=ALG1 PE=1 SV=2 - [ALG1_HUMAN]	4.53	2	2	2	464	52.48394	7.225098	0.843864	0.64494	0.805423	0.7647427	0.0609861	0.1155868
Q9BXN1	Asporin OS=Homo sapiens GN=ASPN PE=1 SV=2 - [ASPN_HUMAN]	35	13	13	29	380	43.38932	7.078613	0.774287	0.781	0.737962	0.764416	0.00320459	0.0364914
Q96LD4	Tripartite motif-containing protein 47 OS=Homo sapiens GN=TRIM47 PE=1 SV=2 - [TRI47_HUMAN]	7.84	4	4	5	638	69.48788	6.442871	0.776951	0.752255	0.763979	0.764395	0.00091509	0.02539
P00748	Coagulation factor XII OS=Homo sapiens GN=F12 PE=1 SV=3 - [FA12_HUMAN]	4.55	2	2	2	615	67.7479	7.737793	0.758665	0.787543	0.742725	0.7629774	0.0030482	0.0360868
O60240	Perilipin-1 OS=Homo sapiens GN=PLIN1 PE=1 SV=2 - [PLIN1_HUMAN]	32.38	13	13	21	522	55.9558	6.41748	0.788584	0.743091	0.757071	0.7629153	0.00320478	0.0364914
B7Z7F5	cDNA FLJ51581 OS=Homo sapiens PE=2 SV=1 - [B7Z7F5_HUMAN]	8.6	2	2	3	279	30.74581	8.909668	0.78207	0.775946	0.727144	0.76172	0.00527708	0.0401071
E9PNJ4	Stromal interaction molecule 1 OS=Homo sapiens GN=STIM1 PE=1 SV=1 - [E9PNJ4_HUMAN]	3.91	2	2	2	512	57.58471	8.309082	0.795436	0.770871	0.717956	0.7614212	0.00905662	0.04652
Q9UBQ0	Vacuolar protein sorting-associated protein 29 OS=Homo sapiens GN=VPS29 PE=1 SV=1 - [VPS29_HUMAN]	20.33	4	4	4	182	20.49268	6.785645	0.705762	0.726038	0.848673	0.7601578	0.0329437	0.0802262
P51884	Lumican OS=Homo sapiens GN=LUM PE=1 SV=2 - [LUM_HUMAN]	43.2	14	14	93	338	38.4048	6.609863	0.752432	0.759251	0.768746	0.7601428	0.000388729	0.0212234
B3VL17	Beta globin (Fragment) OS=Homo sapiens PE=3 SV=1 - [B3VL17_HUMAN]	93.33	4	14	513	105	11.47477	6.366699	0.760358	0.769321	0.749437	0.7597052	0.000571957	0.0241782
Q53FL4	Adducin 3 isoform a variant (Fragment) OS=Homo sapiens PE=2 SV=1 - [Q53FL4_HUMAN]	6.3	3	3	4	667	74.43394	6.507324	0.776333	0.716067	0.786478	0.7596258	0.00825467	0.0456887

B7Z4L7	cDNA FLJ59035, highly similar to RNA-binding region-containing protein 2 OS=Homo sapiens PE=2 SV=1 - [B7Z4L7_HUMAN]	14.54	3	3	3	337	36.44143	5.731934	0.752959	0.75553	0.767486	0.7586584	0.000343731	0.0212234
B2R6C0	Glycerol-3-phosphate dehydrogenase [NAD(+)] OS=Homo sapiens PE=2 SV=1 - [B2R6C0_HUMAN]	5.44	2	2	3	349	37.57529	6.17627	0.717935	0.871928	0.685267	0.7583766	0.052324	0.1051762
O00468	Agrin OS=Homo sapiens GN=AGRN PE=1 SV=5 - [AGRIN_HUMAN]	21.53	27	27	38	2067	217.0917	6.39209	0.800607	0.712718	0.759447	0.757591	0.0107918	0.0492111
B4E1B3	cDNA FLJ53950, highly similar to Angiotensinogen OS=Homo sapiens PE=2 SV=1 - [B4E1B3_HUMAN]	15.67	6	6	10	466	51.03042	6.163574	0.840035	0.721765	0.710507	0.7574358	0.0279514	0.0740148
P00326	Alcohol dehydrogenase 1C OS=Homo sapiens GN=ADH1C PE=1 SV=2 - [ADH1G_HUMAN]	30.93	3	14	27	375	39.84168	8.294434	0.743645	0.767409	0.757149	0.7560676	0.000794862	0.0242397
G9FP35	Guanine nucleotide binding protein OS=Homo sapiens GN=GNAQ PE=2 SV=1 - [G9FP35_HUMAN]	18.94	3	7	10	359	42.08431	5.681152	0.852626	0.719549	0.695809	0.7559947	0.0377468	0.0869204
B3KXS3	cDNA FLJ45947 fis, clone PLACE7007379, highly similar to Homo sapiens carboxypeptidase Z (CPZ), transcript variant 2, mRNA OS=Homo sapiens PE=2 SV=1 - [B3KXS3_HUMAN]	11.54	2	2	2	260	29.35009	9.612793	0.699491	0.650012	0.913079	0.7541939	0.0930272	0.154213
B3KSI7	cDNA FLJ36374 fis, clone THYMU2008185, highly similar to Xaa-Pro aminopeptidase 1 (EC 3.4.11.9) OS=Homo sapiens PE=2 SV=1 - [B3KSI7_HUMAN]	13.22	5	5	8	552	62.10948	5.731934	0.782748	0.714045	0.763959	0.7535842	0.00684991	0.0433728
P04004	Vitronectin OS=Homo sapiens GN=VTN PE=1 SV=1 - [VTNC_HUMAN]	23.22	10	10	33	478	54.27117	5.79541	0.774609	0.705118	0.772243	0.7506568	0.00824316	0.0456887
P59768	Guanine nucleotide-binding protein G(I)/G(S)/G(O) subunit gamma-2 OS=Homo sapiens GN=GNG2 PE=1 SV=2 - [GBG2_HUMAN]	52.11	3	3	4	71	7.845034	7.986816	0.803617	0.755097	0.692327	0.750347	0.0162455	0.055999
P02549	Spectrin alpha chain, erythrocytic 1 OS=Homo sapiens GN=SPTA1 PE=1 SV=5 - [SPTA1_HUMAN]	20.5	37	37	45	2419	279.8419	5.046387	0.779597	0.716873	0.753315	0.7499282	0.00524693	0.0401071

P02760	Protein AMBP OS=Homo sapiens GN=AMBP PE=1 SV=1 - [AMBP_HUMAN]	29.26	8	8	17	352	38.97398	6.252441	0.782983	0.734488	0.727097	0.7481897	0.00480989	0.0401071
Q9BTW9	Tubulin-specific chaperone D OS=Homo sapiens GN=TBCD PE=1 SV=2 - [TBCD_HUMAN]	2.94	2	2	2	1192	132.5153	6.188965	0.749854	0.785702	0.708381	0.7479793	0.00776642	0.045203
M0QZ21	AP-2 complex subunit sigma OS=Homo sapiens GN=AP2S1 PE=1 SV=1 - [M0QZ21_HUMAN]	16.39	2	2	2	122	14.54729	4.995605	0.78305	0.776121	0.682031	0.7470673	0.0161897	0.0558635
Q02252	Methylmalonate-semialdehyde dehydrogenase [acylating], mitochondrial OS=Homo sapiens GN=ALDH6A1 PE=1 SV=2 - [MMSA_HUMAN]	5.23	2	2	2	535	57.80266	8.499512	0.778472	0.743014	0.717616	0.7463672	0.00480639	0.0401071
Q7Z7R0	Smooth muscle myosin heavy chain (Fragment) OS=Homo sapiens PE=2 SV=1 - [Q7Z7R0_HUMAN]	64.26	2	84	276	1052	121.8828	5.046387	0.719168	0.744377	0.773872	0.7458056	0.00384509	0.0387187
B4DYP5	cDNA FLJ54725, highly similar to Ankyrin repeat domain-containing protein 13 OS=Homo sapiens PE=2 SV=1 - [B4DYP5_HUMAN]	4.24	2	2	2	589	67.43373	5.046387	0.851447	0.705451	0.677962	0.7449534	0.0417817	0.0923402
A0A087WSY5	Carboxypeptidase B2 OS=Homo sapiens GN=CPB2 PE=1 SV=1 - [A0A087WSY5_HUMAN]	4.92	2	2	2	386	43.9942	7.708496	0.747246	0.758164	0.72861	0.7446731	0.00113988	0.0276207
P20774	Mimecan OS=Homo sapiens GN=OGN PE=1 SV=1 - [MIME_HUMAN]	28.86	13	13	32	298	33.90089	5.630371	0.748618	0.753356	0.731257	0.7444101	0.000689989	0.0241782
A0A087WU91	Protein IGHV3-49 OS=Homo sapiens GN=IGHV3-49 PE=1 SV=1 - [A0A087WU91_HUMAN]	17.54	2	2	5	114	12.32416	8.924316	0.739527	0.775688	0.715117	0.7434443	0.00467036	0.0398629
P01612	Ig kappa chain V-I region Mev OS=Homo sapiens PE=1 SV=1 - [KV120_HUMAN]	31.19	2	2	4	109	11.86279	6.565918	0.804074	0.650385	0.775853	0.7434372	0.0322627	0.0797133
Q7Z3Z9	L1 cell adhesion molecule (Fragment) OS=Homo sapiens GN=L1CAM PE=2 SV=1 - [Q7Z3Z9_HUMAN]	4.14	5	5	7	1255	139.6852	6.239746	0.797449	0.691489	0.734223	0.7410538	0.0138349	0.0525187
A0A087WU43	Cadherin-1 OS=Homo sapiens GN=CDH1 PE=1 SV=1 - [A0A087WU43_HUMAN]	6.34	3	3	5	647	71.23988	4.944824	0.699951	0.704184	0.818705	0.7409466	0.021812	0.0640917

Biology Open • Supplementary information

H0Y742	SUN domain-containing protein 1 (Fragment) OS=Homo sapiens GN=SUN1 PE=1 SV=1 - [H0Y742_HUMAN]	11.13	6	6	6	710	79.54032	7.093262	0.774487	0.67446	0.772319	0.740422	0.0157681	0.0550258
B4DPB6	cDNA FLJ56537, highly similar to Coiled-coil domain-containing protein 22 OS=Homo sapiens PE=2 SV=1 - [B4DPB6_HUMAN]	7.59	3	3	3	580	65.11631	7.225098	0.719921	0.713806	0.787167	0.7402978	0.0080895	0.0456383
Q9P0M6	Core histone macro-H2A.2 OS=Homo sapiens GN=H2AFY2 PE=1 SV=3 - [H2AW_HUMAN]	10.75	2	3	4	372	40.03337	9.686035	0.688221	0.786694	0.74338	0.7394315	0.011749	0.0494101
Q9BXX0	EMILIN-2 OS=Homo sapiens GN=EMILIN2 PE=1 SV=3 - [EMIL2_HUMAN]	2.85	2	2	2	1053	115.6149	6.455566	0.759008	0.713461	0.742038	0.7381688	0.00256641	0.0357839
B4DIH5	COP9 signalosome complex subunit 2 OS=Homo sapiens GN=COPS2 PE=1 SV=1 - [B4DIH5_HUMAN]	12.66	5	5	5	379	44.2004	5.871582	0.783127	0.740832	0.689662	0.7378735	0.0104601	0.0489337
P01034	Cystatin-C OS=Homo sapiens GN=CST3 PE=1 SV=1 - [CYTC_HUMAN]	11.64	2	2	2	146	15.78908	8.748535	0.755813	0.698664	0.757928	0.7374683	0.00542279	0.0402547
J3KT17	Galectin OS=Homo sapiens GN=LGALS9C PE=4 SV=1 - [J3KT17_HUMAN]	11.11	2	2	3	234	25.6055	8.382324	0.778506	0.680288	0.749835	0.7362096	0.0120002	0.0495979
P51888	Prolargin OS=Homo sapiens GN=PRELP PE=1 SV=1 - [PRELP_HUMAN]	44.5	14	14	44	382	43.78216	9.378418	0.717598	0.747219	0.742268	0.735695	0.00119909	0.0276207
Q6VFQ6	Hemoglobin beta chain (Fragment) OS=Homo sapiens GN=HBB PE=3 SV=1 - [Q6VFQ6_HUMAN]	100	2	5	140	42	4.51041	8.23584	0.756358	0.725412	0.724564	0.7354448	0.00155942	0.0294896
Q9ULA6	Uncharacterized protein (Fragment) OS=Homo sapiens PE=4 SV=1 - [Q9ULA6_HUMAN]	9.41	2	2	2	287	33.44293	9.305176	0.774861	0.667986	0.752511	0.7317859	0.0144069	0.0531235
B4DLL8	cDNA FLJ59335, highly similar to Transmembrane glycoprotein NMB OS=Homo sapiens PE=2 SV=1 - [B4DLL8_HUMAN]	5.93	2	2	5	455	50.54749	6.239746	0.712723	0.80047	0.679832	0.7310083	0.017449	0.05785
B2RMN7	Spectrin, beta, erythrocytic OS=Homo sapiens GN=SPTB PE=2 SV=1 - [B2RMN7_HUMAN]	24.05	38	41	61	2137	246.4158	5.274902	0.73733	0.728643	0.725487	0.7304867	0.000172569	0.0212234

Biology Open • Supplementary information

P02649	Apolipoprotein E OS=Homo sapiens GN=APOE PE=1 SV=1 - [APOE_HUMAN]	42.59	11	11	16	317	36.13176	5.731934	0.736097	0.725794	0.725933	0.7292746	0.000158745	0.0212234
P42025	Beta-centractin OS=Homo sapiens GN=ACTR1B PE=1 SV=1 - [ACTY_HUMAN]	17.02	2	5	6	376	42.26673	6.404785	0.69757	0.740666	0.748356	0.7288639	0.00338008	0.0370277
Q9Y5Y7	Lymphatic vessel endothelial hyaluronic acid receptor 1 OS=Homo sapiens GN=LYVE1 PE=1 SV=2 - [LYVE1_HUMAN]	5.59	2	2	3	322	35.19096	8.279785	0.678827	0.733151	0.773634	0.7285372	0.0100819	0.0483662
Q59FF1	Insulin-like growth factor binding protein 2 variant (Fragment) OS=Homo sapiens PE=2 SV=1 - [Q59FF1_HUMAN]	11.99	2	2	2	292	30.28428	8.382324	0.774464	0.658998	0.751757	0.7284063	0.016493	0.0565435
B7Z4C3	cDNA FLJ50805, highly similar to Erythrocyte membrane protein band 4.2 OS=Homo sapiens PE=2 SV=1 - [B7Z4C3_HUMAN]	8.39	5	5	7	691	76.98874	8.191895	0.71994	0.719944	0.741012	0.7269657	0.000661038	0.0241782
Q03169	Tumor necrosis factor alpha-induced protein 2 OS=Homo sapiens GN=TNFAIP2 PE=2 SV=2 - [TNAP2_HUMAN]	10.7	5	5	5	654	72.61612	6.455566	0.719024	0.712417	0.74643	0.7259572	0.00144056	0.0287984
Q96PE7	Methylmalonyl-CoA epimerase, mitochondrial OS=Homo sapiens GN=MCEE PE=1 SV=1 - [MCEE_HUMAN]	12.5	2	2	3	176	18.73707	9.085449	0.740334	0.667414	0.766752	0.7248332	0.0114542	0.0494101
P39900	Macrophage metalloelastase OS=Homo sapiens GN=MMP12 PE=1 SV=1 - [MMP12_HUMAN]	12.77	5	5	5	470	53.96715	8.660645	0.789214	0.718306	0.665859	0.7244599	0.0164133	0.0563479
Q6UWY5	Olfactomedin-like protein 1 OS=Homo sapiens GN=OLFML1 PE=1 SV=2 - [OLFL1_HUMAN]	21.39	8	8	12	402	45.92136	8.089355	0.694565	0.729119	0.745736	0.7231397	0.00295022	0.0360523
P15090	Fatty acid-binding protein, adipocyte OS=Homo sapiens GN=FABP4 PE=1 SV=3 - [FABP4_HUMAN]	15.91	2	2	4	132	14.7095	7.137207	0.74177	0.74031	0.682058	0.7213795	0.00494468	0.0401071
Q9NTJ5	Phosphatidylinositide phosphatase SAC1 OS=Homo sapiens GN=SACM1L PE=1 SV=2 - [SAC1_HUMAN]	3.41	2	2	2	587	66.92401	7.122559	0.748322	0.720954	0.687865	0.719047	0.00384784	0.0387187
P16157	Ankyrin-1 OS=Homo sapiens GN=ANK1 PE=1 SV=3 - [ANK1_HUMAN]	20.57	27	28	37	1881	206.1369	6.01123	0.702304	0.701398	0.750152	0.7179512	0.00324357	0.036522

Biology Open • Supplementary information

Q96B43	Methionine aminopeptidase (Fragment) OS=Homo sapiens PE=2 SV=1 - [Q96B43_HUMAN]	13.83	2	2	2	253	28.21199	6.112793	0.704991	0.71306	0.73257	0.7168737	0.000835029	0.0247945
A8K7A4	cDNA FLJ76904, highly similar to Homo sapiens methionine adenosyltransferase II, beta (MAT2B), transcript variant 2, mRNA OS=Homo sapiens PE=2 SV=1 - [A8K7A4_HUMAN]	11.46	2	2	3	323	36.40644	6.800293	0.672096	0.712387	0.765801	0.7167613	0.00905598	0.04652
P22105	Tenascin-X OS=Homo sapiens GN=TNXB PE=1 SV=4 - [TENX_HUMAN]	21.31	32	49	73	4242	457.9321	5.17334	0.721481	0.681267	0.742749	0.7151656	0.00398166	0.0390309
A8K6X2	cDNA FLJ77992, highly similar to Homo sapiens ADP-ribosylarginine hydrolase, mRNA OS=Homo sapiens PE=2 SV=1 - [A8K6X2_HUMAN]	8.4	2	2	2	357	39.45134	6.521973	0.724692	0.712139	0.708218	0.7150162	0.000303817	0.0212234
P63096	Guanine nucleotide-binding protein G(i) subunit alpha-1 OS=Homo sapiens GN=GNAI1 PE=1 SV=2 - [GNAI1_HUMAN]	20.34	2	7	16	354	40.33523	5.973145	0.748263	0.604296	0.784295	0.7122849	0.0346388	0.082167
P01613	Ig kappa chain V-I region Ni OS=Homo sapiens PE=1 SV=1 - [KV121_HUMAN]	30.36	2	2	3	112	12.23801	5.36377	0.527366	0.461311	1.14399	0.710889	0.314935	0.3876213
Q8WWI5	Choline transporter-like protein 1 OS=Homo sapiens GN=SLC44A1 PE=1 SV=1 - [CTL1_HUMAN]	4.11	2	2	3	657	73.25327	8.602051	0.686221	0.680225	0.766015	0.7108203	0.00901966	0.04652
Q9BSD7	Cancer-related nucleoside-triphosphatase OS=Homo sapiens GN=NTPCR PE=1 SV=1 - [NTPCR_HUMAN]	15.79	2	2	2	190	20.70011	9.539551	0.753857	0.659246	0.715712	0.7096052	0.00883754	0.046376
P02452	Collagen alpha-1(I) chain OS=Homo sapiens GN=COL1A1 PE=1 SV=5 - [CO1A1_HUMAN]	24.25	11	30	125	1464	138.8566	5.79541	0.712656	0.680547	0.731851	0.7083512	0.00262279	0.0357839
B4E2M1	cDNA FLJ58478, highly similar to Glypican-6 OS=Homo sapiens PE=2 SV=1 - [B4E2M1_HUMAN]	11.68	2	3	5	428	48.52529	6.844238	0.758114	0.668286	0.696083	0.7074942	0.00813901	0.0456419
P10645	Chromogranin-A OS=Homo sapiens GN=CHGA PE=1 SV=7 - [CMGA_HUMAN]	3.94	2	2	2	457	50.65766	4.602051	0.73585	0.683013	0.695584	0.7048158	0.00290178	0.0360252

Biology Open • Supplementary information

B2RUU3	Dedicator of cytokinesis 1 OS=Homo sapiens GN=DOCK1 PE=2 SV=1 - [B2RUU3_HUMAN]	1.5	2	2	2	1865	215.24	7.48877	0.712108	0.708608	0.686862	0.702526	0.000703944	0.0242397
P02461	Collagen alpha-1(III) chain OS=Homo sapiens GN=COL3A1 PE=1 SV=4 - [CO3A1_HUMAN]	12.35	12	13	26	1466	138.479	6.609863	0.720076	0.676947	0.705239	0.700754	0.00178242	0.0311259
B4DSH1	cDNA FLJ51295, highly similar to Cell division cycle 5-like protein OS=Homo sapiens PE=2 SV=1 - [B4DSH1_HUMAN]	4.13	2	2	2	775	89.17118	8.543457	0.739367	0.744055	0.616543	0.6999884	0.0188161	0.0601507
H7BXR3	Sorbin and SH3 domain-containing protein 2 (Fragment) OS=Homo sapiens GN=SORBS2 PE=1 SV=1 - [H7BXR3_HUMAN]	36.16	3	20	40	636	70.1323	9.598145	0.747708	0.627774	0.720192	0.6985582	0.0141721	0.0528571
B3KVN0	cDNA FLJ16785 fis, clone NT2RI2015342, highly similar to Solute carrier family 2, facilitated glucose transporter member 1 OS=Homo sapiens PE=2 SV=1 - [B3KVN0_HUMAN]	13.46	5	5	7	416	45.83925	8.602051	0.643582	0.731536	0.716377	0.6971649	0.00793993	0.0454224
E9PHS0	LanC-like protein 1 (Fragment) OS=Homo sapiens GN=LANCL1 PE=1 SV=1 - [E9PHS0_HUMAN]	18.37	3	3	4	196	22.02824	8.147949	0.693576	0.578808	0.818212	0.6968651	0.0482717	0.1002452
H0YGS3	Microfibrillar-associated protein 5 (Fragment) OS=Homo sapiens GN=MFAP5 PE=1 SV=1 - [H0YGS3_HUMAN]	39.24	3	3	7	79	9.436816	9.466309	0.69405	0.679008	0.694653	0.6892371	0.000271082	0.0212234
P00325	Alcohol dehydrogenase 1B OS=Homo sapiens GN=ADH1B PE=1 SV=2 - [ADH1B_HUMAN]	48	8	19	47	375	39.8286	8.294434	0.67682	0.71899	0.665337	0.687049	0.0027055	0.0357839
B3KME0	Protein argonaute OS=Homo sapiens PE=2 SV=1 - [B3KME0_HUMAN]	6.14	2	4	6	782	88.66266	9.202637	0.680444	0.642748	0.732505	0.6852323	0.0067645	0.0433606
Q9Y6M1	Insulin-like growth factor 2 mRNA-binding protein 2 OS=Homo sapiens GN=IGF2BP2 PE=1 SV=2 - [IF2B2_HUMAN]	5.01	2	2	2	599	66.08062	8.455566	0.769705	0.574443	0.710623	0.6849236	0.0320584	0.0795998
F5H4Z8	Thrombospondin-3 OS=Homo sapiens GN=THBS3 PE=1 SV=2 - [F5H4Z8_HUMAN]	3.17	2	2	2	947	103.186	4.640137	0.749921	0.604022	0.700617	0.6848535	0.0179888	0.0587195
O43670	BUB3-interacting and GLEBS motif-containing protein ZNF207 OS=Homo sapiens GN=ZNF207 PE=1 SV=1 -	5.44	2	2	2	478	50.71715	9.100098	0.714341	0.591878	0.743752	0.6833235	0.0208919	0.0628695

	[ZN207_HUMAN]													
V9GYZ1	Integrin beta (Fragment) OS=Homo sapiens GN=ITGB5 PE=1 SV=1 - [V9GYZ1_HUMAN]	12.95	2	2	2	139	16.12312	7.137207	0.668524	0.671331	0.704656	0.6815037	0.0013249	0.0283421
B4DVF1	cDNA FLJ51111, highly similar to Aldehyde oxidase (EC 1.2.3.1) (Fragment) OS=Homo sapiens PE=2 SV=1 - [B4DVF1_HUMAN]	3.44	2	2	2	785	87.31029	6.961426	0.718791	0.65921	0.663236	0.6804126	0.00359903	0.0379524
B4DRU9	cDNA FLJ57179, highly similar to Homo sapiens ATP-binding cassette, sub-family F (GCN20), member 3 (ABCF3), mRNA OS=Homo sapiens PE=2 SV=1 - [B4DRU9_HUMAN]	3.59	2	2	2	668	75.52714	7.181152	0.670389	0.637072	0.722486	0.6766491	0.00585665	0.0415613
A0A087WTA8	Collagen alpha-2(I) chain OS=Homo sapiens GN=COL1A2 PE=1 SV=1 - [A0A087WTA8_HUMAN]	18.99	20	20	80	1364	129.0724	9.012207	0.710533	0.632985	0.683587	0.6757018	0.00487704	0.0401071
Q68CX8	Putative uncharacterized protein DKFZp686P12272 OS=Homo sapiens GN=DKFZp686P12272 PE=4 SV=2 - [Q68CX8_HUMAN]	4	2	2	2	375	41.55982	8.265137	0.666651	0.627774	0.727325	0.6739166	0.00779895	0.045203
B3KRR1	cDNA FLJ34725 fis, clone MESAN2005958, highly similar to RNA-binding protein Luc7-like 2 OS=Homo sapiens PE=2 SV=1 - [B3KRR1_HUMAN]	9.44	2	3	3	339	40.46676	10.14014	0.684751	0.692444	0.644114	0.67377	0.00210558	0.0331506
B4DZF1	cDNA FLJ56541, highly similar to Ubiquilin-2 OS=Homo sapiens PE=2 SV=1 - [B4DZF1_HUMAN]	6.45	2	2	2	512	54.96508	5.554199	0.730818	0.557753	0.724935	0.6711688	0.0285003	0.0747857
Q96G03	Phosphoglucomutase-2 OS=Homo sapiens GN=PGM2 PE=1 SV=4 - [PGM2_HUMAN]	9.48	4	5	5	612	68.2404	6.727051	0.728901	0.642273	0.642026	0.6710668	0.00764006	0.0449563
H0YKE1	GMP reductase 2 (Fragment) OS=Homo sapiens GN=GMPR2 PE=1 SV=1 - [H0YKE1_HUMAN]	60.94	2	2	2	64	6.954276	5.782715	0.620255	0.72938	0.640573	0.6634026	0.00976393	0.0478721
E9PHN7	Glutathione S-transferase Mu 2 OS=Homo sapiens GN=GSTM2 PE=1 SV=2 - [E9PHN7_HUMAN]	37.57	5	6	8	181	21.34882	8.32373	0.653938	0.697577	0.636117	0.662544	0.00291407	0.0360252

Biology Open • Supplementary information

D6RD17	Immunoglobulin J chain (Fragment) OS=Homo sapiens GN=JCHAIN PE=1 SV=6 - [D6RD17_HUMAN]	21.66	3	3	6	157	18.009	5.60498	0.657085	0.672122	0.65796	0.6623886	0.000208276	0.0212234
B4DRW6	Alpha-1,4 glucan phosphorylase OS=Homo sapiens PE=2 SV=1 - [B4DRW6_HUMAN]	15.14	5	9	9	832	95.84319	7.913574	0.674307	0.657047	0.653976	0.6617766	0.000349843	0.0212234
V9HW11	Epididymis secretory sperm binding protein Li 83p OS=Homo sapiens GN=HEL-S-83p PE=2 SV=1 - [V9HW11_HUMAN]	5.64	2	2	3	461	51.11395	8.719238	0.650274	0.602536	0.730506	0.6611056	0.0119213	0.0495204
Q6FHW3	DF protein OS=Homo sapiens GN=DF PE=2 SV=1 - [Q6FHW3_HUMAN]	35.96	5	5	6	228	24.40741	7.239746	0.669891	0.627931	0.681768	0.6598638	0.00229708	0.0342495
O60844	Zymogen granule membrane protein 16 OS=Homo sapiens GN=ZG16 PE=1 SV=2 - [ZG16_HUMAN]	16.17	2	2	2	167	18.13529	9.36377	0.673265	0.641134	0.661159	0.6585191	0.000751882	0.0242397
Q9H9B4	Sideroflexin-1 OS=Homo sapiens GN=SFXN1 PE=1 SV=4 - [SFXN1_HUMAN]	6.83	2	2	3	322	35.59635	9.070801	0.59367	0.537164	0.837431	0.6560882	0.0648563	0.1201788
Q9BRZ2	E3 ubiquitin-protein ligase TRIM56 OS=Homo sapiens GN=TRIM56 PE=1 SV=3 - [TRI56_HUMAN]	3.71	2	2	2	755	81.43651	7.737793	0.584672	0.648351	0.726871	0.653298	0.0137792	0.0524074
A0A075B785	LisH domain and HEAT repeat-containing protein KIAA1468 OS=Homo sapiens GN=KIAA1468 PE=1 SV=5 - [A0A075B785_HUMAN]	2.36	2	2	3	1017	112.643	5.490723	0.657543	0.566083	0.732431	0.6520188	0.0185755	0.0597133
Q04828	Aldo-keto reductase family 1 member C1 OS=Homo sapiens GN=AKR1C1 PE=1 SV=1 - [AK1C1_HUMAN]	21.36	2	7	8	323	36.76502	7.884277	0.613483	0.613893	0.727573	0.6516496	0.0116684	0.0494101
B3KV06	Matrix metalloproteinase-28 OS=Homo sapiens GN=MMP28 PE=1 SV=1 - [B3KV06_HUMAN]	7.25	3	3	3	510	57.58252	9.64209	0.629298	0.562908	0.748513	0.6469065	0.0228407	0.0654664
A5D8Z7	SIGLEC1 protein (Fragment) OS=Homo sapiens GN=SIGLEC1 PE=2 SV=1 - [A5D8Z7_HUMAN]	1.48	2	2	2	1688	180.3338	6.63916	0.688977	0.576213	0.671883	0.6456907	0.00966528	0.047762
D3DQV2	Guanine nucleotide-binding protein subunit gamma OS=Homo sapiens GN=hCG_1992840 PE=3 SV=1 - [D3DQV2_HUMAN]	26.47	2	2	2	68	7.15766	6.442871	0.641092	0.690762	0.591741	0.6411983	0.00628714	0.0423147

Biology Open • Supplementary information

A8K8W7	cDNA FLJ75224, highly similar to Homo sapiens lipase, hormone-sensitive (LIPE), mRNA OS=Homo sapiens PE=2 SV=1 - [A8K8W7_HUMAN]	2.7	2	2	2	1076	116.511	6.697754	0.679693	0.579123	0.664087	0.6409678	0.00749031	0.0446031
A0A024R159	Hydroxysteroid dehydrogenase like 2, isoform CRA_c OS=Homo sapiens GN=HSDL2 PE=4 SV=1 - [A0A024R159_HUMAN]	12.46	3	3	3	345	37.29621	6.100098	0.702447	0.531092	0.67542	0.6363201	0.0207272	0.0627002
D3DTX7	Collagen, type I, alpha 1, isoform CRA_a OS=Homo sapiens GN=COL1A1 PE=4 SV=1 - [D3DTX7_HUMAN]	30.06	2	21	81	885	84.68833	6.239746	0.660339	0.579597	0.663111	0.6343491	0.00556347	0.0408901
Q68CX1	Putative uncharacterized protein DKFZp761D0422 (Fragment) OS=Homo sapiens GN=DKFZp761D0422 PE=2 SV=1 - [Q68CX1_HUMAN]	3.35	2	2	2	836	93.16882	6.125488	0.590907	0.528831	0.7743	0.6313463	0.0377092	0.0869204
P04275	von Willebrand factor OS=Homo sapiens GN=VWF PE=1 SV=4 - [VWF_HUMAN]	20.8	2	46	60	2813	309.0579	5.478027	0.683451	0.558132	0.646817	0.6294664	0.00993057	0.0482281
P48061	Stromal cell-derived factor 1 OS=Homo sapiens GN=CXCL12 PE=1 SV=1 - [SDF1_HUMAN]	23.66	2	2	2	93	10.65882	9.876465	0.661454	0.604959	0.613761	0.6267246	0.0022031	0.033949
H0YAK9	Nephronectin (Fragment) OS=Homo sapiens GN=NPNT PE=1 SV=1 - [H0YAK9_HUMAN]	16.34	6	6	8	453	49.86766	7.825684	0.673187	0.621117	0.585315	0.6265397	0.00463384	0.0398629
Q08495	Dematin OS=Homo sapiens GN=DMTN PE=1 SV=3 - [DEMA_HUMAN]	8.64	3	3	4	405	45.48623	8.880371	0.701412	0.541709	0.633165	0.6254286	0.014915	0.0540327
B2R950	cDNA, FLJ94213, highly similar to Homo sapiens pregnancy-zone protein (PZP), mRNA OS=Homo sapiens PE=2 SV=1 - [B2R950_HUMAN]	6.14	2	8	16	1482	163.7676	6.379395	0.695226	0.491467	0.677349	0.6213475	0.0283465	0.0744976
B7Z7P4	cDNA FLJ53627, highly similar to Antigen peptide transporter 1 OS=Homo sapiens PE=2 SV=1 - [B7Z7P4_HUMAN]	8.23	2	2	2	547	59.90205	5.592285	0.756249	0.460561	0.644608	0.6204726	0.0479158	0.0996284
Q08209	Serine/threonine-protein phosphatase 2B catalytic subunit alpha isoform OS=Homo sapiens GN=PPP3CA PE=1 SV=1 -	3.65	2	2	2	521	58.65031	5.858887	0.544891	0.663839	0.634032	0.6142538	0.00847204	0.0456887

Biology Open • Supplementary information

	[PP2BA_HUMAN]													
A0A024R7F7	Transportin 2 (Importin 3, karyopherin beta 2b), isoform CRA_b OS=Homo sapiens GN=TNPO2 PE=4 SV=1 - [A0A024R7F7_HUMAN]	9.02	2	5	6	887	100.3427	4.98291	0.678406	0.54464	0.610468	0.6111713	0.00971989	0.0478622
Q6N041	Putative uncharacterized protein DKFZp686O16217 (Fragment) OS=Homo sapiens GN=DKFZp686O16217 PE=2 SV=1 - [Q6N041_HUMAN]	26.91	2	10	25	498	54.09048	7.181152	0.526843	0.628181	0.668184	0.607736	0.0113038	0.0493721
A8K6Q7	cDNA FLJ76889, highly similar to Homo sapiens butyrophilin, subfamily 3, member A3 (BTN3A3), transcript variant 1, mRNA OS=Homo sapiens PE=2 SV=1 - [A8K6Q7_HUMAN]	4.79	2	2	2	584	64.90287	5.60498	0.618529	0.597036	0.601341	0.6056352	0.00027705	0.0212234
A0N5G5	Rheumatoid factor D5 light chain (Fragment) OS=Homo sapiens GN=V<kappa>3 PE=2 SV=1 - [A0N5G5_HUMAN]	22.88	2	2	6	118	12.75845	8.968262	0.622265	0.517097	0.672047	0.603803	0.0130298	0.0512946
A6NDG6	Phosphoglycolate phosphatase OS=Homo sapiens GN=PGP PE=1 SV=1 - [PGP_HUMAN]	9.03	2	2	2	321	33.98451	6.138184	0.591125	0.605998	0.608406	0.601843	0.000184157	0.0212234
Q14388	Galactose-1-phosphate uridyl transferase (Fragment) OS=Homo sapiens GN=GALT PE=4 SV=1 - [Q14388_HUMAN]	44.44	2	2	2	63	7.206764	9.72998	0.692695	0.476449	0.622085	0.5970762	0.0240685	0.0676115
B1AP13	Complement decay-accelerating factor OS=Homo sapiens GN=CD55 PE=1 SV=1 - [B1AP13_HUMAN]	12.16	4	4	7	444	49.30664	8.821777	0.64072	0.560599	0.583522	0.5949468	0.00344154	0.0372192
A0A096LP30	Hemicentin-2 OS=Homo sapiens GN=HMCN2 PE=1 SV=1 - [A0A096LP30_HUMAN]	0.42	2	2	2	5059	541.6393	5.871582	0.597656	0.532207	0.643679	0.5911805	0.00620026	0.0423147
Q15599	Na(+)/H(+) exchange regulatory cofactor NHE-RF2 OS=Homo sapiens GN=SLC9A3R2 PE=1 SV=2 - [NHRF2_HUMAN]	7.12	2	2	2	337	37.39097	7.928223	0.632353	0.513789	0.608615	0.584919	0.00752804	0.0446277
Q00796	Sorbitol dehydrogenase OS=Homo sapiens GN=SORD PE=1 SV=4 - [DHSO_HUMAN]	9.24	2	2	3	357	38.29982	7.972168	0.50334	0.655682	0.590194	0.5830721	0.0110142	0.0493307

E7ENM0	Elastin OS=Homo sapiens GN=ELN PE=1 SV=1 - [E7ENM0_HUMAN]	14.16	5	5	9	706	61.11573	10.44775	0.613899	0.541887	0.57155	0.5757787	0.00241735	0.0349565
Q13976	cGMP-dependent protein kinase 1 OS=Homo sapiens GN=PRKG1 PE=1 SV=3 - [KGP1_HUMAN]	9.84	6	6	7	671	76.31597	6.036621	0.639636	0.472175	0.606044	0.5726181	0.0140228	0.0527413
B3KM95	Phosphatidate cytidylyltransferase OS=Homo sapiens PE=2 SV=1 - [B3KM95_HUMAN]	6.97	2	2	2	445	51.32603	7.093262	0.567332	0.621899	0.525249	0.5714933	0.00423588	0.0391996
O00399	Dynactin subunit 6 OS=Homo sapiens GN=DCTN6 PE=1 SV=1 - [DCTN6_HUMAN]	12.11	2	2	2	190	20.73364	6.315918	0.608659	0.482563	0.603538	0.5649203	0.00885048	0.046376
A0A024R8Q1	Glucosidase, alpha acid (Pompe disease, glycogen storage disease type II), isoform CRA_a OS=Homo sapiens GN=GAA PE=3 SV=1 - [A0A024R8Q1_HUMAN]	3.36	2	2	2	952	105.2708	5.998535	0.578767	0.485387	0.600191	0.5547815	0.00620826	0.0423147
P55058	Phospholipid transfer protein OS=Homo sapiens GN=PLTP PE=1 SV=1 - [PLTP_HUMAN]	8.72	3	3	3	493	54.70455	7.005371	0.617375	0.485448	0.535949	0.5462571	0.00709756	0.0439255
B7Z3U1	cDNA FLJ53798, highly similar to Long-chain fatty acid transport protein 1 (EC 6.2.1.-) OS=Homo sapiens PE=2 SV=1 - [B7Z3U1_HUMAN]	4.33	2	2	2	646	71.03585	8.528809	0.535559	0.526067	0.57657	0.5460654	0.00116341	0.0276207
E7ETR7	Gamma-glutamyltranspeptidase 1 (Fragment) OS=Homo sapiens GN=GGT1 PE=1 SV=1 - [E7ETR7_HUMAN]	12.64	2	2	2	174	18.46193	9.861816	0.620333	0.451682	0.535983	0.5359993	0.0108308	0.0492111
Q05C31	Uncharacterized protein OS=Homo sapiens PE=2 SV=1 - [Q05C31_HUMAN]	5.15	2	2	3	427	47.06553	7.752441	0.519463	0.434261	0.593321	0.5156814	0.0088838	0.0463829
O14880	Microsomal glutathione S-transferase 3 OS=Homo sapiens GN=MGST3 PE=1 SV=1 - [MGST3_HUMAN]	13.82	2	2	2	152	16.50557	9.378418	0.542343	0.491887	0.498033	0.5107543	0.00105369	0.0276207
O43927	C-X-C motif chemokine 13 OS=Homo sapiens GN=CXCL13 PE=1 SV=1 - [CXL13_HUMAN]	18.35	2	2	2	109	12.65592	10.33057	0.505774	0.458905	0.552392	0.5056904	0.0029675	0.0360868
Q13057	Bifunctional coenzyme A synthase OS=Homo sapiens GN=COASY PE=1 SV=4 - [COASY_HUMAN]	4.08	2	2	3	564	62.28995	6.990723	0.425643	0.562522	0.495833	0.4946661	0.00605995	0.0421458

Biology Open • Supplementary information

Q05BU7	WASF2 protein (Fragment) OS=Homo sapiens GN=WASF2 PE=2 SV=1 - [Q05BU7_HUMAN]	19.02	2	2	2	184	21.39618	9.246582	0.44013	0.635343	0.393183	0.4895518	0.0204536	0.0624988
P35625	Metalloproteinase inhibitor 3 OS=Homo sapiens GN=TIMP3 PE=1 SV=2 - [TIMP3_HUMAN]	25.12	5	5	7	211	24.12883	8.719238	0.484818	0.470748	0.490979	0.4821813	0.000133655	0.0212234
Q2M2W7	UPF0450 protein C17orf58 OS=Homo sapiens GN=C17orf58 PE=3 SV=2 - [CQ058_HUMAN]	31.96	3	3	3	97	11.21188	10.68213	0.48486	0.419439	0.506393	0.4702305	0.00242682	0.0349565
U3KQP3	NADH dehydrogenase [ubiquinone] 1 alpha subcomplex subunit 13 (Fragment) OS=Homo sapiens GN=NDUFA13 PE=4 SV=2 - [U3KQP3_HUMAN]	18.67	2	2	2	75	8.702527	9.979004	0.452316	0.388907	0.328539	0.3899207	0.00341335	0.0372107

Notes: Accession is the Swiss-Prot accession number. Ratio (T/N) = Ratio of tumors to controls.

Biology Open • Supplementary information

Table S2: 216 differentially vascular expressed proteins between colorectal cancer and control

Accession	Protein name	Gene names	Unique. Peptides	Ratio(T/N)	t-test p value	FDR
P52597	Heterogeneous nuclear ribonucleoprotein F	HNRNPF	3	1.89	0.0028848	0.03602517
Q9UBX5	Fibulin-5	FBLN5	10	1.81	0.00264451	0.03578389
P13727	Bone marrow proteoglycan	PRG2	8	1.81	0.000692846	0.02417818
Q6PKI6	YBX1 protein (Fragment)	YBX1	2	1.79	0.00314474	0.03645529
P40199	Carcinoembryonic antigen-related cell adhesion molecule 6	CEACAM6	2	1.74	0.0028515	0.03602517
J3KPD9	NME1-NME2 readthrough	NME1-NME2	7	1.73	0.00716588	0.04392547
A0PJ62	RPL14 protein	RPL14	3	1.72	0.0101914	0.04853833
A0A024QZV0	HCG1811539	hCG_1811539	12	1.68	0.000418756	0.0220588
P16402	Histone H1.3	HIST1H1D	4	1.65	0.0110193	0.0493307
A0A024RAL1	Chondroitin sulfate proteoglycan 2	CSPG2	8	1.64	0.00291471	0.03602517
P06702	Protein S100-A9	S100A9	7	1.63	0.00812617	0.04564185
P62280	40S ribosomal protein S11	RPS11	6	1.56	0.00562872	0.04089007
Q53T40	Uncharacterized protein FHL2	FHL2	2	1.55	0.00571149	0.04096058
D6R9B6	40S ribosomal protein S3a (Ribosomal protein S3A, isoform CRA_e)	RPS3A	6	1.55	0.00713285	0.04392547
P62750	60S ribosomal protein L23a (Large ribosomal subunit protein uL23)	RPL23A	6	1.54	0.011882	0.04952044
A0A024R9Q1	Thrombospondin 1	THBS1	11	1.54	0.000822568	0.02464064
H3BRU6	Poly(rC)-binding protein 2	PCBP2	2	1.53	0.00797253	0.04543268
P27824	Calnexin (IP90)	CANX	5	1.53	0.00171092	0.03071175
G3V1A4	Cofilin 1	CFL1	8	1.53	0.00363716	0.03816205
G3V511	Latent-transforming growth factor beta-binding protein 2	LTBP2	6	1.52	0.000451609	0.02242052
P07237	Protein disulfide-isomerase	P4HB	21	1.52	0.0068679	0.04337284
P18077	60S ribosomal protein L35a	RPL35A GIG33	4	1.51	0.00214433	0.03360443
M0R3F1	Heterogeneous nuclear ribonucleoprotein U-like protein 1	HNRNPUL1	3	1.51	0.00560924	0.04089007
P05787	Keratin, type II cytoskeletal 8	KRT8 CYK8	19	1.50	0.00722882	0.04392547
P05109	Protein S100-A8	S100A8	9	1.49	0.00107317	0.0276207
P55145	Mesencephalic astrocyte-derived neurotrophic factor	MANF	3	1.49	0.00113119	0.0276207
Q96AG4	Leucine-rich repeat-containing protein 5959, N-terminally processed]	LRRC59	6	1.48	0.0112661	0.04937208
Q7Z7M4	Superoxide dismutase	SOD2	8	1.47	0.00530828	0.04010711
O75822	Eukaryotic translation initiation factor 3 subunit J	EIF3J	2	1.46	0.00925534	0.0469705
E4W6B6	RPL27/NME2 fusion protein	RPL27	3	1.45	0.00467107	0.03986291
P50552	Vasodilator-stimulated phosphoprotein (VASP)	VASP	4	1.45	0.00941239	0.04729487
P02792	Ferritin light chain	FTL	5	1.45	0.00843711	0.04568865
Q9NS69	Mitochondrial import receptor subunit TOM22 homolog	TOMM22	2	1.44	0.00267284	0.03578389
G3V2V6	V-type proton ATPase subunit D	ATP6V1D	2	1.44	0.00458027	0.03986291
B8ZZU8	Elongin-B	ELOB	2	1.43	0.00537667	0.04010711
A0A024R884	Tenascin C	TNC	39	1.42	0.00110659	0.0276207
P46779	60S ribosomal protein L28	RPL28	5	1.42	0.00980827	0.04787214
P80723	Brain acid soluble protein 1	BASP1	6	1.41	0.00117024	0.0276207
P50454	Serpin H1 (47 kDa heat shock protein)	SERPINH1	16	1.41	0.0027188	0.03578389
P23229	Integrin alpha-6	ITGA6	5	1.41	0.00659722	0.04319699
P62269	40S ribosomal protein S18	RPS18 D6S218E	8	1.40	0.00265327	0.03578389
A0A024R5M3	Cortactin	CTTN hCG_23463	10	1.40	0.00229806	0.03424952
C9JXB8	60S ribosomal protein L24	RPL24	5	1.40	0.00327868	0.03662816
P12724	Eosinophil cationic protein	RNASE3 ECP RNS3	5	1.39	0.00682653	0.04336058
P39019	40S ribosomal protein S19	RPS19	6	1.39	0.00561338	0.04089007
B8ZZQ6	Prothymosin alpha	PTMA	2	1.39	0.00223652	0.03394897
P14780	Matrix metalloproteinase-9	MMP9 CLG4B	4	1.39	0.00225613	0.03397788
P21980	Protein-glutamine gamma-glutamyltransferase 2	TGM2	18	1.39	0.00163351	0.03016679

F8VS81	Twinfilin-1	TWF1	2	1.38	0.00622496	0.04231471
B2MUD5	Neutrophil elastase	ELA2	2	1.38	0.000245451	0.02122335
Q9BRF8	Serine/threonine-protein phosphatase CPPED1	CPPED1 CSTP1	2	1.38	0.00512689	0.04010711
Q8WU39	Marginal zone B- and B1-cell-specific protein	MZB1	5	1.38	0.000523649	0.02417818
M0R210	40S ribosomal protein S16	RPS16	6	1.38	0.0106904	0.04909167
P31151	Protein S100-A7	S100A7	2	1.38	0.00482255	0.04010711
P13639	Elongation factor 2	EEF2 EF2	21	1.38	0.00718396	0.04392547
P80188	Neutrophil gelatinase-associated lipocalin	LCN2	2	1.37	0.0084576	0.04568865
P52907	F-actin-capping protein subunit alpha-1	CAPZA1	6	1.37	0.010524	0.04893371
P06733	Alpha-enolase	ENO1	15	1.37	0.00109488	0.0276207
Q15084	Protein disulfide-isomerase A6	PDIA6	10	1.37	0.00129203	0.02816146
P52209	6-phosphogluconate dehydrogenase	PGD PGDH	13	1.37	0.000845213	0.02482191
V9HWC6	Peptidyl-prolyl cis-trans isomerase	HEL-S-39	11	1.37	9.80E-05	0.01951353
Q06323	Proteasome activator complex subunit 1	PSME1	13	1.36	0.00187285	0.03122954
P62136	Serine/threonine-protein phosphatase PP1-alpha catalytic subunit	PPP1CA	4	1.36	0.00365355	0.03817058
P36952	Serpin B5	SERPINB5	3	1.36	0.0037387	0.03858384
P68366	Tubulin alpha-4A chain	TUBA4A	5	1.36	6.16E-05	0.01737633
O60613	Selenoprotein F (15 kDa selenoprotein)	SELENOF	2	1.36	0.00546354	0.04046845
P68104	Elongation factor 1-alpha 1	EEF1A1	7	1.36	0.00324727	0.03652202
Q9Y5P6	Mannose-1-phosphate guanyltransferase beta	GMPPB	4	1.34	0.00325839	0.03652202
F8VQX6	Methyltransferase-like protein 7A	METTL7A	2	1.34	0.000191992	0.02122335
A0A0C4DGS1	Oligosaccharyl transferase 48 kDa subunit	DDOST	5	1.34	0.00850083	0.04568865
P63244	Receptor of activated protein C kinase 1	RACK1	9	1.34	0.0101019	0.04836624
P00558	Phosphoglycerate kinase 1	PGK1	18	1.34	0.00408899	0.03903086
P16401	Histone H1.5	HIST1H1B	4	1.34	0.00163465	0.03016679
H7C2W9	60S ribosomal protein L31 (Fragment)	RPL31	4	1.33	0.0059664	0.04196594
F2Z388	60S ribosomal protein L35	RPL35	2	1.33	0.0103299	0.04876855
P00338	L-lactate dehydrogenase A chain	LDHA	12	1.33	0.00198675	0.03218843
Q5CAQ5	Tumor rejection antigen	TRA1	26	1.33	0.00204705	0.03237974
Q16543	Hsp90 co-chaperone Cdc37	CDC37 CDC37A	3	1.33	0.000850618	0.02482191
Q5TD97	Four and a half LIM domains protein 5	FHL5 ACT	2	1.33	0.000680493	0.02417818
P31146	Coronin-1A	CORO1A	7	1.32	0.00101842	0.0271445
O15212	Prefoldin subunit 6	PFDN6	2	1.32	0.0105137	0.04893371
P20700	Lamin-B1	LMNB1	15	1.32	0.00612577	0.0421458
J3KTJ8	60S ribosomal protein L26	RPL26	5	1.32	0.00273797	0.03578389
Q14847	LIM and SH3 domain protein 1	LASP1	9	1.32	0.00189481	0.03135947
D6RDM7	Ubiquitin-conjugating enzyme E2 K	UBE2K	2	1.31	0.00589005	0.04162384
P13693	Translationally-controlled tumor protein	TPT1	5	1.31	0.000806614	0.02437847
P13667	Protein disulfide-isomerase A4	PDIA4	24	1.31	0.000249001	0.02122335
P15880	40S ribosomal protein S2	RPS2	10	1.31	0.00334941	0.03693178
Q9BSQ6	RPL13A protein	RPL13A	6	1.31	0.000251157	0.02122335
Q59GN8	PTK2 protein tyrosine kinase 2	PTK2	6	1.31	0.0367465	0.08566591
H0Y8G5	Heterogeneous nuclear ribonucleoprotein D0	HNRNPD	8	1.31	0.00718027	0.04392547
E9PPH5	Acidic leucine-rich nuclear phosphoprotein 32 family member E	ANP32E	3	1.31	0.00818338	0.04568865
Q15907	Ras-related protein Rab-11B	RAB11B	7	1.30	0.00531643	0.04010711
P62906	60S ribosomal protein L10a	RPL10A	5	1.30	0.00316474	0.0364812
A0A0G2JMH6	HLA class II histocompatibility antigen	HLA-DRA	6	1.30	0.0014463	0.02879839
P11678	Eosinophil peroxidase	EPX	12	1.30	0.0102827	0.04874921
P06748	Nucleophosmin	NPM1 NPM	8	1.30	0.000199147	0.02122335
X6RA14	S-formylglutathione hydrolase	ESD	5	1.30	0.011854	0.04952044
Q5H9A7	Metalloproteinase inhibitor 1	TIMP1	2	1.29	0.00324934	0.03652202
P09496	Clathrin light chain A (Lca)	CLTA	3	1.29	0.00774293	0.04520296
P26038	Moesin	MSN	22	1.29	0.00184929	0.03122671
P55769	NHP2-like protein 1	SNU13 NHP2L1	3	1.29	0.00384281	0.03871869
P19338	Nucleolin	NCL	20	1.29	0.00724089	0.04392547
O60493	Sorting nexin-3	SNX3	3	1.28	0.0011031	0.0276207
P04233	HLA class II histocompatibility antigen gamma chain	CD74 DHLAG	2	1.28	0.0115854	0.04941008
P55010	Eukaryotic translation initiation factor 5	EIF5	3	1.28	0.00359669	0.03795239
P48426	Phosphatidylinositol 5-phosphate 4-kinase type-2	PIP4K2A	2	1.28	0.003803	0.03871869

Biology Open • Supplementary information

	alpha					
Q8TCJ2	STT3-B	STT3B	4	1.28	0.00300117	0.03608678
G3XAL9	Solute carrier family 12	SLC12A2	2	1.28	0.000473203	0.02256045
P11021	Endoplasmic reticulum chaperone BiP	HSPA5 GRP78	31	1.27	0.00128533	0.02816146
P31947	14-3-3 protein sigma	SFN HME1	3	1.27	0.0106517	0.04909167
P59998	Actin-related protein 2/3 complex subunit 4	ARPC4	6	1.27	0.00282476	0.03602517
A0A0B4J1Z1	Serine/arginine-rich-splicing factor 7	SRSF7	2	1.27	0.000605588	0.02417818
P36955	Pigment epithelium-derived factor	SERPINF1	12	1.27	0.00946101	0.04729487
P54709	Sodium/potassium-transporting ATPase subunit beta-3	ATP1B3	5	1.27	0.00454318	0.03984794
C9JFR7	Cytochrome c	CYCS	3	1.26	0.00143259	0.02879839
O15144	Actin-related protein 2/3 complex subunit 2	ARPC2	12	1.26	5.33E-05	0.01737633
P04406	Glyceraldehyde-3-phosphate dehydrogenase	GAPDH	13	1.25	0.00641245	0.04237241
P08670	Vimentin	VIM	2	1.24	0.0600817	0.11483713
A0A087X1J7	Glutathione peroxidase	GPX3	7	0.79	0.00518691	0.04010711
A1L377	CABC1 protein	CABC1	2	0.79	0.0111599	0.04937208
Q4VB86	EPB41 protein	EPB41	6	0.79	0.0119007	0.04952044
F8WA11	CLIP-associating protein 1	CLASP1	3	0.79	0.00870577	0.04610634
Q05DK5	ADD2 protein	ADD2	3	0.79	0.00890662	0.04638294
H0YII4	Protein DDI1 homolog 2	DDI2	4	0.79	0.00232694	0.03439603
Q6P163	APOC2 protein	APOC2	2	0.78	0.004769	0.04009646
Q8N335	Glycerol-3-phosphate dehydrogenase 1-like protein	GPD1L	2	0.78	0.00794388	0.04542235
P35555	Fibrillin-1	FBN1	75	0.78	1.82E-05	0.0123214
O75569	PKR-associated protein X	PRKRA	3	0.78	0.00630714	0.04231471
P02042	Hemoglobin subunit delta	HBD	8	0.78	0.00310229	0.03608678
Q96A33	Coiled-coil domain-containing protein 47	CCDC47	2	0.77	0.00520841	0.04010711
O15031	Plexin-B2	PLXNB2	5	0.77	0.00950089	0.04729487
Q6NUQ2	Calmin	CLMN	2	0.77	0.009658	0.047762
E9PC52	Histone-binding protein RBBP7	RBBP7	2	0.77	0.00420094	0.03919959
Q8WUW1	Protein BRICK1	BRK1	2	0.77	0.00824721	0.04568865
G4V2I8	Anion exchange protein		16	0.77	0.0100785	0.04836624
Q96Q06	Perilipin-4	PLIN4	10	0.77	0.000687165	0.02417818
O94911	ATP-binding cassette sub-family A member 8	ABCA8	6	0.77	0.00999793	0.04827816
A6NHR9	SMC hinge domain-containing protein 1	SMCHD1	2	0.77	0.00612224	0.0421458
Q9BXN1	Asporin	ASPN	13	0.76	0.00320459	0.03649144
Q96LD4	Tripartite motif-containing protein 47	TRIM47	4	0.76	0.00091509	0.02539
P00748	Coagulation factor XII	F12	2	0.76	0.0030482	0.03608678
O60240	Perilipin-1	PLIN1	13	0.76	0.00320478	0.03649144
E9PNJ4	Stromal interaction molecule 1	STIM1	2	0.76	0.00905662	0.04651997
P51884	Lumican	LUM	14	0.76	0.000388729	0.02122335
O00468	Agrin	AGRN	27	0.76	0.0107918	0.04921108
P00326	Alcohol dehydrogenase 1C	ADH1C	3	0.76	0.000794862	0.02423971
P04004	Vitronectin	VTN	10	0.75	0.00824316	0.04568865
P02549	Spectrin alpha chain	SPTA1	37	0.75	0.00524693	0.04010711
P02760	Protein AMBP	AMBP	8	0.75	0.00480989	0.04010711
Q9BTW9	Tubulin-specific chaperone D	TBCD	2	0.75	0.00776642	0.04520296
Q02252	Methylmalonate-semialdehyde dehydrogenase	ALDH6A1	2	0.75	0.00480639	0.04010711
A0A087WSY5	Carboxypeptidase B2	CPB2	2	0.74	0.00113988	0.0276207
P20774	Mimecan	OGN OIF	13	0.74	0.000689989	0.02417818
Q9P0M6	Core histone macro-H2A.2	H2AFY2	2	0.74	0.011749	0.04941008
Q9BXX0	EMILIN-2	EMILIN2	2	0.74	0.00256641	0.03578389
B4DIH5	COP9 signalosome complex subunit 2	COPS2	5	0.74	0.0104601	0.04893371
P01034	Cystatin-C	CST3	2	0.74	0.00542279	0.0402547
J3KT17	Galectin	LGALS9C	2	0.74	0.0120002	0.0495979
P51888	Prolargin	PRELP	14	0.74	0.00119909	0.0276207
Q6VFQ6	Hemoglobin beta chain	HBB	2	0.74	0.00155942	0.02948959
B2RMN7	Spectrin beta chain	SPTB	38	0.73	0.000172569	0.02122335
P02649	Apolipoprotein E	APOE	11	0.73	0.000158745	0.02122335
P42025	Beta-centractin	ACTR1B	2	0.73	0.00338008	0.03702774
Q9Y5Y7	Lymphatic vessel endothelial hyaluronic acid receptor 1	LYVE1	2	0.73	0.0100819	0.04836624
Q03169	Tumor necrosis factor alpha-induced protein 2	TNFAIP2	5	0.73	0.00144056	0.02879839

Biology Open • Supplementary information

Q96PE7	Methylmalonyl-CoA epimerase, mitochondrial	MCEE	2	0.72	0.0114542	0.04941008
Q6UWY5	Olfactomedin-like protein 1	OLFML1	8	0.72	0.00295022	0.03605233
P15090	Fatty acid-binding protein	FABP4	2	0.72	0.00494468	0.04010711
Q9NTJ5	Phosphatidylinositide phosphatase SAC1	SACM1L	2	0.72	0.00384784	0.03871869
P16157	Ankyrin-1	ANK1	27	0.72	0.00324357	0.03652202
P22105	Tenascin-X	TNX	32	0.72	0.00398166	0.03903086
Q8WWI5	Choline transporter-like protein 1	SLC44A1	2	0.71	0.00901966	0.04651997
Q9BSD7	Cancer-related nucleoside-triphosphatase	NTPCR	2	0.71	0.00883754	0.04637597
P02452	Collagen alpha-1(I)	COL1A1	11	0.71	0.00262279	0.03578389
P10645	Chromogranin-A	CHGA	2	0.70	0.00290178	0.03602517
B2RUU3	Dedicator of cytokinesis 1	DOCK1	2	0.70	0.000703944	0.02423971
P02461	Collagen alpha-1(III) chain	COL3A1	12	0.70	0.00178242	0.03112593
H0YGS3	Microfibrillar-associated protein 5	MFAP5	3	0.69	0.000271082	0.02122335
P00325	Alcohol dehydrogenase 1B	ADH1B	8	0.69	0.0027055	0.03578389
V9GYZ1	Integrin beta	ITGB5	2	0.68	0.0013249	0.02834212
A0A087WTA8	Collagen alpha-2(I) chain	COL1A2	20	0.68	0.00487704	0.04010711
Q68CX8	Uncharacterized protein DKFZp686P12272	DKFZp686P12272	2	0.67	0.00779895	0.04520296
Q96G03	Phosphoglucomutase-2	PGM2	4	0.67	0.00764006	0.04495633
H0YKE1	GMP reductase 2	GMPR2	2	0.66	0.00976393	0.04787214
E9PHN7	Glutathione S-transferase Mu 2	GSTM2	5	0.66	0.00291407	0.03602517
D6RD17	Immunoglobulin J chain	JCHAIN	3	0.66	0.000208276	0.02122335
V9HW11	Epididymis secretory sperm binding protein Li 83p	HEL-S-83p	2	0.66	0.0119213	0.04952044
Q6FHW3	DF protein	DF	5	0.66	0.00229708	0.03424952
O60844	Zymogen granule membrane protein 16	ZG16	2	0.66	0.000751882	0.02423971
Q04828	Aldo-keto reductase family 1 member C1	AKR1C1 DDH DDH1	2	0.65	0.0116684	0.04941008
A5D8Z7	SIGLEC1 protein (Fragment)	SIGLEC1	2	0.65	0.00966528	0.047762
D3DQV2	Guanine nucleotide-binding protein subunit gamma	hCG_1992840	2	0.64	0.00628714	0.04231471
D3DTX7	Collagen, type I, alpha 1	COL1A1	2	0.63	0.00556347	0.04089007
P04275	von Willebrand factor	VWF F8VWF	2	0.63	0.00993057	0.04822809
P48061	Stromal cell-derived factor 1	CXCL12	2	0.63	0.0022031	0.03394897
H0YAK9	Nephronectin	NPNT	6	0.63	0.00463384	0.03986291
Q08209	Serine/threonine-protein phosphatase 2B catalytic subunit alpha isoform	PPP3CA	2	0.61	0.00847204	0.04568865
A0A024R7F7	Transportin 2	TNPO2	2	0.61	0.00971989	0.04786223
Q6N041	Uncharacterized protein DKFZp686O16217 (Fragment)	DKFZp686O16217	2	0.61	0.0113038	0.04937208
A6NDG6	Glycerol-3-phosphate phosphatase	PGP	2	0.60	0.000184157	0.02122335
B1AP13	Complement decay-accelerating factor	CD55	4	0.59	0.00344154	0.03721921
Q8NDA2	Hemicentin-2	HMCN2	2	0.59	0.00620026	0.04231471
Q15599	Na(+)/H(+) exchange regulatory cofactor NHE-RF2	SLC9A3R2	2	0.58	0.00752804	0.0446277
Q00796	Sorbitol dehydrogenase	SORD	2	0.58	0.0110142	0.0493307
Q13976	PGK1	PRKG1	6	0.57	0.0140228	0.04953151
E7ENM0	Elastin	ELN	5	0.58	0.00241735	0.03495653
O00399	Dynactin subunit 6 (Dynactin subunit p27)	DCTN6 WS3	2	0.56	0.00885048	0.04637597
A0A024R8Q1	Glucosidase, alpha acid	GAA	2	0.55	0.00620826	0.04231471
P55058	Phospholipid transfer protein	PLTP	3	0.55	0.00709756	0.04392547
E7ETR7	Glutathione hydrolase 1 proenzyme	GGT1	2	0.54	0.0108308	0.04921108
O14880	Microsomal glutathione S-transferase 3	MGST3	2	0.51	0.00105369	0.0276207
O43927	C-X-C motif chemokine 13	CXCL13	2	0.51	0.0029675	0.03608678
Q13057	Bifunctional coenzyme A synthase	COASY	2	0.49	0.00605995	0.0421458
P35625	Metalloproteinase inhibitor 3	TIMP3	5	0.48	0.000133655	0.02122335
U3KQP3	NADH dehydrogenase [ubiquinone] 1 alpha subcomplex subunit 13	NDUFA13	2	0.39	0.00341335	0.03721073

Notes: Accession is the Swiss-Prot accession number. Ratio (T/N) = Ratio of tumors to controls.

Table S3: 216 differentially expressed proteins involved 24 pathways.

Term	Description	LogP	InTerm/InList	Symbols
M5930	HALLMARK EPITHELIAL MESENCHYMAL TRANSITION	-13.740173	19/200	SERPINH1,COL1A1,COL1A2,COL3A1,VCAN,FBN1,TNC,ITGB5,LUM,PPIB,CXCL12,TGM2,THBS1,TIMP1,TIMP3,VIM,MFAP5,BASP1,FBLN5
hsa03010	Ribosome	-13.4249556	17/154	RPL10A,RPL23A,RPL24,RPL26,RPL27,RPL28,RPL31,RPL35A,RPS2,RPS3A,RPS11,RPS16,RPS18,RPS19,RPL14,RPL35,RPL13A
hsa04512	ECM-receptor interaction	-8.54462104	10/82	COL1A1,COL1A2,TNC,ITGA6,ITGB5,THBS1,TNXB,VTN,VWF,AGRN,DOCK1,PPP1CA,PTK2,VASP,HSP90B1,CDC37
hsa04510	Focal adhesion	-7.5735134	13/199	COL1A1,COL1A2,DOCK1,TNC,ITGA6,ITGB5,PPP1CA,PTK2,THBS1,TNXB,VASP,VTN,VWF
hsa04151	PI3K-Akt signaling pathway	-4.24789603	12/342	COL1A1,COL1A2,TNC,ITGA6,ITGB5,PTK2,THBS1,TNXB,HSP90B1,VTN,VWF,CDC37
M5944	HALLMARK ANGIOGENESIS	-7.59552005	7/36	COL3A1,VCAN,LUM,PRG2,PTK2,TIMP1,VTN,GPX3,SOD2,TIMP3
M5924	HALLMARK MTORC1 SIGNALING	-6.63096032	12/200	CANX,SERPINH1,ENO1,GAPDH,HSPA5,LDHA,PGK1,SORD,HSP90B1,TUBA4A,CORO1A,ATP6V1D,HLA-DRA,ITGB5,THBS1
M5946	HALLMARK COAGULATION	-6.36703216	10/138	APOC2,CPB2,CFD,F12,FBN1,MMP9,THBS1,TIMP1,TIMP3,VWF,CD55,VTN
hsa05205	Proteoglycans in cancer	-5.68969146	11/203	ANK1,CTTN,ITGB5,LUM,MMP9,MSN,PPP1CA,PTK2,THBS1,TIMP3,VTN,VCAN,FBN1,VASP,VWF,ARPC2,CXCL12
hsa00010	Glycolysis / Gluconeogenesis	-5.67831059	7/67	ADH1B,ADH1C,ENO1,GAPDH,LDHA,PGK1,PGM2,ESD,ALDH6A1,PGD,MCEE,PGP,GAA,HSPA5,TGM2,SRPX,ELOB,TIMP1,VCAN,GMPPB,AGRN
M5891	HALLMARK HYPOXIA	-4.12280712	9/200	ENO1,GAA,GAPDH,HSPA5,LDHA,PGK1,TGM2,SRPX,PGM2
hsa04066	HIF-1 signaling pathway	-3.55599193	6/101	ENO1,GAPDH,LDHA,PGK1,ELOB,TIMP1
hsa04810	Regulation of actin cytoskeleton	-5.507266	11/212	CFL1,DOCK1,ITGA6,ITGB5,MSN,PIP4K2A,PPP1CA,PTK2,ARPC4,ARPC2,BRK1,CLTA,CTTN,NCL,TUBA4A,VASP
M5926	HALLMARK MYC TARGETS V1	-4.91551669	10/200	CANX,HNRNPD,LDHA,NPM1,PGK1,RPS2,SRSF7,EIF3J,RPL14,RACK1
M5950	HALLMARK ALLOGRAFT REJECTION	-4.91551669	10/200	CD74,ELANE,HLA-DRA,MMP9,NPM1,RPS3A,RPS19,TIMP1,EIF3J,CXCL13
hsa04611	Platelet activation	-4.85589967	8/123	COL1A1,COL1A2,COL3A1,PPP1CA,PRKG1,STIM1,VASP,VWF,ATP1B3,CPB2,FHL2,FBLN5
hsa04918	Thyroid hormone synthesis	-4.30548315	6/74	ATP1B3,CANX,GPX3,HSPA5,HSP90B1,PDIA4,DDOST,P4HB,PDIA6,STT3B,EEF2,NPM1
M5945	HALLMARK HEME METABOLISM	-4.12280712	9/200	ADD2,ANK1,EPB41,HBB,HBD,MGST3,ALDH6A1,SPTA1,SPTB
hsa00480	Glutathione metabolism	-3.94442432	5/54	GGT1,GPX3,GSTM2,MGST3,PGD,ADH1B,ADH1C,AKR1C1,MMP9,PTK2,HSP90B1
hsa05310	Asthma	-3.81268955	4/31	HLA-DRA,PRG2,RNASE3,EPX
hsa04657	IL-17 signaling pathway	-3.75144881	6/93	LCN2,MMP9,S100A7,S100A8,S100A9,HSP90B1
M5909	HALLMARK MYOGENESIS	-3.378379	8/200	COL1A1,COL3A1,CFD,GAA,GPX3,ITGB5,PLXNB2,AGRN
hsa04612	Antigen processing and presentation	-3.21994164	5/77	CANX,CD74,HLA-DRA,HSPA5,PSME1
hsa00640	Propanoate metabolism	-2.55540093	3/32	LDHA,ALDH6A1,MCEE,MGST3,ATP6V1D,CYCS,TOMM22

Biology Open • Supplementary information

Table S4: 2058 identified proteins involved 66 pathways.

	Category	Term	Description	InList	Symbols
1	Hallmark Gene Sets	M5936	HALLMARK OXIDATIVE PHOSPHORYLATION	65/200	ACADM,ACADVL,ACAT1,SLC25A5,ATP5F1A,ATP5F1C,ATP5F1D,ATP5F1E,ATP5ME,ATP5PF,ATP6V1E1,ATP5PO,COX4I1,COX5B,COX6B1,COX6C,CPT1A,ECI1,DECR1,DLST,ECH1,ECHS1,ETFA,ETFB,ETFDH,FH,GPI,HSD17B10,HADHA,HADHB,IDH2,IDH3B,LDHA,LDHB,MAOB,MDH1,MGST3,ALDH6A1,NDUFA4,NDUFA8,NDUFB6,NDUFS1,NDUFS2,OPA1,PDHA1,PDHB,SLC25A3,POR,SDHA,SDHB,UQCRC2,UQCRFS1,UQCRH,VDAC1,VDAC3,AIFM1,COX5A,ATP6V1G1,LRPPRC,ATP5PD,PRDX3,NNT,TIMM13,BDH2,TOMM22,GNAI1,GNAI2,GNAI3,NDUFS5,UBA1,UBA7,APOE,ATP2A2,ATP2A3,CAPN2,GAPDH,IDE,ITPR1,LRP1,PPP3CA,MAPK3,ATP6V1A,ATP6V1B2,PPA1,AP2A1,AP2A2,CLTA,CLTB,CLTC,HIP1,TGM2,DCTN2,ATP1A1,ATP1B3,TPM1,TPM3,TPM4,CDC42,EIF2S1,RAC1
2	KEGG Pathway	hsa05012	Parkinson's disease	31/142	SLC25A5,ATP5F1A,ATP5F1C,ATP5F1D,ATP5F1E,ATP5PF,ATP5PO,COX4I1,COX5B,COX6B1,COX6C,GNAI1,GNAI2,GNAI3,NDUFA4,NDUFA8,NDUFB6,NDUFS1,NDUFS2,NDUFS5,SDHA,SDHB,UBA1,UBA7,UQCRC2,UQCRFS1,UQCRH,VDAC1,VDAC3,COX5A,ATP5PD
3	KEGG Pathway	hsa05010	Alzheimer's disease	34/171	APOE,ATP2A2,ATP2A3,ATP5F1A,ATP5F1C,ATP5F1D,ATP5F1E,ATP5PF,ATP5PO,CAPN2,COX4I1,COX5B,COX6B1,COX6C,GAPDH,HSD17B10,IDE,ITPR1,LRP1,NDUFA4,NDUFA8,NDUFB6,NDUFS1,NDUFS2,NDUFS5,PPP3CA,MAPK3,SDHA,SDHB,UQCRC2,UQCRFS1,UQCRH,COX5A,ATP5PD
4	KEGG Pathway	hsa00190	Oxidative phosphorylation	29/133	ATP5F1A,ATP5F1C,ATP5F1D,ATP5F1E,ATP5ME,ATP5PF,ATP6V1A,ATP6V1B2,ATP6V1E1,ATP5PO,COX4I1,COX5B,COX6B1,COX6C,NDUFA4,NDUFA8,NDUFB6,NDUFS1,NDUFS2,NDUFS5,PPA1,SDHA,SDHB,UQCRC2,UQCRFS1,UQCRH,COX5A,ATP6V1G1,ATP5PD
5	KEGG Pathway	hsa05016	Huntington's disease	35/193	AP2A1,AP2A2,SLC25A5,ATP5F1A,ATP5F1C,ATP5F1D,ATP5F1E,ATP5PF,ATP5PO,CLTA,CLTB,CLTC,COX4I1,COX5B,COX6B1,COX6C,HIP1,ITPR1,NDUFA4,NDUFA8,NDUFB6,NDUFS1,NDUFS2,NDUFS5,SDHA,SDHB,TGM2,UQCRC2,UQCRFS1,UQCRH,VDAC1,VDAC3,COX5A,ATP5PD,DCTN2
6	KEGG Pathway	hsa04260	Cardiac muscle contraction	14/78	ATP1A1,ATP1B3,ATP2A2,COX4I1,COX5B,COX6B1,COX6C,TPM1,TPM3,TPM4,UQCRC2,UQCRFS1,UQCRH,COX5A
7	KEGG Pathway	hsa04932	Non-alcoholic fatty liver disease (NAFLD)	19/149	CDC42,COX4I1,COX5B,COX6B1,COX6C,EIF2S1,NDUFA4,NDUFA8,NDUFB6,NDUFS1,NDUFS2,NDUFS5,RAC1,SDHA,SDHB,UQCRC2,UQCRFS1,UQCRH,COX5A
8	Hallmark Gene Sets	M5926	HALLMARK MYC TARGETS V1	63/200	CANX,DHX15,EEF1B2,EIF1AX,EIF2S1,EPRS,ETF1,XRCC6,GLO1,HNRNPA2B1,HPRT1,HSP90AB1,HSPD1,ILF2,KARS,LDHA,NPM1,PCBP1,PCNA,PGK1,PHB,SLC25A3,PSMA1,PSMA2,PSMB2,PSMB3,PSMC6,RAN,RPS2,RPS3,RPS6,RPS10,SET,SRSF1,SNRPA1,SNRPD3,SSBP1,TCP1,CCT3,TUFM,VBP1,VDAC1,VDAC3,XPO1,YWHAE,CNBP,EIF3J,COX5A,HNRNPR,SF3A1,RACK1,CCT4,CCT2,PTGES3,RUVBL2,PRDX3,YWHAQ,COPS5,CBX3,FAM120A,SF3B3,SERBP1,HNRNPA3
9	Hallmark Gene Sets	M5930	HALLMARK EPITHELIAL MESENCHYMAL TRANSITION	56/200	ACTA2,ANPEP,DST,CALD1,CALU,SERPINH1,COL1A1,COL3A1,COL4A2,COL5A1,COL5A2,COL6A2,COL6A3,COPA,VCAN,DAB2,DCN,DPYSL3,ECM1,ELN,FBLN1,FBLN2,FBN1,FLNA,FMOD,GPC1,TNC,ITGB1,LAMA3,LGALS1,LRP1,LUM,MMP2,MYLK,NNMT,CXCL12,SGCD,SNTB1,TAGLN,TGFBI,TGM2,TIMP3,TPM1,TPM4,VIM,PDLIM4,MYL9,BASP1,CAP2,FBLN5,POSTN,FERMT2,NID2,ABI3BP,GREM1,COLGALT1
10	Hallmark Gene Sets	M5935	HALLMARK FATTY ACID METABOLISM	50/158	ACADM,ACADVL,ADH1C,ALAD,ALDH1A1,ALDOA,BLVRA,CA2,CBR1,CD36,CPT1A,CRYZ,ECI1,DECR1,DLST,ECH1,ECHS1,EPHX1,ETFDH,ACSL1,FASN,FH,HSD17B10,HADHB,HMGCS2,HSP90AA1,IDH3B,LDHA,LGALS1,MAOA,MDH1,ME1,MIF,PCBD1,PDHA1,PDHB,PSME1,S100A10,SDHA,UGDH,YWHAH,AOC3,SUCLG2,GRHPR,PRDX6,HSPH1,ERP29,ACSL5,HSDL2,MCEE
11	KEGG Pathway	hsa04510	Focal adhesion	54/199	ACTN4,ACTN1,CAPN2,CAV1,CDC42,COL1A1,COL4A2,COL6A1,COL6A2,COL6A3,CRK,CRKL,DOCK1,FLNA,FLNB,FLNC,TNC,ILK,ITGA6,ITGA2B,ITGA7,ITGB1,LAMA3,LAMA4,LAMA5,LAMB1,LAM

Biology Open • Supplementary information

					B2,MYLK,PPP1R12A,PPP1R12B,PAK2,PPP1CA,PPP1CB,MAPK3,MAP2K1,PTK2,RAC1,RAC2,RAP1A,RAP1B,THBS3,THBS4,TLN1,TNXB,VASP,VCL,VTN,VWF,ZYX,ROCK2,MYL9,MYL12A,PARVA,TLN2,CD36,CD47,DAG1,AGRN,GNB2,HSP90AA1,HSP90AB1,KRAS,PCK2,PPP2R2A,RPS6,YWHAB,YWHAE,YWHAG,YWHAH,YWHAZ,YWHAQ,CDC37,GNG2,GNB4,CTNNA1,DAPK3,FH,GNAI1,GNAI2,GNAI3,GNAS,GSTP1,MMP2,MMP9,CXCL12,TPM3,TPR,CCDC6,CUL2,ARHGEF1,MAPK14,GNAO1,HLA-DRA,HSPA1A,HSPA1B,HSPA2
12	KEGG Pathway	hsa04512	ECM-receptor interaction	24/82	CD36,CD47,COL1A1,COL4A2,COL6A1,COL6A2,COL6A3,DAG1,TNC,ITGA6,ITGA2B,ITGA7,ITGB1,LAMA3,LAMA4,LAMA5,LAMB1,LAMB2,THBS3,THBS4,TNXB,VTN,VWF,AGRN
13	KEGG Pathway	hsa04151	PI3K-Akt signaling pathway	40/342	COL1A1,COL4A2,COL6A1,COL6A2,COL6A3,GNB2,HSP90AA1,HSP90AB1,TNC,ITGA6,ITGA2B,ITGA7,ITGB1,KRAS,LAMA3,LAMA4,LAMA5,LAMB1,LAMB2,PCK2,PPP2R2A,MAPK3,MAP2K1,PTK2,RAC1,RPS6,THBS3,THBS4,TNXB,VTN,VWF,YWHAB,YWHAE,YWHAG,YWHAH,YWHAZ,YWHAQ,CDC37,GNG2,GNB4
14	KEGG Pathway	hsa05200	Pathways in cancer	40/395	CDC42,COL4A2,CRK,CRKL,CTNNA1,DAPK3,FH,GNAI1,GNAI2,GNAI3,GNAS,GNB2,GSTP1,HSP90AA1,HSP90AB1,ITGA6,ITGA2B,ITGB1,KRAS,LAMA3,LAMA4,LAMA5,LAMB1,LAMB2,MMP2,MMP9,MAPK3,MAP2K1,PTK2,RAC1,RAC2,CXCL12,TPM3,TPR,CCDC6,CUL2,ARHGEF1,ROCK2,GNG2,GNB4
15	KEGG Pathway	hsa05145	Toxoplasmosis	17/113	MAPK14,GNAI1,GNAI2,GNAI3,GNAO1,HLA-DRA,HSPA1A,HSPA1B,HSPA2,ITGA6,ITGB1,LAMA3,LAMA4,LAMA5,LAMB1,LAMB2,MAPK3
16	KEGG Pathway	hsa05222	Small cell lung cancer	10/84	COL4A2,ITGA6,ITGA2B,ITGB1,LAMA3,LAMA4,LAMA5,LAMB1,LAMB2,PTK2
17	KEGG Pathway	hsa01200	Carbon metabolism	40/114	ACADM,ACAT1,ACO1,ADH5,ALDOA,CAT,DLST,ECHS1,ENO1,FBP1,FH,GAPDH,GOT1,GPI,HADHA,HK3,IDH2,IDH3B,MDH1,ME1,ME2,ALDH6A1,PCCA,PDHA1,PDHB,PFKL,PFKM,PFKP,PGD,PGK1,PKM,SDHA,SDHB,TALDO1,TPI1,SUCLG2,H6PD,PGLS,MCEE,PGP,ADH1B,ADH1C,ALDH2,ALDH1B1,ALDH7A1,LDHA,LDHB,PCK2,AKR1A1,PGM2,ACY1,BCAT2,MAT2A,ALDH18A1
18	KEGG Pathway	hsa00010	Glycolysis / Gluconeogenesis	25/67	ADH1B,ADH1C,ADH5,ALDH2,ALDH1B1,ALDOA,ALDH7A1,ENO1,FBP1,GAPDH,GPI,HK3,LDHA,LDHB,PCK2,PDHA1,PDHB,PFKL,PFKM,PFKP,PGK1,PKM,TPI1,AKR1A1,PGM2
19	KEGG Pathway	hsa01230	Biosynthesis of amino acids	19/75	ACO1,ACY1,ALDOA,ALDH7A1,BCAT2,ENO1,GAPDH,GOT1,IDH2,IDH3B,MAT2A,PFKL,PFKM,PFKP,PGK1,PKM,ALDH18A1,TALDO1,TPI1
20	KEGG Pathway	M00001	Glycolysis (Embden-Meyerhof pathway), glucose => pyruvate	11/25	ALDOA,ENO1,GAPDH,GPI,HK3,PFKL,PFKM,PFKP,PGK1,PKM,TPI1
21	KEGG Pathway	M00003	Gluconeogenesis, oxaloacetate => fructose-6P	7/17	ALDOA,ENO1,FBP1,GAPDH,PCK2,PGK1,TPI1
22	KEGG Pathway	M00002	Glycolysis, core module involving three-carbon compounds	5/12	ENO1,GAPDH,PGK1,PKM,TPI1
23	KEGG Pathway	hsa04610	Complement and coagulation cascades	32/79	A2M,SERPINC1,SERPING1,C1QC,C2,C3,C5,C8A,C8B,C8G,CLU,CPB2,CD55,F2,F5,F10,F12,F13A1,F13B,FGA,FGB,FGG,SERPIND1,CFH,CFI,ITGAM,ITGB2,KLKB1,PLG,SERPINF2,VTN,VWF,ANXA1,APOA1,APOC3,CAPN2,CRIP2,DPP4,FBN1,GNB2,GSN,HMGCS2,LRP1,LTA4H,MMP2,MMP9,PF4,PREP,RAC1,S100A13,TIMP3,PEF1,ANXA5,RHOG,CA2,CD36,COL4A2,CTSD,GNAI2,GNAI3,HSPA1A,HSPA5,ME1,MMP12,PFN1,S100A9,S100A12,EHD1,LAP3,GNG2,GNB4
24	Hallmark Gene Sets	M5946	HALLMARK COAGULATION	41/138	A2M,ANXA1,APOA1,APOC3,SERPINC1,SERPING1,C2,C3,C8A,C8B,C8G,CAPN2,CLU,CPB2,CRIP2,DPP4,F2,F10,F12,F13B,FBN1,FGA,FGG,GNB2,GSN,CFH,HMGCS2,CFI,KLKB1,LRP1,LTA4H,MMP2,MMP9,PF4,PLG,PREP,RAC1,S100A13,TIMP3,VWF,PEF1
25	Hallmark Gene Sets	M5921	HALLMARK COMPLEMENT	39/200	ANXA5,RHOG,SERPINC1,SERPING1,C1QC,C2,C3,CA2,CD36,CLU,COL4A2,CTSD,CD55,DPP4,F2,F5,F10,GNAI2,GNAI3,GNB2,CFH,HSPA1A,HSPA5,ITGAM,KLKB1,LRP1,LTA4H,ME1,MMP12,PF

Biology Open • Supplementary information

					N1,PLG,PREP,S100A9,S100A12,S100A13,EHD1,LAP3,GNG2,GNB4
26	Hallmark Gene Sets	M5924	HALLMARK MTORC1 SIGNALING	49/200	ACLY,ADD3,AK4,ALDOA,ATP2A2,CANX,SERPINH1,ENO1,EPRS,ETF1,GAPDH,GLRX,GOT1,GPI,GSR,HPRT1,HSPA4,HSPA5,HSPD1,ITGB2,LDHA,LTA4H,M6PR,ME1,PNP,PFKL,PGK1,PPA1,PSMA3,PSMB5,PSMC6,PSMD12,QDPR,RAB1A,RPA1,SORD,TCEA1,TPI1,TUBA4A,WARS,USO1,ACTR3,ACTR2,NAMPT,COPS5,CORO1A,ERO1A,ARPC5L,FAM129A
27	KEGG Pathway	hsa04810	Regulation of actin cytoskeleton	50/212	ACTN4,ACTN1,CDC42,CFL2,CRK,CRKL,DOCK1,F2,GSN,ITGA6,ITGA2B,ITGA7,ITGAM,ITGB1,ITGB2,KRAS,MSN,MYH9,MYH10,MYLK,PPP1R12A,PPP1R12B,PAK2,PFN1,PIP4K2A,PPP1CA,PPP1CB,MAPK3,MAP2K1,PTK2,RAC1,RAC2,RDX,RRAS,VCL,ARHGEF7,ARHGEF1,ROCK2,ARPC5,ARPC4,ARPC1B,ARPC2,MYL9,ARPC1A,MYL12A,NCKAP1,CYFIP1,BRK1,MYH14,ARPC5L,RHOG,CAV1,CLTA,CLTB,CLTC,CTNNA1,DNM2,CTTN,ILK,SEPT2,ELMO1,CD2AP,MAPK14,DYNC1H1,DYNC1I2,FLNA,FLNB,FLNC,LBP,TJP1,RAB7A,DYNC1LI1,BIN1,ARF6,PTPRC,VASP,PLPP1,MARCKSL1,NCL,TUBA4A,YWHAZ,YWHAQ,TUBB1,TUBB6,TUBB
28	KEGG Pathway	hsa04810	Regulation of actin cytoskeleton	50/212	ACTN4,ACTN1,CDC42,CFL2,CRK,CRKL,DOCK1,F2,GSN,ITGA6,ITGA2B,ITGA7,ITGAM,ITGB1,ITGB2,KRAS,MSN,MYH9,MYH10,MYLK,PPP1R12A,PPP1R12B,PAK2,PFN1,PIP4K2A,PPP1CA,PPP1CB,MAPK3,MAP2K1,PTK2,RAC1,RAC2,RDX,RRAS,VCL,ARHGEF7,ARHGEF1,ROCK2,ARPC5,ARPC4,ARPC1B,ARPC2,MYL9,ARPC1A,MYL12A,NCKAP1,CYFIP1,BRK1,MYH14,ARPC5L
29	KEGG Pathway	hsa05100	Bacterial invasion of epithelial cells	26/76	RHOG,CAV1,CDC42,CLTA,CLTB,CLTC,CRK,CRKL,CTNNA1,DNM2,DOCK1,CTTN,ILK,ITGB1,SEPT2,PTK2,RAC1,VCL,ELMO1,ARPC5,ARPC4,ARPC1B,ARPC2,ARPC1A,CD2AP,ARPC5L
30	KEGG Pathway	hsa05132	Salmonella infection	25/86	RHOG,CDC42,MAPK14,DYNC1H1,DYNC1I2,FLNA,FLNB,FLNC,LBP,MYH9,MYH10,PFN1,MAPK3,RAC1,TJP1,RAB7A,ROCK2,ARPC5,ARPC4,ARPC1B,ARPC2,ARPC1A,DYNC1LI1,MYH14,ARPC5L
31	KEGG Pathway	hsa05131	Shigellosis	20/65	RHOG,CDC42,CRK,CRKL,MAPK14,DOCK1,CTTN,ITGB1,PFN1,MAPK3,RAC1,VCL,ROCK2,ELMO1,ARPC5,ARPC4,ARPC1B,ARPC2,ARPC1A,ARPC5L
32	KEGG Pathway	hsa04666	Fc gamma R-mediated phagocytosis	22/91	BIN1,ARF6,CDC42,CFL2,CRK,CRKL,DNM2,GSN,MAPK3,MAP2K1,PTPRC,RAC1,RAC2,VASP,PLPP1,ARPC5,ARPC4,ARPC1B,ARPC2,ARPC1A,MARCKSL1,ARPC5L
33	KEGG Pathway	hsa05130	Pathogenic Escherichia coli infection	17/55	CDC42,CTTN,ITGB1,NCL,TUBA4A,YWHAZ,ROCK2,ARPC5,ARPC4,ARPC1B,ARPC2,ARPC1A,YWHAQ,TUBB1,ARPC5L,TUBB6,TUBB
34	Hallmark Gene Sets	M5905	HALLMARK ADIPOGENESIS	42/200	ACADM,ACLY,ALDH2,ALDOA,APOE,ATP1B3,ATP5PO,C3,CAT,CD36,DECR1,ECH1,ECHS1,ETFB,FABP4,FAH,ITGA7,LAMA4,LIPE,ME1,MGST3,MYLK,ORM1,PFKL,POR,QDPR,SDHB,SLC25A1,TALDO1,TST,YWHAG,AIFM1,SORBS1,PRDX3,PDCD4,DHRS7,CMPK1,RMDN3,AGPAT3,ITIH5,CMBL,CAVIN1
35	KEGG Pathway	hsa04144	Endocytosis	48/260	AP2A1,AP2A2,BIN1,ARF3,ARF6,CAPZA1,CAPZA2,CAV1,CDC42,CLTA,CLTB,CLTC,DAB2,DNM2,HSPA1A,HSPA1B,HSPA2,RAB5A,RAB5B,RAB5C,RAB7A,EEA1,SNX4,SNX3,GBF1,RAB11B,VPS26A,GIT2,WASHC5,ARPC5,ARPC4,ARPC1B,ARPC2,ARPC1A,EHD1,RAB31,WASHC4,SNX12,SH3KBP1,EHD3,EHD2,SH3GLB1,CHMP5,VPS29,VPS35,RUFY1,ARPC5L,WASHC2A
36	KEGG Pathway	hsa04145	Phagosome	36/154	ATP6V1A,ATP6V1B2,ATP6V1E1,C3,CANX,CD36,CYBB,DYNC1H1,DYNC1I2,HLA-DRA,ITGAM,ITGB1,ITGB2,M6PR,MPO,RAB5A,RAB5B,RAB5C,RAC1,TAP1,TAP2,THBS3,THBS4,TUBA4A,RAB7A,EEA1,STX7,ATP6V1G1,SEC22B,MRC2,CORO1A,SEC61A1,DYNC1LI1,TUBB1,TUBB6,TUBB,CAMK2D,CAMP,CD74,MAPK14,CTSD,HSPD1,LBP,PPP3CA,MAPK3,TRADD
37	KEGG Pathway	hsa04145	Phagosome	36/154	ATP6V1A,ATP6V1B2,ATP6V1E1,C3,CANX,CD36,CYBB,DYNC1H1,DYNC1I2,HLA-DRA,ITGAM,ITGB1,ITGB2,M6PR,MPO,RAB5A,RAB5B,RAB5C,RAC1,TAP1,TAP2,THBS3,THBS4,TUBA4A,RAB7A,EEA1,STX7,ATP6V1G1,SEC22B,MRC2,CORO1A,SEC61A1,DYNC1LI1,TUBB1,TUBB6,TUBB
38	KEGG Pathway	hsa05152	Tuberculosis	21/179	C3,CAMK2D,CAMP,CD74,MAPK14,CTSD,HLA-DRA,HSPD1,ITGAM,ITGB2,LBP,PPP3CA,MAPK3,RAB5A,RAB5B,RAB5C,RAB7A,EEA1,TRADD,MRC2,CORO1A
39	KEGG Pathway	hsa04611	Platelet activation	31/123	COL1A1,COL3A1,MAPK14,FGA,FGB,FGG,GNAI1,GNAI2,GNAI3,GNAS,GUCY1A1,ITGA2B,ITGB1,ITPR1,MYLK,PPP1R12A,PPP1CA,PPP1CB,PRKG1,MAPK3,RAP1A,RAP1B,TLN1,VASP,VWF,SNAP23,ARHGEF1,ROCK2,MYL12A,TLN2,FERMT3,SLC25A5,ATP1A1,ATP1B3,ATP2A2,ATP2A3,GTF

Biology Open • Supplementary information

					2I,PPP3CA,MAP2K1,VDAC1,VDAC3,PDE5A,MYL9,GNAO1,HSPA1A,HSPA1B,HSPA2,HSP90AA1,HSP90AB1,KRAS,MMP2,MMP9,TJP1,TUBA4A,TUBB1,TUBB6,TUBB,ACTA2,CALD1,MYH11,PPP1R12B,PLA2G2A,CAMK2D,EEF2,COMT,GNB2,MAOA,MAOB,PPP2R2A,GNG2,GNB4,SCN7A,TPM1,TPM3,TPM4,LIPE,RAC1,RAC2,RRAS,PLIN1,RPS6,CA2,FABP4
40	KEGG Pathway	hsa04022	cGMP-PKG signaling pathway	25/163	SLC25A5,ATP1A1,ATP1B3,ATP2A2,ATP2A3,GNAI1,GNAI2,GNAI3,GTF2I,GUCY1A1,ITPR1,MYLK,PPP1R12A,PPP1CA,PPP1CB,PPP3CA,PRKG1,MAPK3,MAP2K1,VASP,VDAC1,VDAC3,PDE5A,ROCK2,MYL9
41	KEGG Pathway	hsa04915	Estrogen signaling pathway	16/98	GNAI1,GNAI2,GNAI3,GNAO1,GNAS,HSPA1A,HSPA1B,HSPA2,HSP90AA1,HSP90AB1,ITPR1,KRAS,MMP2,MMP9,MAPK3,MAP2K1
42	KEGG Pathway	hsa04540	Gap junction	15/88	GNAI1,GNAI2,GNAI3,GNAS,GUCY1A1,ITPR1,KRAS,PRKG1,MAPK3,MAP2K1,TJP1,TUBA4A,TUBB1,TUBB6,TUBB
43	KEGG Pathway	hsa04270	Vascular smooth muscle contraction	18/121	ACTA2,CALD1,GNAS,GUCY1A1,ITPR1,MYH11,MYLK,PPP1R12A,PPP1R12B,PLA2G2A,PPP1CA,PPP1CB,PRKG1,MAPK3,MAP2K1,ARHGEF1,ROCK2,MYL9
44	KEGG Pathway	hsa04921	Oxytocin signaling pathway	20/153	CAMK2D,EEF2,GNAI1,GNAI2,GNAI3,GNAO1,GNAS,GUCY1A1,ITPR1,KRAS,MYLK,PPP1R12A,PPP1R12B,PPP1CA,PPP1CB,PPP3CA,MAPK3,MAP2K1,ROCK2,MYL9
45	KEGG Pathway	hsa04728	Dopaminergic synapse	18/130	CAMK2D,COMT,MAPK14,GNAI1,GNAI2,GNAI3,GNAO1,GNAS,GNB2,ITPR1,MAOA,MAOB,PPP1CA,PPP1CB,PPP2R2A,PPP3CA,GNG2,GNB4
46	KEGG Pathway	hsa04730	Long-term depression	11/60	GNAI1,GNAI2,GNAI3,GNAO1,GNAS,GUCY1A1,ITPR1,KRAS,PRKG1,MAPK3,MAP2K1
47	KEGG Pathway	hsa04261	Adrenergic signaling in cardiomyocytes	17/144	ATP1A1,ATP1B3,ATP2A2,CAMK2D,MAPK14,GNAI1,GNAI2,GNAI3,GNAS,PPP1CA,PPP1CB,PPP2R2A,MAPK3,SCN7A,TPM1,TPM3,TPM4
48	KEGG Pathway	hsa04024	cAMP signaling pathway	21/198	ATP1A1,ATP1B3,ATP2A2,CAMK2D,GNAI1,GNAI2,GNAI3,GNAS,LIPE,PPP1R12A,PPP1CA,PPP1CB,MAPK3,MAP2K1,RAC1,RAC2,RAP1A,RAP1B,RRAS,ROCK2,MYL9
49	KEGG Pathway	hsa04713	Circadian entrainment	13/96	CAMK2D,GNAI1,GNAI2,GNAI3,GNAO1,GNAS,GNB2,GUCY1A1,ITPR1,PRKG1,MAPK3,GNG2,GNB4
50	KEGG Pathway	hsa04371	Apelin signaling pathway	16/138	ACTA2,GNAI1,GNAI2,GNAI3,GNB2,ITPR1,KRAS,LIPE,MYLK,PLIN1,MAPK3,MAP2K1,RPS6,RRAS,GNG2,GNB4
51	KEGG Pathway	hsa04726	Serotonergic synapse	14/113	GNAI1,GNAI2,GNAI3,GNAO1,GNAS,GNB2,ITPR1,KRAS,MAOA,MAOB,MAPK3,MAP2K1,GNG2,GNB4
52	KEGG Pathway	hsa04971	Gastric acid secretion	10/75	ATP1A1,ATP1B3,CA2,CAMK2D,GNAI1,GNAI2,GNAI3,GNAS,ITPR1,MYLK
53	KEGG Pathway	hsa04066	HIF-1 signaling pathway	6/101	ENO1,GAPDH,LDHA,PGK1,ELOB,TIMP1,MAPK3,MAP2K2,RPS6,CUL2
54	KEGG Pathway	hsa04725	Cholinergic synapse	12/112	CAMK2D,GNAI1,GNAI2,GNAI3,GNAO1,GNB2,ITPR1,KRAS,MAPK3,MAP2K1,GNG2,GNB4
55	KEGG Pathway	hsa03040	Spliceosome	32/134	DHX15,HNRNPK,HSPA1A,HSPA1B,HSPA2,HNRNPM,SNU13,PCBP1,SRSF1,SRSF5,SNRPA1,SNRPD3,DDX39B,PRPF4,EFTUD2,DDX23,EIF4A3,DDX46,RBM8A,ALYREF,SF3A1,PRPF8,TCERG1,SF3A3,LSM6,DDX42,ACIN1,SF3B3,SF3B1,PRPF19,RBM25,HNRNPA3
56	KEGG Pathway	hsa05146	Amoebiasis	26/96	ACTN4,ACTN1,C8A,C8B,C8G,COL1A1,COL3A1,COL4A2,CTSG,GNAS,HSPB1,ITGAM,ITGB2,LAMA3,LAMA4,LAMA5,LAMB1,LAMB2,SERPINB6,SERPINB9,PTK2,RAB5A,RAB5B,RAB5C,VCL,RAB7A
57	Hallmark Gene Sets	M5915	HALLMARK APICAL JUNCTION	38/200	ACTA1,ACTN4,ACTN1,CALB2,MAPK14,VCAN,CTNNA1,FBN1,FLNC,GNAI1,GNAI2,ITGB1,LAMA3,MMP2,MMP9,MSN,MYH9,MYH10,PFN1,PTK2,PTPRC,RAC2,RRAS,RSU1,FSCN1,TGFBI,THBS3,TJP1,VASP,VCL,VWF,YWHAH,ZYX,ARPC2,SORBS3,MYL9,PARVA,NEXN,CDC42,CYBB,GNAI3,ITGAM,ITGB2,PTPN11,RAC1,RAP1A,RAP1B,CXCL12,ROCK2,MYL12A,CTTN,HSPA4,MYH11,PCNA,PPP2R2A,RDX,TUBA4A,TJP2,ACTR3,ACTR2,MYH14
58	KEGG Pathway	hsa04670	Leukocyte transendothelial migration	27/114	ACTN4,ACTN1,CDC42,MAPK14,CTNNA1,CYBB,GNAI1,GNAI2,GNAI3,ITGAM,ITGB1,ITGB2,MMP2,MMP9,MSN,PTK2,PTPN11,RAC1,RAC2,RAP1A,RAP1B,CXCL12,VASP,VCL,ROCK2,MYL9,MYL12A
59	KEGG Pathway	hsa04530	Tight junction	25/170	ACTN4,ACTN1,CDC42,CTTN,HSPA4,ITGB1,MSN,MYH9,MYH10,MYH11,PCNA,PPP2R2A,RAC1,R

Biology Open • Supplementary information

60	KEGG Pathway	hsa05322	Systemic lupus erythematosus	30/133	AP1A,RDX,TJP1,TUBA4A,VASP,TJP2,ROCK2,ACTR3,ACTR2,MYL9,MYL12A,MYH14 ACTN4,ACTN1,C1QC,C2,C3,C5,C8A,C8B,C8G,CTSG,HLA-DRA,SNRPD3,TROVE2,HIST1H4I,HIST1H4A,HIST1H4D,HIST1H4F,HIST1H4K,HIST1H4J,HIST1H4C,HIST1H4H,HIST1H4B,HIST1H4E,HIST1H4L,HIST2H4A,HIST1H2BJ,H2AFY,H2AFY2,HIST4H4,HIST2H4B,CDC42,GSN,HNRNPK,KRAS,PKM,MAPK3,PSMC1,RAC1,VDAC3,YWHAB,YWHAE,YWHAG,YWHAH,YWHAZ,TRADD,YWHAQ,UBR4,GNAI1,GNAI2,GNAI3,GNAO1,GNAS,GNB2,MAOA,MAOB,PPP1CA,PPP1CB,MAP2K1,GNG2,GNB4
61	KEGG Pathway	hsa05203	Viral carcinogenesis	35/201	ACTN4,ACTN1,C3,CDC42,GSN,HNRNPK,KRAS,PKM,MAPK3,PSMC1,RAC1,VDAC3,YWHAB,YWHAE,YWHAG,YWHAH,YWHAZ,HIST1H4I,HIST1H4A,HIST1H4D,HIST1H4F,HIST1H4K,HIST1H4J,HIST1H4C,HIST1H4H,HIST1H4B,HIST1H4E,HIST1H4L,HIST2H4A,TRADD,HIST1H2BJ,YWHAQ,UBR4,HIST4H4,HIST2H4B
62	KEGG Pathway	hsa05034	Alcoholism	32/180	GNAI1,GNAI2,GNAI3,GNAO1,GNAS,GNB2,KRAS,MAOA,MAOB,PPP1CA,PPP1CB,MAPK3,MAP2K1,HIST1H4I,HIST1H4A,HIST1H4D,HIST1H4F,HIST1H4K,HIST1H4J,HIST1H4C,HIST1H4H,HIST1H4B,HIST1H4E,HIST1H4L,HIST2H4A,HIST1H2BJ,H2AFY,GNG2,H2AFY2,GNB4,HIST4H4,HIST2H4B
63	Hallmark Gene Sets	M5909	HALLMARK MYOGENESIS	37/200	ACTA1,AK1,BIN1,APOD,CD36,CKB,CLU,CNN3,COL1A1,COL3A1,COL4A2,COL6A2,COL6A3,DMD,DTNA,EIF4A2,ACSL1,FDPS,GNAO1,GSN,IGFBP7,ITGA7,ITGB1,MYH9,MYH11,MYLK,PFKM,PTGIS,SGCD,SOD3,TAGLN,TPM3,PDLIM7,SORBS3,SORBS1,PLXNB2,AGRN
64	Hallmark Gene Sets	M5934	HALLMARK XENOBIOTIC METABOLISM	37/200	ACP2,ADH1C,ADH5,AHCY,ALDH2,APOE,ATP2A2,BLVRB,CA2,CAT,CBR1,CD36,CES1,COMT,ECH1,EPHX1,ETFDH,F10,FAH,FBLN1,FBP1,GSR,HPRT1,KARS,MAOA,PGD,PLG,POR,UGDH,VTN,AKR1C3,PAPSS2,PDLIM5,PTGES3,PGRMC1,DDAH2,DHRS7
65	Hallmark Gene Sets	M5891	HALLMARK HYPOXIA	37/200	AK4,ALDOA,ANXA2,CAV1,COL5A1,CSRP2,DCN,DTNA,ENO1,FBP1,GAPDH,GLRX,GPC1,GPI,HDLBP,HSPA5,LDHA,MIF,MYH9,PFKL,PFKP,PGK1,S100A4,SLC25A1,TGFBI,TGM2,TPD52,TPI1,SELENBP1,GRHPR,AKAP12,ERO1A,PGM2,NAGK,LXN,CAVIN3,CAVIN1,ALDH7A1,VCAN,GFPT1,GOT1,MDH1,ME1,ME2,NASP,PKM,RARS,TALDO1,TSTA3,TXN,COPB2,AKR1A1,PGLS,GMPPB,AK3,ALG1,AGRN
66	Hallmark Gene Sets	M5937	HALLMARK GLYCOLYSIS	37/200	AK4,ALDOA,ALDH7A1,COL5A1,VCAN,DCN,ENO1,GFPT1,GLRX,GOT1,GPC1,HDLBP,HSPA5,LDHA,MDH1,ME1,ME2,MIF,NASP,PFKP,PGK1,PKM,RARS,TALDO1,TGFBI,TPI1,TSTA3,TXN,COPB2,AKR1A1,PGLS,GMPPB,ERO1A,AK3,PGM2,ALG1,AGRN

Biology Open • Supplementary information

Table S5: EMT involved the identified proteins in colorectal cancer biological system.

Accession	Protein name	Gene name	Unique. Peptides	Ratio (T/N)	t-test p value
Q9UBX5	Fibulin-5	FBLN5	10	1.81	0.0026445
A0A024RAL1	Versican	VCAN	8	1.64	0.0029147
K7ENT6	Tropomyosin alpha-4 chain	TPM4	2	1.50	0.0501056
P40261	Nicotinamide N-methyltransferase	NNMT	4	1.46	0.0315235
A0A024R884	Tenascin C	TNC	39	1.42	0.0011066
P80723	Brain acid soluble protein 1	BASP1	6	1.41	0.0011702
P50454	Serpin H1	SERPINH1	16	1.41	0.0027188
P21980	Protein-glutamine gamma-glutamyltransferase 2	TGM2	18	1.39	0.0016335
B3KQF5	Calumenin	CALU	10	1.31	0.0033446
Q05682	Caldesmon	CALD1	5	1.28	0.0223301
Q92629	Delta-sarcoglycan	SGCD	2	1.26	0.0135417
P53621	Coatomer subunit alpha	COPA	14	1.26	0.0513663
P08670	Vimentin	VIM	2	1.24	0.0600817
P15144	Aminopeptidase N	ANPEP	3	1.23	0.0274478
Q01995	Transgelin	TAGLN	18	1.22	0.0204575
B1ALD9	Periostin	POSTN	3	1.21	0.0074766
P50479	PDZ and LIM domain protein 4	PDLIM4	6	1.11	0.106185
P09382	Galectin-1	LGALS1	8	1.08	0.001319
P05997	Collagen alpha-2(V) chain	COL5A2	3	1.05	0.107794
Q8NBJ5	Procollagen galactosyltransferase 1	COLGALT1	5	1.03	0.4726
Q15746	Myosin light chain kinase	MYLK	25	0.99	0.275123
B3KS64	FIBROMODULIN	FMOD	2	0.97	0.565324
B2RAW0	Disabled homolog 2	DAB2	2	0.93	0.354377
A0A0A0MSA0	Laminin subunit alpha-3	LAMA3	4	0.87	0.0275171
F8W9J4	Dystonin	DST	2	0.82	0.0281896
P35555	Fibrillin-1	FBN1	75	0.78	1.82E-05
B3KV06	Matrix metalloproteinase-28	MMP2	3	0.65	0.0228407
P48061	Stromal cell-derived factor 1	CXCL12	2	0.63	0.0022031
E7ENM0	Elastin	ELN	5	0.58	0.0024174
P35625	Metalloproteinase inhibitor 3	TIMP3	5	0.48	0.0001337

Notes: “Accession” is the Swiss-Prot accession number. Ratio (T/N) = Ratio of tumors to controls.

Biology Open • Supplementary information

Table S6: 78 differentially expressed proteins identified as colorectal cancer angiogenesis-related proteins.

Accession	Protein name	Gene names	Unique. Peptides	Ratio(T/N)	t-test p value	Antiangiogenic target
Q9UBX5	Fibulin-5	FBLN5	10	1.81	0.00264451	Yes
Q6PKI6	YBX1 protein	YBX1	2	1.79	0.00314474	-
P40199	Carcinoembryonic antigen-related cell adhesion molecule 6	CEACAM6	2	1.74	0.0028515	Yes
P06702	Protein S100-A9	S100A9	7	1.63	0.00812617	Yes
Q53T40	Uncharacterized protein FHL2	FHL2	2	1.55	0.00571149	-
A0A024R9Q1	Thrombospondin 1	THBS1	11	1.54	0.000822568	Yes
P27824	Calnexin	CANX	5	1.53	0.00171092	Yes
G3V1A4	Cofilin 1	CFL1	8	1.53	0.00363716	-
G3V511	Latent-transforming growth factor beta-binding protein 2	LTBP2	6	1.52	0.000451609	-
P07237	Protein disulfide-isomerase	PDIA1	21	1.52	0.0068679	Yes
P05787	Keratin, type II cytoskeletal 8	KRT8	19	1.50	0.00722882	-
P05109	Protein S100-A8	S100A8	9	1.49	0.00107317	Yes
Q7Z7M4	Superoxide dismutase	SOD2	8	1.47	0.00530828	-
P02792	Ferritin light chain	FTL	5	1.45	0.00843711	-
B8ZZU8	Elongin-B	TCEB2	2	1.43	0.00537667	-
A0A024R884	Tenascin C	TNC	39	1.42	0.00110659	Yes
P80723	Brain acid soluble protein 1	BASP1	6	1.41	0.00117024	-
P50454	Serpin H1 (47 kDa heat shock protein)	HSP47	16	1.41	0.0027188	Yes
P23229	Integrin alpha-6	ITGA6	5	1.41	0.00659722	-
A0A024R5M3	Cortactin	CTTN	10	1.40	0.00229806	Yes
P12724	Eosinophil cationic protein	ECP	5	1.39	0.00682653	-
B8ZZQ6	Prothymosin alpha	PTMA	2	1.39	0.00223652	-
P14780	Matrix metalloproteinase-9	MMP9	4	1.39	0.00225613	Yes
P21980	Protein-glutamine gamma-glutamyltransferase 2	TGM2	18	1.39	0.00163351	Yes
P31151	Protein S100-A7	S100A7	2	1.38	0.00482255	Yes
P13639	Elongation factor 2	EEF2	21	1.38	0.00718396	Yes
P80188	Neutrophil gelatinase-associated lipocalin	LCN2	2	1.37	0.0084576	Yes
P06733	Alpha-enolase	ENO1	15	1.37	0.00109488	-
P62136	Serine/threonine-protein phosphatase PP1-alpha catalytic subunit	PPP1CA	4	1.36	0.00365355	Yes
P63244	Receptor of activated protein C kinase 1	RACK1	9	1.34	0.0101019	Yes
P00558	Phosphoglycerate kinase 1	PGK1	18	1.34	0.00408899	Yes
Q59GN8	PTK2 protein tyrosine kinase 2	PTK2	6	1.31	0.0367465	-
P13667	Protein disulfide-isomerase A4	ERP70	24	1.31	0.000249001	-
P11678	Eosinophil peroxidase	EPO	12	1.30	0.0102827	Yes
P06748	Nucleophosmin	NPM1	8	1.30	0.000199147	-
Q5H9A7	Metalloproteinase inhibitor 1	TIMP1	2	1.29	0.00324934	-
P19338	Nucleolin	NCL	20	1.29	0.00724089	-
P04233	HLA class II histocompatibility antigen gamma chain	CD74	2	1.28	0.0115854	Yes
P11021	Endoplasmic reticulum chaperone BiP	GRP78	31	1.27	0.00128533	Yes
P31947	14-3-3 protein sigma	SFN	3	1.27	0.0106517	Yes
P36955	Pigment epithelium-derived factor	SERPINF1	12	1.27	0.00946101	-
P04406	Glyceraldehyde-3-phosphate dehydrogenase	GAPDH	13	1.25	0.00641245	-
P08670	Vimentin	VIM	2	1.24	0.0600817	-
A0A087X1J7	Glutathione peroxidase	GPX3	7	0.79	0.00518691	-
Q6P163	APOC2 protein	APOC2	2	0.78	0.004769	-
Q8N335	Glycerol-3-phosphate dehydrogenase 1-like protein	GPD1L	2	0.78	0.00794388	-
P35555	Fibrillin-1	FBN1	75	0.78	1.82E-05	-
P00748	Coagulation factor XII	F12	2	0.76	0.0030482	-
E9PNJ4	Stromal interaction molecule 1	STIM1	2	0.76	0.00905662	-
P51884	Lumican	LUM	14	0.76	0.000388729	-
O00468	Agrin	AGRN	27	0.76	0.0107918	-
P04004	Vitronectin	VTN	10	0.75	0.00824316	-
P20774	Mimecan	OGN	13	0.74	0.000689989	-
Q9BXX0	EMILIN-2	EMILIN2	2	0.74	0.00256641	-
P01034	Cystatin-C	CST3	2	0.74	0.00542279	-
P51888	Prolargin	PRELP	14	0.74	0.00119909	-
Q6VFQ6	Hemoglobin beta chain	HBB	2	0.74	0.00155942	-
P02649	Apolipoprotein E	APOE	11	0.73	0.000158745	Yes
Q9Y5Y7	Lymphatic vessel endothelial hyaluronic acid receptor 1	LYVE1	2	0.73	0.0100819	-
Q03169	Tumor necrosis factor alpha-induced protein 2	TNFAIP2	5	0.73	0.00144056	-
P15090	Fatty acid-binding protein	FABP4	2	0.72	0.00494468	Yes
P22105	Tenascin-X	TNX	32	0.72	0.00398166	-
P02452	Collagen alpha-1(I)	COL1A1	11	0.71	0.00262279	-
P10645	Chromogranin-A	CHGA	2	0.70	0.00290178	Yes

Biology Open • Supplementary information

P02461	Collagen alpha-1(III) chain	COL3A1	12	0.70	0.00178242	-
H0YGS3	Microfibrillar-associated protein 5	MFAP5	3	0.69	0.000271082	-
V9GYZ1	Integrin beta	ITGB5	2	0.68	0.0013249	-
A0A087WTA8	Collagen alpha-2(I) chain	COL1A2	20	0.68	0.00487704	-
E9PHN7	Glutathione S-transferase Mu 2	GSTM2	5	0.66	0.00291407	-
P04275	von Willebrand factor	VWF	2	0.63	0.00993057	Yes
P48061	Stromal cell-derived factor 1	CXCL12	2	0.63	0.0022031	-
H0YAK9	Nephronectin	NPNT	6	0.63	0.00463384	-
Q08209	Serine/threonine-protein phosphatase 2B catalytic subunit alpha isoform	PPP3CA	2	0.61	0.00847204	-
Q15599	Na(+)/H(+) exchange regulatory cofactor NHE-RF2	NHERF2	2	0.58	0.00752804	-
E7ENM0	Elastin	ELN	5	0.58	0.00241735	-
Q13976	cGMP-dependent protein kinase 1	PRKG1	6	0.57	0.0140228	-
O43927	C-X-C motif chemokine 13	CXCL13	2	0.51	0.0029675	-
P35625	Metalloproteinase inhibitor 3	TIMP3	5	0.48	0.000133655	Yes

Notes: Accession is the Swiss-Prot accession number. Ratio (T/N) = Ratio of tumors to controls. Antiangiogenic targets with “Yes” means the protein has been reported or predicted as new targets for cancer antiangiogenic treatment.
